# Establishing the Principal Descriptor for Electrochemical Urea Production via the Dispersed Dual‐Metals Anchored on the N‐Decorated Graphene

**DOI:** 10.1002/advs.202105697

**Published:** 2022-01-31

**Authors:** Changyan Zhu, Miao Wang, Chaoxia Wen, Min Zhang, Yun Geng, Guangshan Zhu, Zhongmin Su

**Affiliations:** ^1^ Institute of Functional Material Chemistry Faculty of Chemistry National and Local United Engineering Laboratory for Power Batteries Northeast Normal University Changchun 130024 China; ^2^ School of Chemistry and Environmental Engineering Changchun University of Science and Technology Changchun 130022 China

**Keywords:** dispersed dual‐metals, electrochemical urea production, linear correlation, MN_3_ moiety, principal descriptor

## Abstract

Urea electrosynthesis under mild conditions starting from the adsorption of inert N_2_ molecules has brought out a promising alternative experimentally to conquer its huge energy consumption in industrial Haber‐Bosch process. The most crucial and inevitable reaction is the formation of urea precursor *NCON from *N_2_ and CO based on the pre‐selected reaction pathway, together with the following protonated processes. It is significant to comprehend their intrinsic intercorrelation and explore the principal descriptor from massive reaction data. Hereby, the authors study the dispersed dual‐metals (homonuclear MN_3_–MN_3_ moiety and heteronuclear MN_3_–M'N_3_ moiety) anchored on N‐doped graphene as electrocatalysts to synthesize urea. Based on the screened out 72 stable systems by ab initio molecular dynamics (AIMD) simulations as the database, six significant linear correlations between the computed Gibbs free energy and other important factors are achieved. Most encouragingly, the principal descriptor (Δ*E*(*NCONH)) is established because 72% low‐performance systems can be filtered out and its effective range (−1.0 eV < Δ*E*E(*NCONH) < 0.5 eV) is identified by eight optimal systems. This study not only suggests that dispersed dual‐metals via MN_3_ moiety can serve as promising active sites for urea production, but also identifies the principal descriptor and its effective range in high‐throughput methods.

## Graphical abstract

Based on the screened out 72 stable systems by ab initio molecular dynamics simulations as the database, six significant linear correlations between the computed Gibbs free energy of these key reactions and other important factors are achieved, and principal descriptor (Δ*E*(*NCONH)) is established, and its effective range (−1.0 eV < Δ*E*(*NCONH) < 0.5 eV) is identified.

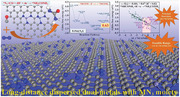

## 1 Introduction

Urea (NH_2_CONH_2_) is one of the most valuable nitrogen fertilizers in agricultural production and the starting materials for the manufacture of plastics, drugs, and other chemical syntheses with an output of 100 million tons per year.^[^ ^1^ ^]^ Currently, the industrial synthesis of urea is dominated by two traditional processes (N_2_ + 6H^+^ + 6e^–^ → 2NH_3_ and 2NH_3_ + CO_2_ → NH_2_CONH_2_ + H_2_O) under harsh reaction conditions (350–550 ℃, 150–350 bar and 150–200 ℃, 150–250 bar, respectively), which requires ≈2% of world's energy consumption annually.^[^ ^2^ ^]^ A promising strategy to reduce energy consumption for urea production is the electrocatalytic reduction of N_2_ and subordinate NO_3_^–^ pass through hydronitrogen intermediates, which can provide an environmental‐friendly and sustainable approach under ambient conditions.^[^ ^3^ ^]^ Last year, the electrochemical urea synthesis with formation rate of 3.36 mmol g^–1^ h^–1^ and Faradic efficiency of 8.92% at −0.4 V versus reversible hydrogen electrode has been achieved via the PdCu alloy nanoparticles on TiO_2_ nanosheets.^[^ ^4^ ^]^ In this year, the urea yield rate has been increased to 5.91 mmol g^–1^ h^–1^ with a Faradic efficiency of 12.55% and 4.94 mmol g^–1^ h^–1^ with a Faradic efficiency of 17.18% at the same applied potential via the Mott–Schottky Bi‐BiVO_4_ heterostructures and the BiFeO_3_/BiVO_4_ heterojunction, respectively.^[^ ^5^ ^]^ Recently, three MBene (Mo_2_B_2_, Ti_2_B_2_, and Cr_2_B_2_) 2D materials have been confirmed to possess superior catalytic activity with the limiting potentials ranging from −0.49 to −0.65 V by means of density functional theory methods and they can significantly suppress the competitive reaction of N_2_ reduction to NH_3_.^[^ ^6^ ^]^ According to these reported studies, the corresponding reaction mechanism through directly CO inserting into *N_2_ to form *NCON intermediate has been proposed to be one feasible reaction pathway for urea formation. Notwithstanding its significance, it is well recognized as a great challenge until now for electrochemical urea production, mainly ascribed to three aspects: 1) the arduous adsorption and ineffective activation of the inert N_2_ molecule on the electrocatalysts,^[^ ^7^ ^]^ 2) the high‐barrier dissociation of the N≡N triple bond and the formation of C—N bond,^[^ ^8^ ^]^ and 3) the inevitable competitive N_2_ reduction reaction (NRR) and hydrogen evolution reaction (HER).^[^ ^4^, ^5^, ^9^ ^]^ Therefore, it is more significant to comprehend their intrinsic intercorrelation and explore the principal descriptor based on massive data of reaction processes, which is prerequisite to overcome trial‐and‐error approaches and high‐efficiency explore other high‐performance electrocatalysts for urea formation using the high‐throughput and machine learning methods.^[^ ^10^ ^]^

Focusing on the aforementioned three main challenges, how to efficiently adsorb and active the inert N_2_ molecule is the most fundamental, because the strong chemisorption of side‐on N_2_ is the ideal initiation to active and dissociate the N≡N bond, and then to form the urea precursor *NCON, and simultaneously to suppress HER side reaction. Inspired by the theoretical recognition that long‐distance dual‐metals can enhance the interaction of catalytic sites with the adsorbed side‐on N_2_ as an excellent stragety to active and polarize the N≡N triple bond due to asymmetric interaction and better favorite orbital matching from double‐sites,^[^ ^11^ ^]^ the systems with dispersed dual‐metals are supposed to possess potential catalytic activity for the urea synthesis.^[^ ^10^, ^12^ ^]^ Experimentally, several instances with dispersed dual‐metals anchored on the N‐decorated graphene have been successfully prepared. The atomically dispersed heteronuclear transition metals (Fe–Ni, Co–Ni, Fe–Co, Co–Cu, Cu–Zn, and Fe–Pt) have been coanchored and constructed the FeN_4_–NiN_4_,^[^ ^13^ ^]^ CoN_4_–NiN_4_,^[^ ^14^ ^]^ FeN_4_–CoN_4_,^[^ ^15^ ^]^ CoN_4_–CuN_4_,^[^ ^16^ ^]^ CuN_4_–ZnN_4_,^[^ ^17^ ^]^ and FeN_4_–PtN_4_ moieties^[^ ^18^ ^]^ on the graphene. Moreover, the synergetic effect of the coexisting dual‐metals promotes the outstanding oxygen reduction reaction activity experimentally. Meanwhile, the homonuclear mixed‐valence CuN_3_ and CuN_4_ dual‐sites have been reported via the Cu metal anchored in MN_3_ moiety and MN_4_ moiety separately.^[^ ^19^ ^]^ Interestingly, these two Cu atoms with different valences play different role in the oxyphosphorylation of alkenes. The lower‐coordinated CuN_3_ site can capture the oxygen and trigger further oxidizing process and the CuN_4_ site can provide moderate adsorption strength for the phosphonyl radicals. In comparison, the lower‐coordinated MN_3_ moiety with out‐of‐plane metal site is expected to strongly react with the N_2_ molecule.^[^ ^22a^ ^]^ Encouragingly, the single atom catalysts (SACs) RuN_3_ and FeN_3_ moieties anchored on graphene have been prepared and confirmed to be the superior electrocatalyst for N_2_ reduction to NH_3_ with the Faradaic efficiency of 29.6% and 39.6%, respectively.^[^ ^20^ ^]^ Therefore, combining complementary merits of the tricoordinated MN_3_ and the long‐distance dual‐metals, hereby the atomically dispersed dual‐metals (homonuclear MN_3_–MN_3_ moiety and heteronuclear MN_3_–M'N_3_ moiety) constructed on N‐decorated graphene (M_2_@N_6_G and MM’@N_6_G in short), are investigated as the latent electrocatalysts on urea production.

Finally, 72 stable systems with long‐distance dispersed dual‐metals, including 13 homonuclear M_2_@N_6_G and 59 heteronuclear MM’@N_6_G, are screened out from the constructed 378 possible systems through the ab initio molecular dynamics (AIMD) simulations. Based on all these stable systems, their reaction mechanism through CO inserting into the activated *N_2_ intermediate is investigated, and subsequently the corresponding intermediates for electrochemical urea synthesis. Three crucial uphill steps in the Gibbs free energy diagrams are specified through investigating and analyzing on these elementary reaction of all 72 stable systems: 1) the C—N coupling reaction, *N_2_ + CO → *NCON; 2) the final hydrogenation step, *NHCONH_2_ + H^+^ + e^–^ → *NH_2_CONH_2_; 3) the desorption process of urea, *NH_2_CONH_2_ → * + NH_2_CONH_2_. More importantly, six significant linear correlations between the computed Gibbs free energy of these elementary reactions and other important factors (integrated‐crystal orbital Hamilton population (ICOHP) values, the adsorption energies of interaction intermediates and d‐band center) are achieved. Most encouragingly, the principal descriptor (Δ*E*(*NCONH)) is established because 72% low‐performance systems can be filtered out and its effective range (−1.0 eV < Δ*E*(*NCONH) < 0.5 eV) is identified via eight optimal systems (homonuclear Co_2_@N_6_G, heteronuclear ScNi@N_6_G, MnFe@N_6_G, FeNi@N_6_G, CoNi@N_6_G, CoRh@N_6_G, RuRh@N_6_G, and RhNi@N_6_G) in our databases.

## 2 Results and Discussion

### 2.1 Screening Stable Electrocatalyst Models of M_2_@N_6_G and MM’@N_6_G

**Figure 1:**
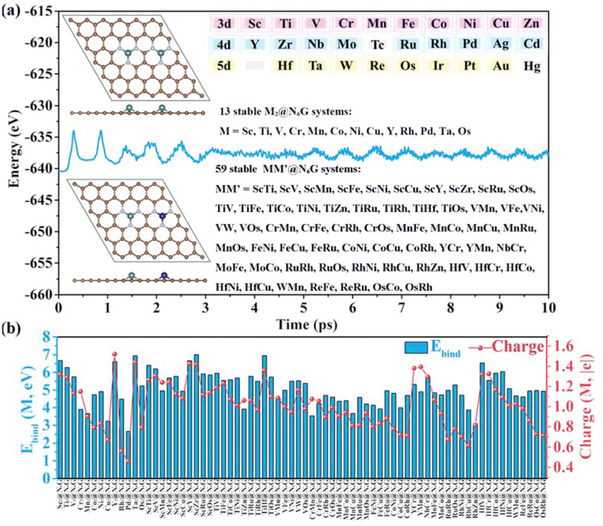
a) Energy profile of AIMD simulation on Pd_2_@N_6_G at ambient temperature in blue curve. The insets including: the representative structures of homonuclear (M_2_@N_6_G) and heteronuclear (MM’@N_6_G) shown in the top left and bottom left (top view and side view); the considered 27 metal atoms listed in the top right; 13 stable homonuclear M_2_@N_6_G listed in center right and 59 stable heteronuclear MM’@N_6_G dual‐metal pairs listed in bottom right. b) Average binding energies of dual‐metals anchored on N‐decorated graphene system and average charge transfer values from dual‐metals to N atoms.

Following the experimental preparation of tricoordinated single‐metal MN_3_ (M = Fe, Ru, Ni) and dispersed dual‐metals CuN_3_–CuN_4_ configurations,^[^ ^19^, ^20^, ^21^ ^]^ and complementary merits of the tricoordinated MN_3_ with sufficient strong interaction with N_2_ molecule and the long‐distance dual‐metals with superior tendency to form side‐on N_2_ configuration,^[^ ^7^, ^10^, ^11^, ^22^ ^]^ the structural models comprising 6 × 6 graphene unit cells with homonuclear MN_3_–MN_3_ moiety and heteronuclear MN_3_–M'N_3_ moiety are constructed, namely M_2_@N_6_G and MM’@N_6_G. In this work, 27 kinds of transition metals (without the radioactive Tc and toxic Hg) in the periodic table of elements are taken into consideration (**Figure** **1**a), generating 27 groups of M_2_@N_6_G and 351 (27×26÷2) groups of MM’@N_6_G systems.

Excellent stability and robust framework are a must to anchor dispersed dual‐metals on the N‐decorated graphene and their potential application as electrocatalysts. To screen out stable systems, the AIMD simulation is performed at ambient temperature (the detail description is given in the Supporting Information). The results indicate that 72 dispersed dual‐metals, including 13 homonuclear dual‐metals and 59 heteronuclear dual‐metals, can be steadily anchored on the tricoordinated N‐decorated graphene after the 10 ps simulation (Figure S1, Supporting Information). Therefore, these 72 systems with good thermal stability are selected out for electrochemical synthesis of urea. Their average binding energies relative to metal atoms range from 2.62 eV (Pd_2_@N_6_G) to 7.01 eV (ScZr@N_6_G), suggesting their good thermodynamic stability and strong interaction between the dispersed dual‐metals and the N‐decorated graphene (Figure 1b). Moreover, the stable energy profile of AIMD simulation on Pd_2_@N_6_G with the weakest binding strength at ambient temperature again proves that the rationality of AIMD simulations to screen out the stable systems (Figure 1a). According to Bader charge analysis,^[^ ^23^ ^]^ there are 60 systems with substantial charge transfer (> 0.80 |e|) from dual‐metals to the adjacent N atoms (Figure 1b). It demonstrates that these dispersed dual‐metals is oxidized to positive valence, which is the intrinsic potentiality to bind with N_2_ molecule.^[^ ^24^ ^]^ Promisingly, the calculated average binding energies of dual‐metals anchored on N‐decorated graphene present an approximate linear correlation with the average charge transfer values from dual‐metals to adjacent N atoms (Figure S2, Supporting Information), indicating that generally the higher charge transfer is more beneficial to enhance the interaction between the anchored dual‐metals and the N‐decorated grapheme to a great extent. The dispersed dual‐metals are separately surrounded by three N atoms with the M—N bond lengths of 1.78 – 2.20 Å (Tables S2 and S3, Supporting Information). In our systems, most of the distances between dispersed dual‐metals (form 3.40 Å for TiHf@N_6_G to 5.14 Å for TiV@N_6_G) are longer than the longest values between the metals in the previous reports (3.42 Å for IrZnB_7_ and 3.42 Å for Bi_2_‐Pc), which can further enhance the adsorption and activation of the side‐on N_2_ molecule.^[^ ^10^, ^11^ ^]^

### 2.2 Investigating the Adsorption and Activation of Side‐on N_2_

**Figure 2:**
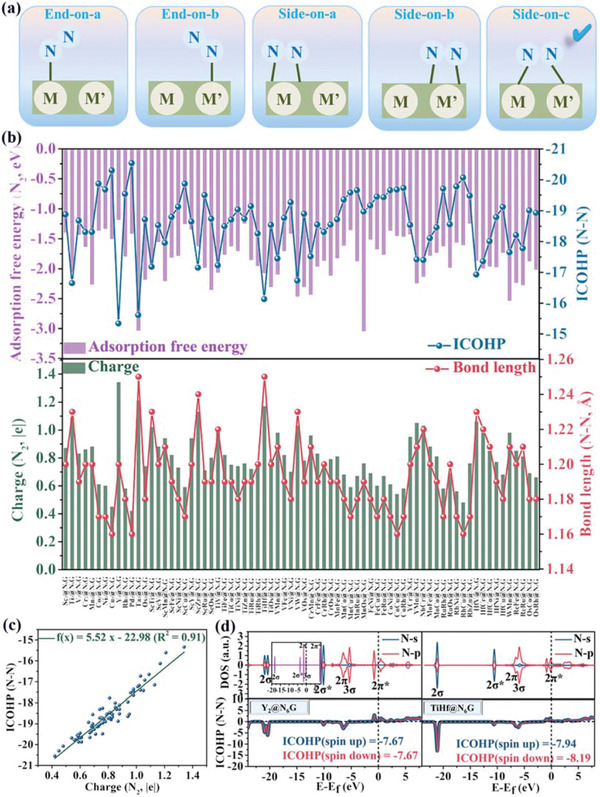
a) Five possible configurations of N_2_ adsorbed on MM’@N_6_G surface. b) Computed adsorption free energies, integrated‐crystal orbital Hamilton population (ICOHP, N—N) values, Bader charges and N—N bond lengths of the side‐on‐c *N_2_. c) Calculated ICOHP values between N—N as a function of the corresponding Bader charges of the side‐on‐c *N_2_. d) Computed partial density of states (PDOS) and ICOHP values of the side‐on‐c *N_2_ on the homonuclear Y_2_@N_6_G and heteronuclear TiHf@N_6_G. The molecule orbital of free N_2_ is inserted in the top left.

The reaction pathways and synthetic mechanisms for urea production are complicated, the potential determining step is various in different reaction pathways on different electrocatalysts. This reaction pathway through CO directly inserting into the activated *N_2_ intermediate (namely NCON pathway) is not the only reaction pathway or the most probable reaction pathway for urea production, but it is still one feasible pathway to achieve urea production.^[^ ^4^, ^6^ ^]^ Hence, this pathway in this investigation is preselected as the feasible reaction mechanism for urea formation using high‐throughput methods. Based on the defined NCON pathway, the effective activation of the inert N≡N triple bond is regarded as its initiation to form the tower‐like urea precursor *NCON.^[^ ^4^ ^]^ Five possible N_2_ adsorption configurations are first designed on 72 stable M_2_@N_6_G and MM’@N_6_G systems, including the end‐on configuration on the single metal‐M (end‐on‐a), end‐on configuration on the single metal‐M’ (end‐on‐b), side‐on configuration on the single metal‐M (side‐on‐a), side‐on configuration on the single metal‐M’ (side‐on‐b) and side‐on configuration on the dual‐metals‐MM’ (side‐on‐c) (**Figure** **2**a). The side‐on‐c *N_2_ is the energetically most favorable configuration on all stable 72 systems through the calculated adsorption energies (Table S4, Supporting Information). Moreover, the adsorption free energies of the side‐on‐c *N_2_ are all below ‐1.00 eV, which is consistent with our initial expectation as a strong chemisorption configuration because tricoordinate and long‐distance pattern with dispersed dual‐metals can collaboratively enhance the interaction between the side‐on *N_2_ and substrates (Figure 2b and Table S5, Supporting Information).

Furthermore, this strong interaction can also be attributed to that the unoccupied d orbitals of anchored dual‐metals can accept the terminal distributed electron of N_2_ molecule, meanwhile occupied d orbitals of the anchored dual‐metals can donate electrons into the 2π* antibonding orbitals of N_2_ molecule. This “acception‐donation” process can be further confirmed by the partial density of states (PDOS) of the adsorbed *N_2_ on 72 stable systems with the side‐on‐c configuration (Figure 2d and Figure S3, Supporting Information). Comparing with the molecular orbitals of free N_2_ molecule, it is easy to find that for the 3*σ* occupied orbitals of the *N_2_ low‐energy shift happen and the corresponding PDOS are more far away from the Fermi level; for their 2π* empty orbitals lower‐energy shift also appears and the corresponding PDOS as a whole move toward or even below the Fermi level. Moreover, the analysis on the PDOS change of the metal d orbitals before and after *N_2_ adsorption are also introduced (Figures S80 and S81, Supporting Information). It is clear that these occupied d orbitals shift to high‐energy region after the N_2_ molecule is adsorbed on the metal atoms. The total d‐band center also shift toward high‐energy region for most systems, expect for the systems containing Cu and Zn with 3d^10^ orbital, which further confirms that the occupied d orbitals of dual‐metals donate electrons into the 2π* antibonding orbitals of N_2_ molecule. As a result, the adsorbed side‐on‐c *N_2_ is stabilized and the inert N≡N triple bond is weakened in nature, which can be proved by the evident charge transfer from the anchored dual‐metals to the adsorbed *N_2_ molecule (0.42–1.34 |e|) and obvious elongated N—N bond lengths (from 1.12 Å in isolated N_2_ to 1.16–2.23 Å, Figure 2b and Table S5, Supporting Information).

The ICOHP after integration up to the Fermi level is also investigated to quantitatively assess the activated N—N binding strength (Figure 2d and Figure S4, Supporting Information).^[^ ^25^ ^]^ Generally, a more negative ICOHP value corresponds to a stronger binding strength. All the computed ICOHP values of N—N bonds in the adsorbed side‐on‐c *N_2_ on 72 stable systems (in the range of −20.30 and −15.34) are higher than that in the free N_2_ molecule (−23.02), indicating that the N≡N triple bond has been successfully activated using the side‐on‐c *N_2_ configuration adsorbed by the dispersed dual‐metals on MM’@N_6_G surface. The highest ICOHP values indicate the N—N bonds of homonuclear Y_2_@N_6_G and heteronuclear TiHf@N_6_G possess the weakest binding strength among them. More interestingly, the ICOHP values of the side‐on‐c *N_2_ present an excellent linear correlation with the charge transfer values from the dispersed dual‐metals to the *N_2_ with *R* ^2^ of 0.91. The accepted charge of *N_2_ may serve as a basic descriptor of their binding strength (Figure 2c), because the values can quantitatively reflect the activation degree of N—N bonds and then help us to develop the potential intercorrelation between the activated strength and the catalytic performance for urea production.

### 2.3 Building the Database about the Catalytic Activity of Urea Production

**Figure 3:**
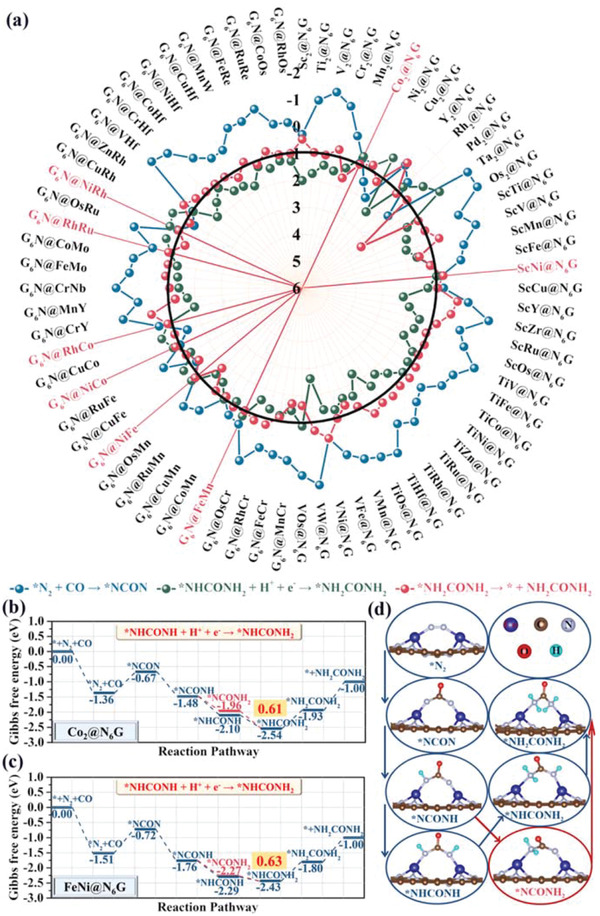
a) Computed Gibbs free energy of three main uphill steps (*N_2_ + CO → *NCON, *NHCONH_2_ + H^+^ + e^–^ → *NH_2_CONH_2_, *NH_2_CONH_2_ → * + NH_2_CONH_2_) on 72 stable systems. The Δ*G* value of 1.00 eV for three main uphill steps is set as the benchmark, marked in black line. The selected out eight optimal systems are marked in red. b) Gibbs free energy diagram for urea production on homonuclear Co_2_@N_6_G. c) Gibbs free energy diagram for urea production on heteronuclear FeNi@N_6_G. d) Optimized structures of various intermediates along the hydrogenation pathway of urea production on Co_2_@N_6_G.

It is significant to build the fundamental database containing crucial factors for urea formation and further explore the potential descriptor based on 72 stable systems using the high‐throughput methods based on the preselected NCON pathway. The whole hydrogenation process of urea formation starting from the adsorbed side‐on‐c *N_2_ and the free CO molecule on 72 stable systems are all systematically investigated and analyzed. The computed total energies, zero‐potential correction energies and entropy contribution energies associated with the reaction intermediates are presented in Tables S6–S77 in the Supporting Information. The entire Gibbs free energy diagrams and optimized structures of various intermediates along the hydrogenation pathway of urea production are illustrated in Figures S5–S76 in the Supporting Information, respectively. Based on the complete Gibbs free energy diagrams for urea production on 72 stable systems based on the preselected NCON pathway, there exist three main uphill steps: 1) the C—N coupling reaction, *N_2_ + CO → *NCON; 2) the final hydrogenation step, *NHCONH_2_ + H^+^ + e^–^ → *NH_2_CONH_2_; 3) the desorption process of urea, *NH_2_CONH_2_ → * + NH_2_CONH_2_. The Gibbs free energy change (Δ*G*) values for three main uphill steps are summarized in **Figure** **3**a and Table S78 in the Supporting Information. In this work, the Δ*G* value of 1.00 eV for three main uphill steps is set as the benchmark. Hence, eight systems are selected out as promising candidates for electrochemical synthesis of urea because their Δ*G* values below 1.00 eV, including homonuclear Co_2_@N_6_G, heteronuclear ScNi@N_6_G, MnFe@N_6_G, FeNi@N_6_G, CoNi@N_6_G, CoRh@N_6_G, RuRh@N_6_G, and RhNi@N_6_G (marked in red in Figure 3a). Subsequently, the Gibbs free energy diagram of these eight optimal systems for urea production is all resimulated in aqueous solvation using the linearized implicit Poisson–Boltzmann solvation model. Three main uphill steps and the potential determining step are the same between the updated results and the original data (Tables S79–S86, Supporting Information). The updated Δ*G* values solely decrease somewhat compared to the values without solvation effects for majority systems. The better catalytic activity for our systems in actual aqueous solvation maybe exists. Moreover, the calculated Δ*G* values for 72 stable systems without solvation effects are adequate to comprehend their intrinsic intercorrelation and explore the principal descriptor. Thus, solvation effects are not adopted in further investigation to economize the computational resource.

The Gibbs free energy diagrams on the representative homonuclear Co_2_@N_6_G and heteronuclear FeNi@N_6_G are presented in Figure 3b,c and the corresponding structures of various intermediates involved in the urea production process on the Co_2_@N_6_G system are given in Figure 3d. The hydrogenation steps of urea production on these two representative systems follow the same reaction route, in which the adsorbed side‐on‐c *N_2_ interact with another reactant CO in the catalytic environment (Heyrovsky‐type mechanism)^[^ ^26^ ^]^ to generate the tower‐like urea precursor *NCON with the positive Δ*G* value of 0.69 eV for Co_2_@N_6_G and 0.79 eV for FeNi@N_6_G. Notably, previous experimental studies reported that the CO molecule with the lone‐pair electrons could strongly bind with the catalytic sites to hinder the adsorption of side‐on‐c *N_2_,^[^ ^4^, ^5^ ^]^ thus it is necessary for improving the yield rate to discover an opportune moment and control the amount of the reactant CO. One N atom of the *NCON intermediate is first attacked by a proton‐electron (H^+^/e^–^) pair to form the *NCONH intermediate with a negative Δ*G* value. The formed *NCONH intermediate can be further converted to distal product *NCONH_2_ or alternative product *NHCONH. Our computations indicate that the *NHCONH intermediate is more favorable than the *NCONH_2_ intermediate due to the more negative Δ*G* value (−0.62 eV for *NHCONH vs −0.48 eV for *NCONH_2_ on Co_2_@N_6_G, −0.53 eV for *NHCONH vs −0.51 eV for *NCONH_2_ on FeNi@N_6_G), which is the same with that on the MBenes.^[^ ^6^ ^]^ Subsequently, the *NHCONH intermediate can be exothermically reduced to *NHCONH_2_. The final hydrogenation step of the *NHCONH_2_ to*NH_2_CONH_2_ intermediate is endothermic and is the potential determining step for urea production. The computed Δ*G* values for this step are 0.61 eV for Co_2_@N_6_G and 0.63 eV for FeNi@N_6_G, respectively, which are comparable to that on the PdCu surface (0.78 eV),^[^ ^4^ ^]^ the Mott–Schottky Bi–BiVO_4_ heterostructures (0.48 eV),^[^ ^5a^ ^]^ the BiFeO_3_/BiVO_4_ heterojunction (0.54 eV),^[^ ^5b^ ^]^ Mo_2_B_2_ (0.49 eV),^[^ ^6^ ^]^ Ti_2_B_2_ (0.65 eV),^[^ ^6^ ^]^ and Cr_2_B_2_ (0.52 eV)^[^ ^6^ ^]^ 2D materials. Different from the exothermic desorption of *NH_2_CONH_2_ from the three experimentally prepared catalyst surfaces (PdCu surface,^[^ ^4^ ^]^ Mott–Schottky Bi–BiVO_4_ heterostructures,^[^ ^5a^ ^]^ and BiFeO_3_/BiVO_4_ heterojunction^[^ ^5b^ ^]^), the desorption of *NH_2_CONH_2_ from our system requires an energy consumption (0.93 eV for Co_2_@N_6_G and 0.80 eV for FeNi@N_6_G). We speculate that this case can be explained by the Lewis theory, in which the interaction between the dual‐metals as Lewis acid with empty d orbitals and the urea as Lewis base with lone pairs of N atom may result in the energy consumption in the desorption process. As another comparison, the urea molecule can be more easily released from our system than the theoretically proposed MBenes materials due to the lower desorption free energies (1.28 eV for Mo_2_B_2_, 1.55 eV for Ti_2_B_2_, and 1.21 eV for Cr_2_B_2_).^[^ ^6^ ^]^

**Figure 4:**
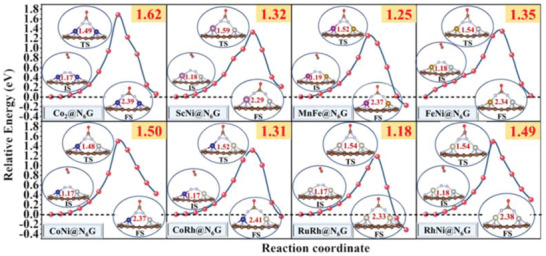
Kinetic energy barrier for C—N bond formation on eight optimal systems. The optimized structures and the N—N distance of the adsorbed side‐on‐c *N_2_ on the initial (IS), transition (TS), and final states (FS) along the C—N bond formation pathway are inserted.

Except for the thermodynamical evaluation of the maximum Δ*G* value of the potential determining step, the kinetic evaluation of the energy barrier for C—N coupling reaction is another indispensable factor to determine the catalytic activity for electrochemical urea production.^[^ ^4^, ^6^ ^]^ Therefore, the kinetic energy barriers for C—N bond formation via the activated side‐on‐c *N_2_ reacting with the free CO molecule are assessed on eight optimal systems (**Figure** **4**). The computed kinetic energy barrier is 1.62 eV for Co_2_@N_6_G, 1.32 eV for ScNi@N_6_G, 1.25 eV for MnFe@N_6_G, 1.35 eV for FeNi@N_6_G, 1.50 eV for CoNi@N_6_G, 1.31 eV for CoRh@N_6_G, 1.18 eV for RuRh@N_6_G, and 1.49 eV for RhNi@N_6_G, respectively, which are slightly higher than that of the Pd–Cu catalyst (0.79 eV) and the MBenes materials (0.58–0.81 eV).^[^ ^4^, ^6^ ^]^ However, it is still accessible at ambient temperature and without applied potential, suggesting that the coupling of *N_2_ and CO molecule on our systems is kinetically feasible. Remarkably, the triangle N—N—C composition is formed with the N—N distance of ≈1.50 Å in the transition state, which can facilitate the formation of multicenter multielectron chemical bonds. Moreover, the delocalized electrons in the triangle N—N—C composition can further weaken the N—N bond and strengthen the C—N bond, which is beneficial to promote the formation of *NCON.

### 2.4 Comprehending the Potential Intrinsic Intercorrelation and Establishing the Principal Descriptor for Electrochemical Urea Production

**Figure 5:**
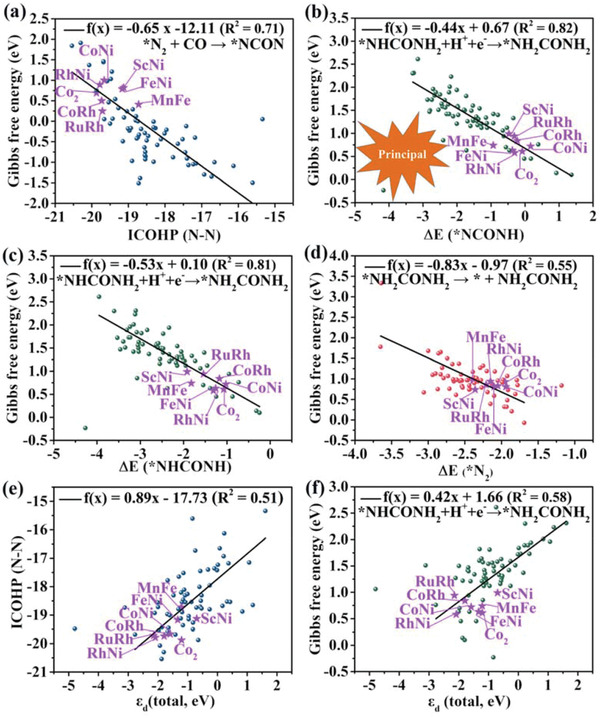
a) Computed Gibbs free energy of *N_2_ + CO → *NCON versus the ICOHP (N—N) values. b) Calculated Gibbs free energy of *NHCONH_2_ + H^+^ + e^–^ → *NH_2_CONH_2_ as a function of the adsorption energy of *NCONH (Δ*E*(*NCONH)). Δ*E*(*NCONH) = *E*(*NCONH) – E* – *E*(N_2_CONH_2_) + 3/2*E*(H_2_). c) Calculated Gibbs free energy of *NHCONH_2_ + H^+^ + e^–^ → *NH_2_CONH_2_ as a function of the adsorption energy of *NHCONH (Δ*E*(*NHCONH)). Δ*E*(*NHCONH) = *E*(*NHCONH) – E* – *E*(N_2_CONH_2_) + *E*(H_2_). d) Calculated Gibbs free energy of *NH_2_CONH_2_ → * + NH_2_CONH_2_ versus the adsorption energy of side‐on‐c *N_2_ (Δ*E*(*N_2_)). e) Calculated ICOHP (N—N) values versus the d‐band center of the total metal orbitals (*ε*_d_(total)). f) Computed Gibbs free energy of *NHCONH_2_ + H^+^ + e^–^ → *NH_2_CONH_2_ versus the d‐band center of the total metal orbitals (*ε*_d_(total)). The eight optimal systems are marked in purple stars. Note that five points with the largest deviation are ignored in linear fitting.

Generally, seven even more reaction intermediates should be considered for urea production, which will consume massive computational resource for large‐scale screening of every potential high‐performance electrocatalyst. Therefore, it is more significant to comprehend the intrinsic intercorrelation based on this database mentioned above and establish the principal descriptor for other possible electrocatalysts. The data including the computed ΔG values of three main uphill steps and other key factors, together with the ICOHP (N—N) values and the adsorption energies of reaction intermediates are collected and possible linear correlations among them are first examined. Four potential linear correlations are identified for three main uphill steps based on the preselected NCON pathway: 1) For the C—N coupling reaction *N_2_ + CO → *NCON, there exists an approximately linear correlation between the ΔG values and the ICOHP (N—N) values (**Figure** **5**a). As expected, this correlation indicates that an increase of ICOHP (N—N) value corresponds to a decrease of Δ*G* value of *N_2_ + CO → *NCON, which further implies that the superior activation of the adsorbed *N_2_ can promote the C—N bond formation. Therefore, the high‐efficiency activation of the adsorbed side‐on *N_2_ is beneficial to the C—N coupling reaction and urea production. 2) For the final hydrogenation step *NHCONH_2_ + H^+^ + e^–^ → *NH_2_CONH_2_, it is always the potential determining step in four proton‐electron transferred processes for urea production on 72 stable systems, expect for the Ta_2_@N_6_G system (*NHCONH + H^+^ + e^–^ → *NHCONH_2_). Generally, the Gibbs free energies of determining step is determined by the adsorption energies of the reaction intermediates.^[^ ^10^, ^22^ ^]^ Therefore, it is crucial to identify the relationship between the Δ*G* values of *NHCONH_2_ + H^+^ + e^–^ → *NH_2_CONH_2_ and the adsorption energies of key reaction intermediates (*NCONH, *NHCONH, *NHCONH_2_, *NH_2_CONH_2_) for rational search of more‐effective catalysts. The results demonstrate that the Δ*G* values of *NHCONH_2_ + H^+^ + e^–^ → *NH_2_CONH_2_ display an excellent scaling relation with the adsorption energies of *NCONH (ΔE(*NCONH)) and *NHCONH (ΔE(*NHCONH)) with the adjusted *R* ^2^ of 0.82 and 0.81, respectively (Figure 5b,c). Therefore, the Δ*E*(*NCONH) and Δ*E*(*NHCONH) values can be considered as the independent variable to describe the catalytic activity for urea production on all 72 stable systems. However, there is no linear correlation between the Δ*G* values and the adsorption energies of *NHCONH_2_ (Δ*E*(*NHCONH_2_)) and *NH_2_CONH_2_ (Δ*E*(*NH_2_CONH_2_)) (Figures S77 and S78, Supporting Information). 3) For the eventual desorption process of urea *NH_2_CONH_2_ → * + NH_2_CONH_2_, the high Δ*G* values can be attributed to the interaction between Lewis acid and Lewis base as mentioned above, thus we further deduce that there is possible correlation between the desorption free energies of *NH_2_CONH_2_ and the adsorption free energies of the side‐on‐c *N_2_. Indeed, a nearly line correlation is found (Figure 5d), suggesting that the balance the adsorption of *N_2_ and desorption of *NH_2_CONH_2_ is a considerable issue. Moreover, the possible linear correlations between the surface charge and the Gibbs free energy of three main uphill steps are also investigated based on the substantial effect on the electrochemical activity of grapheme‐based catalysts (Figure S79, Supporting Information). An excellent linear correction between the average charge transfer from dual‐metals to N atoms and the Gibbs free energy change for the limiting‐potential reaction *NHCONH_2_ + H^+^ + e^–^ → *NH_2_CONH_2_, indicating that the less number of charge transfer from dual‐metals to N atoms will result in the smaller Gibbs free energy change. We speculate that this can be attributed to the less number of charge transfer corresponding to the weaker stability and the higher activity, which is beneficial to adsorb the *NH_2_CONH_2_ intermediate and to decrease the Gibbs free energy change of *NHCONH_2_ + H^+^ + e^–^ → *NH_2_CONH_2_. However, there are no relations between the Gibbs free energy change and another two uphill steps.

The intrinsic properties of the catalytic sites are vital to determine the adsorption energy of key intermediates and impact the catalytic performance for urea production. Here, the primitive PDOS of 72 stable systems are focused and plotted in Figure S79 of the Supporting Information. It is revealed that the occupied and unoccupied d‐orbitals simultaneously near the Fermi level can ensure the “acception‐donation” interaction between the anchored dual‐metals and the reaction intermediates. The d‐band center (*ε*_d_(total)) of the dispersed dual‐metals is also identified as a significant factor to quantitatively reflect the potential correlation between the catalytic activity and the intrinsic electronic properties.^[^ ^27^ ^]^ As expected, an approximate linear correlation between the ICOHP (N—N) values and the *ε*_d_(total) values of the anchored dual‐metals is found (Figure 5e), suggesting the more positive *ε*_d_(total) value corresponding to the less negative ICOHP (N—N) value. In other words, the more positive *ε*_d_(total) is more beneficial to donate electrons to the adsorbed side‐on‐c *N_2_ to weaken the N≡N triple bond. Furthermore, there is also an approximate linear correlation between the computed Δ*G* values of *NHCONH_2_ + H^+^ + e^–^ → *NH_2_CONH_2_ and the *ε*_d_(total) of the anchored dual‐metals (Figure 5f), whereas no relations between the computed ΔG values of another two uphill steps and the *ε*_d_(total) exist (Figures S80 and S81, Supporting Information).

Most importantly, the distribution of eight optimal systems (the purple stars) concentrated on a small range in these linear corrections implies that the principal descriptor can be established for electrochemical urea production. Marked by eight optimal systems, the optimal adsorption energy of the side‐on‐c *N_2_ ranging from −2.39 to −1.94 eV (corresponding to the Δ*G* value from −1.82 to −1.36 eV) is the decisive factor to appropriately active the N≡N triple bond, and then to facilitate the C—N coupling reaction, and finally to accelerate desorption of *NH_2_CONH_2_ product (Figure 5a,d). The adsorption energies of *NCONH from −0.93 to 0.16 eV and *NHCONH from −1.92 eV to – 1.02 eV correspond to the Δ*G* values of *NHCONH_2_ + H^+^ + e^–^ → *NH_2_CONH_2_ on the eight optimal systems (Figure 5b,c). The corresponding *ε*_d_(total) values of eight optimal systems are ranging from −2.09 eV to −0.70 eV (Figure 5e,f).

According to the six proposed linear correlations in Figure 5 and their corresponding correlation coefficient, the ΔE(*NCONH) ranging from the −1.0 to 0.5 eV could be utilized as the principal descriptor to screen the potential electrocatalytsts for urea production. Besides eight investigated optimal systems in the range (−1.0 eV < Δ*E*(*NCONH) < 0.5 eV), 12 other systems (Ni_2_@N_6_G, Rh_2_@N_6_G, ScCu@N_6_G, VNi@N_6_G, MnCo@N_6_G, MnCu@N_6_G, MnRu@N_6_G, FeCu@N_6_G, FeRu@N_6_G, CoCu@N_6_G, RhCu@N_6_G, and RhZn@N_6_G) are also in the same range. Even so, this Δ*E*(*NCONH) descriptor can filter out 72% low‐performance systems through this proposed standard because of 52 systems out of the range. The electrocatalytic performance of the other twelve systems in the range is also reinspected. Excitedly, the corresponding maximum Δ*G* values are all below 1.50 eV. These external data also strongly support the Δ*E*(*NCONH) as the principal descriptor, which can well scale with the electrocatalytic performance for urea production.

Remarkably, the preselected NCON pathway in this study could not contain all possible pathways and also might not be the only mechanism for other electrocatalyst, but it is one feasible reaction pathway in principle through directly CO inserting into *N_2_ intermediate for urea formation. The more complete reaction mechanisms for urea production are on the developing way and need to be considered and inspected in future, especially for other specific electrocatalyst. The final hydrogenation step as the limiting‐potential step might also be amended when the other reaction mechanisms are considered, but its relative Δ*G* value still remarkably determines the final Faradic efficiency. The high‐throughput strategy in this study is carried out to obtain the high‐performance urea electrocatalysts based on directly CO inserting into activated *N_2_ intermediate when this pathway is feasible, which has consumed huge computing resources and time for 72 candidates in one database. Fortunately, designing the dispersed dual‐metals in graphene as electrocatalyst through the NCON pathway is available and this conclusion based on these 72 stable systems is also significant to explore other excellent urea catalysts with dual‐metal sites.

### 2.5 Inspecting the Blockage of Out‐of‐Plane Dual‐Metals by *O/*OH/*OH_2_ and Evaluating Catalytic Selectivity of Urea Production versus HER and NRR

**Figure 6:**
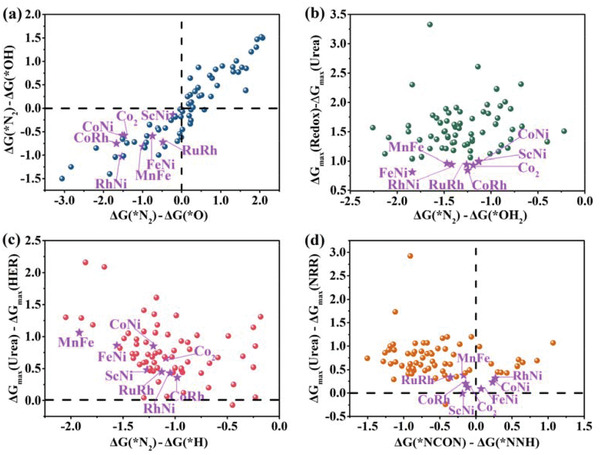
a) Computed Gibbs free energy differences between *N_2_ and *OH (Δ*G*(*N_2_) – Δ*G*(*OH)) versus that between *N_2_ and *O (Δ*G*(*N_2_) – Δ*G*(*O)). b) Computed Gibbs free energy differences between redox reaction and urea production (Δ*G*(Redox) – Δ*G*(Urea)) versus the computed Gibbs free energy differences between *N_2_ and *OH_2_ (Δ*G*(*N_2_) – Δ*G*(*OH_2_)). c) Computed Gibbs free energy differences between urea production and H_2_ production (Δ*G*_max_(Urea) – Δ*G*_max_(HER)) versus that between *N_2_ and *H (Δ*G*(*N_2_) – Δ*G*(*H)). d) Computed Gibbs free energy differences between urea production and N_2_ reduction (Δ*G*_max_(Urea) – Δ*G*_max_(NRR)) versus that between *NCON and *NNH (Δ*G*(*NCON) – Δ*G*(*NNH)). The eight optimal systems are marked in purple stars.

It is well known that the out‐of‐plane dual‐metals can be easily covered by the oxygen and hydroxyl groups or water molecule (*O, *OH, *OH_2_), which will hinder the *N_2_ adsorption and urea production.^[^ ^6^, ^10^, ^21^, ^28^ ^]^ Hence, the adsorption free energy differences between the targeted *N_2_ and the unfavorable *O/*OH/*OH_2_ (Δ*G*(*N_2_) – Δ*G*(*O), Δ*G*(*N_2_) – Δ*G*(*OH), Δ*G*(*N_2_) – Δ*G*(*OH_2_) are shown in **Figure** **6**a,b, and the optimized structures and their computed ΔG(*O/*OH/*OH_2_) values are also summarized in Figures S82–S84 and Table S87 in the Supporting Information. The corresponding adsorption free energy differences for 72 stable systems are all negative, suggesting that the targeted *N_2_ is preferred to be adsorbed on these dual‐metals. The Δ*G* values in the process of the adsorbed *O/*OH converted to *OH_2_ and the OH_2_ molecule removed from these systems are also computed (Table S87, Supporting Information). The results indicate that the maximum Δ*G* values of the redox reaction are all higher than that of urea production on 72 stable systems based on the positive Δ*G*(Redox) – Δ*G*(Urea) values (Figure 6b). Therefore, dispersed dual‐metals via MN_3_ moiety on the N‐doped graphene through these 72 systems possess superior electrochemical capacity for urea production against surface oxidation or hydroxylation and the dispersed dual‐metals are preferred to be occupied by the targeted side‐on‐c *N_2_.

The HER and NRR are both competitive reactions to the urea production, especially the HER side reaction.^[^ ^4^ ^]^ As a common recognition for the HER side reaction that the *H is more favorable to be adsorbed than the *N_2_ on most metal surfaces, the side‐on‐c *N_2_ can preferentially occupy the dual‐metals sites on 72 stable systems due to the negative adsorption free energy differences between *N_2_ and *H (Δ*G*(*N_2_) – Δ*G*(*H)) (Figure 6c, the optimized structures and the computed Δ*G*(*H) shown in Figure S85 and Table S87 of the Supporting Information). It can be attributed to the structural characteristic of the tricoordinated MN_3_ moiety and long‐distance dispersed dual‐metals. Unfortunately, the Gibbs free energy differences between urea production and H_2_ production (Δ*G*_max_(Urea) – Δ*G*_max_(HER)) are all positive (Figure 6c) on eight optimal systems, implying that the lower applied potential is needed for HER than the urea formation. In other words, the H_2_ molecule would be the main side product when the H atom is adsorbed on the dual‐metals, which is consistent with the experimental result that the reaction is always dominated by HER reaction on the PdCu alloy nanoparticles.^[^ ^4^ ^]^ However, the HER side reaction can be efficiently suppressed experimentally by adjusting the electrolyte pH in a neutral condition. It is thus possible to maximize the urea production at the real experimental adjustments and suppress the influence of the HER side reaction by the applied potentials.^[^ ^29^ ^]^

To evaluate the selectivity of our systems toward urea production comparing with the NRR competitive reaction, the possible reaction mechanisms and intermediates starting from the side‐on‐c *N_2_ in the NRR process^[^ ^30^ ^]^ are all considered (Figures S86–S157, Supporting Information). The Δ*G* values of the first hydrogenation step *N_2_ + H^+^ + e^–^ → *NNH, the potential determining steps and the corresponding maximum ΔG values are summarized in Table S88 in the Supporting Information. The Gibbs free energy differences between generating the crucial intermediates *NCON for urea production and *NNH for NH_3_ production (ΔG(*NCON) – ΔG(*NNH)) are computed. And then, the Gibbs free energy differences of urea production and N_2_ reduction to NH_3_ (Δ*G*_max_(Urea) – ΔG_max_(NRR)) are also computed to select out the lowest‐energy reaction pathway and the most possible primary product (Figure 6d). The more negative Gibbs free energy differences represent the better selectivity for urea production. As a result, only ScNi@N_6_G system is screened out to meet the harsh criteria. Note that the Δ*G*_max_(Urea) – Δ*G*_max_(NRR) values for the remaining seven optimal systems are ranging from 0.09 eV (FeNi@N_6_G) to 0.38 eV (MnFe@N_6_G). These low Δ*G*_max_(Urea) – Δ*G*_max_(NRR) values indicate that the primary product is easily influenced by the applied potential, which can account for the experimental observation that the faradic efficiency for NH_3_ and urea formation at different applied potential is variable.^[^ ^4^, ^5^ ^]^ Therefore, the applied potential is an important parameter to determine the faradic efficiency of urea production.

## 3 Conclusions

In summary, 72 stable systems with long‐distance dispersed dual‐metals anchored on the N‐doped graphene, including 13 homonuclear M_2_@N_6_G and 59 heteronuclear MM’@N_6_G, are screened out from the constructed 378 systems through the AIMD simulations. Their entire catalytic mechanism of these stable 72 systems for electrochemical urea formation starting from the lowest‐energy absorption of side‐on *N_2_ and free CO molecule through the preselected NCON pathway are all computed in detail to build the database containing the corresponding reaction intermediates, the computed Gibbs free energy of every elementary reaction, and their characteristic parameters of electronic properties (PDOS, d‐band center, ICOHP values). First, three main uphill steps in the Gibbs free energy diagrams are specified: 1) the C—N coupling reaction, *N_2_ + CO → *NCON; 2) the final hydrogenation step, *NHCONH_2_ + H^+^ + e^–^ → *NH_2_CONH_2_; 3) the desorption process of urea, *NH_2_CONH_2_ → * + NH_2_CONH_2_. Second, eight optimal systems are selected out as promising electrocatalysts for urea production containing homonuclear Co_2_@N_6_G, heteronuclear ScNi@N_6_G, MnFe@N_6_G, FeNi@N_6_G, CoNi@N_6_G, CoRh@N_6_G, RuRh@N_6_G, RhNi@N_6_G because of all computed Gibbs free energy below 1.0 eV and the kinetic energy barrier for C—N coupling reaction in the range of 1.18–1.62 eV. Thirdly, according to the Gibbs free energies of the three uphill steps and key factors (ICOHP values, the adsorption energies of interaction intermediates and d‐band center), six potential linear correlations are discovered. Most importantly, the principal descriptor (Δ*E*(*NCONH)) is established to screen other potential electrocatalysts for urea production in future, and its effective range (−1.0 eV < Δ*E*(*NCONH) < 0.5 eV) is identified via our eight optimal systems and twelve other systems in this range. This study not only suggests that dispersed dual‐metals via MN_3_ moiety as high‐performance active sites for urea production but also identifies the principal descriptor and its effective range to screen out and hunt other excellent urea catalysts in the high‐throughput methods.

## 4 Experimental Section

All the computations were performed by spin‐polarized density functional theory (DFT)^[^ ^31^ ^]^ using the Vienna ab initio simulation package.^[^ ^32^ ^]^ The Perew–Burke–Ernzerhof functional within the generalized gradient approximation was adopted to present the electron exchange and correlation effects, while the projector‐augmented‐wave potentials was used to treat the core electrons.^[^ ^33^ ^]^ The Grimme's semiempirical DFT‐D3 method of dispersion correction was utilized for properly describing the van der Waals (vdW) interactions between reaction intermediates and catalysts.^[^ ^34^ ^]^ The plane‐wave cutoff energy of 500 eV was used for all the computations, and the Brillouin zone was sampled by a 3 × 3 × 1 Monkhorst–Pack *k*‐points mesh. The convergence criteria were set at 10^–5^ eV for total energy change and 0.02 eV Å^–1^ for the maximum forces on each atom, respectively. The vacuum region was added to 15 Å in the vertical direction to prevent interactions between periodic images. The solvation effect was simulated using the linearized implicit Poisson‐Boltzmann solvation model, and the dielectric constant of 78.5 for water and a Debye length of 3.0 Å to simulate 1 mol of electrolyte solution of monovalent cations.^[^ ^35^ ^]^ The thermal stability of the anchored dual‐metals was evaluated by AIMD simulations at 300 K under the constant volume and temperature (NVT) ensemble with a time step of 1.0 fs and lasting for 10 ps.^[^ ^36^ ^]^

The Gibbs free energy diagram of each elementary step in the electrochemical synthesis of urea was obtained by the computational hydrogen electrode model proposed by Nørskov et al.^[^ ^37^ ^]^ The transition states and kinetic barriers for the C—N coupling reaction were searched by the climbing‐image nudges elastic band method.^[^ ^38^ ^]^ The computational details of stability evaluation, adsorption energy, Gibbs free energy and effects of coulomb interaction can be found in the Supporting Information.

## Conflict of Interest

The authors declare no conflict of interest.

## Supporting information

Supporting Information

## Data Availability

The data that support the findings of this study are available from the corresponding author upon reasonable request.
