# Supplementary material for: Establishing the Principal Descriptor for Electrochemical Urea Production via the Dispersed Dual‐Metals Anchored on the N‐Decorated Graphene
Source: Adv Sci (Weinh). 2022 Jan 31;9(10):2105697. doi: 10.1002/advs.202105697 (PMC8981460; doi:10.1002/advs.202105697)
Supplement: Supplementary file 1 — Supporting Information [file ADVS-9-2105697-s001.pdf]

ADVANCED SCIENCE
Open Access

Supporting Information

for *Adv. Sci.*, DOI 10.1002/advs.202105697

Establishing the Principal Descriptor for Electrochemical Urea Production via the Dispersed Dual-Metals Anchored on the N-Decorated Graphene

Changyan Zhu, Miao Wang, Chaoxia Wen, Min Zhang, Yun Geng, Guangshan Zhu* and Zhongmin Su**

ADVANCED SCIENCE
Open Access

Supporting Information

for *Adv. Sci.*, DOI: 10.1002/advs.202105697

Establishing the Principal Descriptor for Electrochemical Urea Production via the Dispersed Dual-Metals Anchored on the N-Decorated Graphene

Changyan Zhu, Miao Wang, Chaoxia Wen, Min Zhang, Yun Geng, Guangshan Zhu,* Zhongmin Su**

Establishing the Principal Descriptor for Electrochemical Urea Production via the Dispersed Dual-Metals Anchored on the N-Decorated Graphene

Changyan Zhu, Miao Wang, Chaoxia Wen, Min Zhang,*

Yun Geng, Guangshan Zhu,* Zhongmin Su*,

Dr. C. Zhu, C. Wen, M. Wang, Dr. M. Zhang, Dr. Y. Geng, Prof. G. Zhu, Prof. Z. Su

Institute of Functional Material Chemistry, Faculty of Chemistry, National & Local United Engineering Laboratory for Power Batteries, Northeast Normal University, Changchun 130024, China

Email: mzhang@nenu.edu.cn; zhugs100@nenu.edu.cn; zmsu@nenu.edu.cn

Prof. Z. Su

School of Chemistry and Environmental Engineering, Changchun University of Science and Technology, Changchun 130022, China

Table of contents

(Total Number of Pages:225, Total of Number of Tables:88, Total of Figures:159)

1. Computational details	Page S4
2. Total energy (E_{tot}), zero-potential correction energy (Ezpe), entropy contribution (TS, T=298.15 K) of free molecules from NIST database. (Table S1)	Page S9
3. Structural and electronic properties of 72 stable systems, including average bond lengths of M-N and M-M, average binding energies of dual-metals anchored on the N-decorated graphene, magnetic moment and average charge transfer from dual-metals to N atoms. (Table S2 and S3)	Page S9
4. Calculated adsorption energies of five possible configurations of N_2 on 72 stable systems. (Table S4)	Page S12
5. Computed adsorption free energies, integrated-crystal orbital Hamilton population (ICOHP) values for N-N bonds, Bader charges and N-N bond lengths of the adsorbed side-on-c N_2 on 72 stable systems. (Table S5)	Page S14
6. Computed total energy (E_{tot}), zero-potential correction energy (E_{zpe}) and entropy contribution (TS, T=298.15 K) of the optimized intermediates for urea production on 72 stable systems. (Table S6-S77)	Page S16
7. Computed Gibbs free energy of three main uphill steps (*N_2 + CO → *NCON, *$NHCONH_2$ + H^+ + e^- → *NH_2CONH_2, *NH_2CONH_2 → * + NH_2CONH_2) on 72 stable systems. (Table S78)	Page S40
8. Computed Gibbs free energy of each elementary step for urea formation on eight optimal systems without and with solvation effects. (Table S79-S86)	Page S42
9. Computed adsorption free energies of hydrogen atom (ΔG(*H)), oxygen atom (ΔG(*O)), hydroxyl group (ΔG(*OH)), water molecule (ΔG(*OH_2)), and the Gibbs free energies of *O + H^+ +e^- → *OH and *OH + H^+ +e^- → *OH_2 on 72 stable systems. (Table S87)	Page S45
10. Computed Gibbs free energies of the first hydrogenated step *N_2 + H^+ + e^- → *NNH, the potential determined steps (PDS) and the corrsponding ΔG values (ΔG (PDS)) for N_2 reduction to NH_3 on 72 stable systems. (Table S88)	Page S47
11. Equilibrium structures of our systems at ambient temperatures through 10 *ps* AIMD simulations. (Figure S1)	Page S49
12. Average binding energies of dual-metals anchored on N-decorated graphene versus average charge transfer from dual-metals to N atoms. (Figure S2)	Page S52

13. Computed partial density of states (PDOS) of side-on-c N_2 adsorbed on our systems using PBE functional. The molecular orbitals of free N_2 molecule are inset. The Fermi level is set to zero. (Figure S3)	Page S53
14. Computed crystal orbital Hamilton populations (COHPs) for the N-N bonds on 72 stable systems. (Figure S4)	Page S56
15. Gibbs free energy diagram for urea production and the optimized structures of various intermediates along the hydrogenation pathway of urea production on 72 stable systems. (Figure S5-S76)	Page S59
16. Calculated Gibbs free energy of $*NHCONH_2 + H^+ + e^- \rightarrow *NH_2CONH_2$ versus the adsorption energy of $*NHCONH_2$ ($\Delta E(*NHCONH_2)$) and $*NH_2CONH_2$ ($\Delta E(*NH_2CONH_2)$) on 72 stable systems. (Figure S77 and S78)	Page S131
17. Computed Gibbs free energy of $*N_2 + CO \rightarrow *NCON$, $*NHCONH_2 + H^+ + e^- \rightarrow *NH_2CONH_2$ and *NH2CONH$_2$ $\rightarrow * + NH_2CONH_2$ versus the average charge transfer from dual-metals to N atoms. (Figure S79)	Page S133
18. Computed partial density of states (PDOS) of 72 stable systems. (Figure S80 and Figure S81)	Page S134
19. Calculated Gibbs free energy of $*N_2 + CO \rightarrow *NCON$ and $*NH_2CONH_2 \rightarrow * + NH_2CONH_2$ versus the d-band center of the total metal orbitals (ε_d(total)). (Figure S82 and S83)	Page S140
20. Optimized structures and the corresponding adsorption free energies of oxygen atom ($\Delta G(*O)$), hydroxyl group ($\Delta G(*OH)$), water molecule ($\Delta G(*OH_2)$) and hydrogen atom ($\Delta G(*H)$) on 72 stable systems. (Figure S84-S87)	Page S141
21. Gibbs free energy diagram and the optimized structures of various intermediates along the hydrogenation pathway of N_2 reduction to NH_3 production on 72 stable systems (Figure S88-S159)	Page S153
22. References	Page S225

Computational details

Stability evaluation: The cohesion between the anchored dual-metals and the N-decorated graphene system is crucial for electrocatalysts, which can be evaluated by various parameters in the previous reported literatures, including:

(1) the binding energy (E_{bind}), which is defined as:[1]

$$E_{bind} = (E_{N_6G} + E_M + E_{M'} - E_{MM'@N_6G})/2$$

where E_{N_6G} and $E_{MM'@N_6G}$ are the energies of the N-decorated graphene without and with anchored dual-metals, E_M and $E_{M'}$ are the energies of an isolated metal trimer in vacuum, respectively. The more positive E_{bind} value indicates the stronger cohesion between the anchored dual-metals and the N-decorated graphene monolayer.

(2) the cohesive energy (E_{coh}), which is defined as:[1]

$$E_{coh} = (E_{bulk(M)} - nE_M)/n$$

where $E_{bulk(M)}$ and E_M are the energies of the metal atom in its most stable bulk structure and the isolated metal trimer in vacuum.

(3) the formation energy (E_f), which is defined as:[2]

$$E_f = (E_{MM'@N_6G} - E_{N_6G} - E_{bulk(M)} - E_{bulk(M')})/2$$

where E_{N_6G} and $E_{MM'@N_6G}$ are the energies of the N-decorated graphene without and with anchored dual-metals, $E_{bulk(M)}$ and $E_{bulk(M')}$ are the total energies of the metal atom in its most stable bulk structure, respectively. The system with $E_f < 0$ eV is considered to be thermodynamically stable.

(4) the dissolution potential (U_{diss}), which is defined as:[2,3]

$$U_{diss} = U^0_{diss}\ (\text{metal,bulk}) - E_f/eN_e$$

where U^0_{diss} (metal,bulk) is the standard dissolution potential of bulk metal, N_e is the number of electrons involved in the dissolution. The more positive U_{diss} ($U_{diss} > 0$) value suggests that the dissolution of metal atoms can be avoided under the electrochemical conditions.

(5) *ab initio* molecular dynamics (AIMD) simulations,[4] which is considered to evaluate the thermal stability of the anchored dual-metals. The structural integrity can be well maintained at ambient temperature through the 10 ps AIMD simulation, which demonstrates the system possesses good thermal stability.

However, above five evaluation parameters are not appropriate foa all systems at present. For example, the computed U_{diss} value of Fe@g-N_3P is negative (-0.22), yet the Fe@g-N_3P has been prepared and determined using systematic X-ray adsorption fine structure analysis and displays excellent ORR catalytic activity.[5] In our opinion, the contradiction is due to the atomic weaker interaction between the Fe and P than that between Fe and N. Hence, searching for an appropriate stability evaluation parameter for our systems is the prerequisite to determine the structural stability. In this work, the prepared Fe@N_3G,[6] Ni@N_3G[7] and Ru@N_3G[8] in experiment was selected to verify the suitability of the above five stability evaluation parameters due to the identical MN_3 moiety. According to the computation results (Table S0), it is apparent that the formation energy and the dissolution potential are both not appropriate parameters for the stability evaluation of the MN_3 moiety. However, the structural integrity of the Ni@N_3G can be well maintained at ambient temperatures through the 10 ps AIMD

simulation, which is consistent with its actural preparation in experiment. Therefore, the structural integrity of our systems at ambient temperatures through the 10 ps AIMD simulation is considered to be the criterion to evaluate the structural stability.

Table S0. Computed binding energy (E_{bind}), cohesive energy, formation energy (E_f) and dissolution potential (U_{diss}) of the prepared Fe@N_3G, Ni@N_3G and Ru@N_3G in experiment.

systems	E_{bind} (eV)	E_{coh} (eV)	E_f (eV)	U_{diss} (V)
Fe@N_3G	4.77	-5.59	0.81	-0.86
Ni@N_3G	5.10	-5.69	0.59	-0.56
Ru@N_3G	5.48	-7.72	2.24	-0.66

Electrochemical reaction computations: The adsorption energy (E_{ads}) was computed to assess the ineraction between the adsorbed N_2 molecule and the MM'@N_6G systems by

$$E_{ads} = E_{total} - E_{MM'@N_6G} - E_{N_2}$$

in which E_{total} and $E_{MM'@N_6G}$ represent the total energies of the MM'@N_6G systems with and without adsorption of N_2 molecule, respectively; E_{N_2} is the energy of a N_2 molecule in vacuum.

The Gibbs free energy change (ΔG) for each elementary step in the electrochemical synthesis of urea was obtained by the compatational hydrogen electrode (CHE) model proposed by Nørskov et al,[9] which can be computed by:

$$\Delta G = \Delta E + \Delta E_{zpe} - T\Delta S + \Delta G_U + \Delta G_{pH}$$

where ΔE is the electronic energy difference between the free standing and adsorption states of reaction intermediates, which can be directly obtained from DFT calculations.

ΔE_{zpe} and ΔS are the changes between the adsorbed species and the gas phase molecules in zero-point energy and entropy, respectively, which can be obtained from the vibrational frequency. For each adsorbed species, its E_{zpe} and TS can be calculated by the following equations,[10] respectively:

$$E_{zpe} = 1/2\Sigma h\upsilon_i$$

$$TS = k_B T[\sum_i \ln\left(\frac{1}{1\text{-}e^{h\nu_i/k_B T}}\right) + \sum_i \frac{h\nu_i}{k_B T} \frac{1}{e^{h\nu_i/k_B T}\text{-}1}+1]$$

where k_B is the Boltzmann's constant; h represents the Planck constant; ν_i denotes the frequency of the normal mode of the adsorbed species; T is the thermodynamic temperature of the reaction (298.15 K). Moreover, the corresponding E_{zpe} and S of the gas phase molecules are taken from the NIST database (Table S1).[11] Furthermore, the corrections for zero point energy and entropy of reaction intermediates are only needed for the adsorbed species because the contribution of the substrate can be offset, which has been verified in the previous report. ΔG_U is the free energy contribution related to applied potential U, and U is the operating electrochemical potential relative to the reversible hydrogen electrode (RHE), which is determined by the potential-limiting step with the most positive ΔG_{max} value ($U = -\Delta G_{max}/e$). $\Delta G_{pH} = 2.303\times k_B\times T\times pH$, which represents the free energy correction due to the variations in the H concentration. Moreover, the pH will not change the overpotential, and in this work the value of pH was assumed to be zero in a highly acidic solution.

Effects of Coulomb interaction: Although the inclusion of the Hubbard-U term, or using the DFT+U approach, normally give more accurate results for the systems with

highly localized orbitals, the DFT+U approach also suffers from a strong (linear) dependence of the energetics on the choice of the value of the parameter U, and on the choice of the localized projector functions that enter the definition of the U-dependent energy term. For example, the reduction energy (ΔH) of $CeO_2 \rightarrow Ce_2O_3$ process can vary between -5.1 (U=0 eV) and -1.9 eV (U=5.0 eV) using the DFT+U method,[12] while the GGA-PBE value of -4.18 eV is in good agreement with the experimental measurements (-3.57 to -4.03 eV). Particularly, the U-value is usually chosen based on its accuracy in reproducing the electronic structures (i.e., experimental band gap) of the bulk materials. However, to simulate catalysts, it is better to choose U by fitting the electronic energy of their redox reaction.[13] Our specific case involves complex surface-adsorbate interactions, thus, it is not correct to describe the reaction energetics using the bulk properties-based U values in a locally changing surface environment. Note that the results based on the GGA-PBE (the method used in this work) showed very good high performance in understanding the reaction mechanisms and activity trends observed in experiments.[14] Moreover, the computed theoretical limiting potential as well as the potential-limiting step obtained by PBE+U were in good agreement with the PBE results in recent reports.[2b,15] Therefore, we did not use DFT+U to consider the effect of the highly localized orbitals of the metal atoms in this work.

Table S1. Total energy (E_{tot}), zero-potential correction energy (Ezpe), entropy contribution (TS, T=298.15 K) of free molecules from NIST database.

Speciese	E_{tot} (eV)	E_{zpe} (eV)	TS (eV)
H_2(g)	-6.67	0.27	0.40
H_2O(l)	-14.54	0.56	0.58
N_2(g)	-16.32	0.14	0.59
NH_3(g)	-19.73	0.60	0.89
CO(g)	-14.78	0.13	0.61

Table S2. Structural and electronic properties of 13 stable $M_2@N_6G$ systems, including average bond lengths of M-N ($d_{M\text{-}N}$, Å) and M-M ($d_{M\text{-}M}$, Å), average binding energies of homonuclear dual-metals anchored on the N-decorated graphene (E_{bind}, eV), magnetic moment (mag, μB) and average charge transfer from dual-metals to N atoms (CT, |e|).

system	$d_{M\text{-}N}$	$d_{M\text{-}M}$	E_{bind}	Mag	CT
$Sc_2@N_6G$	2.06	3.86	6.69	0.10	1.32
$Ti_2@N_6G$	1.98	4.42	6.28	0.67	1.29
$V_2@N_6G$	1.93	4.63	5.75	2.40	1.13
$Cr_2@N_6G$	1.90	4.54	3.90	2.86	1.15
$Mn_2@N_6G$	1.90	4.94	3.66	2.38	0.90
$Co_2@N_6G$	1.85	4.66	4.74	1.75	0.79
$Ni_2@N_6G$	1.85	4.68	4.92	1.15	0.83
$Cu_2@N_6G$	1.91	4.72	3.24	0.00	0.67
$Y_2@N_6G$	2.20	3.80	6.62	0.17	1.52
$Rh_2@N_6G$	2.02	4.68	4.49	0.49	0.56
$Pd_2@N_6G$	2.19	4.90	2.62	0.58	0.46
$Ta_2@N_6G$	2.00	4.54	6.95	0.65	1.44
$Os_2@N_6G$	1.95	4.47	5.24	0.09	0.79

Table S3. Structural and electronic properties of 59 stable MM'@N_6G systems, including average bond lengths of M/M'-N (d_{M-N}, $d_{M'-N}$, Å) and M-M ($d_{M-M'}$, Å), average binding energies of heteronuclear dual-metals anchored on the N-decorated graphene (E_{bind}, eV), magnetic moment (mag, μB) and average charge transfer from dual-metals to N atoms (CT, |e|).

system	d_{M-N}	$d_{M'-N}$	$d_{M-M'}$	E_{bind}	Mag-M	Mag-M'	CT-M	CT-M'
ScTi@N_6G	2.05	1.95	4.17	6.40	0.00	1.24	1.38	1.15
ScV@N_6G	2.05	1.90	4.50	6.20	0.00	2.34	1.49	1.12
ScMn@N_6G	2.05	1.78	4.54	4.96	0.00	0.70	1.49	0.99
ScFe@N_6G	2.05	1.86	4.52	5.68	0.00	2.95	1.51	0.97
ScNi@N_6G	2.06	1.82	4.47	5.78	0.00	0.96	1.52	0.73
ScCu@N_6G	2.05	1.88	4.52	4.93	0.00	0.00	1.53	0.63
ScY@N_6G	2.06	2.20	3.85	6.66	0.00	0.00	1.27	1.58
ScZr@N_6G	2.07	2.13	3.56	7.01	0.17	0.19	1.35	1.49
ScRu@N_6G	2.05	1.92	4.51	5.91	0.00	0.00	1.50	0.73
ScOs@N_6G	2.05	1.91	4.49	5.85	0.00	0.00	1.50	0.78
TiV@N_6G	1.94	1.90	5.14	5.96	1.29	2.37	1.24	1.14
TiFe@N_6G	2.01	1.87	4.55	5.54	0.44	3.10	1.46	1.00
TiCo@N_6G	1.93	1.83	4.52	5.58	1.00	0.24	1.38	0.76
TiNi@N_6G	1.95	1.85	4.50	5.68	1.19	0.04	1.31	0.71
TiZn@N_6G	2.00	2.00	4.41	3.92	1.03	0.18	1.40	0.72
TiRu@N_6G	2.01	1.92	4.60	5.78	0.92	0.02	1.37	0.75
TiRh@N_6G	1.93	1.99	4.48	5.50	1.06	0.20	1.36	0.57
TiHf@N_6G	2.03	2.10	3.40	6.96	0.88	0.23	1.26	1.46
TiOs@N_6G	2.01	1.92	4.58	5.74	0.86	0.04	1.38	0.82
VMn@N_6G	1.90	1.78	5.12	4.51	2.35	3.61	1.13	1.04
VFe@N_6G	1.90	1.86	5.10	4.99	2.34	0.43	1.14	0.85
VNi@N_6G	1.91	1.85	4.50	5.50	2.38	0.07	1.17	0.72
VW@N_6G	1.90	1.91	5.14	5.53	2.34	0.92	1.14	1.19
VOs@N_6G	1.90	1.91	5.14	5.38	2.28	0.00	1.14	0.81
CrMn@N_6G	1.82	1.78	5.10	3.53	2.00	0.54	1.16	0.99
CrFe@N_6G	1.82	1.85	5.11	4.23	1.86	2.93	1.15	0.96
CrRh@N_6G	1.86	1.98	4.47	4.26	2.78	0.08	1.19	0.60
CrOs@N_6G	1.93	1.91	4.54	4.59	3.11	0.06	1.13	0.85
MnFe@N_6G	2.02	1.87	5.08	4.37	4.28	2.97	0.84	0.97
MnCo@N_6G	1.95	1.85	4.51	4.39	4.06	0.65	1.11	0.77
MnCu@N_6G	2.00	1.92	4.84	3.67	4.24	0.00	0.95	0.67
MnRu@N_6G	2.02	1.92	5.03	4.58	4.24	0.00	0.85	0.78
MnOs@N_6G	1.80	1.92	5.08	4.21	3.55	0.01	1.04	0.83
FeNi@N_6G	1.87	1.87	4.70	4.14	3.14	0.72	0.87	0.73
FeCu@N_6G	1.78	1.92	4.86	3.93	2.96	0.00	0.99	0.67
FeRu@N_6G	1.86	1.92	5.07	4.96	2.87	0.00	0.97	0.79
CoNi@N_6G	1.85	1.82	4.51	4.82	1.05	1.10	0.80	0.75
CoCu@N_6G	1.87	1.89	4.57	4.00	1.45	0.04	0.81	0.63

**CoRh@N_6G**	1.83	1.99	4.63	4.70	1.92	0.00	0.80	0.62
**YCr@N_6G**	2.19	1.94	3.77	5.32	0.00	2.98	1.77	0.99
**YMn@N_6G**	2.20	1.81	4.40	4.90	0.00	1.91	1.79	1.00
**NbCr@N_6G**	2.11	1.92	4.43	5.78	0.76	3.26	1.48	1.11
**MoFe@N_6G**	2.05	1.87	4.45	4.85	1.76	3.04	1.11	1.02
**MoCo@N_6G**	1.94	1.83	5.09	4.73	1.28	0.82	1.09	0.77
**RuRh@N_6G**	1.92	2.02	4.48	4.98	0.00	0.41	0.81	0.54
**RuOs@N_6G**	1.98	1.92	4.50	5.29	0.00	0.00	0.70	0.85
**RhNi@N_6G**	1.99	1.85	4.51	4.71	0.51	0.00	0.67	0.73
**RhCu@N_6G**	2.04	1.92	4.87	3.87	0.94	0.00	0.55	0.68
**RhZn@N_6G**	2.03	1.87	4.51	3.08	0.43	0.00	0.56	1.07
**HfV@N_6G**	2.10	1.92	4.34	6.54	0.16	2.39	1.49	1.15
**HfCr@N_6G**	2.11	1.94	4.16	5.55	0.10	3.30	1.59	1.06
**HfCo@N_6G**	2.08	1.83	4.52	5.96	0.28	1.83	1.54	0.79
**HfNi@N_6G**	2.02	1.85	4.50	6.04	0.36	0.00	1.45	0.73
**HfCu@N_6G**	2.03	1.93	4.82	5.08	0.44	0.00	1.36	0.66
**WMn@N_6G**	1.92	2.03	5.05	4.67	1.15	4.28	1.18	0.87
**ReFe@N_6G**	1.92	1.88	4.49	4.62	0.74	1.80	1.02	0.94
**ReRu@N_6G**	1.92	1.98	4.50	4.95	0.54	0.00	1.02	0.70
**OsCo@N_6G**	1.92	1.84	4.53	4.98	0.00	0.23	0.91	0.54
**OsRh@N_6G**	1.92	2.02	4.47	4.93	0.00	0.40	0.87	0.55

Table S4. Calculated adsorption energies (E_{ad}, eV) of five possible configurations of N_2 on 72 stable systems.

system	E_{ad}(End-on-a)	E_{ad}(End-on-b)	E_{ad}(Side-on-a)	E_{ad}(Side-on-b)	E_{ad}(Side-on-c)
$Sc_2@N_6G$	-0.76	-	-	-	-1.92
$Ti_2@N_6G$	-1.20	-	-1.21	-	-2.65
$V_2@N_6G$	-	-	-1.13	-	-1.98
$Cr_2@N_6G$	-1.31	-	-1.04	-	-2.19
$Mn_2@N_6G$	-	-	-1.08	-	-2.83
$Co_2@N_6G$	-1.69	-	-1.12	-	-1.95
$Ni_2@N_6G$	-1.31	-	-1.09	-	-1.91
$Cu_2@N_6G$	-1.59	-	-1.09	-	-2.09
$Y_2@N_6G$	-	-	-0.38	-	-1.69
$Rh_2@N_6G$	-1.58	-	-1.02	-	-2.38
$Pd_2@N_6G$	-1.28	-	-0.95	-	-1.99
$Ta_2@N_6G$	-1.37	-	-1.72	-	-3.64
$Os_2@N_6G$	-2.03	-	-1.22	-	-2.79
$ScTi@N_6G$	-1.05	-1.18	-	-	-2.46
$ScV@N_6G$	-0.85	-1.15	-0.74	-1.14	-2.10
$ScMn@N_6G$	-0.85	-1.82	-1.01	-1.53	-2.74
$ScFe@N_6G$	-	-1.48	-0.70	-0.85	-2.39
$ScNi@N_6G$	-0.80	-1.26	-0.42	-1.11	-2.36
$ScCu@N_6G$	-0.82	-1.48	-0.70	-0.99	-1.79
$ScY@N_6G$	-0.71	-1.86	-	-0.42	-1.86
$ScZr@N_6G$	-0.72	-0.86	-	-1.93	-2.15
$ScRu@N_6G$	-0.87	-1.67	-0.76	-	-2.54
$ScOs@N_6G$	-0.83	-2.09	-	-1.45	-2.94
$TiV@N_6G$	-1.24	-1.21	-1.37	-1.27	-2.66
$TiFe@N_6G$	-1.36	-1.39	-1.34	-1.12	-2.34
$TiCo@N_6G$	-1.16	-1.51	-1.13	-1.00	-2.21
$TiNi@N_6G$	-1.26	-	-1.29	-0.83	-2.27
$TiZn@N_6G$	-1.22	-0.45	-1.18	-0.45	-1.78
$TiRu@N_6G$	-	-1.63	-1.12	-2.16	-2.43
$TiRh@N_6G$	-1.16	-1.50	-1.14	-1.06	-2.53
$TiHf@N_6G$	-1.19	-1.13	-	-2.41	-2.62
$TiOs@N_6G$	-1.27	-2.01	-	-	-2.90
$VMn@N_6G$	-1.26	-1.81	-1.11	-1.49	-2.67
$VFe@N_6G$	-1.29	-1.40	-1.24	-1.38	-2.28
$VNi@N_6G$	-1.19	-1.42	-1.08	-1.01	-1.96
$VW@N_6G$	-1.26	-1.72	-1.13	-2.36	-3.06
$VOs@N_6G$	-1.31	-2.28	-1.15	-1.76	-2.86
$CrMn@N_6G$	-1.58	-1.57	-1.31	-1.47	-3.00
$CrFe@N_6G$	-1.32	-1.51	-1.31	-1.33	-2.55
$CrRh@N_6G$	-1.24	-1.43	-1.07	-0.98	-2.44
$CrOs@N_6G$	-1.25	-1.98	-0.95	-1.38	-2.71

**MnFe@N_6G**	-0.78	-1.46	-0.79	-1.30	-2.39
**MnCo@N_6G**	-1.17	-1.45	-1.02	-1.45	-2.13
**MnCu@N_6G**	-1.19	-1.48	-0.92	-1.09	-1.92
**MnRu@N_6G**	-1.20	-1.83	-1.09	-1.23	-2.45
**MnOs@N_6G**	-1.93	-2.27	-1.82	-1.78	-3.65
**FeNi@N_6G**	-1.40	-	-1.14	-1.07	-2.10
**FeCu@N_6G**	-1.70	-1.49	-1.07	-1.08	-2.25
**FeRu@N_6G**	-1.51	-1.81	-1.32	-1.14	-2.34
**CoNi@N_6G**	-1.40	-1.48	-0.99	-1.22	-1.94
**CoCu@N_6G**	-1.48	-1.63	-0.76	-1.04	-2.05
**CoRh@N_6G**	-1.43	-1.46	-0.94	-1.04	-2.05
**YCr@N_6G**	-0.58	-	-0.42	-	-1.93
**YMn@N_6G**	-0.67	-1.86	-1.28	-	-2.81
**NbCr@N_6G**	-1.21	-1.17	-1.23	-	-2.73
**MoFe@N_6G**	-1.45	-1.34	-1.53	-1.16	-2.37
**MoCo@N_6G**	-1.55	-1.51	-1.72	-0.81	-2.32
**RuRh@N_6G**	-1.63	-1.54	-0.96	-1.10	-2.21
**RuOs@N_6G**	-1.59	-2.01	-1.15	-1.34	-2.58
**RhNi@N_6G**	-1.35	-1.33	-0.77	-1.07	-2.15
**RhCu@N_6G**	-1.59	-1.49	-1.02	-1.09	-2.20
**RhZn@N_6G**	-1.50	-0.51	-1.07	-0.50	-1.81
**HfV@N_6G**	-1.24	-1.17	-1.29	-	-2.44
**HfCr@N_6G**	-1.26	-1.28	-1.15	-	-2.57
**HfCo@N_6G**	-1.33	-1.42	-1.31	-	-2.54
**HfNi@N_6G**	-1.25	-1.34	-1.42	-0.78	-2.55
**HfCu@N_6G**	-1.14	-1.45	-1.36	-1.07	-2.29
**WMn@N_6G**	-2.11	-1.12	-3.13	-1.73	-1.18
**ReFe@N_6G**	-	-1.51	-1.81	-1.16	-2.83
**ReRu@N_6G**	-1.99	-1.60	-1.82	-1.14	-2.87
**OsCo@N_6G**	-1.99	-1.50	-1.45	-1.50	-2.47
**OsRh@N_6G**	-2.03	-1.53	-1.27	-1.08	-2.61

Table S5. Computed adsorption free energies, integrated-crystal orbital Hamilton population (ICOHP) values for N-N bonds, Bader charges and N-N bond lengths of the adsorbed side-on-c N_2 on 72 stable systems.

system	ΔG ($*N_2$)	d_{N-N}	Bader ($*N_2$)	ICOHP (N-N)
Sc_2@N_6G	-1.39	1.20	0.87	-18.88
Ti_2@N_6G	-2.05	1.23	1.09	-16.65
V_2@N_6G	-1.43	1.19	0.83	-18.68
Cr_2@N_6G	-1.63	1.20	0.86	-18.31
Mn_2@N_6G	-2.26	1.20	0.88	-18.31
Co_2@N_6G	-1.36	1.17	0.61	-19.87
Ni_2@N_6G	-1.33	1.17	0.60	-19.68
Cu_2@N_6G	-1.50	1.16	0.45	-20.30
Y_2@N_6G	-1.18	1.20	1.34	-15.34
Rh_2@N_6G	-1.79	1.18	0.58	-19.54
Pd_2@N_6G	-1.41	1.16	0.42	-20.54
Ta_2@N_6G	-3.03	1.25	1.21	-15.61
Os_2@N_6G	-2.18	1.18	0.74	-18.72
ScTi@N_6G	-1.92	1.23	1.02	-17.18
ScV@N_6G	-1.55	1.20	0.88	-18.53
ScMn@N_6G	-2.20	1.21	0.94	-17.96
ScFe@N_6G	-1.81	1.19	0.82	-18.80
ScNi@N_6G	-1.78	1.18	0.73	-19.13
ScCu@N_6G	-1.25	1.17	0.59	-19.87
ScY@N_6G	-1.34	1.20	0.94	-18.65
ScZr@N_6G	-1.62	1.24	1.13	-17.15
ScRu@N_6G	-1.98	1.19	0.71	-19.50
ScOs@N_6G	-2.35	1.19	0.80	-18.74
TiV@N_6G	-2.06	1.22	1.01	-17.23
TiFe@N_6G	-1.76	1.19	0.82	-18.50
TiCo@N_6G	-1.62	1.19	0.75	-18.71
TiNi@N_6G	-1.70	1.18	0.74	-19.04
TiZn@N_6G	-1.24	1.19	0.76	-18.74
TiRu@N_6G	-1.85	1.19	0.72	-19.15
TiRh@N_6G	-1.95	1.20	0.76	-18.26
TiHf@N_6G	-2.07	1.25	1.17	-16.13
TiOs@N_6G	-2.30	1.20	0.79	-18.54
VMn@N_6G	-2.09	1.21	0.98	-17.45
VFe@N_6G	-1.70	1.19	0.82	-18.77
VNi@N_6G	-1.41	1.18	0.70	-19.27
VW@N_6G	-2.46	1.23	1.03	-16.73
VOs@N_6G	-2.30	1.19	0.78	-18.90
CrMn@N_6G	-2.43	1.21	0.96	-17.52
CrFe@N_6G	-1.96	1.19	0.83	-18.55
CrRh@N_6G	-1.86	1.20	0.74	-18.31

**CrOs@N_6G**	-2.11	1.19	0.79	-18.55
**MnFe@N_6G**	-1.82	1.19	0.81	-18.72
**MnCo@N_6G**	-1.61	1.18	0.68	-19.36
**MnCu@N_6G**	-1.35	1.17	0.59	-19.58
**MnRu@N_6G**	-1.87	1.18	0.67	-19.66
**MnOs@N_6G**	-3.04	1.19	0.76	-18.97
**FeNi@N_6G**	-1.51	1.18	0.69	-19.17
**FeCu@N_6G**	-1.67	1.17	0.60	-19.45
**FeRu@N_6G**	-1.76	1.18	0.67	-19.43
**CoNi@N_6G**	-1.36	1.17	0.61	-19.66
**CoCu@N_6G**	-1.45	1.16	0.54	-19.68
**CoRh@N_6G**	-1.46	1.17	0.58	-19.73
**YCr@N_6G**	-1.42	1.20	0.95	-18.54
**YMn@N_6G**	-2.24	1.21	1.05	-17.42
**NbCr@N_6G**	-2.13	1.22	1.03	-17.40
**MoFe@N_6G**	-1.78	1.20	0.88	-18.11
**MoCo@N_6G**	-1.73	1.19	0.81	-18.46
**RuRh@N_6G**	-1.62	1.17	0.58	-19.71
**RuOs@N_6G**	-1.98	1.20	0.69	-18.56
**RhNi@N_6G**	-1.56	1.17	0.56	-19.78
**RhCu@N_6G**	-1.60	1.16	0.48	-20.07
**RhZn@N_6G**	-1.24	1.17	0.76	-19.48
**HfV@N_6G**	-1.86	1.23	1.06	-16.93
**HfCr@N_6G**	-1.99	1.22	0.98	-17.36
**HfCo@N_6G**	-1.96	1.21	0.85	-18.01
**HfNi@N_6G**	-1.97	1.19	0.77	-18.79
**HfCu@N_6G**	-1.73	1.18	0.68	-19.12
**WMn@N_6G**	-2.53	1.21	0.98	-17.65
**ReFe@N_6G**	-2.23	1.20	0.85	-18.21
**ReRu@N_6G**	-2.27	1.21	0.81	-17.78
**OsCo@N_6G**	-1.87	1.18	0.69	-19.01
**OsRh@N_6G**	-2.01	1.18	0.66	-18.93

Table S6. Computed total energy (E_{tot}), zero-potential correction energy (E_{zpe}) and entropy contribution (TS, T=298.15 K) of the optimized intermediates for urea production on the Sc_2@N_6G system.

Sc_2@N_6G	E_{tot} (eV)	E_{ZPE} (eV)	TS (eV)
*N_2	-672.82	0.19	0.11
*NCON	-688.35	0.42	0.20
*NCONH	-694.28	0.77	0.19
*NHCONH	-698.61	1.10	0.21
*$NCONH_2$	-698.26	1.11	0.19
*$NHCONH_2$	-701.72	1.46	0.18
*NH_2CONH_2	-703.44	1.76	0.20

Table S7. Computed total energy (E_{tot}), zero-potential correction energy (E_{zpe}) and entropy contribution (TS, T=298.15 K) of the optimized intermediates for urea production on the Ti_2@N_6G system.

Ti_2@N_6G	E_{tot} (eV)	E_{ZPE} (eV)	TS (eV)
*N_2	-673.83	0.22	0.07
*NCON	-690.31	0.50	0.13
*NCONH	-695.23	0.78	0.18
*NHCONH	-699.01	1.10	0.19
*$NCONH_2$	-698.81	1.14	0.17
*$NHCONH_2$	-702.19	1.47	0.18
*NH_2CONH_2	-704.18	1.75	0.19

Table S8. Computed total energy (E_{tot}), zero-potential correction energy (E_{zpe}) and entropy contribution (TS, T=298.15 K) of the optimized intermediates for urea production on the V_2@N_6G system.

V_2@N_6G	E_{tot} (eV)	E_{ZPE} (eV)	TS (eV)
*N_2	-674.29	0.20	0.10
*NCON	-691.12	0.46	0.18
*NCONH	-695.60	0.78	0.18
*NHCONH	-699.67	1.12	0.16
*$NCONH_2$	-698.84	1.10	0.20
*$NHCONH_2$	-703.03	1.49	1.17
*NH_2CONH_2	-705.39	1.79	0.19

Table S9. Computed total energy (E_{tot}), zero-potential correction energy (E_{zpe}) and entropy contribution (TS, T=298.15 K) of the optimized intermediates for urea production on the Cr_2@N_6G system.

Cr_2@N_6G	E_{tot} (eV)	E_{ZPE} (eV)	TS (eV)
*N_2	-674.59	0.20	0.10
*NCON	-691.04	0.46	0.15
*NCONH	-695.35	0.77	0.19
*NHCONH	-699.48	1.10	0.20
*$NCONH_2$	-699.13	1.12	0.19
*$NHCONH_2$	-702.85	1.45	0.22
*NH_2CONH_2	-705.21	1.81	0.19

Table S10. Computed total energy (E_{tot}), zero-potential correction energy (E_{zpe}) and entropy contribution (TS, T=298.15 K) of the optimized intermediates for urea production on the Mn_2@N_6G system.

Mn_2@N_6G	E_{tot} (eV)	E_{ZPE} (eV)	TS (eV)
*N_2	-674.09	0.21	0.09
*NCON	-689.53	0.45	0.15
*NCONH	-694.13	0.80	0.17
*NHCONH	-698.37	1.11	0.20
*$NCONH_2$	-698.10	1.13	0.18
*$NHCONH_2$	-702.54	1.49	0.17
*NH_2CONH_2	-704.97	1.78	0.22

Table S11. Computed total energy (E_{tot}), zero-potential correction energy (E_{zpe}) and entropy contribution (TS, T=298.15 K) of the optimized intermediates for urea production on the Co_2@N_6G system.

Co_2@N_6G	E_{tot} (eV)	E_{ZPE} (eV)	TS (eV)
*N_2	-669.07	0.22	0.08
*NCON	-683.86	0.49	0.12
*NCONH	-688.38	0.78	0.15
*NHCONH	-692.75	1.10	0.17
*$NCONH_2$	-692.65	1.13	0.16
*$NHCONH_2$	-697.01	1.47	0.18
*NH_2CONH_2	-703.44	1.76	0.20

Table S12. Computed total energy (E_{tot}), zero-potential correction energy (E_{zpe}) and entropy contribution (TS, T=298.15 K) of the optimized intermediates for urea production on the $Ni_2@N_6G$ system.

$Ni_2@N_6G$	E_{tot} (eV)	E_{ZPE} (eV)	TS (eV)
*N_2	-666.12	0.22	0.09
*NCON	-680.13	0.47	0.13
*NCONH	-685.00	0.77	0.19
*NHCONH	-698.50	1.09	0.19
*$NCONH_2$	-689.32	1.13	0.16
*$NHCONH_2$	-694.04	1.47	0.18
*NH_2CONH_2	-697.02	1.80	0.22

Table S13. Computed total energy (E_{tot}), zero-potential correction energy (E_{zpe}) and entropy contribution (TS, T=298.15 K) of the optimized intermediates for urea production on the $Cu_2@N_6G$ system.

$Cu_2@N_6G$	E_{tot} (eV)	E_{ZPE} (eV)	TS (eV)
*N_2	-662.77	0.23	0.09
*NCON	-676.29	0.47	0.15
*NCONH	-680.49	0.77	0.17
*NHCONH	-685.49	1.09	0.20
*$NCONH_2$	-684.89	1.12	0.18
*$NHCONH_2$	-690.29	1.47	0.19
*NH_2CONH_2	-693.94	1.80	0.21

Table S14. Computed total energy (E_{tot}), zero-potential correction energy (E_{zpe}) and entropy contribution (TS, T=298.15 K) of the optimized intermediates for urea production on the $Y_2@N_6G$ system.

$Y_2@N_6G$	E_{tot} (eV)	E_{ZPE} (eV)	TS (eV)
*N_2	-672.85	0.19	0.12
*NCON	-688.25	0.44	0.18
*NCONH	-695.60	0.78	0.12
*NHCONH	-698.82	1.10	0.19
*$NCONH_2$	-699.07	1.13	0.17
*$NHCONH_2$	-701.69	1.44	0.20
*NH_2CONH_2	-703.11	1.74	0.22

Table S15. Computed total energy (E_{tot}), zero-potential correction energy (E_{zpe}) and entropy contribution (TS, T=298.15 K) of the optimized intermediates for urea production on the $Rh_2@N_6G$ system.

$Rh_2@N_6G$	E_{tot} (eV)	E_{ZPE} (eV)	TS (eV)
$*N_2$	-667.67	0.22	0.09
*NCON	-682.68	0.48	0.12
*NCONH	-687.03	0.82	0.12
*NHCONH	-691.40	1.11	0.16
$*NCONH_2$	-691.26	1.15	0.16
$*NHCONH_2$	-695.43	1.50	0.15
$*NH_2CONH_2$	-698.41	1.78	0.20

Table S16. Computed total energy (E_{tot}), zero-potential correction energy (E_{zpe}) and entropy contribution (TS, T=298.15 K) of the optimized intermediates for urea production on the $Pd_2@N_6G$ system.

$Pd_2@N_6G$	E_{tot} (eV)	E_{ZPE} (eV)	TS (eV)
$*N_2$	-663.97	0.23	0.09
*NCON	-677.54	0.45	0.16
*NCONH	-682.23	0.79	0.14
*NHCONH	-686.86	1.11	0.18
$*NCONH_2$	-686.72	1.11	0.20
$*NHCONH_2$	-691.38	1.47	0.18
$*NH_2CONH_2$	-694.99	1.79	0.20

Table S17. Computed total energy (E_{tot}), zero-potential correction energy (E_{zpe}) and entropy contribution (TS, T=298.15 K) of the optimized intermediates for urea production on the $Ta_2@N_6G$ system.

$Ta_2@N_6G$	E_{tot} (eV)	E_{ZPE} (eV)	TS (eV)
$*N_2$	-678.04	0.23	0.06
*NCON	-695.01	0.50	0.13
*NCONH	-699.74	0.78	0.18
*NHCONH	-703.23	1.13	0.17
$*NCONH_2$	-702.78	1.13	0.19
$*NHCONH_2$	-705.90	1.50	0.15
$*NH_2CONH_2$	-709.80	1.74	0.17

Table S18. Computed total energy (E_{tot}), zero-potential correction energy (E_{zpe}) and entropy contribution (TS, T=298.15 K) of the optimized intermediates for urea production on the Os_2@N_6G system.

Os_2@N_6G	E_{tot} (eV)	E_{ZPE} (eV)	TS (eV)
*N_2	-672.64	0.23	0.07
*NCON	-689.61	0.49	0.13
*NCONH	-693.82	0.81	0.14
*NHCONH	-697.45	1.11	0.17
*$NCONH_2$	-697.13	1.15	0.16
*$NHCONH_2$	-700.74	1.49	0.16
*NH_2CONH_2	-702.99	1.81	0.19

Table S19. Computed total energy (E_{tot}), zero-potential correction energy (E_{zpe}) and entropy contribution (TS, T=298.15 K) of the optimized intermediates for urea production on the ScTi@N_6G system.

ScTi@N_6G	E_{tot} (eV)	E_{ZPE} (eV)	TS (eV)
*N_2	-673.35	0.19	0.10
*NCON	-689.24	0.48	0.15
*NCONH	-694.80	0.77	0.19
*NHCONH	-698.68	1.11	0.19
*$NCONH_2$	-698.60	1.12	0.18
*$NHCONH_2$	-701.90	1.46	0.20
*NH_2CONH_2	-705.68	1.73	0.24

Table S20. Computed total energy (E_{tot}), zero-potential correction energy (E_{zpe}) and entropy contribution (TS, T=298.15 K) of the optimized intermediates for urea production on the ScV@N_6G system.

ScV@N_6G	E_{tot} (eV)	E_{ZPE} (eV)	TS (eV)
*N_2	-673.68	0.20	0.10
*NCON	-689.95	0.45	0.19
*NCONH	-694.98	0.79	0.17
*NHCONH	-698.98	1.10	0.19
*$NCONH_2$	-698.74	1.11	0.19
*$NHCONH_2$	-702.42	1.49	0.17
*NH_2CONH_2	-705.87	1.72	0.20

Table S21. Computed total energy (E_{tot}), zero-potential correction energy (E_{zpe}) and entropy contribution (TS, T=298.15 K) of the optimized intermediates for urea production on the ScMn@N_6G system.

ScMn@N_6G	E_{tot} (eV)	E_{ZPE} (eV)	TS (eV)
*N_2	-673.44	0.20	0.10
*NCON	-689.12	0.47	0.16
*NCONH	-693.86	0.80	0.17
*NHCONH	-697.88	1.11	0.20
*$NCONH_2$	-697.32	1.09	0.21
*$NHCONH_2$	-702.09	1.46	0.21
*NH_2CONH_2	-704.31	1.81	0.20

Table S22. Computed total energy (E_{tot}), zero-potential correction energy (E_{zpe}) and entropy contribution (TS, T=298.15 K) of the optimized intermediates for urea production on the ScFe@N_6G system.

ScFe@N_6G	E_{tot} (eV)	E_{ZPE} (eV)	TS (eV)
*N_2	-672.48	0.22	0.09
*NCON	-687.91	0.48	0.14
*NCONH	-692.54	0.78	0.18
*NHCONH	-696.80	1.11	0.18
*$NCONH_2$			
*$NHCONH_2$	-700.72	1.48	0.18
*NH_2CONH_2	-703.35	1.76	0.17

Table S23. Computed total energy (E_{tot}), zero-potential correction energy (E_{zpe}) and entropy contribution (TS, T=298.15 K) of the optimized intermediates for urea production on the ScNi@N_6G system.

ScNi@N_6G	E_{tot} (eV)	E_{ZPE} (eV)	TS (eV)
*N_2	-669.87	0.22	0.09
*NCON	-684.52	0.49	0.14
*NCONH	-689.16	0.79	0.16
*NHCONH	-693.99	1.13	0.17
*$NCONH_2$	-	-	-
*$NHCONH_2$	-697.56	1.47	0.19
*NH_2CONH_2	-700.39	1.81	0.17

Table S24. Computed total energy (E_{tot}), zero-potential correction energy (E_{zpe}) and entropy contribution (TS, T=298.15 K) of the optimized intermediates for urea production on the ScCu@N_6G system.

ScCu@N_6G	E_{tot} (eV)	E_{ZPE} (eV)	TS (eV)
*N_2	-667.53	0.21	0.11
*NCON	-682.12	0.30	0.12
*NCONH	-687.26	0.78	0.18
*NHCONH	-692.11	1.12	0.18
*$NCONH_2$	-691.93	1.14	0.17
*$NHCONH_2$	-696.35	1.46	0.20
*NH_2CONH_2	-698.82	1.80	0.18

Table S25. Computed total energy (E_{tot}), zero-potential correction energy (E_{zpe}) and entropy contribution (TS, T=298.15 K) of the optimized intermediates for urea production on the ScY@N_6G system.

ScY@N_6G	E_{tot} (eV)	E_{ZPE} (eV)	TS (eV)
*N_2	-672.91	0.19	0.12
*NCON	-688.55	0.43	0.17
*NCONH	-694.40	0.78	0.18
*NHCONH	-698.73	1.09	0.20
*$NCONH_2$	-698.32	1.09	0.21
*$NHCONH_2$	-701.99	1.47	0.19
*NH_2CONH_2	-703.31	1.76	0.21

Table S26. Computed total energy (E_{tot}), zero-potential correction energy (E_{zpe}) and entropy contribution (TS, T=298.15 K) of the optimized intermediates for urea production on the ScZr@N_6G system.

ScZr@N_6G	E_{tot} (eV)	E_{ZPE} (eV)	TS (eV)
*N_2	-675.83	0.19	0.11
*NCON	-692.31	0.44	0.17
*NCONH	-697.56	0.77	0.20
*NHCONH	-701.48	1.11	0.19
*$NCONH_2$	-701.13	1.11	0.20
*$NHCONH_2$	-704.30	1.45	0.23
*NH_2CONH_2	-706.03	1.68	0.22

Table S27. Computed total energy (E_{tot}), zero-potential correction energy (E_{zpe}) and entropy contribution (TS, T=298.15 K) of the optimized intermediates for urea production on the ScRu@N_6G system.

ScRu@N_6G	E_{tot} (eV)	E_{ZPE} (eV)	TS (eV)
*N_2	-672.25	0.22	0.11
*NCON	-687.74	0.46	0.16
*NCONH	-692.46	0.78	0.19
*NHCONH	-696.74	1.11	0.18
*$NCONH_2$	-695.72	1.09	0.21
*$NHCONH_2$	-700.37	1.47	0.18
*NH_2CONH_2	-702.63	1.80	0.20

Table S28. Computed total energy (E_{tot}), zero-potential correction energy (E_{zpe}) and entropy contribution (TS, T=298.15 K) of the optimized intermediates for urea production on the ScOs@N_6G system.

ScOs@N_6G	E_{tot} (eV)	E_{ZPE} (eV)	TS (eV)
*N_2	-673.10	0.23	0.09
*NCON	-688.93	0.47	0.15
*NCONH	-693.76	0.77	0.21
*NHCONH	-697.57	1.11	0.18
*$NCONH_2$	-697.40	1.14	0.16
*$NHCONH_2$	-701.19	1.48	0.18
*NH_2CONH_2	-703.23	1.70	0.23

Table S29. Computed total energy (E_{tot}), zero-potential correction energy (E_{zpe}) and entropy contribution (TS, T=298.15 K) of the optimized intermediates for urea production on the TiV@N_6G system.

TiV@N_6G	E_{tot} (eV)	E_{ZPE} (eV)	TS (eV)
*N_2	-674.31	0.22	0.07
*NCON	-690.76	0.48	0.17
*NCONH	-695.32	0.79	0.16
*NHCONH	-699.29	1.11	0.19
*$NCONH_2$	-698.78	1.12	0.19
*$NHCONH_2$	-702.76	1.45	0.21
*NH_2CONH_2	-704.69	1.78	0.22

Table S30. Computed total energy (E_{tot}), zero-potential correction energy (E_{zpe}) and entropy contribution (TS, T=298.15 K) of the optimized intermediates for urea production on the TiFe@N_6G system.

TiFe@N_6G	E_{tot} (eV)	E_{ZPE} (eV)	TS (eV)
*N_2	-672.69	0.22	0.09
*NCON	-688.60	0.48	0.16
*NCONH	-693.04	0.82	0.14
*NHCONH	-697.17	1.11	0.17
*$NCONH_2$	-697.16	1.15	0.17
*$NHCONH_2$	-700.97	1.47	0.18
*NH_2CONH_2	-703.40	1.77	0.23

Table S31. Computed total energy (E_{tot}), zero-potential correction energy (E_{zpe}) and entropy contribution (TS, T=298.15 K) of the optimized intermediates for urea production on the TiCo@N_6G system.

TiCo@N_6G	E_{tot} (eV)	E_{ZPE} (eV)	TS (eV)
*N_2	-671.51	0.22	0.08
*NCON	-687.20	0.45	0.21
*NCONH	-691.60	0.79	0.15
*NHCONH	-695.84	1.10	0.18
*$NCONH_2$	-695.62	1.10	0.23
*$NHCONH_2$	-699.75	1.49	0.17
*NH_2CONH_2	-702.20	1.77	0.23

Table S32. Computed total energy (E_{tot}), zero-potential correction energy (E_{zpe}) and entropy contribution (TS, T=298.15 K) of the optimized intermediates for urea production on the TiNi@N_6G system.

TiNi@N_6G	E_{tot} (eV)	E_{ZPE} (eV)	TS (eV)
*N_2	-670.15	0.22	0.09
*NCON	-685.34	0.46	0.15
*NCONH	-690.16	0.80	0.15
*NHCONH	-694.38	1.10	0.18
*$NCONH_2$	-694.32	1.13	0.18
*$NHCONH_2$	-698.18	1.50	0.16
*NH_2CONH_2	-700.70	1.77	0.23

Table S33. Computed total energy (E_{tot}), zero-potential correction energy (E_{zpe}) and entropy contribution (TS, T=298.15 K) of the optimized intermediates for urea production on the TiZn@N_6G system.

TiZn@N_6G	E_{tot} (eV)	E_{ZPE} (eV)	TS (eV)
*N_2	-665.82	0.20	0.10
*NCON	-681.30	0.47	0.14
*NCONH	-687.00	0.81	0.16
*NHCONH	-691.19	1.11	0.18
*$NCONH_2$	-690.93	1.11	0.19
*$NHCONH_2$	-694.75	1.48	0.17
*NH_2CONH_2	-696.97	1.78	0.20

Table S34. Computed total energy (E_{tot}), zero-potential correction energy (E_{zpe}) and entropy contribution (TS, T=298.15 K) of the optimized intermediates for urea production on the TiRu@N_6G system.

TiRu@N_6G	E_{tot} (eV)	E_{ZPE} (eV)	TS (eV)
*N_2	-672.44	0.22	0.09
*NCON	-688.39	0.49	0.13
*NCONH	-692.91	0.79	0.16
*NHCONH	-697.03	1.12	0.17
*$NCONH_2$	-696.76	1.15	0.16
*$NHCONH_2$	-700.91	1.47	0.18
*NH_2CONH_2	-702.81	1.77	0.22

Table S35. Computed total energy (E_{tot}), zero-potential correction energy (E_{zpe}) and entropy contribution (TS, T=298.15 K) of the optimized intermediates for urea production on the TiRh@N_6G system.

TiRh@N_6G	E_{tot} (eV)	E_{ZPE} (eV)	TS (eV)
*N_2	-671.00	0.22	0.08
*NCON	-686.62	0.48	0.14
*NCONH	-690.98	0.81	0.14
*NHCONH	-695.18	1.09	0.22
*$NCONH_2$	-695.05	1.14	0.17
*$NHCONH_2$	-699.06	1.45	0.23
*NH_2CONH_2	-701.33	1.76	0.20

Table S36. Computed total energy (E_{tot}), zero-potential correction energy (E_{zpe}) and entropy contribution (TS, T=298.15 K) of the optimized intermediates for urea production on the TiHf@N_6G system.

TiHf@N_6G	E_{tot} (eV)	E_{ZPE} (eV)	TS (eV)
*N_2	-675.93	0.20	0.09
*NCON	-692.46	0.46	0.16
*NCONH	-697.78	0.81	0.15
*NHCONH	-701.47	1.13	0.15
*$NCONH_2$	-701.19	1.13	0.16
*$NHCONH_2$	-704.40	1.45	0.19
*NH_2CONH_2	-705.86	1.67	0.23

Table S37. Computed total energy (E_{tot}), zero-potential correction energy (E_{zpe}) and entropy contribution (TS, T=298.15 K) of the optimized intermediates for urea production on the TiOs@N_6G system.

TiOs@N_6G	E_{tot} (eV)	E_{ZPE} (eV)	TS (eV)
*N_2	-673.38	0.23	0.08
*NCON	-689.71	0.48	0.17
*NCONH	-694.00	0.80	0.16
*NHCONH	-698.10	1.11	0.17
*$NCONH_2$	-697.35	1.14	0.17
*$NHCONH_2$	-701.53	1.48	0.17
*NH_2CONH_2	-703.72	1.80	0.20

Table S38. Computed total energy (E_{tot}), zero-potential correction energy (E_{zpe}) and entropy contribution (TS, T=298.15 K) of the optimized intermediates for urea production on the VMn@N_6G system.

VMn@N_6G	E_{tot} (eV)	E_{ZPE} (eV)	TS (eV)
*N_2	-674.20	0.21	0.08
*NCON	-690.34	0.44	0.19
*NCONH	-694.97	0.82	0.14
*NHCONH	-699.13	1.12	0.19
*$NCONH_2$	-698.88	1.12	0.20
*$NHCONH_2$	-702.86	1.47	0.18
*NH_2CONH_2	-704.98	1.79	0.20

Table S39. Computed total energy (E_{tot}), zero-potential correction energy (E_{zpe}) and entropy contribution (TS, T=298.15 K) of the optimized intermediates for urea production on the VFe@N_6G system.

VFe@N_6G	E_{tot} (eV)	E_{ZPE} (eV)	TS (eV)
*N_2	-673.14	0.22	0.09
*NCON	-689.00	0.46	0.15
*NCONH	-693.40	0.79	0.16
*NHCONH	-697.57	1.08	0.23
*$NCONH_2$	-697.07	1.14	0.16
*$NHCONH_2$	-701.45	1.46	0.19
*NH_2CONH_2	-703.79	1.79	0.22

Table S40. Computed total energy (E_{tot}), zero-potential correction energy (E_{zpe}) and entropy contribution (TS, T=298.15 K) of the optimized intermediates for urea production on the VNi@N_6G system.

VNi@N_6G	E_{tot} (eV)	E_{ZPE} (eV)	TS (eV)
*N_2	-670.55	0.21	0.12
*NCON	-685.73	0.48	0.13
*NCONH	-690.56	0.80	0.15
*NHCONH	-694.68	1.11	0.19
*$NCONH_2$	-694.68	1.13	0.18
*$NHCONH_2$	-698.68	1.48	0.17
*NH_2CONH_2	-701.03	1.80	0.19

Table S41. Computed total energy (E_{tot}), zero-potential correction energy (E_{zpe}) and entropy contribution (TS, T=298.15 K) of the optimized intermediates for urea production on the VW@N_6G system.

VW@N_6G	E_{tot} (eV)	E_{ZPE} (eV)	TS (eV)
*N_2	-675.93	0.22	0.07
*NCON	-692.69	0.47	0.17
*NCONH	-697.19	0.80	0.16
*NHCONH	-701.38	1.11	0.17
*$NCONH_2$	-700.39	1.14	0.16
*$NHCONH_2$	-704.47	1.46	0.20
*NH_2CONH_2	-705.59	1.75	0.21

Table S42. Computed total energy (E_{tot}), zero-potential correction energy (E_{zpe}) and entropy contribution (TS, T=298.15 K) of the optimized intermediates for urea production on the VOs@N_6G system.

VOs@N_6G	E_{tot} (eV)	E_{ZPE} (eV)	TS (eV)
*N_2	-673.70	0.22	0.11
*NCON	-690.14	0.48	0.14
*NCONH	-694.42	0.80	0.15
*NHCONH	-698.47	1.11	0.18
*$NCONH_2$	-697.68	1.12	0.18
*$NHCONH_2$	-701.64	1.46	0.18
*NH_2CONH_2	-704.54	1.75	0.17

Table S43. Computed total energy (E_{tot}), zero-potential correction energy (E_{zpe}) and entropy contribution (TS, T=298.15 K) of the optimized intermediates for urea production on the CrMn@N_6G system.

CrMn@N_6G	E_{tot} (eV)	E_{ZPE} (eV)	TS (eV)
*N_2	-674.36	0.21	0.08
*NCON	-690.26	0.48	0.14
*NCONH	-694.84	0.80	0.18
*NHCONH	-699.07	1.10	0.20
*$NCONH_2$	-698.02	1.10	0.21
*$NHCONH_2$	-702.77	1.46	0.19
*NH_2CONH_2	-705.13	1.79	0.20

Table S44. Computed total energy (E_{tot}), zero-potential correction energy (E_{zpe}) and entropy contribution (TS, T=298.15 K) of the optimized intermediates for urea production on the CrFe@N_6G system.

CrFe@N_6G	E_{tot} (eV)	E_{ZPE} (eV)	TS (eV)
*N_2	-673.28	0.22	0.08
*NCON	-689.02	0.47	0.14
*NCONH	-693.30	0.77	0.18
*NHCONH	-697.07	1.12	0.17
*$NCONH_2$	-697.47	1.13	0.17
*$NHCONH_2$	-701.37	1.47	0.18
*NH_2CONH_2	-703.86	1.77	0.24

Table S45. Computed total energy (E_{tot}), zero-potential correction energy (E_{zpe}) and entropy contribution (TS, T=298.15 K) of the optimized intermediates for urea production on the CrRh@N_6G system.

CrRh@N_6G	E_{tot} (eV)	E_{ZPE} (eV)	TS (eV)
*N_2	-671.43	0.22	0.08
*NCON	-686.95	0.44	0.19
*NCONH	-691.31	0.76	0.21
*NHCONH	-695.59	1.12	0.17
*$NCONH_2$	-695.49	1.11	0.22
*$NHCONH_2$	-699.42	1.48	0.17
*NH_2CONH_2	-701.78	1.77	0.21

Table S46. Computed total energy (E_{tot}), zero-potential correction energy (E_{zpe}) and entropy contribution (TS, T=298.15 K) of the optimized intermediates for urea production on the CrOs@N_6G system.

CrOs@N_6G	E_{tot} (eV)	E_{ZPE} (eV)	TS (eV)
*N_2	-673.89	0.23	0.08
*NCON	-690.13	0.46	0.15
*NCONH	-694.32	0.80	0.16
*NHCONH	-698.39	1.12	0.18
*$NCONH_2$	-697.84	1.12	0.20
*$NHCONH_2$	-701.87	1.49	0.17
*NH_2CONH_2	-703.93	1.73	0.24

Table S47. Computed total energy (E_{tot}), zero-potential correction energy (E_{zpe}) and entropy contribution (TS, T=298.15 K) of the optimized intermediates for urea production on the MnFe@N_6G system.

MnFe@N_6G	E_{tot} (eV)	E_{ZPE} (eV)	TS (eV)
*N_2	-673.06	0.22	0.10
*NCON	-688.10	0.46	0.14
*NCONH	-692.78	0.79	0.18
*NHCONH	-697.05	1.11	0.21
*$NCONH_2$	-696.75	1.13	0.16
*$NHCONH_2$	-700.70	1.46	0.18
*NH_2CONH_2	-703.73	1.79	0.19

Table S48. Computed total energy (E_{tot}), zero-potential correction energy (E_{zpe}) and entropy contribution (TS, T=298.15 K) of the optimized intermediates for urea production on the MnCo@N_6G system.

MnCo@N_6G	E_{tot} (eV)	E_{ZPE} (eV)	TS (eV)
*N_2	-671.72	0.20	0.13
*NCON	-686.69	0.47	0.14
*NCONH	-691.29	0.79	0.19
*NHCONH	-695.60	1.12	0.18
*$NCONH_2$	-695.29	1.13	0.17
*$NHCONH_2$	-699.90	1.46	0.20
*NH_2CONH_2	-702.56	1.79	0.20

Table S49. Computed total energy (E_{tot}), zero-potential correction energy (E_{zpe}) and entropy contribution (TS, T=298.15 K) of the optimized intermediates for urea production on the MnCu@N_6G system.

MnCu@N_6G	E_{tot} (eV)	E_{ZPE} (eV)	TS (eV)
*N_2	-668.33	0.21	0.10
*NCON	-682.71	0.43	0.20
*NCONH	-687.90	0.80	0.16
*NHCONH	-691.92	1.14	0.15
*$NCONH_2$	-692.21	1.14	0.19
*$NHCONH_2$	-696.73	1.47	0.20
*NH_2CONH_2	-699.62	1.82	0.18

Table S50. Computed total energy (E_{tot}), zero-potential correction energy (E_{zpe}) and entropy contribution (TS, T=298.15 K) of the optimized intermediates for urea production on the MnRu@N_6G system.

MnRu@N_6G	E_{tot} (eV)	E_{ZPE} (eV)	TS (eV)
*N_2	-672.72	0.23	0.09
*NCON	-688.11	0.47	0.14
*NCONH	-692.37	0.77	0.20
*NHCONH	-696.88	1.12	0.17
*$NCONH_2$	-696.51	1.14	0.16
*$NHCONH_2$	-701.04	1.48	0.17
*NH_2CONH_2	-703.38	1.81	0.19

Table S51. Computed total energy (E_{tot}), zero-potential correction energy (E_{zpe}) and entropy contribution (TS, T=298.15 K) of the optimized intermediates for urea production on the MnOs@N_6G system.

MnOs@N_6G	E_{tot} (eV)	E_{ZPE} (eV)	TS (eV)
*N_2	-673.59	0.23	0.08
*NCON	-689.24	0.47	0.14
*NCONH	-693.92	0.81	0.16
*NHCONH	-697.96	1.10	0.19
*$NCONH_2$	-697.82	1.14	0.17
*$NHCONH_2$	-701.54	1.47	0.17
*NH_2CONH_2	-703.85	1.81	0.18

Table S52. Computed total energy (E_{tot}), zero-potential correction energy (E_{zpe}) and entropy contribution (TS, T=298.15 K) of the optimized intermediates for urea production on the FeNi@N_6G system.

FeNi@N_6G	E_{tot} (eV)	E_{ZPE} (eV)	TS (eV)
*N_2	-668.95	0.22	0.08
*NCON	-683.60	0.46	0.13
*NCONH	-688.40	0.79	0.16
*NHCONH	-692.67	1.10	0.17
*$NCONH_2$	-692.66	1.13	0.18
*$NHCONH_2$	-696.63	1.47	0.18
*NH_2CONH_2	-699.81	1.82	0.17

Table S53. Computed total energy (E_{tot}), zero-potential correction energy (E_{zpe}) and entropy contribution (TS, T=298.15 K) of the optimized intermediates for urea production on the FeCu@N_6G system.

FeCu@N_6G	E_{tot} (eV)	E_{ZPE} (eV)	TS (eV)
*N_2	-667.16	0.22	0.09
*NCON	-681.37	0.45	0.15
*NCONH	-686.54	0.80	0.16
*NHCONH	-690.85	1.11	0.18
*$NCONH_2$	-690.84	1.15	0.15
*$NHCONH_2$	-695.18	1.47	0.19
*NH_2CONH_2	-698.32	1.79	0.20

Table S54. Computed total energy (E_{tot}), zero-potential correction energy (E_{zpe}) and entropy contribution (TS, T=298.15 K) of the optimized intermediates for urea production on the FeRu@N_6G system.

FeRu@N_6G	E_{tot} (eV)	E_{ZPE} (eV)	TS (eV)
*N_2	-671.35	0.22	0.09
*NCON	-686.74	0.46	0.13
*NCONH	-691.11	0.79	0.15
*NHCONH	-695.30	1.10	0.20
*$NCONH_2$	-695.18	1.14	0.16
*$NHCONH_2$	-699.59	1.47	0.18
*NH_2CONH_2	-702.11	1.79	0.21

Table S55. Computed total energy (E_{tot}), zero-potential correction energy (E_{zpe}) and entropy contribution (TS, T=298.15 K) of the optimized intermediates for urea production on the CoNi@N_6G system.

CoNi@N_6G	E_{tot} (eV)	E_{ZPE} (eV)	TS (eV)
*N_2	-667.60	0.22	0.09
*NCON	-682.03	0.45	0.14
*NCONH	-686.66	0.79	0.16
*NHCONH	-691.24	1.11	0.16
*$NCONH_2$	-691.25	1.17	0.13
*$NHCONH_2$	-695.57	1.49	0.15
*NH_2CONH_2	-698.60	1.81	0.18

Table S56. Computed total energy (E_{tot}), zero-potential correction energy (E_{zpe}) and entropy contribution (TS, T=298.15 K) of the optimized intermediates for urea production on the CoCu@N_6G system.

CoCu@N_6G	E_{tot} (eV)	E_{ZPE} (eV)	TS (eV)
*N_2	-665.98	0.23	0.08
*NCON	-679.91	0.45	0.16
*NCONH	-684.93	0.80	0.15
*NHCONH	-689.36	1.11	0.17
*$NCONH_2$	-689.53	1.14	0.17
*$NHCONH_2$	-693.64	1.45	0.19
*NH_2CONH_2	-696.97	1.80	0.21

Table S57. Computed total energy (E_{tot}), zero-potential correction energy (E_{zpe}) and entropy contribution (TS, T=298.15 K) of the optimized intermediates for urea production on the CoRh@N_6G system.

**CoRh@N_6G**	**E_{tot} (eV)**	**E_{ZPE} (eV)**	**TS (eV)**
**$*N_2$**	-668.44	0.23	0.08
***NCON**	-683.34	0.46	0.16
***NCONH**	-687.79	0.79	0.14
***NHCONH**	-692.12	1.10	0.18
**$*NCONH_2$**	-692.04	1.15	0.16
**$*NHCONH_2$**	-696.43	1.49	0.16
**$*NH_2CONH_2$**	-699.33	1.79	0.18

Table S58. Computed total energy (E_{tot}), zero-potential correction energy (E_{zpe}) and entropy contribution (TS, T=298.15 K) of the optimized intermediates for urea production on the YCr@N_6G system.

**YCr@N_6G**	**E_{tot} (eV)**	**E_{ZPE} (eV)**	**TS (eV)**
**$*N_2$**	-673.84	0.19	0.13
***NCON**	-690.03	0.45	0.19
***NCONH**	-695.09	0.81	0.14
***NHCONH**	-698.80	1.10	0.18
**$*NCONH_2$**	-698.88	1.09	0.21
**$*NHCONH_2$**	-702.48	1.45	0.19
**$*NH_2CONH_2$**	-704.06	1.77	0.22

Table S59. Computed total energy (E_{tot}), zero-potential correction energy (E_{zpe}) and entropy contribution (TS, T=298.15 K) of the optimized intermediates for urea production on the YMn@N_6G system.

**YMn@N_6G**	**E_{tot} (eV)**	**E_{ZPE} (eV)**	**TS (eV)**
**$*N_2$**	-673.55	0.21	0.09
***NCON**	-689.13	0.48	0.15
***NCONH**	-694.28	0.81	0.15
***NHCONH**	-698.83	1.12	0.16
**$*NCONH_2$**	-698.45	1.11	0.19
**$*NHCONH_2$**	-702.06	1.47	0.20
**$*NH_2CONH_2$**	-704.06	1.77	0.22

Table S60. Computed total energy (E_{tot}), zero-potential correction energy (E_{zpe}) and entropy contribution (TS, T=298.15 K) of the optimized intermediates for urea production on the NbCr@N_6G system.

NbCr@N_6G	E_{tot} (eV)	E_{ZPE} (eV)	TS (eV)
*N_2	-675.02	0.22	0.07
*NCON	-691.33	0.46	0.17
*NCONH	-695.70	0.79	0.19
*NHCONH	-699.89	1.10	0.16
*$NCONH_2$	-699.33	1.14	0.16
*$NHCONH_2$	-703.08	1.46	0.21
*NH_2CONH_2	-705.42	1.75	0.20

Table S61. Computed total energy (E_{tot}), zero-potential correction energy (E_{zpe}) and entropy contribution (TS, T=298.15 K) of the optimized intermediates for urea production on the MoFe@N_6G system.

MoFe@N_6G	E_{tot} (eV)	E_{ZPE} (eV)	TS (eV)
*N_2	-673.07	0.22	0.08
*NCON	-688.91	0.46	0.17
*NCONH	-693.30	-0.79	0.16
*NHCONH	-697.59	1.11	0.20
*$NCONH_2$	-697.02	1.12	0.21
*$NHCONH_2$	-701.28	1.46	0.19
*NH_2CONH_2	-703.71	1.82	0.21

Table S62. Computed total energy (E_{tot}), zero-potential correction energy (E_{zpe}) and entropy contribution (TS, T=298.15 K) of the optimized intermediates for urea production on the MoCo@N_6G system.

MoCo@N_6G	E_{tot} (eV)	E_{ZPE} (eV)	TS (eV)
*N_2	-671.67	0.22	0.08
*NCON	-687.47	0.47	0.14
*NCONH	-692.05	0.78	0.19
*NHCONH	-695.94	1.11	0.17
*$NCONH_2$	-695.89	1.14	0.17
*$NHCONH_2$	-699.91	1.46	0.19
*NH_2CONH_2	-702.37	1.80	0.22

Table S63. Computed total energy (E_{tot}), zero-potential correction energy (E_{zpe}) and entropy contribution (TS, T=298.15 K) of the optimized intermediates for urea production on the RuRh@N_6G system.

RuRh@N_6G	E_{tot} (eV)	E_{ZPE} (eV)	TS (eV)
*N_2	-669.47	0.23	0.08
*NCON	-684.66	0.46	0.14
*NCONH	-689.06	0.79	0.15
*NHCONH	-693.36	1.11	0.16
*$NCONH_2$	-692.85	1.15	0.15
*$NHCONH_2$	-697.40	1.47	0.17
*NH_2CONH_2	-700.19	1.78	0.21

Table S64. Computed total energy (E_{tot}), zero-potential correction energy (E_{zpe}) and entropy contribution (TS, T=298.15 K) of the optimized intermediates for urea production on the RuOs@N_6G system.

RuOs@N_6G	E_{tot} (eV)	E_{ZPE} (eV)	TS (eV)
*N_2	-671.98	0.23	0.07
*NCON	-688.05	0.48	0.13
*NCONH	-692.29	0.80	0.15
*NHCONH	-692.32	1.13	0.15
*$NCONH_2$	-695.74	1.15	0.15
*$NHCONH_2$	-699.74	1.47	0.17
*NH_2CONH_2	-702.80	1.75	0.21

Table S65. Computed total energy (E_{tot}), zero-potential correction energy (E_{zpe}) and entropy contribution (TS, T=298.15 K) of the optimized intermediates for urea production on the RhNi@N_6G system.

RhNi@N_6G	E_{tot} (eV)	E_{ZPE} (eV)	TS (eV)
*N_2	-666.91	0.22	0.09
*NCON	-681.46	0.46	0.13
*NCONH	-686.25	0.78	0.17
*NHCONH	-690.63	1.11	0.17
*$NCONH_2$	-690.61	1.14	0.15
*$NHCONH_2$	-694.63	1.47	0.17
*NH_2CONH_2	-697.75	1.78	0.23

Table S66. Computed total energy (E_{tot}), zero-potential correction energy (E_{zpe}) and entropy contribution (TS, T=298.15 K) of the optimized intermediates for urea production on the RhCu@N_6G system.

RhCu@N_6G	E_{tot} (eV)	E_{ZPE} (eV)	TS (eV)
*N_2	-665.21	0.23	0.09
*NCON	-679.23	0.44	0.16
*NCONH	-684.21	0.79	0.16
*NHCONH	-688.82	1.11	0.18
*$NCONH_2$	-688.92	1.13	0.18
*$NHCONH_2$	-692.98	1.46	0.19
*NH_2CONH_2	-696.05	1.77	0.23

Table S67. Computed total energy (E_{tot}), zero-potential correction energy (E_{zpe}) and entropy contribution (TS, T=298.15 K) of the optimized intermediates for urea production on the RhZn@N_6G system.

RhZn@N_6G	E_{tot} (eV)	E_{ZPE} (eV)	TS (eV)
*N_2	-663.03	0.22	0.10
*NCON	-677.43	0.46	0.15
*NCONH	-683.04	0.81	0.14
*NHCONH	-687.38	1.12	0.17
*$NCONH_2$	-686.75	1.12	0.18
*$NHCONH_2$	-691.24	1.47	0.18
*NH_2CONH_2	-693.95	1.80	0.19

Table S68. Computed total energy (E_{tot}), zero-potential correction energy (E_{zpe}) and entropy contribution (TS, T=298.15 K) of the optimized intermediates for urea production on the HfV@N_6G system.

HfV@N_6G	E_{tot} (eV)	E_{ZPE} (eV)	TS (eV)
*N_2	-675.98	0.21	0.08
*NCON	-692.76	0.49	0.14
*NCONH	-697.34	0.80	0.16
*NHCONH	-701.42	1.10	0.20
*$NCONH_2$	-701.05	1.12	0.20
*$NHCONH_2$	-704.62	1.48	0.18
*NH_2CONH_2	-706.47	1.78	0.19

Table S69. Computed total energy (E_{tot}), zero-potential correction energy (E_{zpe}) and entropy contribution (TS, T=298.15 K) of the optimized intermediates for urea production on the HfCr@N_6G system.

HfCr@N_6G	E_{tot} (eV)	E_{ZPE} (eV)	TS (eV)
*N_2	-676.04	0.22	0.08
*NCON	-692.54	0.47	0.15
*NCONH	-697.34	0.80	0.16
*NHCONH	-701.38	1.09	0.21
*$NCONH_2$	-	-	-
*$NHCONH_2$	-704.25	1.46	0.19
*NH_2CONH_2	-708.43	1.73	0.19

Table S70. Computed total energy (E_{tot}), zero-potential correction energy (E_{zpe}) and entropy contribution (TS, T=298.15 K) of the optimized intermediates for urea production on the HfCo@N_6G system.

HfCo@N_6G	E_{tot} (eV)	E_{ZPE} (eV)	TS (eV)
*N_2	-673.35	0.22	0.08
*NCON	-689.10	0.47	0.16
*NCONH	-693.59	0.80	0.15
*NHCONH	-697.92	1.11	0.17
*$NCONH_2$	-	-	-
*$NHCONH_2$	-701.23	1.45	0.20
*NH_2CONH_2	-703.80	1.76	0.24

Table S71. Computed total energy (E_{tot}), zero-potential correction energy (E_{zpe}) and entropy contribution (TS, T=298.15 K) of the optimized intermediates for urea production on the HfNi@N_6G system.

HfNi@N_6G	E_{tot} (eV)	E_{ZPE} (eV)	TS (eV)
*N_2	-671.89	0.00	0.09
*NCON	-687.26	0.49	0.12
*NCONH	-692.05	0.81	0.15
*NHCONH	-696.49	1.13	0.17
*$NCONH_2$	-696.28	1.15	0.17
*$NHCONH_2$	-700.27	1.48	0.18
*NH_2CONH_2	-702.37	1.77	0.22

Table S72. Computed total energy (E_{tot}), zero-potential correction energy (E_{zpe}) and entropy contribution (TS, T=298.15 K) of the optimized intermediates for urea production on the HfCu@N_6G system.

HfCu@N_6G	E_{tot} (eV)	E_{ZPE} (eV)	TS (eV)
*N_2	-669.62	0.21	0.10
*NCON	-685.00	0.47	0.14
*NCONH	-690.96	0.79	0.16
*NHCONH	-695.28	1.11	0.19
*$NCONH_2$	-	-	-
*$NHCONH_2$	-698.56	1.46	0.20
*NH_2CONH_2	-700.73	1.79	0.17

Table S73. Computed total energy (E_{tot}), zero-potential correction energy (E_{zpe}) and entropy contribution (TS, T=298.15 K) of the optimized intermediates for urea production on the WMn@N_6G system.

WMn@N_6G	E_{tot} (eV)	E_{ZPE} (eV)	TS (eV)
*N_2	-675.85	0.22	0.08
*NCON	-691.88	0.49	0.13
*NCONH	-696.56	0.79	0.17
*NHCONH	-700.26	1.12	0.20
*$NCONH_2$	-700.33	1.16	0.16
*$NHCONH_2$	-703.73	1.48	0.18
*NH_2CONH_2	-705.65	1.79	0.19

Table S74. Computed total energy (E_{tot}), zero-potential correction energy (E_{zpe}) and entropy contribution (TS, T=298.15 K) of the optimized intermediates for urea production on the ReFe@N_6G system.

ReFe@N_6G	E_{tot} (eV)	E_{ZPE} (eV)	TS (eV)
*N_2	-673.48	0.23	0.08
*NCON	-689.48	0.46	0.15
*NCONH	-693.84	0.82	0.16
*NHCONH	-697.54	1.12	0.17
*$NCONH_2$	-697.74	1.13	0.17
*$NHCONH_2$	-701.29	1.46	0.19
*NH_2CONH_2	-703.84	1.78	0.20

Table S75. Computed total energy (E_{tot}), zero-potential correction energy (E_{zpe}) and entropy contribution (TS, T=298.15 K) of the optimized intermediates for urea production on the ReRu@N_6G system.

ReRu@N_6G	E_{tot} (eV)	E_{ZPE} (eV)	TS (eV)
*N_2	-673.40	0.23	0.07
*NCON	-689.70	0.48	0.15
*NCONH	-693.66	0.80	0.16
*NHCONH	-697.56	1.12	0.20
*$NCONH_2$	-697.30	1.15	0.16
*$NHCONH_2$	-701.02	1.47	0.18
*NH_2CONH_2	-703.42	1.82	0.17

Table S76. Computed total energy (E_{tot}), zero-potential correction energy (E_{zpe}) and entropy contribution (TS, T=298.15 K) of the optimized intermediates for urea production on the OsCo@N_6G system.

OsCo@N_6G	E_{tot} (eV)	E_{ZPE} (eV)	TS (eV)
*N_2	-670.93	0.23	0.08
*NCON	-686.53	0.47	0.14
*NCONH	-690.91	0.79	0.15
*NHCONH	-695.02	1.11	0.18
*$NCONH_2$	-695.03	1.14	0.17
*$NHCONH_2$	-698.95	1.45	0.19
*NH_2CONH_2	-701.55	1.81	0.19

Table S77. Computed total energy (E_{tot}), zero-potential correction energy (E_{zpe}) and entropy contribution (TS, T=298.15 K) of the optimized intermediates for urea production on the OsRh@N_6G system.

OsRh@N_6G	E_{tot} (eV)	E_{ZPE} (eV)	TS (eV)
*N_2	-670.33	0.23	0.08
*NCON	-685.98	0.50	0.11
*NCONH	-690.31	0.80	0.15
*NHCONH	-694.43	1.11	0.17
*$NCONH_2$	-694.37	1.15	0.15
*$NHCONH_2$	-698.31	1.46	0.18
*NH_2CONH_2	-700.77	1.78	0.21

Table S78. Computed Gibbs free energy of three main uphill steps ($*N_2$ + CO → *NCON, $*NHCONH_2 + H^+ + e^-$ → $*NH_2CONH_2$, $*NH_2CONH_2$ → * + NH_2CONH_2) on 72 stable systems.

system	ΔG ($*N_2$ + CO → *NCON)	ΔG ($*NHCONH_2 + H^+ + e^-$ → $*NH_2CONH_2$)	ΔG ($*NH_2CONH_2$ → * + NH_2CONH_2)
**$Sc_2@N_6G$**	0.29	2.01	0.48
**$Ti_2@N_6G$**	-1.00	1.73	0.94
**$V_2@N_6G$**	-1.39	1.37	0.98
**$Cr_2@N_6G$**	-0.99	1.50	0.68
**$Mn_2@N_6G$**	-0.01	1.26	1.65
**$Co_2@N_6G$**	0.69	0.61	0.93
**$Ni_2@N_6G$**	1.44	0.76	0.73
**$Cu_2@N_6G$**	1.91	0.10	1.18
**$Y_2@N_6G$**	0.05	2.31	-0.07
**$Rh_2@N_6G$**	0.46	0.70	1.04
**$Pd_2@N_6G$**	1.84	0.14	0.92
**$Ta_2@N_6G$**	-1.50	-0.23	3.33
**$Os_2@N_6G$**	-1.51	1.50	1.02
**$ScTi@N_6G$**	-0.40	1.73	0.97
**$ScV@N_6G$**	-0.86	1.89	0.59
**$ScMn@N_6G$**	-0.21	1.59	1.50
**$ScFe@N_6G$**	0.02	1.12	1.17
**$ScNi@N_6G$**	0.82	0.99	0.74
**$ScCu@N_6G$**	0.75	1.33	0.97
**$ScY@N_6G$**	-0.19	2.23	0.21
**$ScZr@N_6G$**	-1.03	1.96	0.39
**$ScRu@N_6G$**	-0.06	1.49	0.83
**$ScOs@N_6G$**	-0.39	1.58	1.10
**$TiV@N_6G$**	-1.05	1.84	0.99
**$TiFe@N_6G$**	-0.47	1.27	1.01
**$TiCo@N_6G$**	-0.33	1.22	0.85
**$TiNi@N_6G$**	0.24	1.12	0.79
**$TiZn@N_6G$**	0.00	1.51	0.85
**$TiRu@N_6G$**	-0.49	1.81	0.75
**$TiRh@N_6G$**	-0.17	1.54	0.79
**$TiHf@N_6G$**	-1.09	2.30	0.61
**$TiOs@N_6G$**	-0.93	1.56	1.13
**$VMn@N_6G$**	-0.76	1.63	1.36
**$VFe@N_6G$**	-0.42	1.31	0.97
**$VNi@N_6G$**	0.32	1.40	0.32
**$VW@N_6G$**	-1.35	2.61	0.67
**$VOs@N_6G$**	-0.97	0.85	1.62
**$CrMn@N_6G$**	-0.44	1.42	1.68
**$CrFe@N_6G$**	-0.29	1.18	1.11

**CrRh@N_6G**	-0.15	1.34	0.74
**CrOs@N_6G**	-0.83	1.57	0.76
**MnFe@N_6G**	0.41	0.74	0.96
**MnCo@N_6G**	0.54	1.12	0.88
**MnCu@N_6G**	0.98	0.94	1.07
**MnRu@N_6G**	0.05	1.42	1.00
**MnOs@N_6G**	-0.23	1.47	1.78
**FeNi@N_6G**	0.79	0.63	0.81
**FeCu@N_6G**	1.21	0.63	1.32
**FeRu@N_6G**	0.06	1.22	1.02
**CoNi@N_6G**	1.00	0.72	0.81
**CoCu@N_6G**	1.46	0.45	0.96
**CoRh@N_6G**	0.50	0.84	0.83
**YCr@N_6G**	-0.75	1.94	0.32
**YMn@N_6G**	-0.12	1.72	1.27
**NbCr@N_6G**	-0.91	1.42	1.07
**MoFe@N_6G**	-0.44	1.36	0.90
**MoCo@N_6G**	-0.36	1.29	0.94
**RuRh@N_6G**	0.25	0.94	0.86
**RuOs@N_6G**	-0.64	0.63	1.37
**RhNi@N_6G**	0.89	0.58	0.94
**RhCu@N_6G**	1.37	0.45	1.19
**RhZn@N_6G**	1.03	1.06	0.63
**HfV@N_6G**	-1.30	1.89	0.84
**HfCr@N_6G**	-1.07	1.51	0.95
**HfCo@N_6G**	-0.30	1.16	0.96
**HfNi@N_6G**	0.12	1.60	0.97
**HfCu@N_6G**	0.09	1.64	1.27
**WMn@N_6G**	-0.55	1.82	0.84
**ReFe@N_6G**	-0.58	1.21	1.11
**ReRu@N_6G**	-0.88	1.40	0.75
**OsCo@N_6G**	-0.18	1.21	0.97
**OsRh@N_6G**	-0.17	1.27	0.98

Table S79. Computed Gibbs free energy of each elementary step for urea formation on the $Co_2@N_6G$ system without and with solvation effects.

$Co_2@N_6G$	ΔG (without solvation effects)	ΔG (with solvation effects)
$* + N_2 \rightarrow *N_2$	-1.36	-1.60
$*N_2 + CO \rightarrow *NCON$	0.69	0.21
$*NCON + H^+ + e^- \rightarrow *NCONH$	-0.81	-0.78
$*NCONH + H^+ + e^- \rightarrow *NHCONH$	-0.63	-0.60
$*NCONH + H^+ + e^- \rightarrow *NCONH_2$	-0.48	-0.49
$*NHCONH + H^+ + e^- \rightarrow *NHCONH_2$	-0.44	-0.43
$*NCONH_2 + H^+ + e^- \rightarrow *NHCONH_2$	-0.58	-0.54
$*NHCONH_2 + H^+ + e^- \rightarrow *NH_2CONH_2$	0.61	0.51
$*NH_2CONH_2 \rightarrow * + NH_2CONH_2$	0.93	0.63

Table S80. Computed Gibbs free energy of each elementary step for urea formation on the $ScNi@N_6G$ system without and with solvation effects.

$ScNi@N_6G$	ΔG (without solvation effects)	ΔG (with solvation effects)
$* + N_2 \rightarrow *N_2$	-1.78	-1.92
$*N_2 + CO \rightarrow *NCON$	0.82	0.54
$*NCON + H^+ + e^- \rightarrow *NCONH$	-0.91	-0.82
$*NCONH + H^+ + e^- \rightarrow *NHCONH$	-1.05	-0.95
$*NHCONH + H^+ + e^- \rightarrow *NHCONH_2$	0.19	-0.20
$*NHCONH_2 + H^+ + e^- \rightarrow *NH_2CONH_2$	0.99	0.69
$*NH_2CONH_2 \rightarrow * + NH_2CONH_2$	0.74	0.60

Table S81. Computed Gibbs free energy of each elementary step for urea formation on the $MnFe@N_6G$ system without and with solvation effects.

$MnFe@N_6G$	ΔG (without solvation effects)	ΔG (with solvation effects)
$* + N_2 \rightarrow *N_2$	-1.82	-1.46
$*N_2 + CO \rightarrow *NCON$	0.41	-0.12
$*NCON + H^+ + e^- \rightarrow *NCONH$	-0.93	-0.89
$*NCONH + H^+ + e^- \rightarrow *NHCONH$	-0.54	-0.42
$*NCONH + H^+ + e^- \rightarrow *NCONH_2$	-0.17	-0.20
$*NHCONH + H^+ + e^- \rightarrow *NHCONH_2$	0.18	0.05
$*NCONH_2 + H^+ + e^- \rightarrow *NHCONH_2$	-0.19	-0.17
$*NHCONH_2 + H^+ + e^- \rightarrow *NH_2CONH_2$	0.74	0.44
$*NH_2CONH_2 \rightarrow * + NH_2CONH_2$	0.96	0.34

Table S82. Computed Gibbs free energy of each elementary step for urea formation on the FeNi@N_6G system without and with solvation effects.

FeNi@N_6G	ΔG (without solvation effects)	ΔG (with solvation effects)
* + N_2 → *N_2	-1.51	-1.40
*N_2 + CO → *NCON	0.79	0.07
*NCON + H^+ + e^- → *NCONH	-1.04	-0.88
*NCONH + H^+ + e^- → *NHCONH	-0.53	-0.61
*NCONH + H^+ + e^- → *$NCONH_2$	-0.51	-0.55
*NHCONH + H^+ + e^- → *$NHCONH_2$	-0.14	-0.08
*$NCONH_2$ + H^+ + e^- → *$NHCONH_2$	-0.17	-0.14
*$NHCONH_2$ + H^+ + e^- → *NH_2CONH_2	0.63	0.44
*NH_2CONH_2 → * + NH_2CONH_2	0.81	0.40

Table S83. Computed Gibbs free energy of each elementary step for urea formation on the CoNi@N_6G system without and with solvation effects.

CoNi@N_6G	ΔG (without solvation effects)	ΔG (with solvation effects)
* + N_2 → *N_2	-1.36	-1.65
*N_2 + CO → *NCON	1.00	0.45
*NCON + H^+ + e^- → *NCONH	-0.87	-1.00
*NCONH + H^+ + e^- → *NHCONH	-0.81	-0.65
*NCONH + H^+ + e^- → *$NCONH_2$	-0.73	-0.50
*NHCONH + H^+ + e^- → *$NHCONH_2$	-0.49	-0.46
*$NCONH_2$ + H^+ + e^- → *$NHCONH_2$	-0.57	-0.61
*$NHCONH_2$ + H^+ + e^- → *NH_2CONH_2	0.72	0.39
*NH_2CONH_2 → * + NH_2CONH_2	0.81	0.87

Table S84. Computed Gibbs free energy of each elementary step for urea formation on the CoRh@N_6G system without and with solvation effects.

CoRh@N_6G	ΔG (without solvation effects)	ΔG (with solvation effects)
* + N_2 → *N_2	-1.46	-1.71
*N_2 + CO → *NCON	0.50	0.05
*NCON + H^+ + e^- → *NCONH	-0.64	-0.64
*NCONH + H^+ + e^- → *NHCONH	-0.60	-0.42
*NCONH + H^+ + e^- → *$NCONH_2$	-0.46	-0.48
*NHCONH + H^+ + e^- → *$NHCONH_2$	-0.46	-0.64
*$NCONH_2$ + H^+ + e^- → *$NHCONH_2$	-0.61	-0.58
*$NHCONH_2$ + H^+ + e^- → *NH_2CONH_2	0.84	0.32
*NH_2CONH_2 → * + NH_2CONH_2	0.83	0.99

Table S85. Computed Gibbs free energy of each elementary step for urea formation on the RuRh@N_6G system without and with solvation effects.

RuRh@N_6G	ΔG (without solvation effects)	ΔG (with solvation effects)
* + N_2 → *N_2	-1.62	-1.84
*N_2 + CO → *NCON	0.25	-0.03
*NCON + H^+ + e^- → *NCONH	-0.63	-0.77
*NCONH + H^+ + e^- → *NHCONH	-0.54	-0.38
*NCONH + H^+ + e^- → *$NCONH_2$	0.02	-0.01
*NHCONH + H^+ + e^- → *$NHCONH_2$	-0.25	-0.36
*$NCONH_2$ + H^+ + e^- → *$NHCONH_2$	-0.80	-0.74
*$NHCONH_2$ + H^+ + e^- → *NH_2CONH_2	0.94	0.39
*NH_2CONH_2 → * + NH_2CONH_2	0.86	0.93

Table S86. Computed Gibbs free energy of each elementary step for urea formation on the RuRh@N_6G system without and with solvation effects.

RuRh@N_6G	ΔG (without solvation effects)	ΔG (with solvation effects)
* + N_2 → *N_2	-1.56	-1.77
*N_2 + CO → *NCON	0.89	0.35
*NCON + H^+ + e^- → *NCONH	-1.05	-1.00
*NCONH + H^+ + e^- → *NHCONH	-0.61	-0.53
*NCONH + H^+ + e^- → *$NCONH_2$	-0.53	-0.45
*NHCONH + H^+ + e^- → *$NHCONH_2$	-0.19	-0.21
*$NCONH_2$ + H^+ + e^- → *$NHCONH_2$	-0.27	-0.29
*$NHCONH_2$ + H^+ + e^- → *NH_2CONH_2	0.58	0.14
*NH_2CONH_2 → * + NH_2CONH_2	0.94	0.96

Table S87. Computed adsorption free energies of hydrogen atom (ΔG(*H)), oxygen atom (ΔG(*O)), hydroxyl group (ΔG(*OH)), water molecule (ΔG($*OH_2$)), and the Gibbs free energies of *O + H^+ +e^- → *OH and *OH + H^+ +e^- → $*OH_2$ on 72 stable systems.

system	ΔG(*H)	ΔG(*O)	ΔG(*OH)	ΔG($*OH_2$)	ΔG(*O + H^+ +e^- → *OH)	ΔG(*OH + H^+ +e^- → $*OH_2$)
$Sc_2@N_6G$	-1.16	-3.32	-2.86	-0.54	0.46	0.32
$Ti_2@N_6G$	-1.30	-3.50	-2.82	-0.47	0.68	0.34
$V_2@N_6G$	-0.57	-2.21	-2.07	-0.64	0.14	1.43
$Cr_2@N_6G$	-0.44	-1.81	-1.72	-0.13	0.09	1.59
$Mn_2@N_6G$	-0.36	-1.05	-1.54	-0.72	-0.48	0.82
$Co_2@N_6G$	-0.27	0.11	-0.78	-0.17	-0.89	0.61
$Ni_2@N_6G$	-0.03	0.86	-0.48	0.05	-1.34	0.53
$Cu_2@N_6G$	0.18	1.56	0.00	-0.13	-1.55	-0.14
$Y_2@N_6G$	-1.00	-3.25	-2.68	-0.50	0.57	2.18
$Rh_2@N_6G$	-0.58	-0.09	-0.75	0.05	-0.65	0.80
$Pd_2@N_6G$	-0.23	1.42	-0.16	-0.08	-1.58	0.08
$Ta_2@N_6G$	-1.17	-4.67	-3.41	-1.38	1.26	2.03
$Os_2@N_6G$	-0.77	-2.07	-1.48	-0.30	0.60	1.17
$ScTi@N_6G$	-1.48	-3.70	-3.12	-0.99	0.58	2.36
$ScV@N_6G$	-0.56	-2.95	-2.56	-0.76	0.38	2.02
$ScMn@N_6G$	-0.41	-3.07	-3.03	-1.53	0.05	1.67
$ScFe@N_6G$	-0.44	-1.77	-1.79	-0.59	-0.02	1.35
$ScNi@N_6G$	-0.51	-1.56	-1.64	-0.65	-0.08	1.19
$ScCu@N_6G$	-0.66	-1.47	-1.89	-0.85	-0.43	1.27
$ScY@N_6G$	-1.09	-3.38	-2.86	-0.40	0.53	2.69
$ScZr@N_6G$	-1.27	-3.53	-2.92	-0.58	0.60	2.55
$ScRu@N_6G$	-1.05	-2.32	-2.34	-0.89	-0.02	1.69
$ScOs@N_6G$	-1.11	-2.59	-2.32	-0.97	0.27	1.49
$TiV@N_6G$	-0.76	-2.97	-2.54	-0.98	0.43	1.80
$TiFe@N_6G$	-0.39	-1.60	-1.57	-0.87	0.02	0.90
$TiCo@N_6G$	-0.17	-1.62	-1.60	-0.80	0.02	0.97
$TiNi@N_6G$	-0.68	-1.54	-1.52	-0.69	0.02	1.00
$TiZn@N_6G$	-0.99	-1.67	-2.11	-1.02	-0.44	1.28
$TiRu@N_6G$	-0.78	-2.30	-2.11	-0.82	0.19	1.44
$TiRh@N_6G$	-0.58	-1.93	-1.77	-1.18	0.16	0.76
$TiHf@N_6G$	-1.28	-3.72	-2.92	-0.23	0.80	2.83
$TiOs@N_6G$	-0.90	-2.49	-2.14	-0.96	0.35	1.31
$VMn@N_6G$	-1.15	-2.51	-2.53	-1.14	-0.02	1.58
$VFe@N_6G$	-0.55	-0.88	-1.35	-0.29	-0.47	1.34
$VNi@N_6G$	-0.47	-1.63	-1.70	0.05	-0.07	1.92
$VW@N_6G$	-1.26	-3.04	-2.43	-1.32	0.61	1.23
$VOs@N_6G$	-1.02	-2.03	-2.04	-0.91	0.00	1.28
$CrMn@N_6G$	-0.38	-2.58	-2.68	-0.96	-0.10	1.92

**CrFe@N_6G**	-0.62	-1.05	-1.23	-0.56	-0.19	0.88
**CrRh@N_6G**	-0.37	-1.25	-1.44	-0.66	-0.19	0.87
**CrOs@N_6G**	-0.85	-2.10	-1.49	0.15	-0.61	1.84
**MnFe@N_6G**	0.10	-0.83	-1.00	-0.35	-0.18	0.94
**MnCo@N_6G**	-0.61	0.23	-0.21	0.52	-0.44	0.97
**MnCu@N_6G**	-0.47	-0.93	-0.93	0.39	0.01	1.49
**MnRu@N_6G**	-0.68	-1.28	-0.87	-0.41	-0.41	0.70
**MnOs@N_6G**	-1.74	-2.99	-2.50	-1.42	0.49	1.29
**FeNi@N_6G**	0.05	-0.76	-0.92	0.33	-0.16	1.50
**FeCu@N_6G**	-0.64	-1.08	-1.21	-0.28	-0.13	1.08
**FeRu@N_6G**	-0.54	-0.82	-0.92	-0.24	-0.10	0.93
**CoNi@N_6G**	-0.15	0.16	-0.78	-0.23	-0.94	0.76
**CoCu@N_6G**	-0.28	0.06	-0.79	-0.24	-0.85	0.75
**CoRh@N_6G**	-0.48	0.21	-0.71	-0.21	-0.92	0.77
**YCr@N_6G**	-0.93	-2.73	-2.30	-0.52	0.43	2.00
**YMn@N_6G**	-1.79	-3.28	-3.14	-1.04	0.14	2.28
**NbCr@N_6G**	-0.60	-2.88	-2.38	-0.49	0.50	2.06
**MoFe@N_6G**	-0.68	-1.80	-1.71	-0.02	0.09	1.93
**MoCo@N_6G**	-0.79	-2.24	-2.01	-0.48	0.23	1.73
**RuRh@N_6G**	-0.49	-1.14	-0.90	-0.36	0.24	0.73
**RuOs@N_6G**	-0.78	-1.09	-1.39	-0.78	-0.31	0.79
**RhNi@N_6G**	-0.51	-0.03	-0.53	-0.14	-0.50	0.60
**RhCu@N_6G**	-0.59	-0.11	-0.58	-0.02	-0.47	0.80
**RhZn@N_6G**	-1.01	0.16	-0.50	-0.02	-0.65	0.75
**HfV@N_6G**	-0.98	-3.40	-2.73	-0.38	0.67	2.54
**HfCr@N_6G**	-1.31	-3.31	-2.77	-0.50	0.54	2.44
**HfCo@N_6G**	-1.04	-2.21	-2.09	-1.04	0.12	1.23
**HfNi@N_6G**	-0.69	-2.21	-2.10	-0.09	0.11	2.24
**HfCu@N_6G**	-1.06	-2.45	-2.43	-0.40	0.02	2.24
**WMn@N_6G**	-1.23	-2.82	-2.55	-1.08	0.27	1.64
**ReFe@N_6G**	-1.15	-1.80	-1.41	-0.75	0.39	0.83
**ReRu@N_6G**	-1.11	-2.49	-1.94	-0.18	0.56	1.99
**OsCo@N_6G**	-0.70	-2.11	-1.61	-0.22	0.50	1.62
**OsRh@N_6G**	-0.77	-2.03	-1.56	-0.73	0.47	0.93

Table S88. Computed Gibbs free energies of the first hydrogenated step $*N_2 + H^+ + e^-$ → *NNH, the potential determined steps (PDS) and the corrsponding ΔG values (ΔG (PDS)) for N_2 reduction to NH_3 on 72 stable systems.

system	ΔG ($*N_2 + H^+ + e^-$ → *NNH)	PDS	ΔG (PDS)
$Sc_2@N_6G$	-0.01	$*NH_2 + H^+ + e^-$ → $*NH_3$	1.56
$Ti_2@N_6G$	-0.14	$*NH_2 + H^+ + e^-$ → $*NH_3$	1.25
$V_2@N_6G$	-0.11	$*NH_2 + H^+ + e^-$ → $*NH_3$	0.70
$Cr_2@N_6G$	0.14	$*NHNH + H^+ + e^-$ → $*NHNH_2$	0.58
$Mn_2@N_6G$	0.38	$*NHNH + H^+ + e^-$ → $*NHNH_2$	0.45
$Co_2@N_6G$	0.81	$*N_2 + H^+ + e^-$ → *NNH	0.81
$Ni_2@N_6G$	0.87	$*N_2 + H^+ + e^-$ → *NNH	0.87
$Cu_2@N_6G$	1.48	$*N_2 + H^+ + e^-$ → *NNH	1.48
$Y_2@N_6G$	0.00	$*NH_2 + H^+ + e^-$ → $*NH_3$	1.32
$Rh_2@N_6G$	0.55	$*N_2 + H^+ + e^-$ → *NNH	0.55
$Pd_2@N_6G$	0.77	$*N_2 + H^+ + e^-$ → *NNH	0.77
$Ta_2@N_6G$	-0.59	$*NNH + H^+ + e^-$ → $*NNH_2$	0.41
$Os_2@N_6G$	0.00	$*NHNH + H^+ + e^-$ → $*NHNH_2$	0.76
$ScTi@N_6G$	-0.13	$*NH_2 + H^+ + e^-$ → $*NH_3$	1.38
$ScV@N_6G$	-0.26	$*NH_2 + H^+ + e^-$ → $*NH_3$	1.22
$ScMn@N_6G$	0.22	$*NH_2 + H^+ + e^-$ → $*NH_3$	1.83
$ScFe@N_6G$	0.45	$*NH_2 + H^+ + e^-$ → $*NH_3$	0.79
$ScNi@N_6G$	1.00	$*N_2 + H^+ + e^-$ → *NNH	1.00
$ScCu@N_6G$	0.54	$*NH_2 + H^+ + e^-$ → $*NH_3$	0.71
$ScY@N_6G$	0.12	$*NH_2 + H^+ + e^-$ → $*NH_3$	1.41
$ScZr@N_6G$	0.00	$*NH_2 + H^+ + e^-$ → $*NH_3$	1.38
$ScRu@N_6G$	0.32	$*NH_2 + H^+ + e^-$ → $*NH_3$	1.19
$ScOs@N_6G$	0.07	$*NH_2 + H^+ + e^-$ → $*NH_3$	1.11
$TiV@N_6G$	0.15	$*NH_2 + H^+ + e^-$ → $*NH_3$	0.92
$TiFe@N_6G$	0.40	$*NH_2 + H^+ + e^-$ → $*NH_3$	0.75
$TiCo@N_6G$	0.60	$*NH_2 + H^+ + e^-$ → $*NH_3$	0.70
$TiNi@N_6G$	0.80	$*N_2 + H^+ + e^-$ → *NNH	0.80
$TiZn@N_6G$	0.07	$*NH_2 + H^+ + e^-$ → $*NH_3$	0.31
$TiRu@N_6G$	0.46	$*NH_2 + H^+ + e^-$ → $*NH_3$	0.97
$TiRh@N_6G$	0.49	$*NH_2 + H^+ + e^-$ → $*NH_3$	1.02
$TiHf@N_6G$	-0.24	$*NH_2 + H^+ + e^-$ → $*NH_3$	1.24
$TiOs@N_6G$	0.18	$*NH_2 + H^+ + e^-$ → $*NH_3$	0.90
$VMn@N_6G$	0.11	$*NH_2 + H^+ + e^-$ → $*NH_3$	0.80
$VFe@N_6G$	0.43	$*N_2 + H^+ + e^-$ → *NNH	0.43
$VNi@N_6G$	0.74	$*NH_2 + H^+ + e^-$ → $*NH_3$	0.78
$VW@N_6G$	-0.25	$*NH_2 + H^+ + e^-$ → $*NH_3$	0.88
$VOs@N_6G$	0.22	$*NH_2 + H^+ + e^-$ → $*NH_3$	0.62
$CrMn@N_6G$	0.29	$*NH_2 + H^+ + e^-$ → $*NH_3$	0.61
$CrFe@N_6G$	0.54	$*N_2 + H^+ + e^-$ → *NNH	0.54
$CrRh@N_6G$	0.59	$*N_2 + H^+ + e^-$ → *NNH	0.59

**CrOs@N_6G**	0.31	$*N_2 + H^+ + e^- \rightarrow *NNH$	0.53
**MnFe@N_6G**	0.58	$*N_2 + H^+ + e^- \rightarrow *NNH$	0.58
**MnCo@N_6G**	0.69	$*N_2 + H^+ + e^- \rightarrow *NNH$	0.69
**MnCu@N_6G**	0.63	$*N_2 + H^+ + e^- \rightarrow *NNH$	0.63
**MnRu@N_6G**	0.48	$*N_2 + H^+ + e^- \rightarrow *NNH$	0.48
**MnOs@N_6G**	0.24	$*NH_2 + H^+ + e^- \rightarrow *NH_3$	0.61
**FeNi@N_6G**	0.72	$*N_2 + H^+ + e^- \rightarrow *NNH$	0.72
**FeCu@N_6G**	0.61	$*N_2 + H^+ + e^- \rightarrow *NNH$	0.61
**FeRu@N_6G**	0.63	$*N_2 + H^+ + e^- \rightarrow *NNH$	0.63
**CoNi@N_6G**	0.77	$*N_2 + H^+ + e^- \rightarrow *NNH$	0.77
**CoCu@N_6G**	0.81	$*N_2 + H^+ + e^- \rightarrow *NNH$	0.81
**CoRh@N_6G**	0.64	$*N_2 + H^+ + e^- \rightarrow *NNH$	0.64
**YCr@N_6G**	-0.07	$*NH_2 + H^+ + e^- \rightarrow *NH_3$	1.10
**YMn@N_6G**	0.23	$*NH_2 + H^+ + e^- \rightarrow *NH_3$	1.11
**NbCr@N_6G**	0.22	$*NH_2 + H^+ + e^- \rightarrow *NH_3$	1.13
**MoFe@N_6G**	0.23	$*NH_2 + H^+ + e^- \rightarrow *NH_3$	0.29
**MoCo@N_6G**	0.27	$*NHNH + H^+ + e^- \rightarrow *NHNH_2$	0.43
**RuRh@N_6G**	0.60	$*N_2 + H^+ + e^- \rightarrow *NNH$	0.60
**RuOs@N_6G**	0.27	$*NHNH + H^+ + e^- \rightarrow *NHNH_2$	0.75
**RhNi@N_6G**	0.62	$*N_2 + H^+ + e^- \rightarrow *NNH$	0.62
**RhCu@N_6G**	0.72	$*N_2 + H^+ + e^- \rightarrow *NNH$	0.72
**RhZn@N_6G**	0.18	$*NNH + H^+ + e^- \rightarrow *NHNH$	0.37
**HfV@N_6G**	-0.13	$*NH_2 + H^+ + e^- \rightarrow *NH_3$	1.27
**HfCr@N_6G**	-0.13	$*NH_2 + H^+ + e^- \rightarrow *NH_3$	1.10
**HfCo@N_6G**	0.41	$*NHNH + H^+ + e^- \rightarrow *NHNH_2$	0.68
**HfNi@N_6G**	0.65	$*N_2 + H^+ + e^- \rightarrow *NNH$	0.65
**HfCu@N_6G**	0.31	$*NH_2 + H^+ + e^- \rightarrow *NH_3$	0.60
**WMn@N_6G**	0.20	$*NH_2 + H^+ + e^- \rightarrow *NH_3$	0.93
**ReFe@N_6G**	0.31	$*NH_2 + H^+ + e^- \rightarrow *NH_3$	0.80
**ReRu@N_6G**	0.38	$*NH_2 + H^+ + e^- \rightarrow *NH_3$	0.54
**OsCo@N_6G**	0.59	$*NH_2 + H^+ + e^- \rightarrow *NH_3$	0.61
**OsRh@N_6G**	0.44	$*N_2 + H^+ + e^- \rightarrow *NNH$	0.44

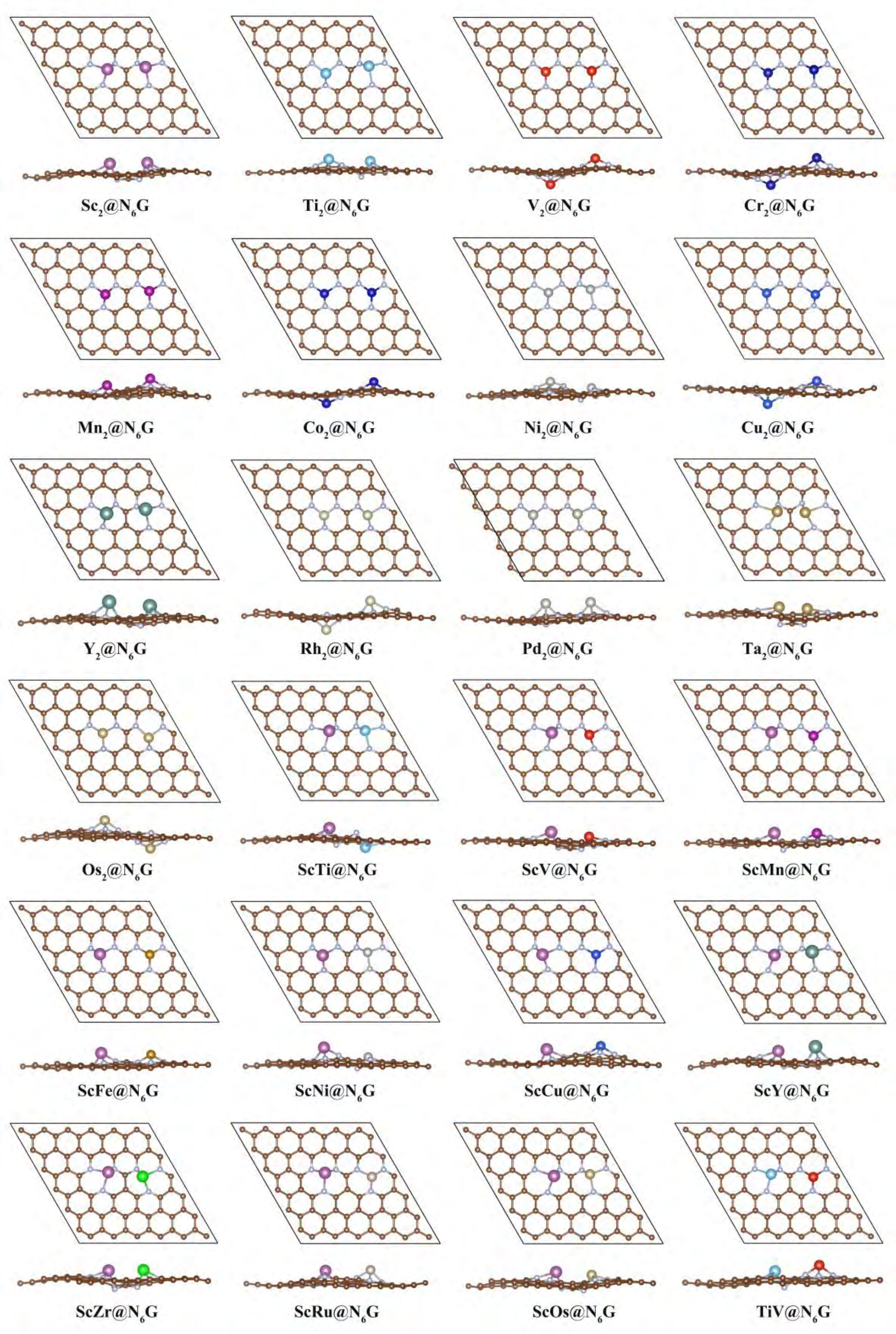

Figure S1. (a) Equilibrium structures of our systems at ambient temperatures through 10 *ps* AIMD simulations.

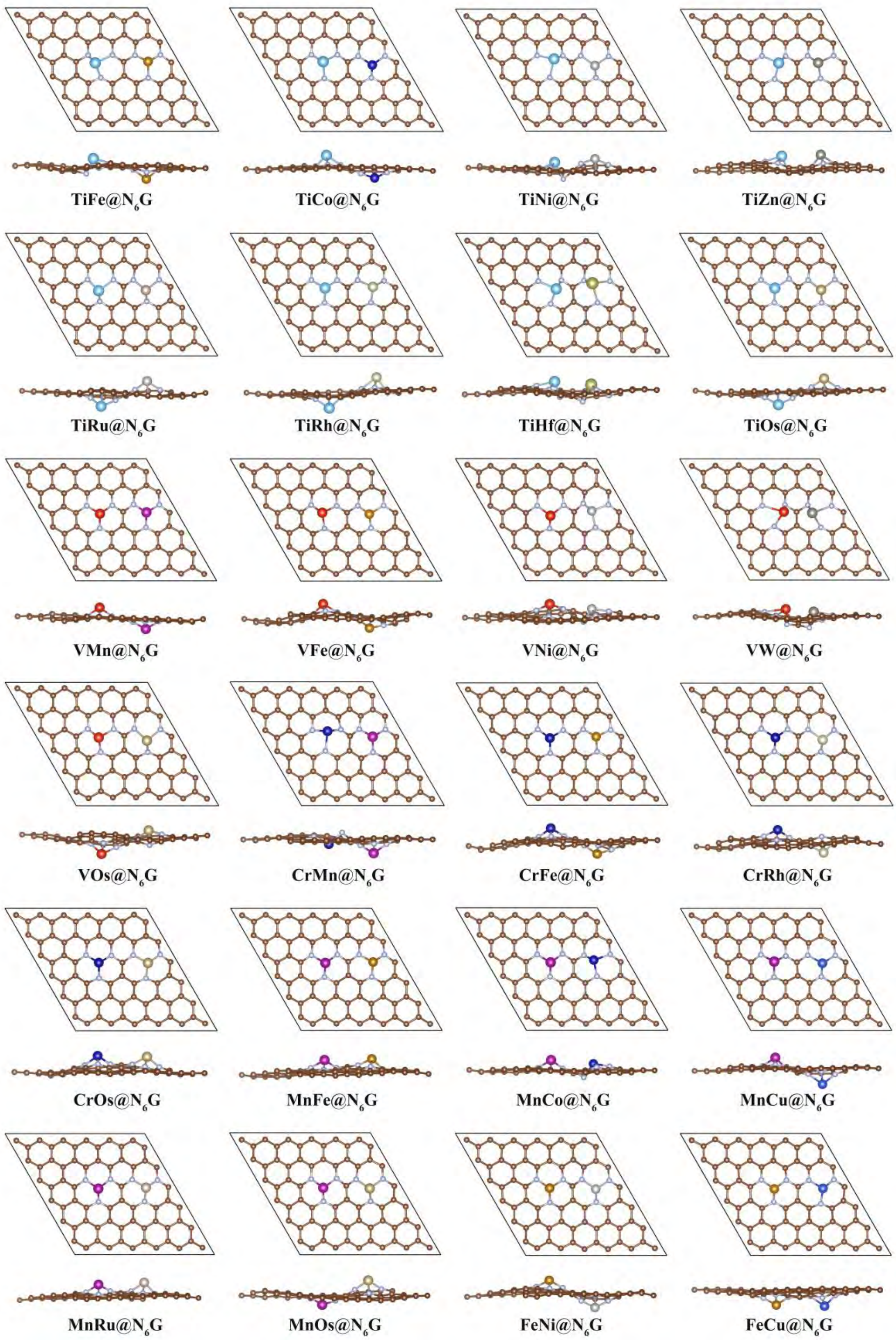

Figure S1. (b) Equilibrium structures of our systems at ambient temperatures through 10 *ps* AIMD simulations.

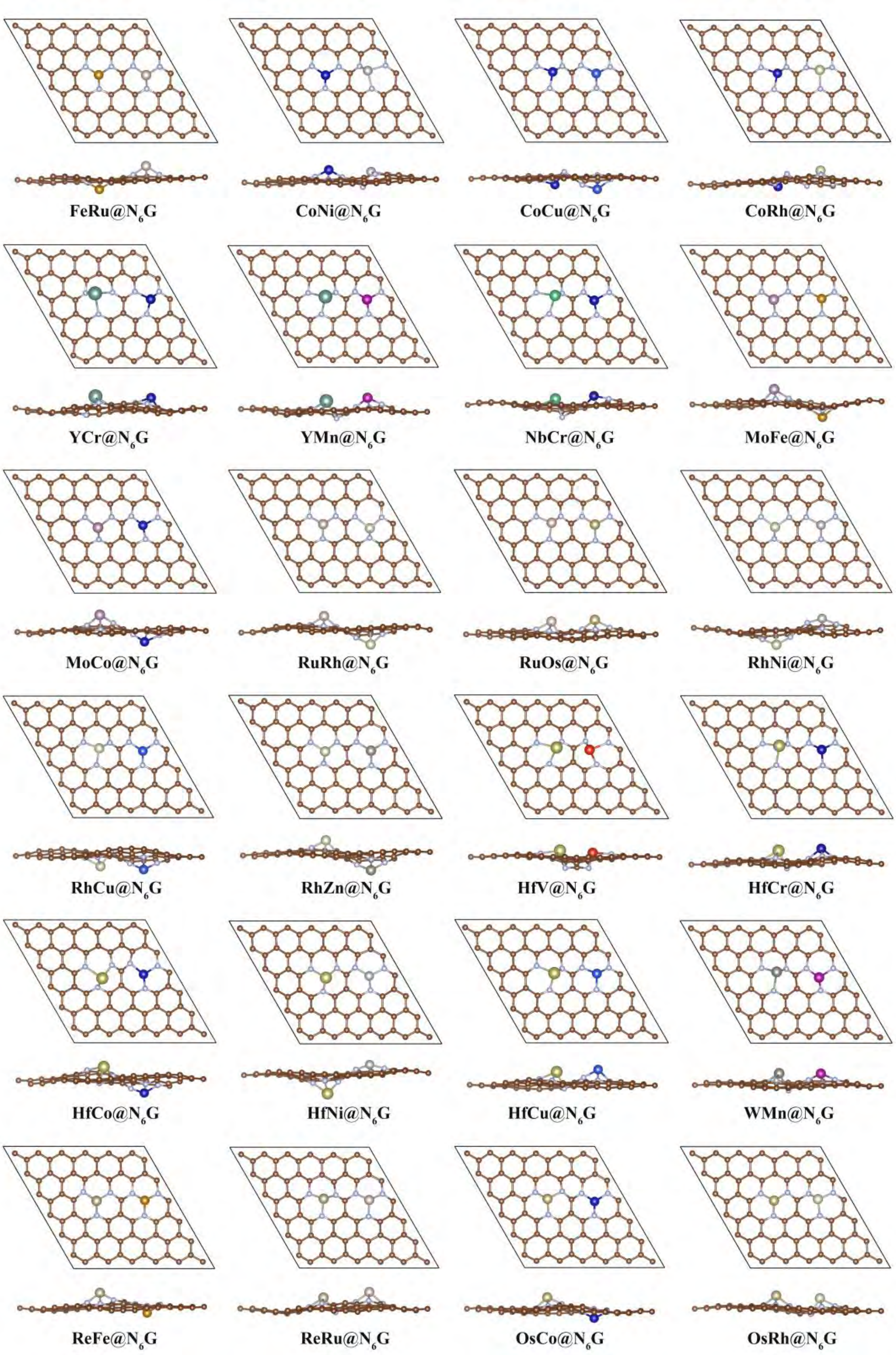

Figure S1. (c) Equilibrium structures of our systems at ambient temperatures through 10 *ps* AIMD simulations.

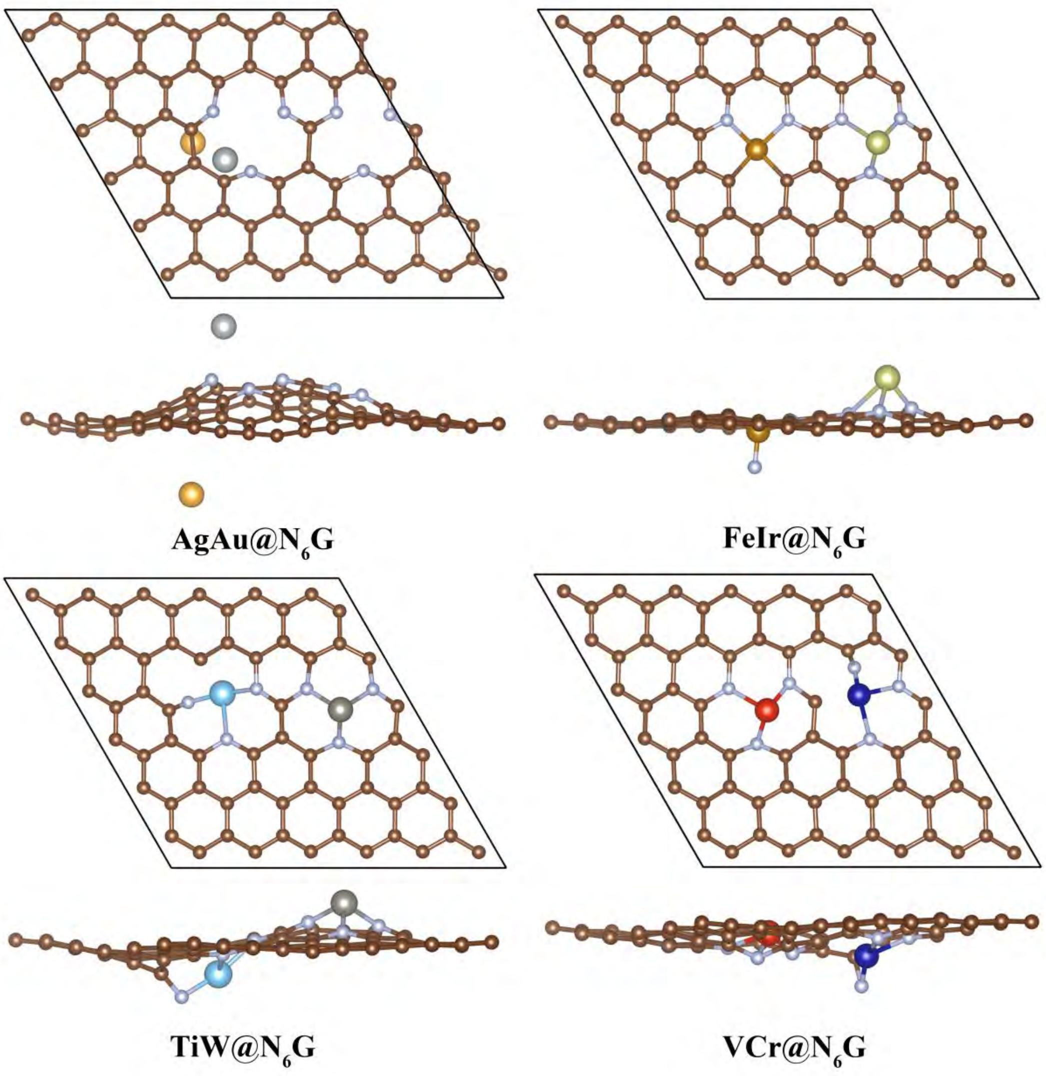

Figure S1. (d) Four representative schematic diagrams of the unstable structures that have been excluded in our work. Note: the judging criteria of whether the structures are stable are the structural integrity (especially the MN_3-$M'N_3$ moiety).

For the AgAu@N_6G system, Ag and Au atoms are far away from the initial sites, suggesting that Ag and Au atoms cannot be anchored on the MN_3-$M'N_3$ moiety, thus the AgAu@N_6G system is unstable. For the FeIr@N_6G system, one coordinated N atom bonding with Fe atom is squeezed out of the plane, and the four-coordination FeN_2C_2 moiety is finally constructed, thus the original FeN_3-IrN_3 moiety is also unstable. For TiW@N_6G and VCr@N_6G systems, the C-N bonds are broken although there are still TiN_3-WN_3 moiety and VN_3-CrN_3 moiety, thus TiW@N_6G and VCr@N_6G systems are both unstable.

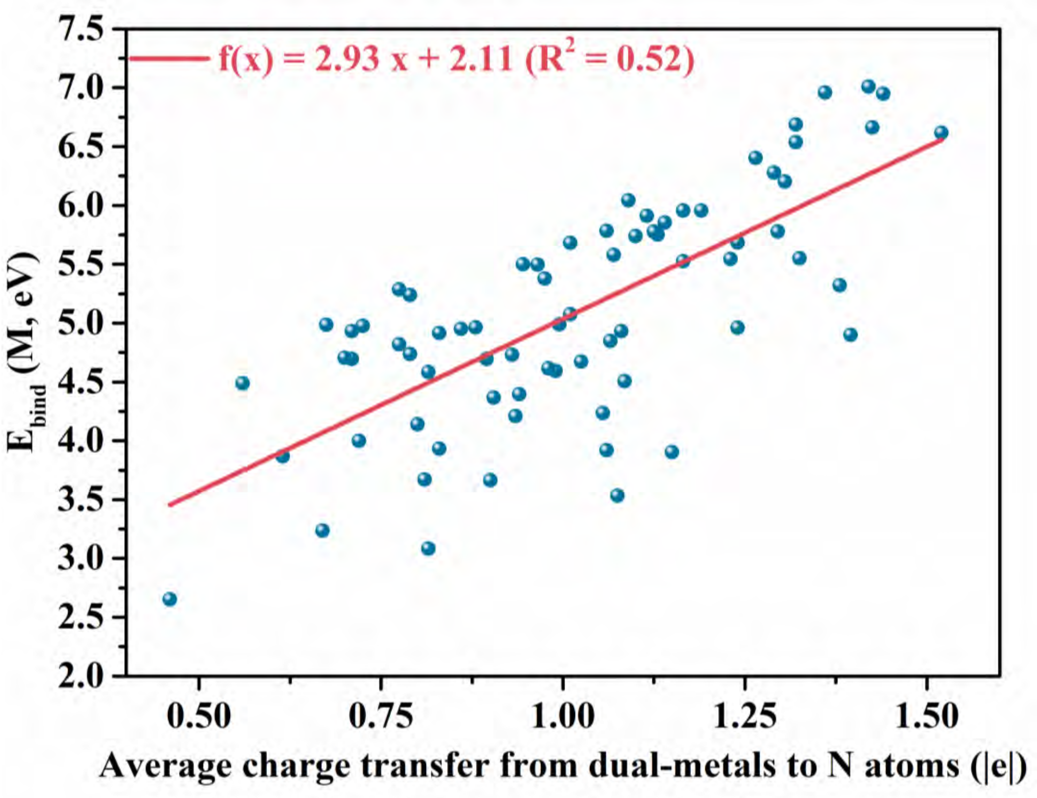

Figure S2. Average binding energies of dual-metals anchored on N-decorated graphene versus average charge transfer from dual-metals to N atoms.

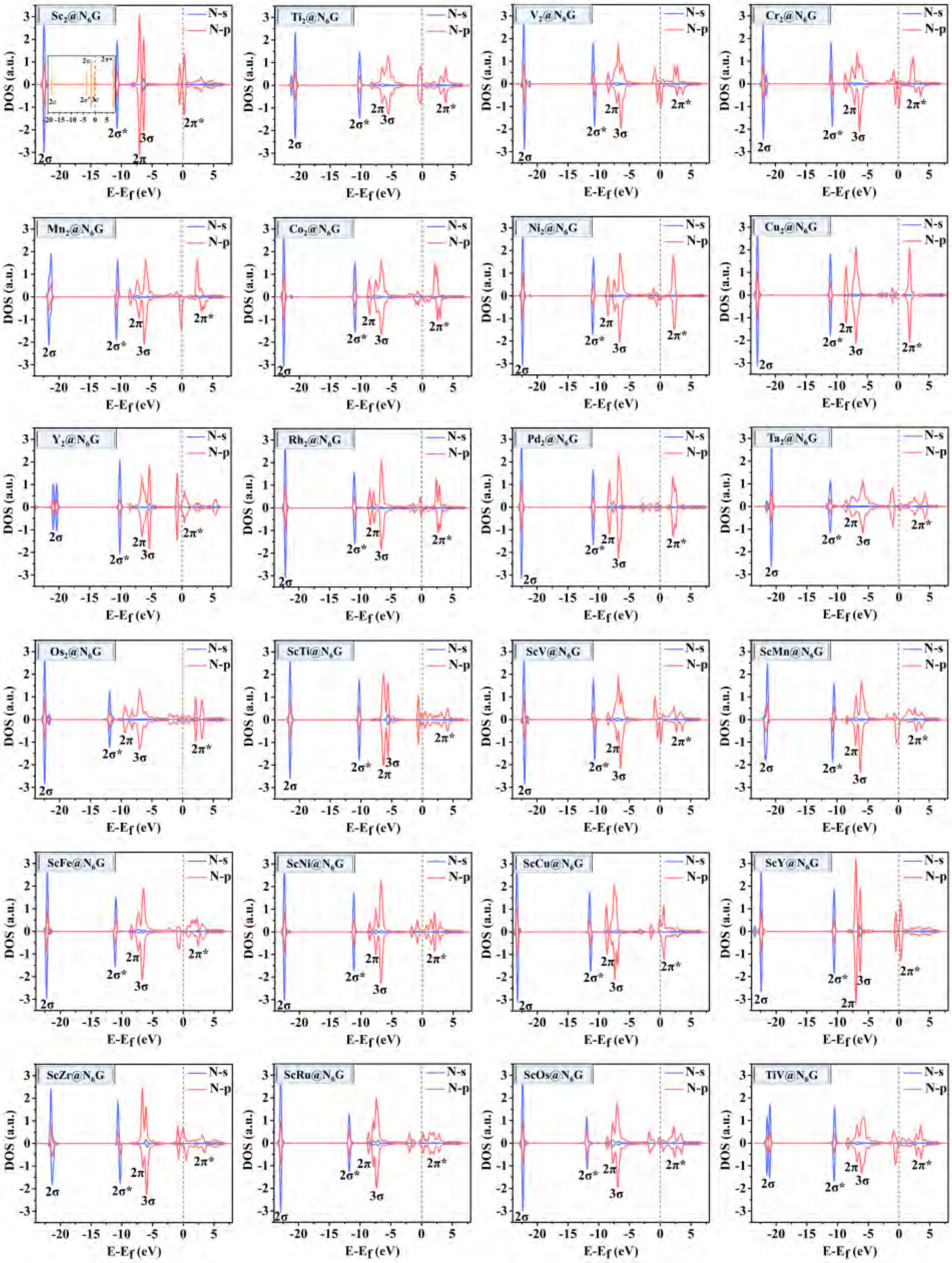

Figure S3. (a) Computed partial density of states (PDOS) of side-on-c N_2 adsorbed on 72 stable systems using PBE functional. The molecular orbitals of free N_2 molecule are inset. The Fermi level is set to zero.

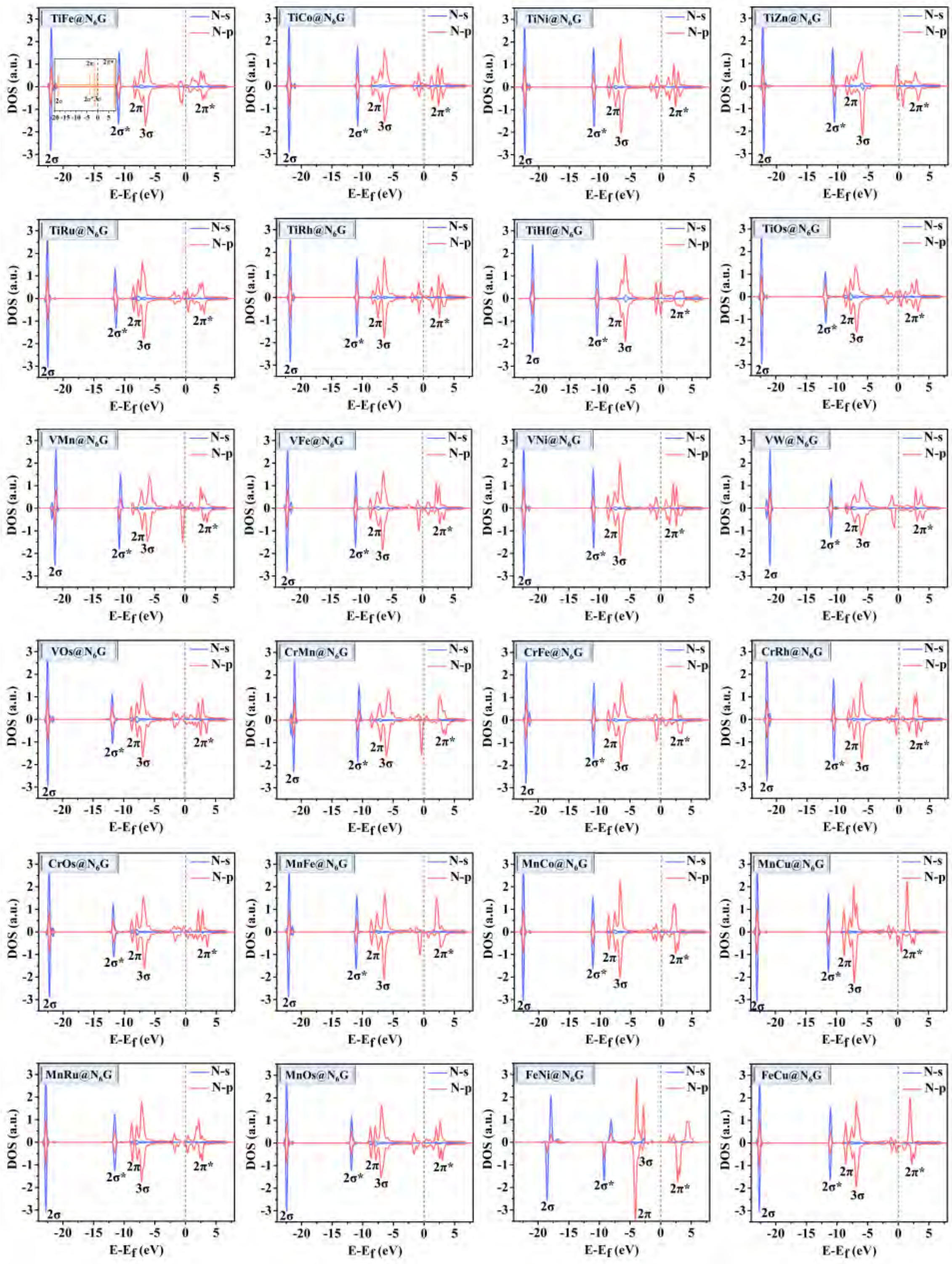

Figure S3. (b) Computed partial density of states (PDOS) of side-on-c N_2 adsorbed on 72 stable systems using PBE functional. The molecular orbitals of free N_2 molecule are inset. The Fermi level is set to zero.

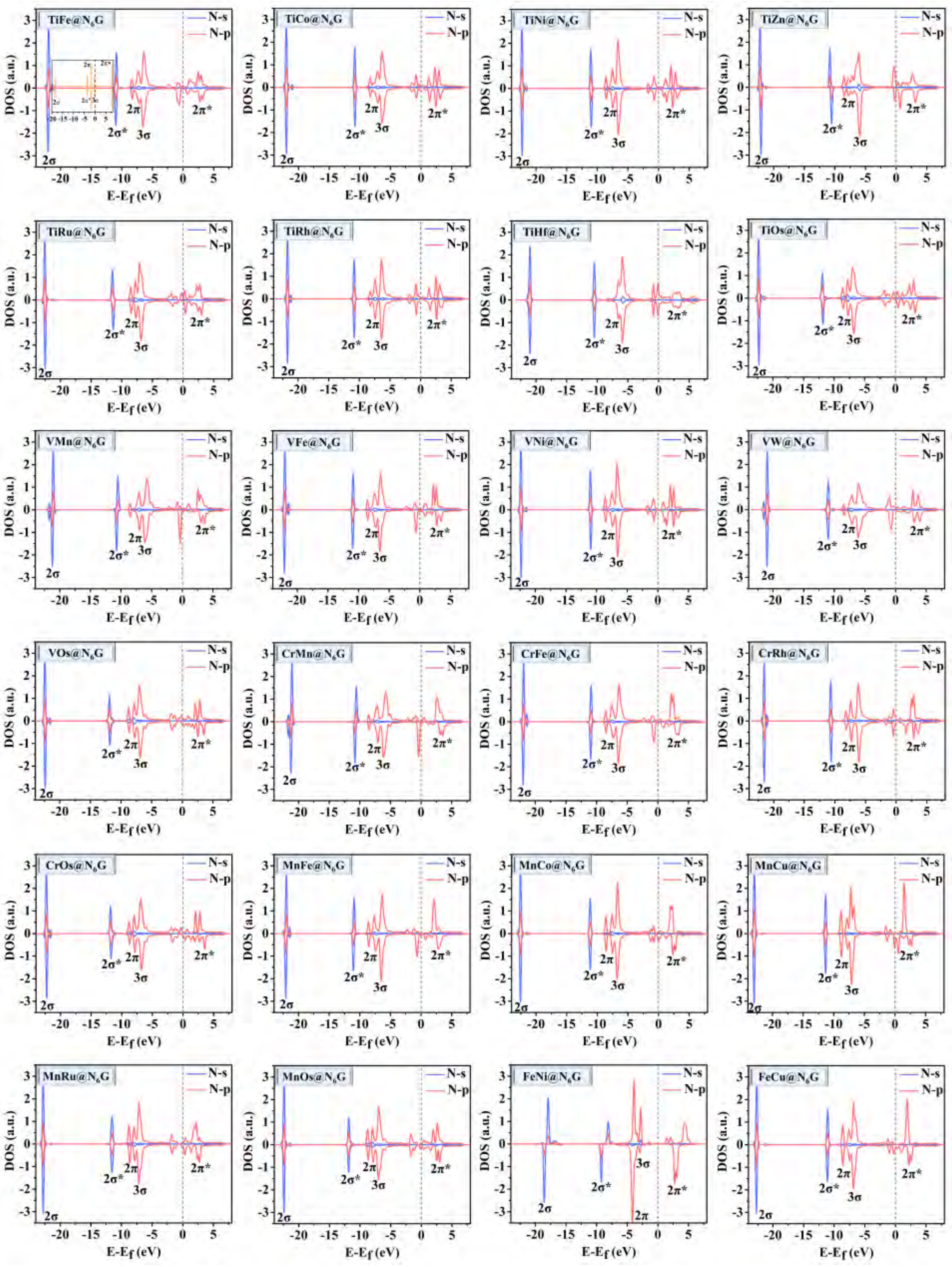

Figure S3. (c) Computed partial density of states (PDOS) of side-on-c N_2 adsorbed on 72 stable systems using PBE functional. The molecular orbitals of free N_2 molecule are inset. The Fermi level is set to zero.

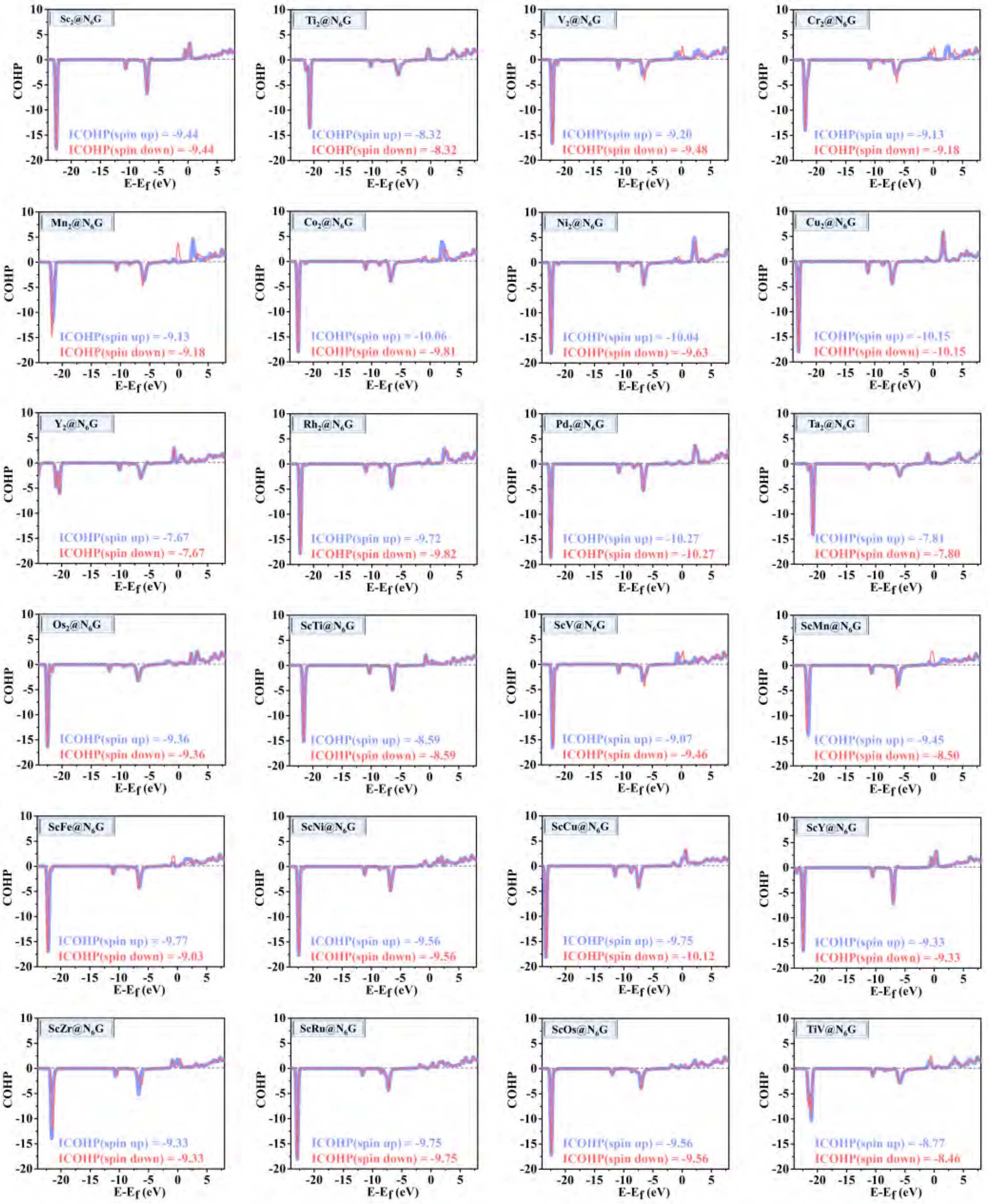

Figure S4. (a) Computed crystal orbital Hamilton populations (COHPs) for the N-N bonds on 72 stable systems.

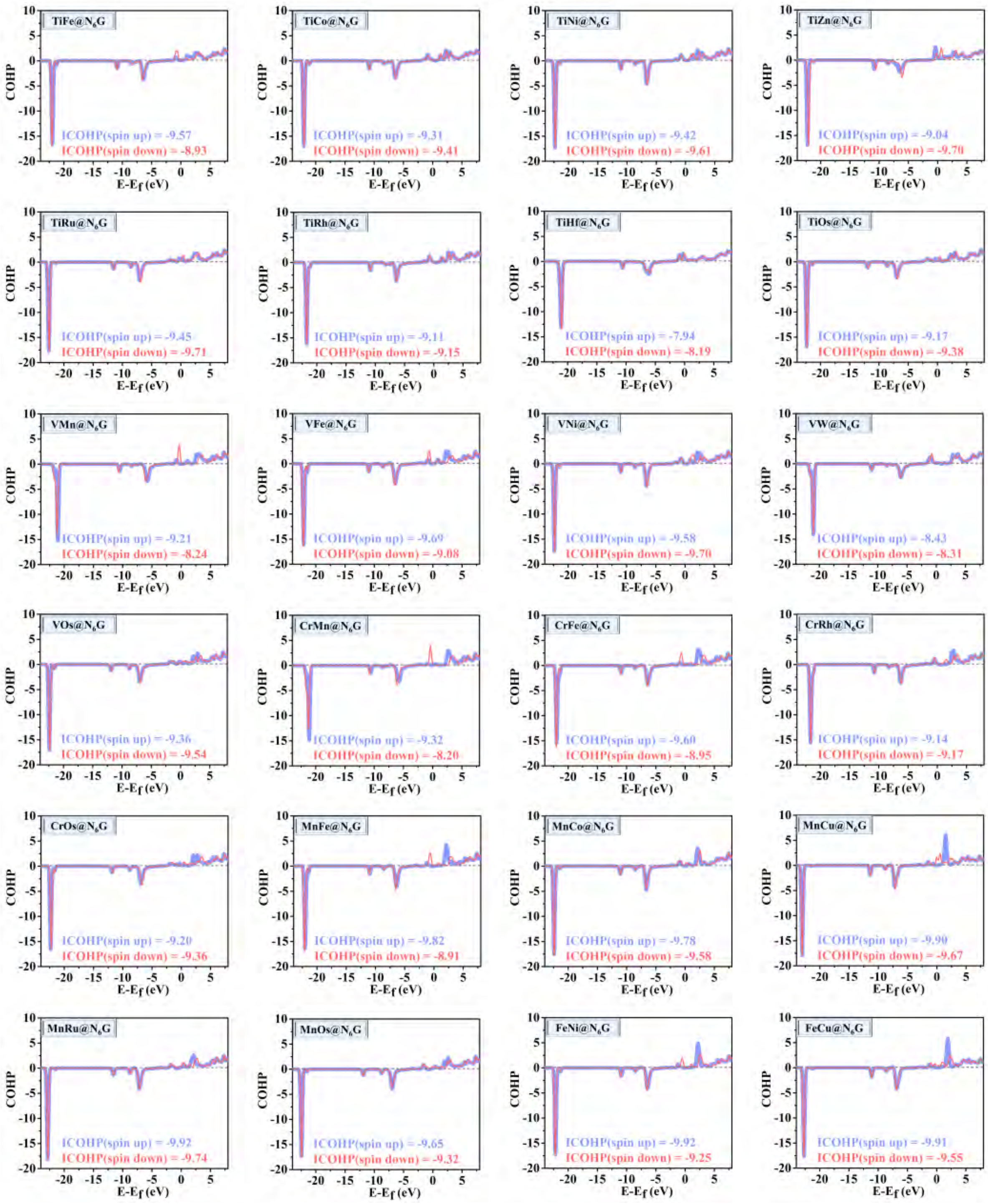

Figure S4. (b) Computed crystal orbital Hamilton populations (COHPs) for the N-N bonds on 72 stable systems.

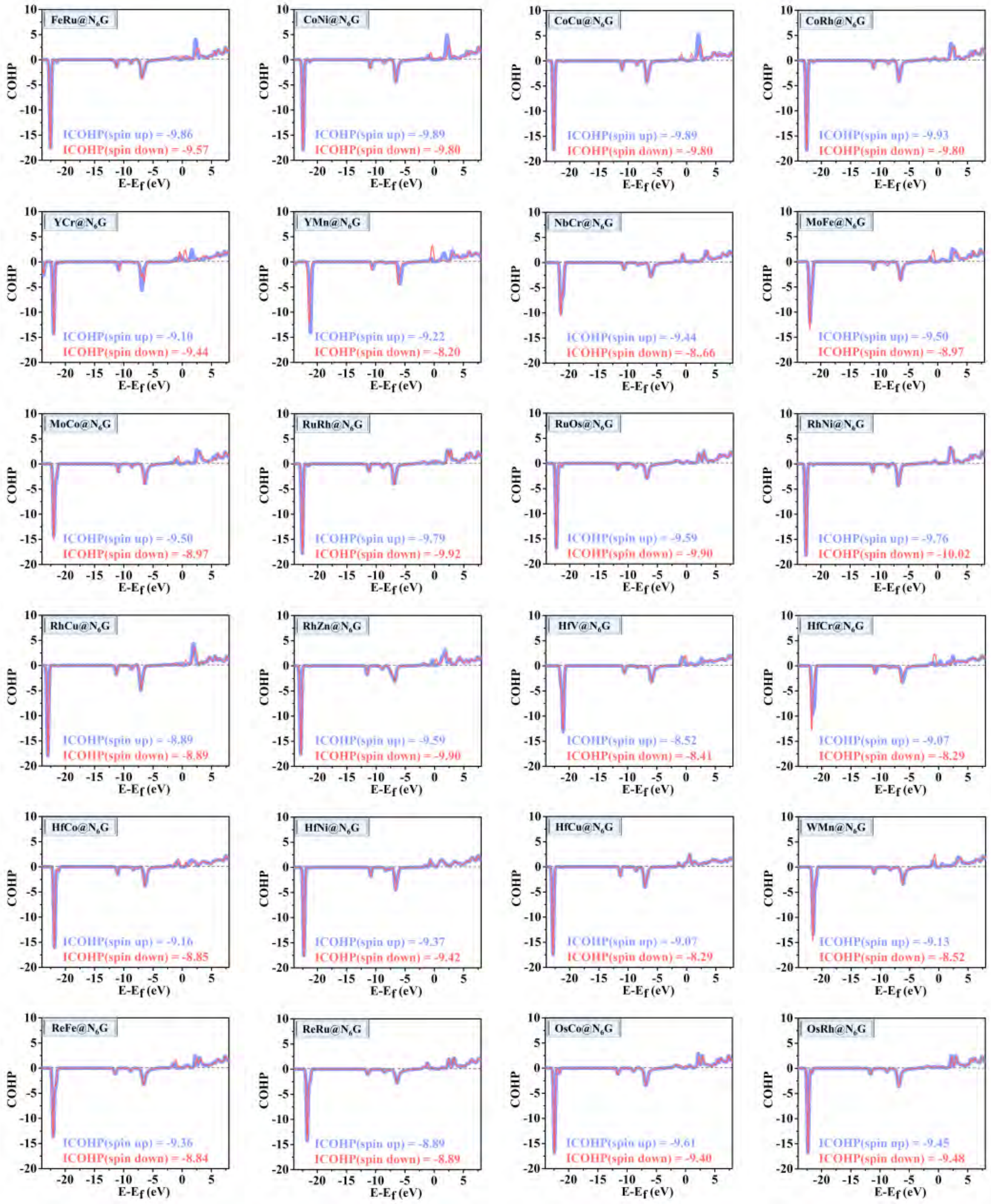

Figure S4. (c) Computed crystal orbital Hamilton populations (COHPs) for the N-N bonds on 72 stable systems.

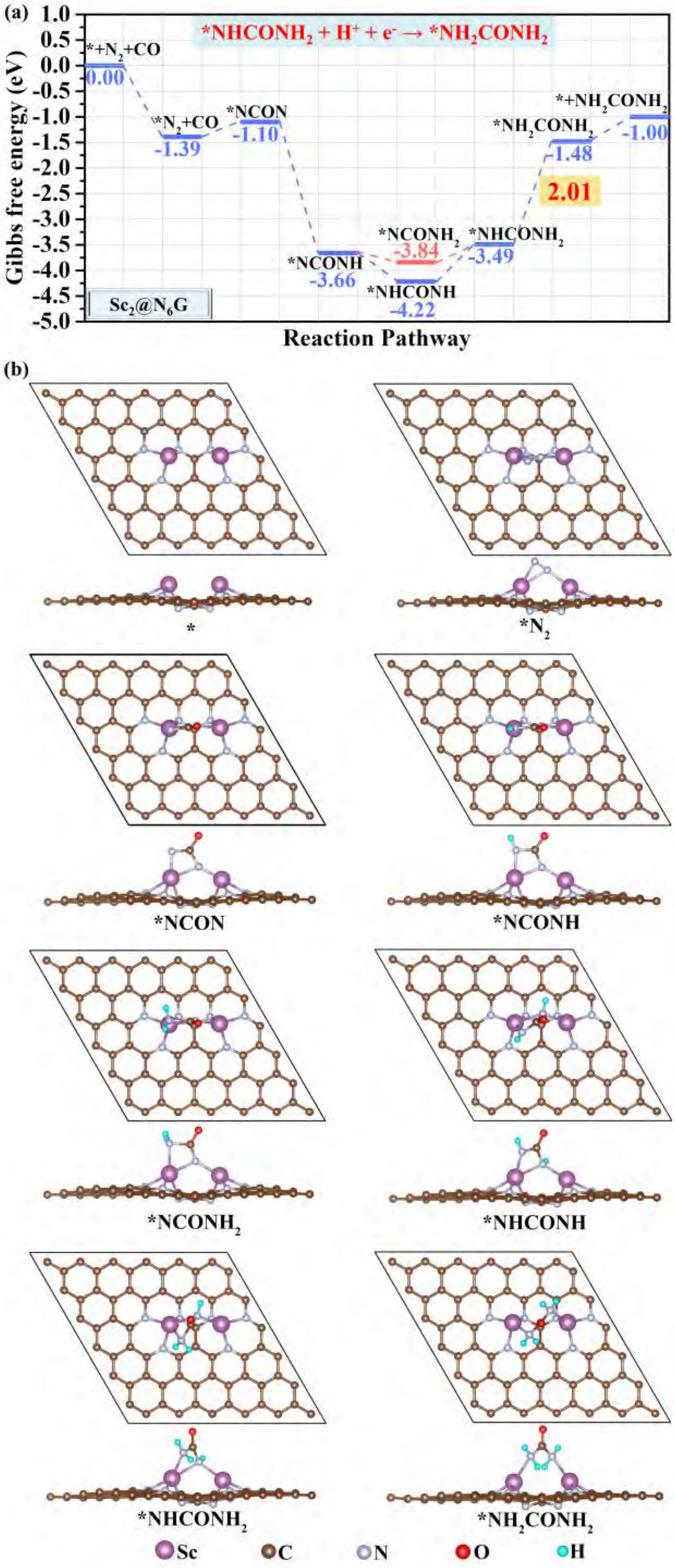

Figure S5. (a) Gibbs free energy diagram for urea production on the $Sc_2@N_6G$ system. (b) Optimized structures of various intermediates along the hydrogenation pathway of urea production on the $Sc_2@N_6G$ system.

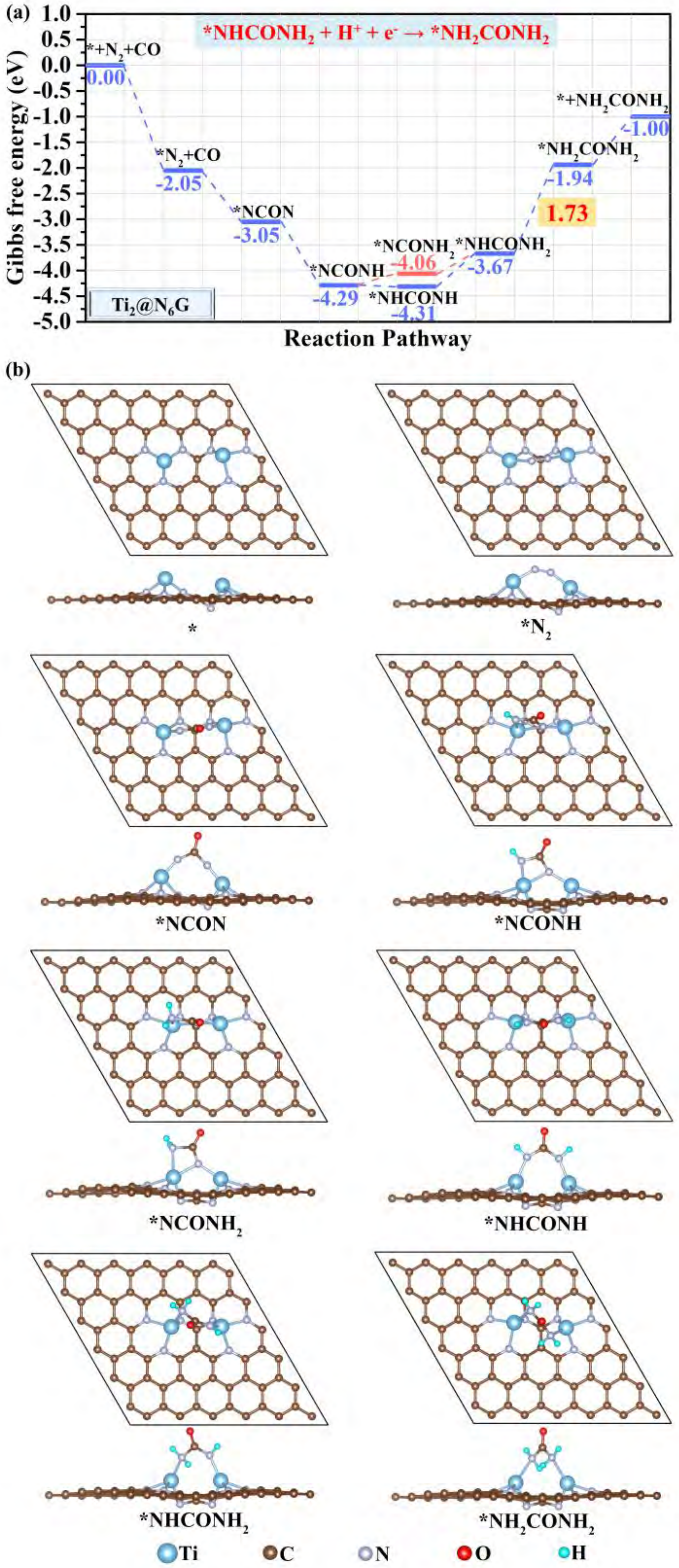

Figure S6. (a) Gibbs free energy diagram for urea production on the $Ti_2@N_6G$ system. (b) Optimized structures of various intermediates along the hydrogenation pathway of urea production on the $Ti_2@N_6G$ system.

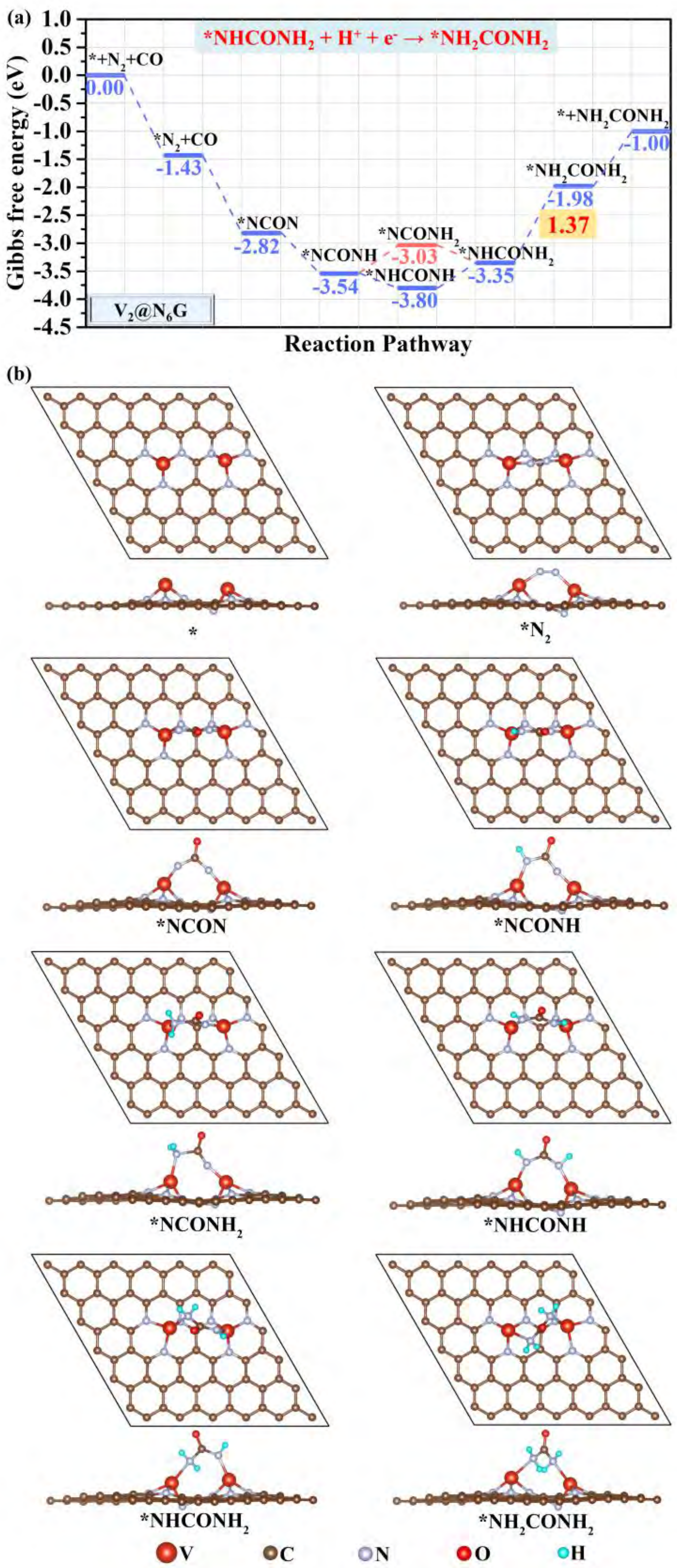

Figure S7. (a) Gibbs free energy diagram for urea production on the $V_2@N_6G$ system. (b) Optimized structures of various intermediates along the hydrogenation pathway of urea production on the $V_2@N_6G$ system.

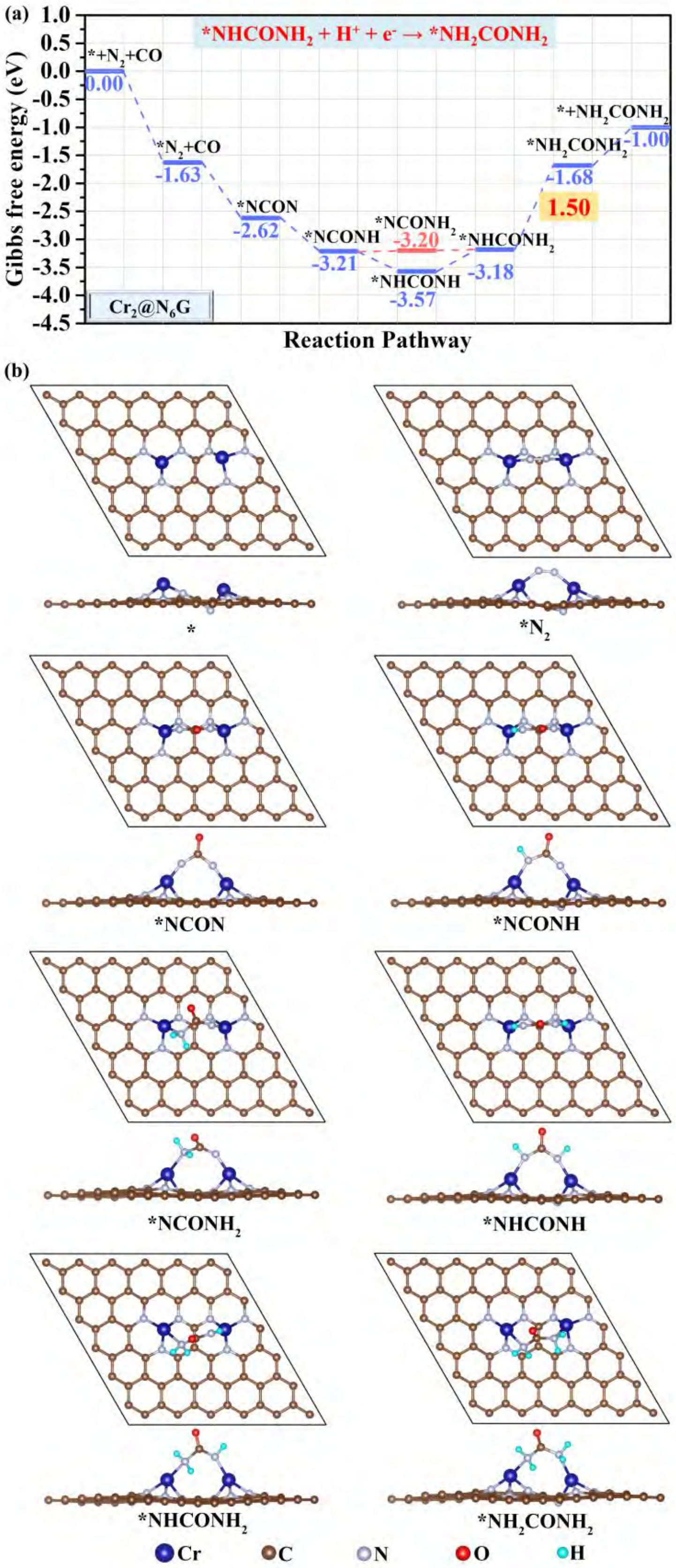

Figure S8. (a) Gibbs free energy diagram for urea production on the $Cr_2@N_6G$ system. (b) Optimized structures of various intermediates along the hydrogenation pathway of urea production on the $Cr_2@N_6G$ system.

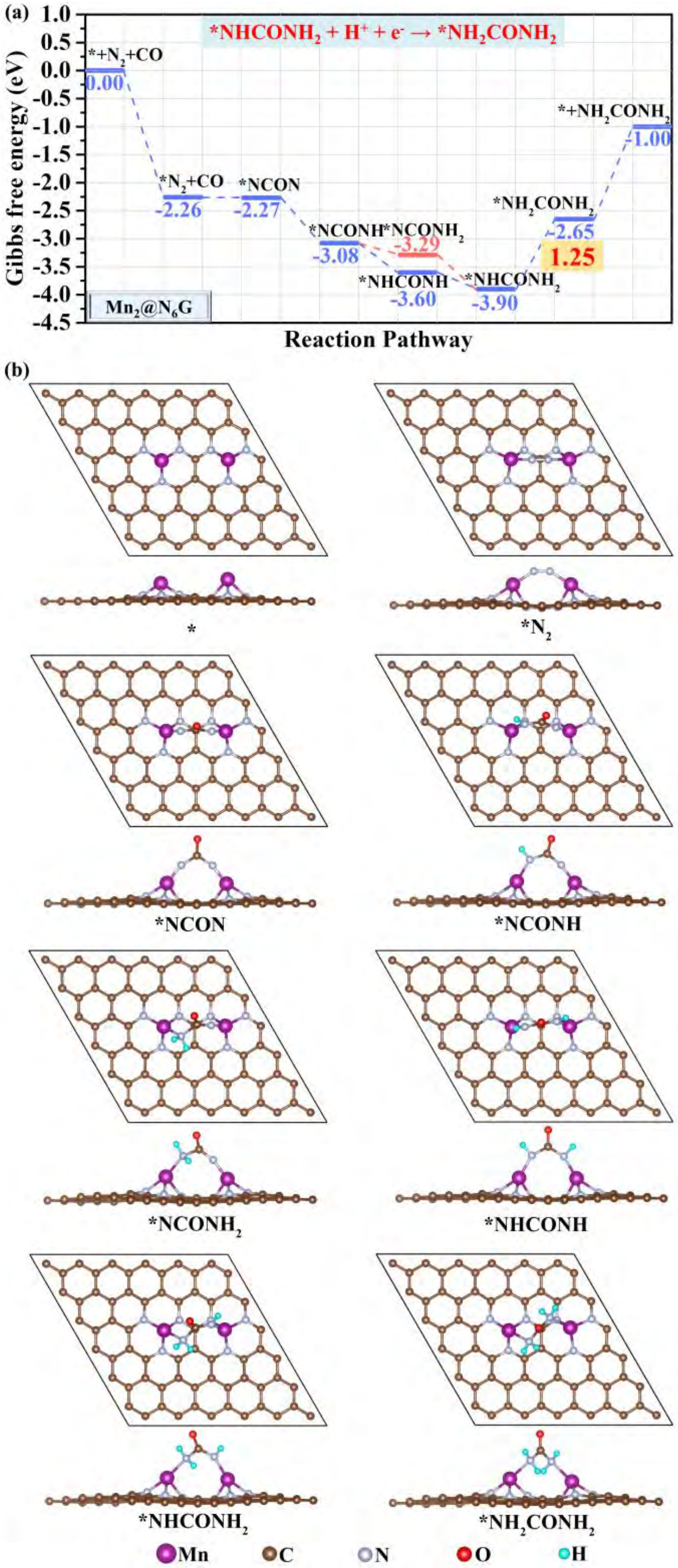

Figure S9. (a) Gibbs free energy diagram for urea production on the $Mn_2@N_6G$ system. (b) Optimized structures of various intermediates along the hydrogenation pathway of urea production on the $Mn_2@N_6G$ system.

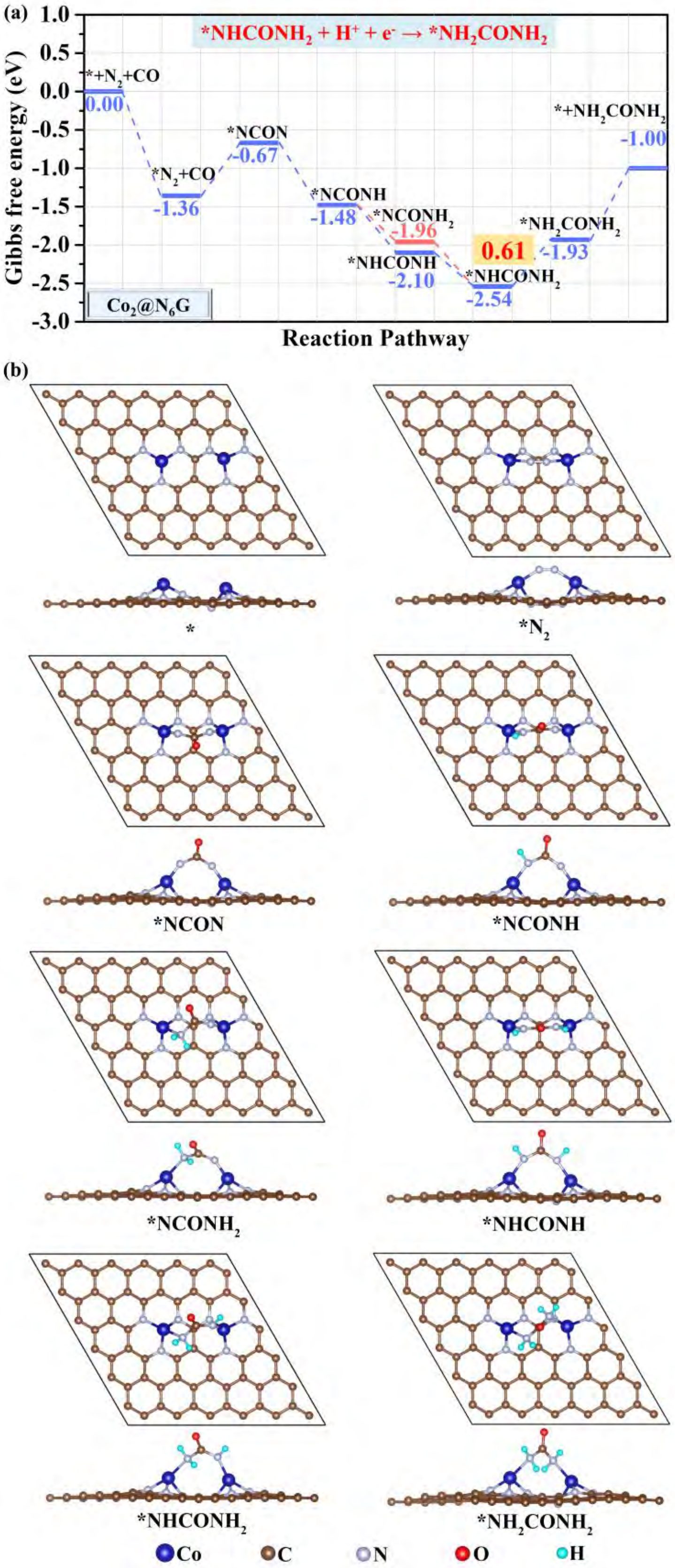

Figure S10. (a) Gibbs free energy diagram for urea production on the $Co_2@N_6G$ system. (b) Optimized structures of various intermediates along the hydrogenation pathway of urea production on the $Co_2@N_6G$ system.

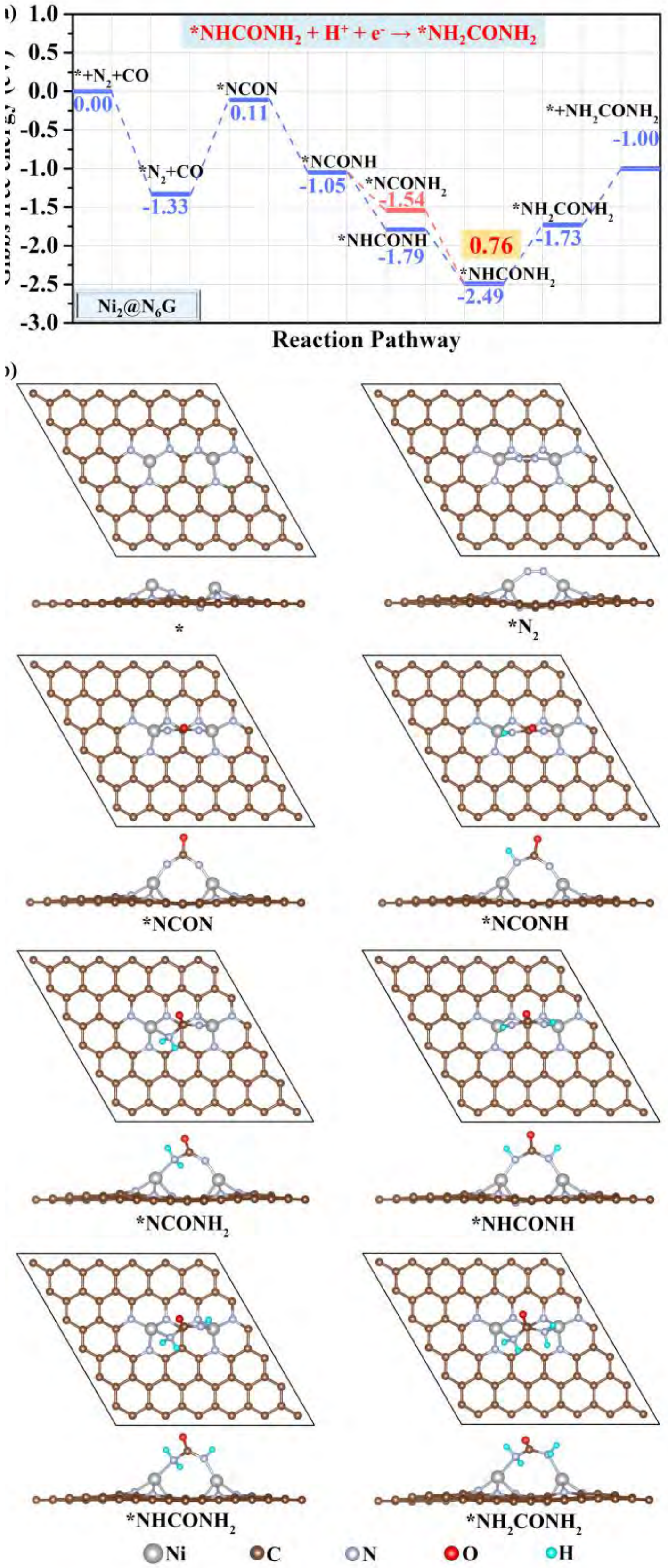

Figure S11. (a) Gibbs free energy diagram for urea production on the $Ni_2@N_6G$ system. (b) Optimized structures of various intermediates along the hydrogenation pathway of urea production on the $Ni_2@N_6G$ system.

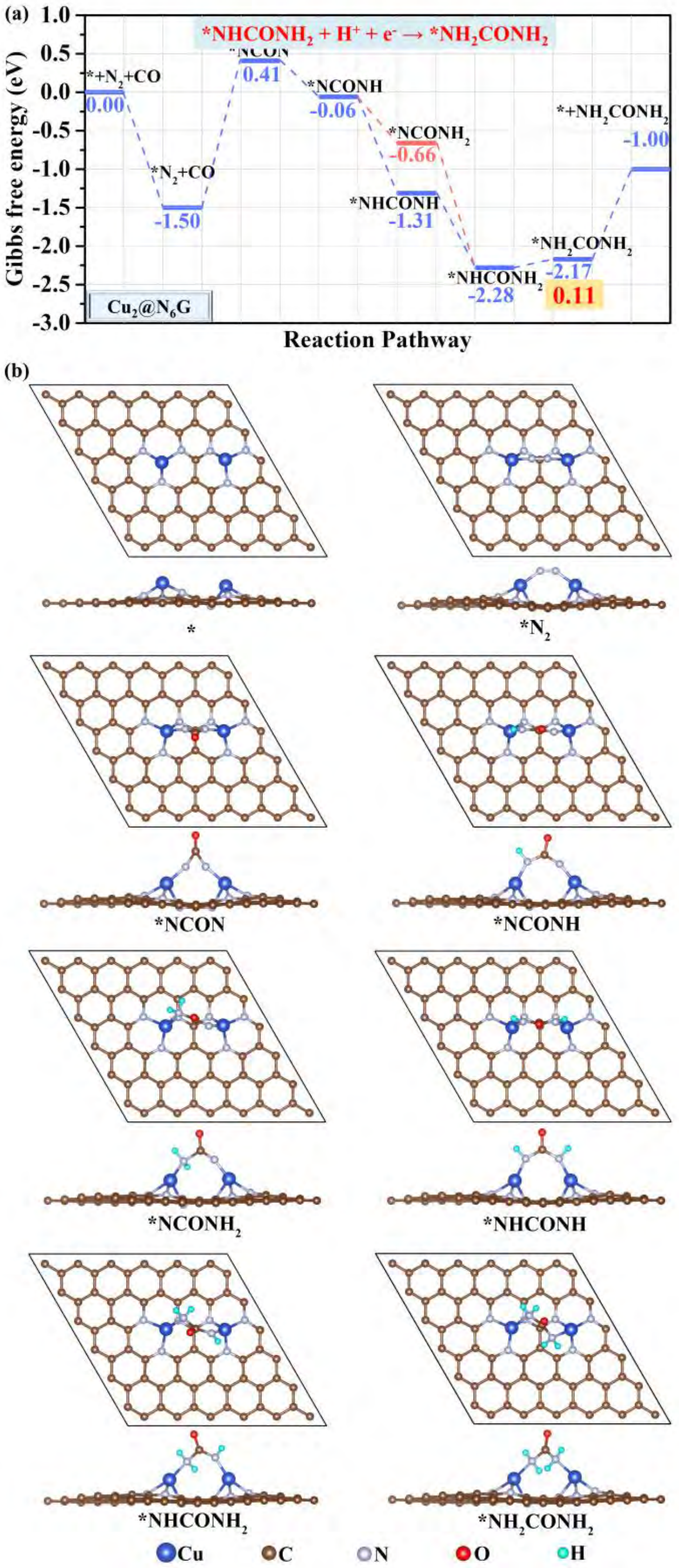

Figure S12. (a) Gibbs free energy diagram for urea production on the $Cu_2@N_6G$ system. (b) Optimized structures of various intermediates along the hydrogenation pathway of urea production on the $Cu_2@N_6G$ system.

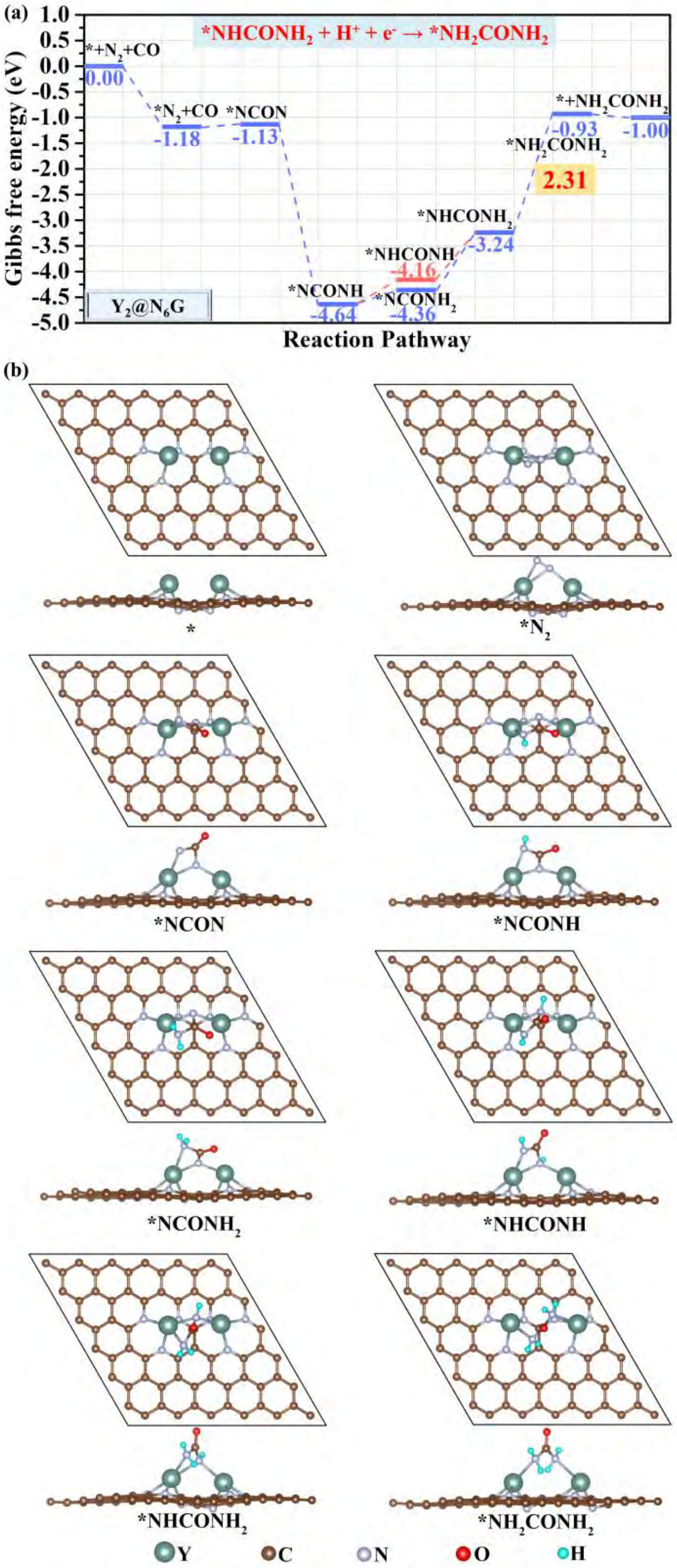

Figure S13. (a) Gibbs free energy diagram for urea production on the $Y_2@N_6G$ system. (b) Optimized structures of various intermediates along the hydrogenation pathway of urea production on the $Y_2@N_6G$ system.

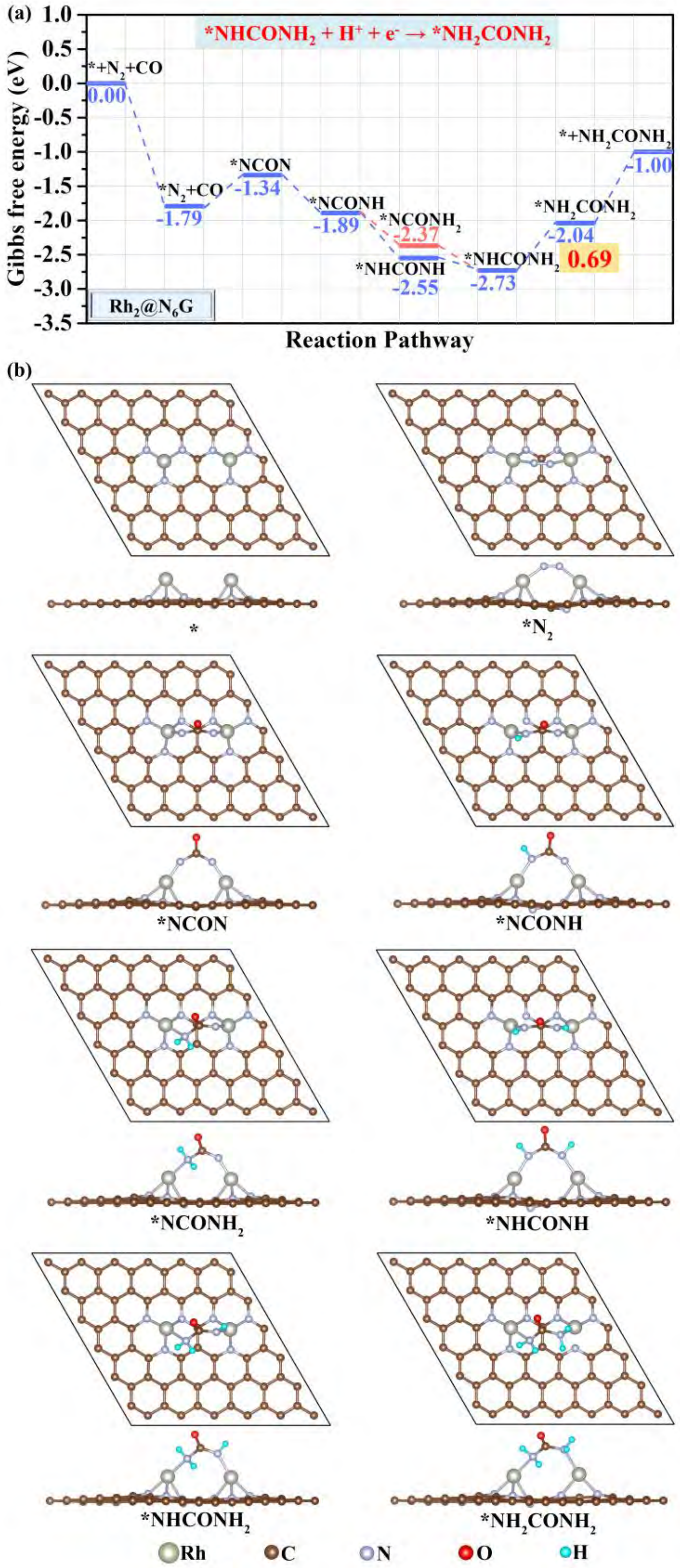

Figure S14. (a) Gibbs free energy diagram for urea production on the $Rh_2@N_6G$ system. (b) Optimized structures of various intermediates along the hydrogenation pathway of urea production on the $Rh_2@N_6G$ system.

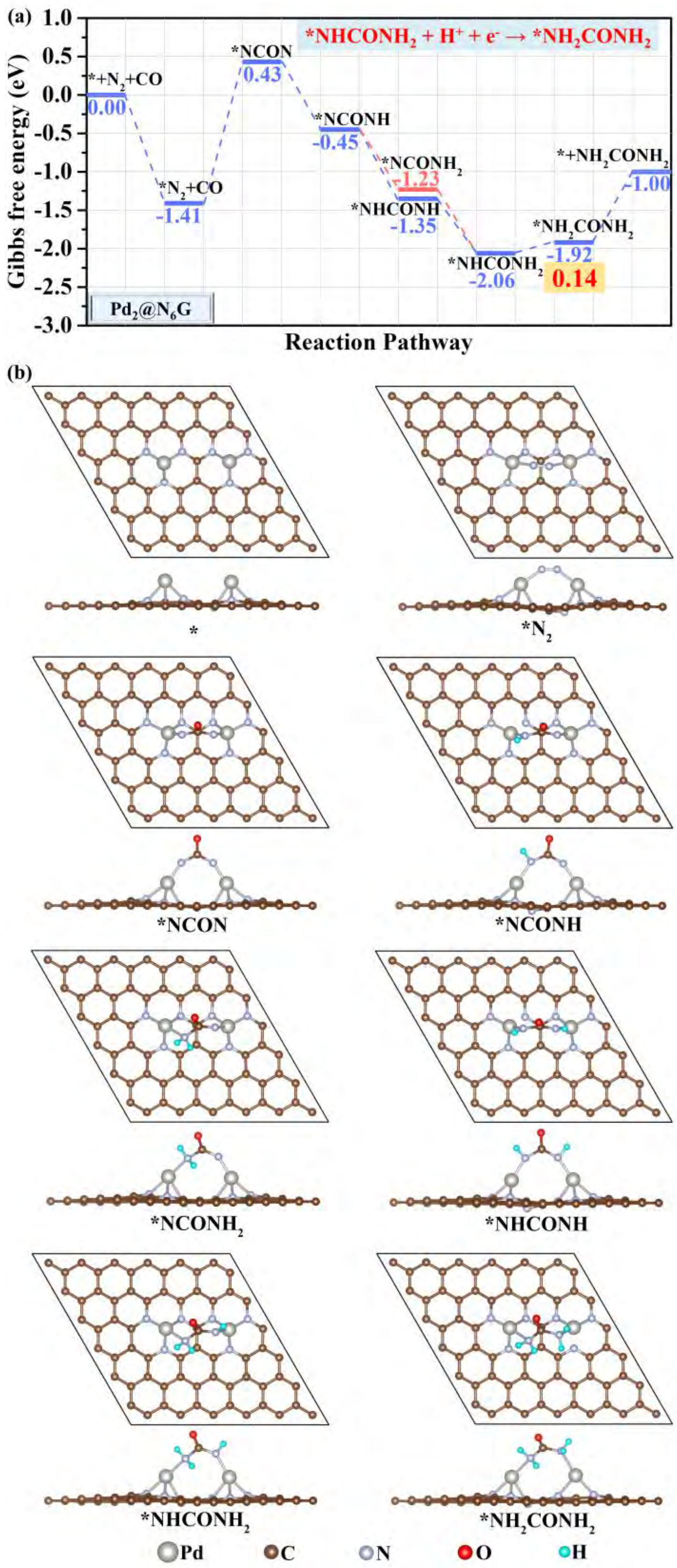

Figure S15. (a) Gibbs free energy diagram for urea production on the $Pd_2@N_6G$ system. (b) Optimized structures of various intermediates along the hydrogenation pathway of urea production on the $Pd_2@N_6G$ system.

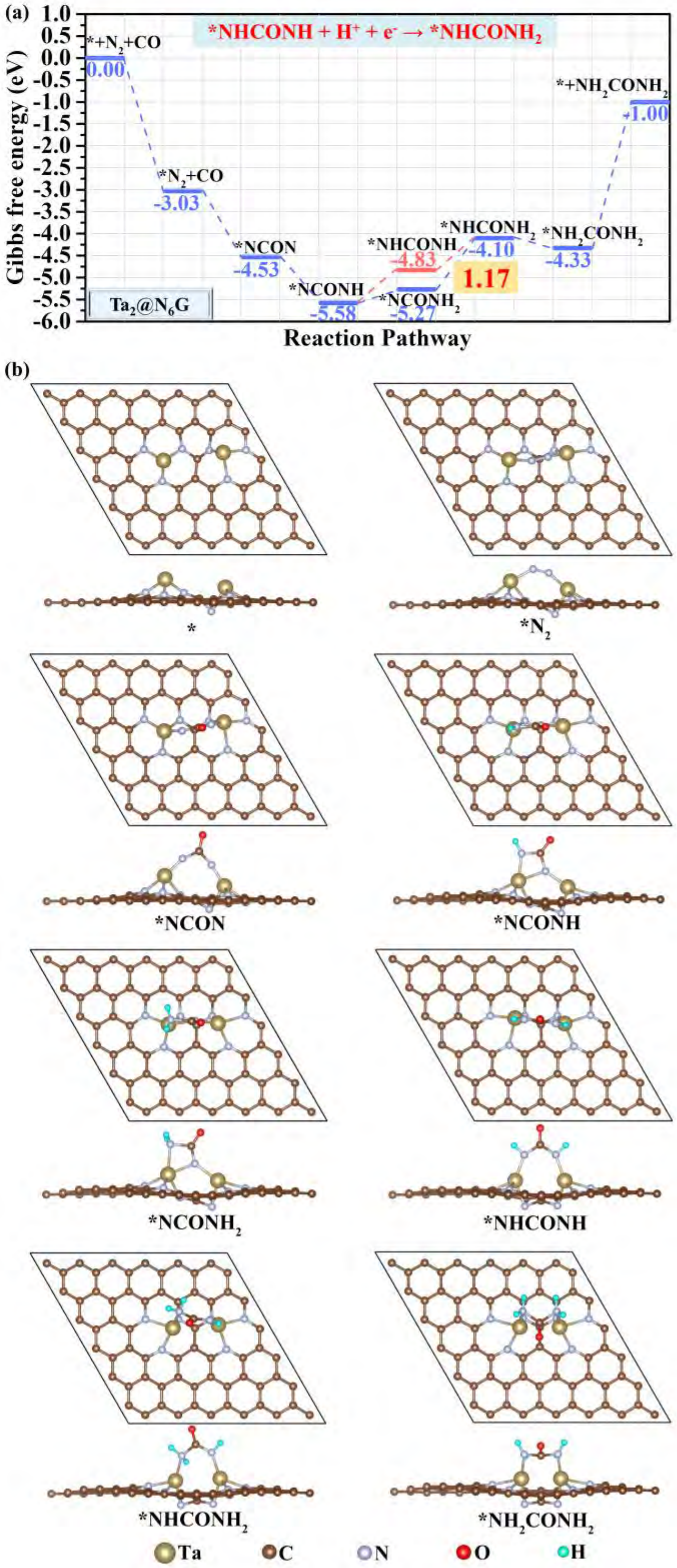

Figure S16. (a) Gibbs free energy diagram for urea production on the $Ta_2@N_6G$ system. (b) Optimized structures of various intermediates along the hydrogenation pathway of urea production on the $Ta_2@N_6G$ system.

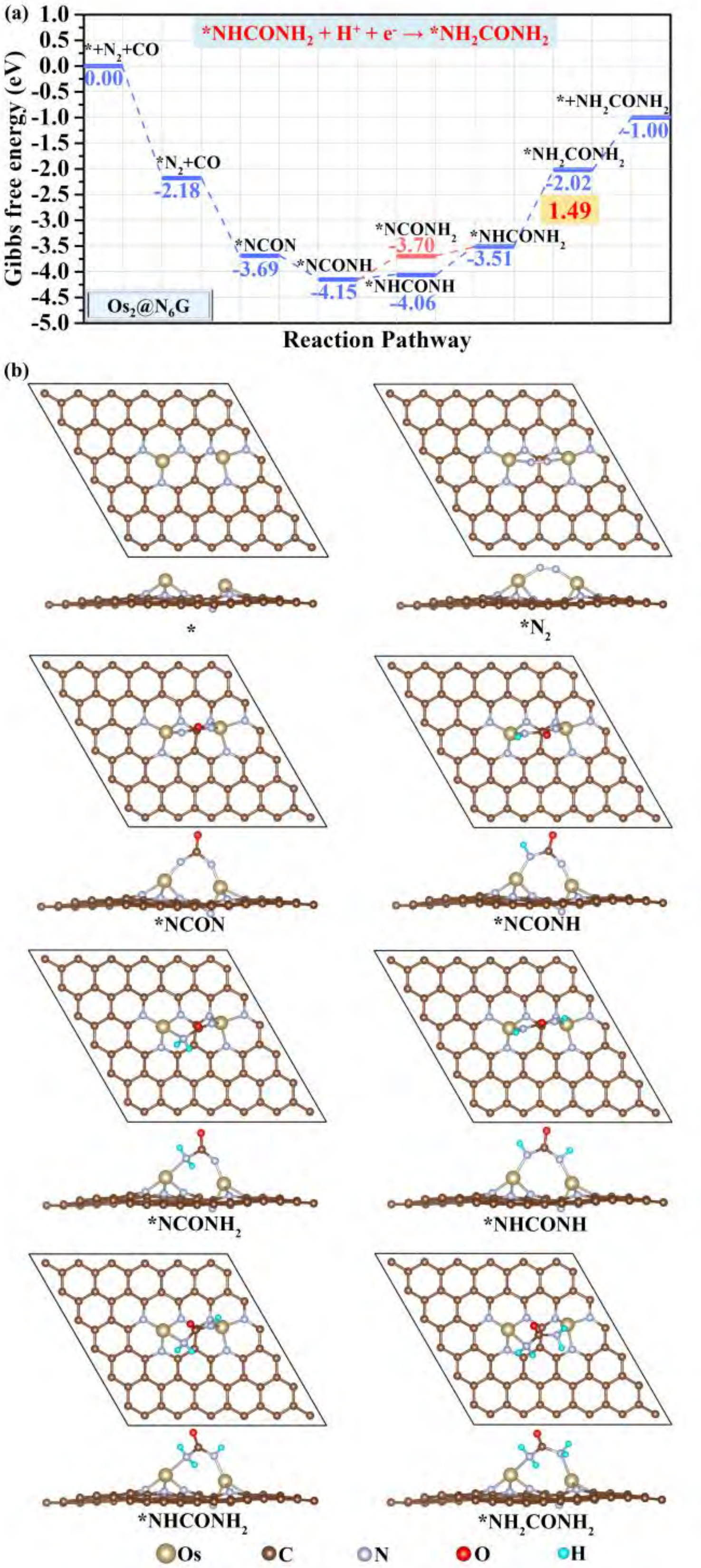

Figure S17. (a) Gibbs free energy diagram for urea production on the $Os_2@N_6G$ system. (b) Optimized structures of various intermediates along the hydrogenation pathway of urea production on the $Os_2@N_6G$ system.

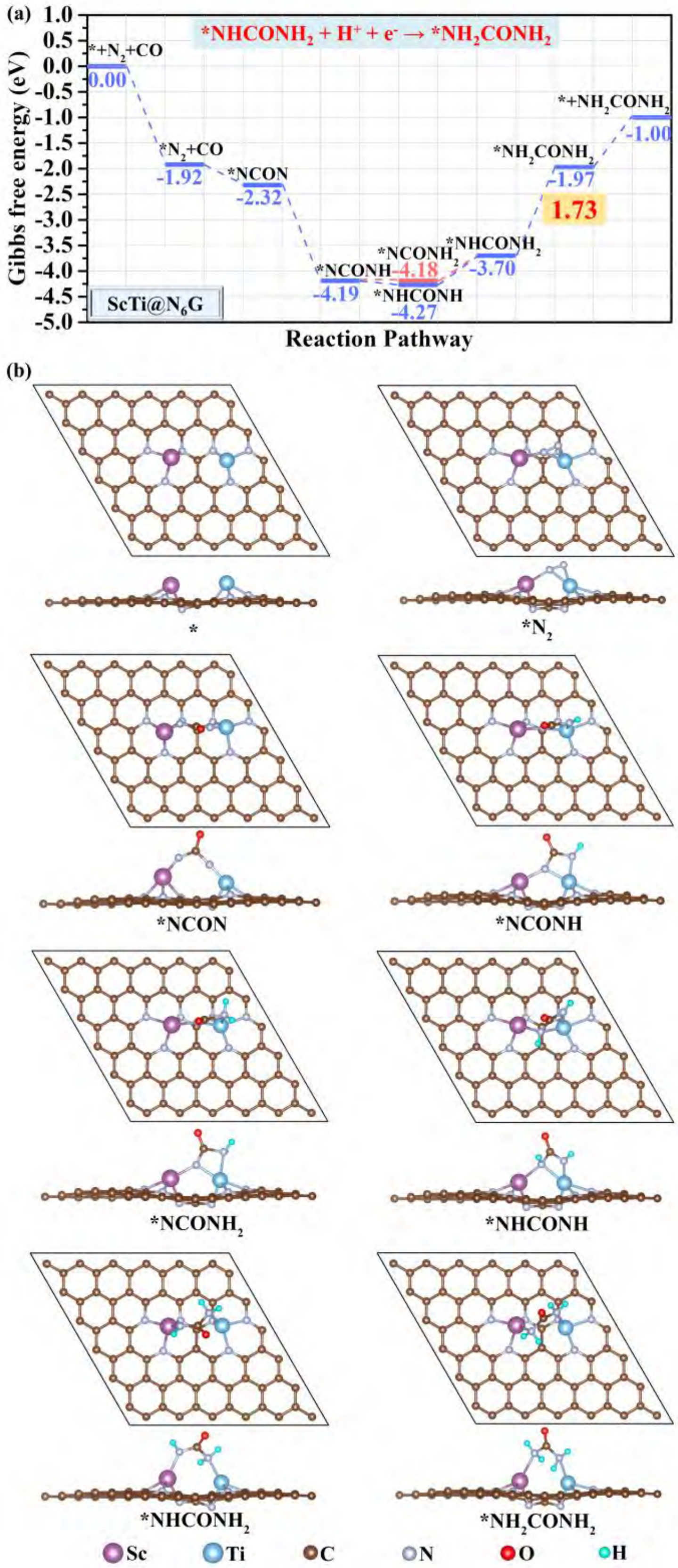

Figure S18. (a) Gibbs free energy diagram for urea production on the ScTi@N$_6$G system. (b) Optimized structures of various intermediates along the hydrogenation pathway of urea production on the ScTi@N$_6$G system.

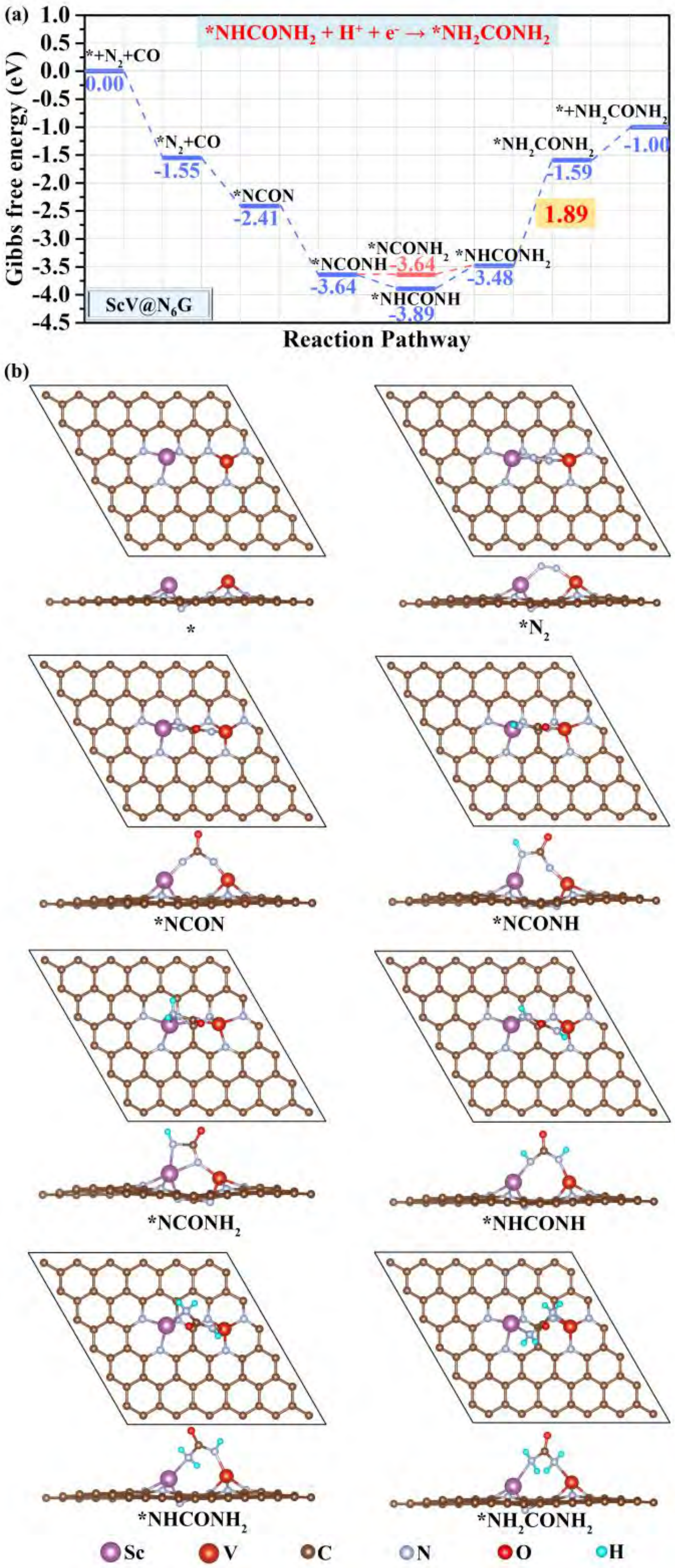

Figure S19. (a) Gibbs free energy diagram for urea production on the ScV@N$_6$G system. (b) Optimized structures of various intermediates along the hydrogenation pathway of urea production on the ScV@N$_6$G system.

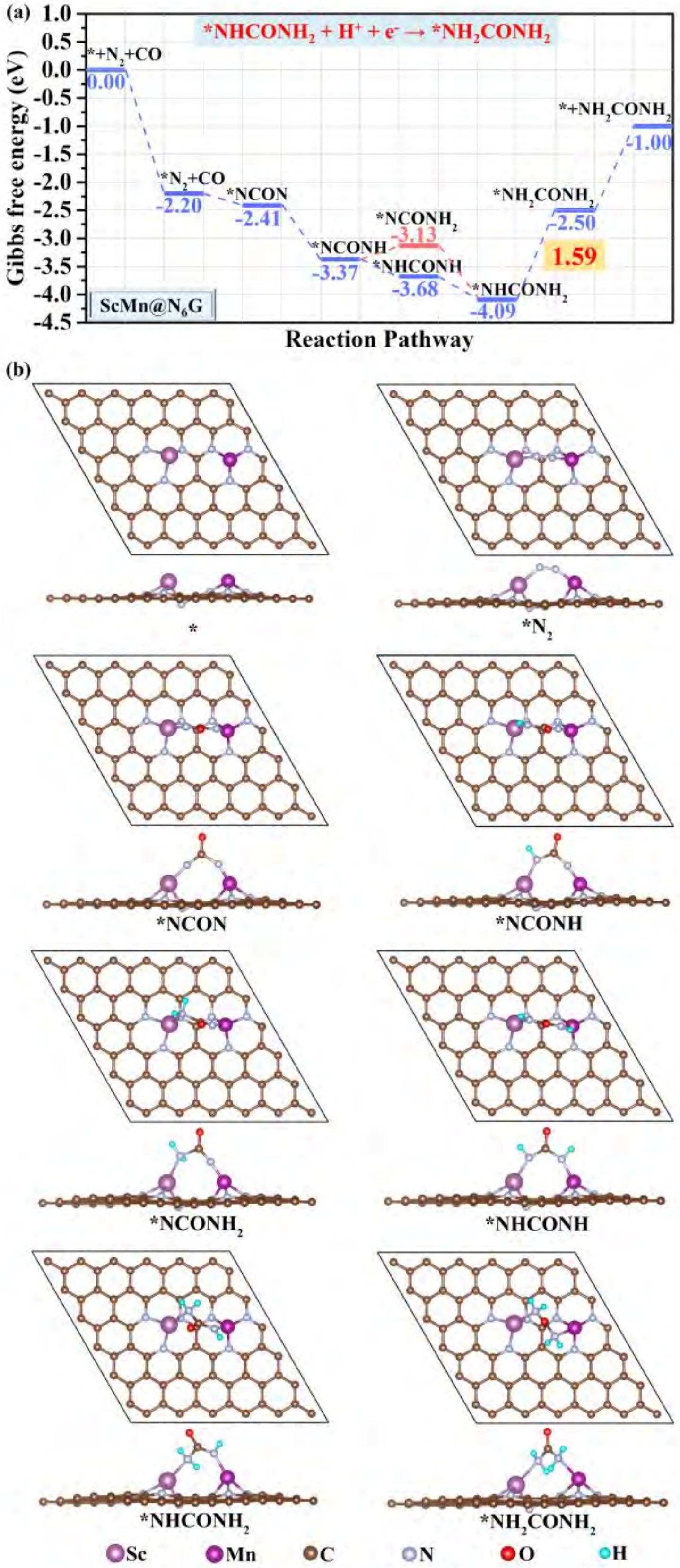

Figure S20. (a) Gibbs free energy diagram for urea production on the ScMn@N_6G system. (b) Optimized structures of various intermediates along the hydrogenation pathway of urea production on the ScMn@N_6G system.

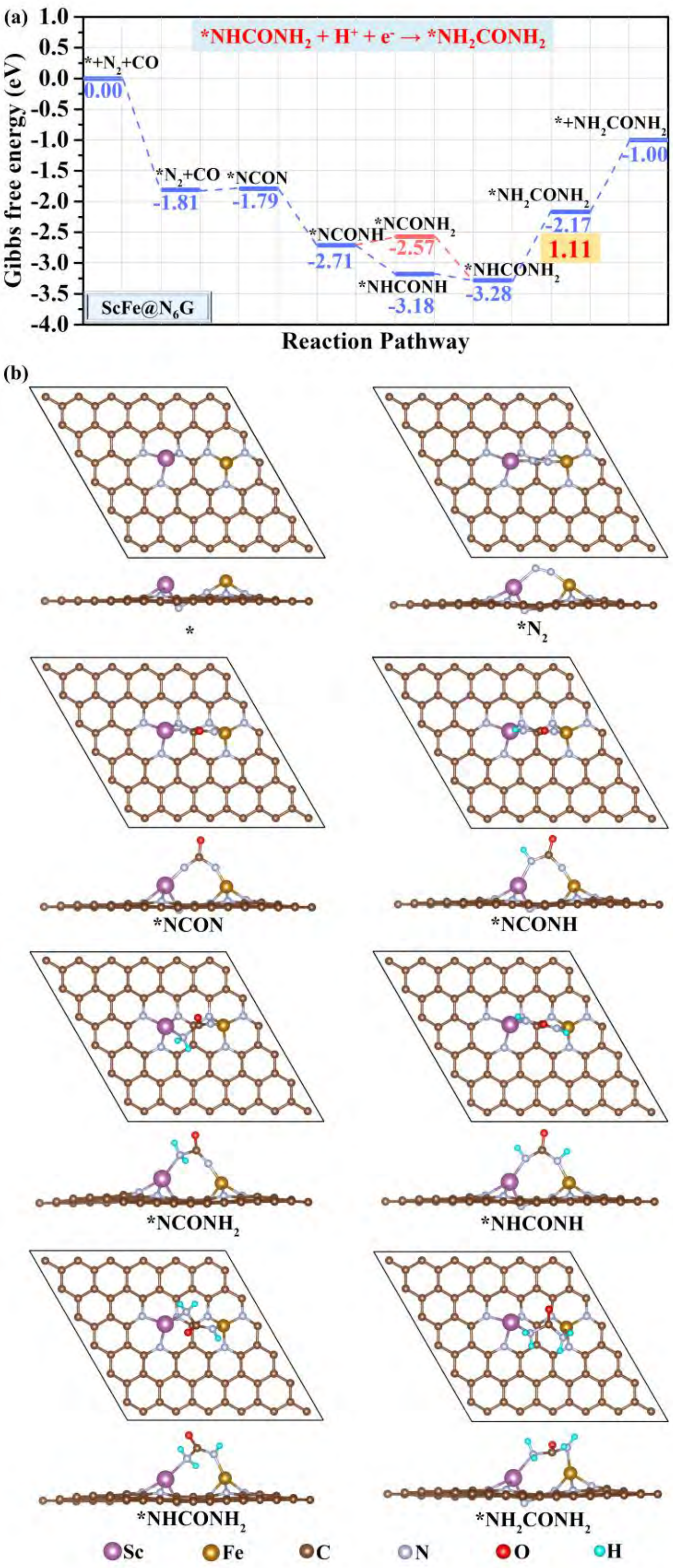

Figure S21. (a) Gibbs free energy diagram for urea production on the ScFe@N_6G system. (b) Optimized structures of various intermediates along the hydrogenation pathway of urea production on the ScFe@N_6G system.

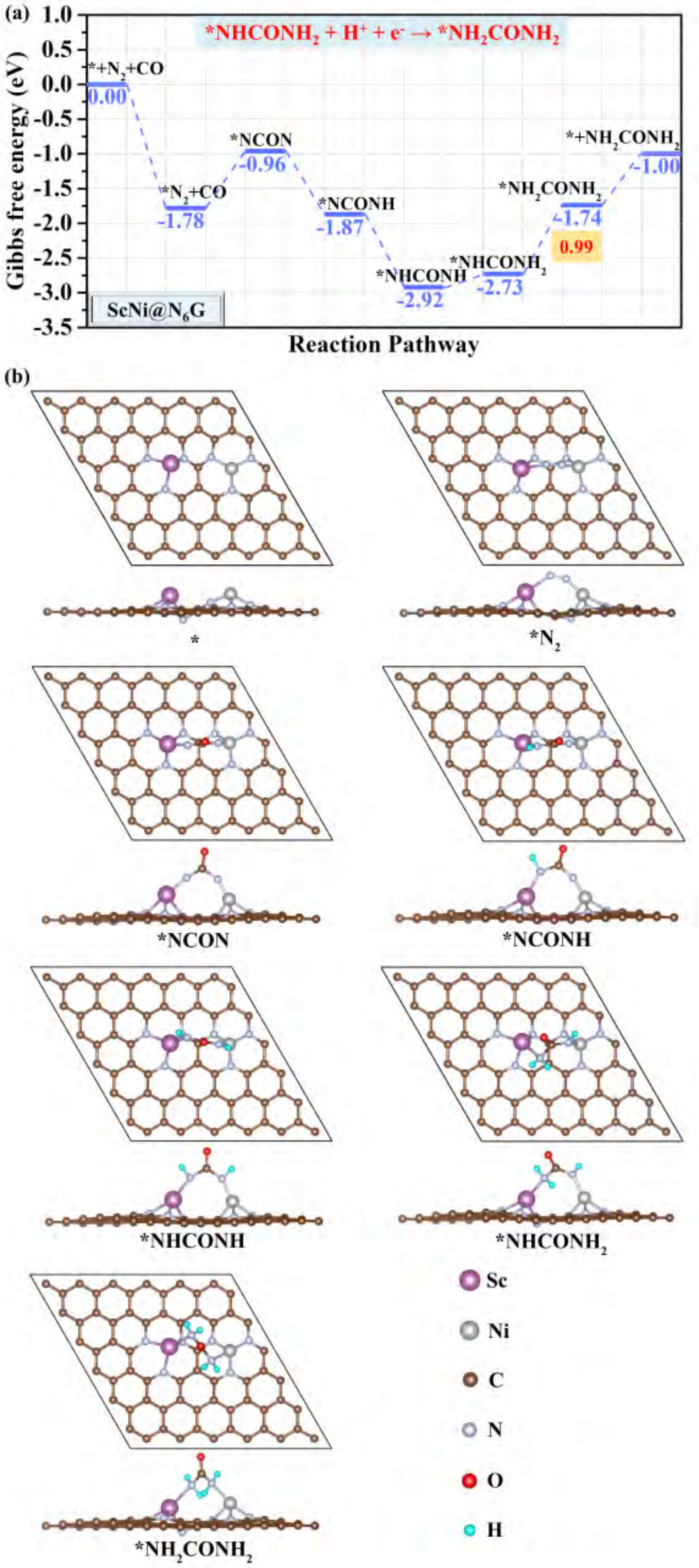

Figure S22. (a) Gibbs free energy diagram for urea production on the ScNi@N_6G system. (b) Optimized structures of various intermediates along the hydrogenation pathway of urea production on the ScNi@N_6G system.

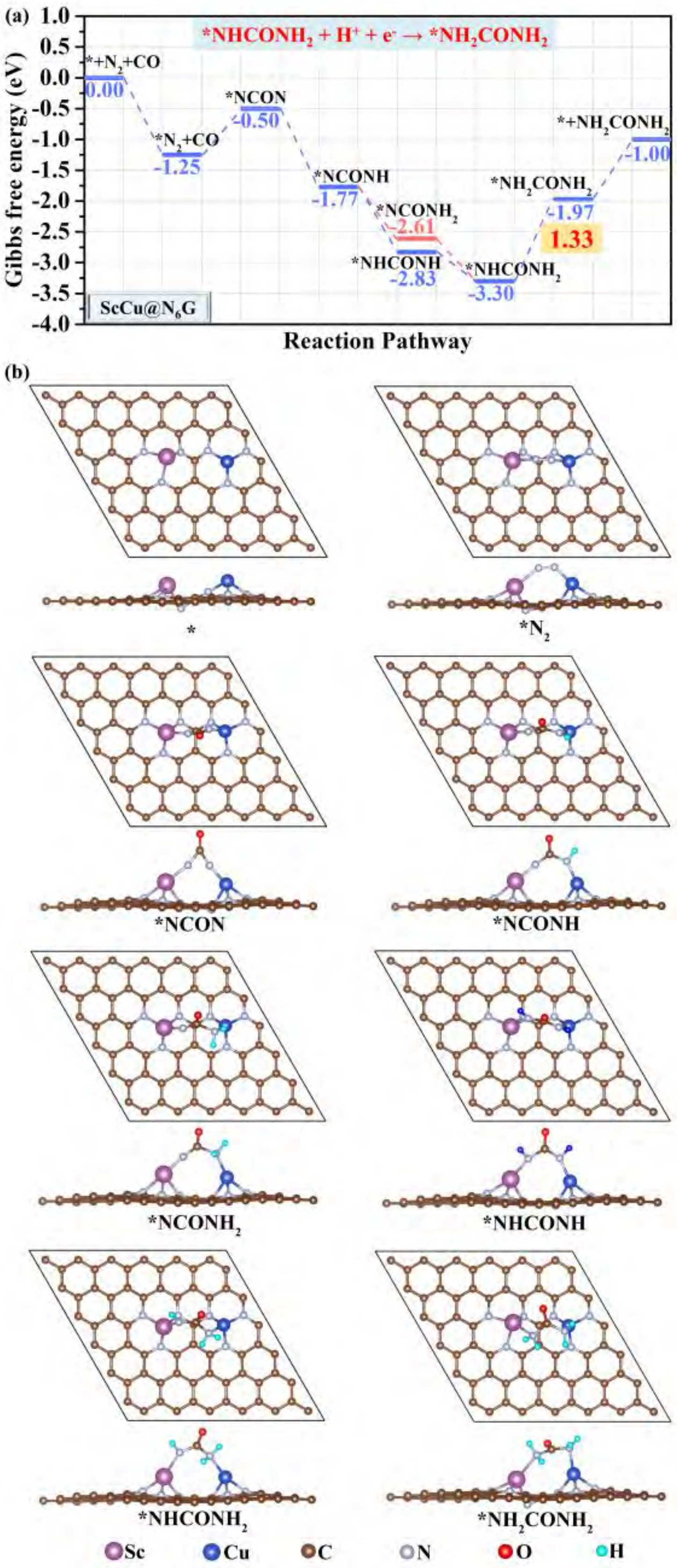

Figure S23. (a) Gibbs free energy diagram for urea production on the ScCu@N_6G system. (b) Optimized structures of various intermediates along the hydrogenation pathway of urea production on the ScCu@N_6G system.

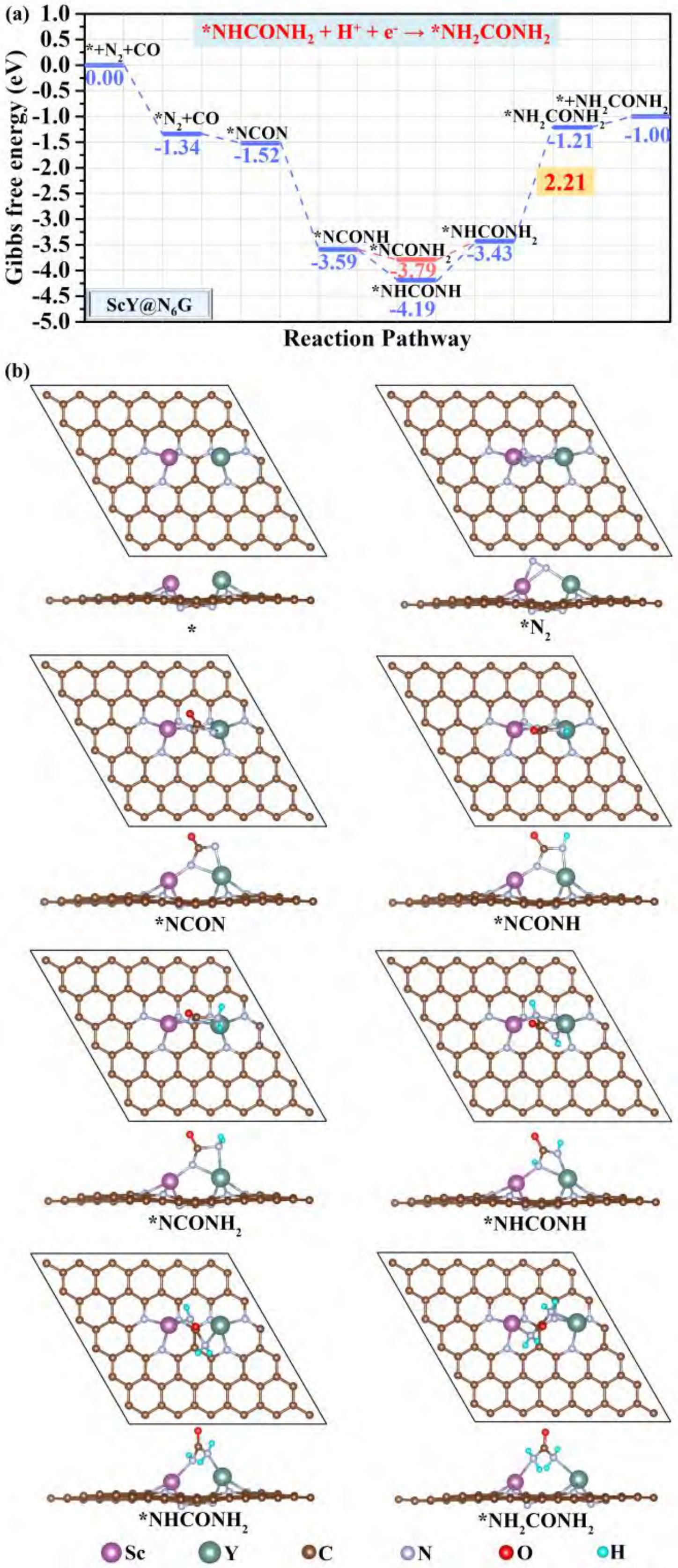

Figure S24. (a) Gibbs free energy diagram for urea production on the ScY@N_6G system. (b) Optimized structures of various intermediates along the hydrogenation pathway of urea production on the ScY@N_6G system.

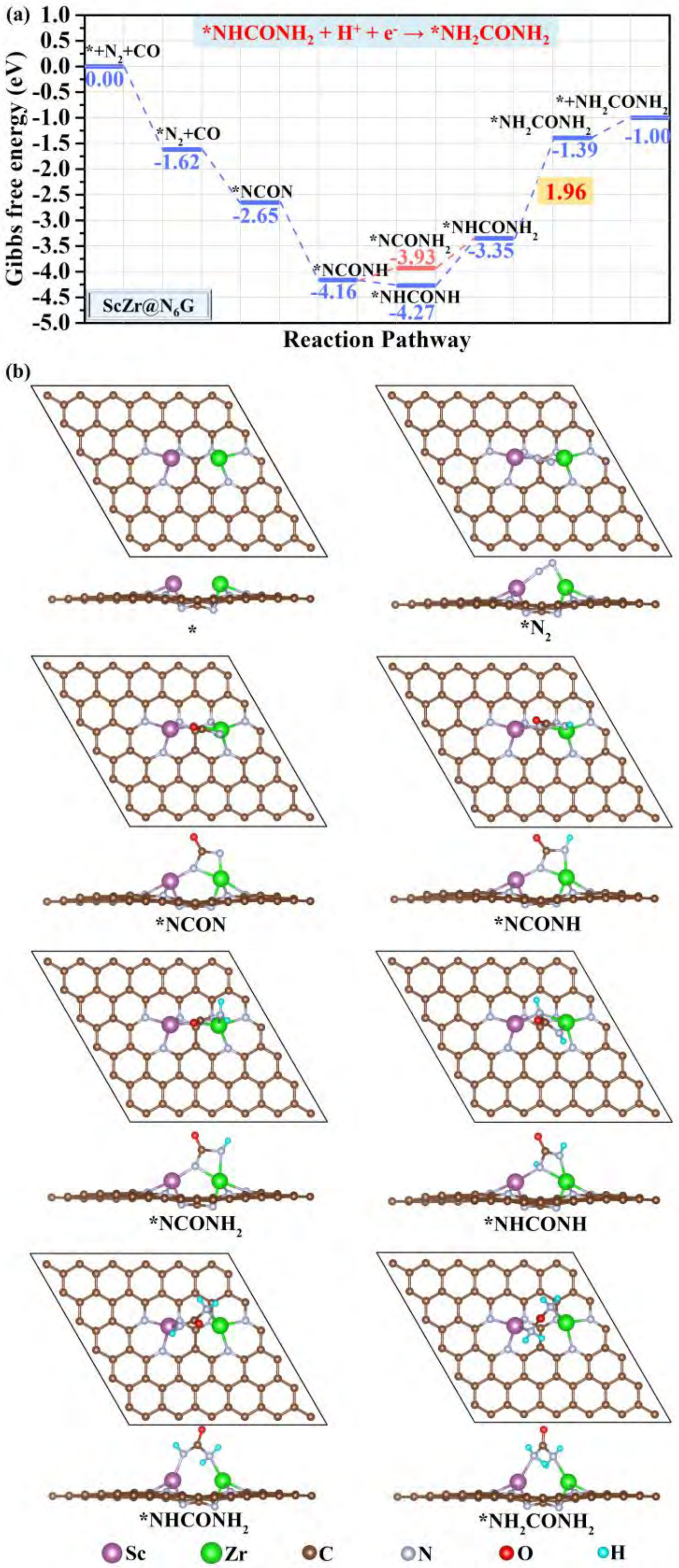

Figure S25. (a) Gibbs free energy diagram for urea production on the ScZr@N_6G system. (b) Optimized structures of various intermediates along the hydrogenation pathway of urea production on the ScZr@N_6G system.

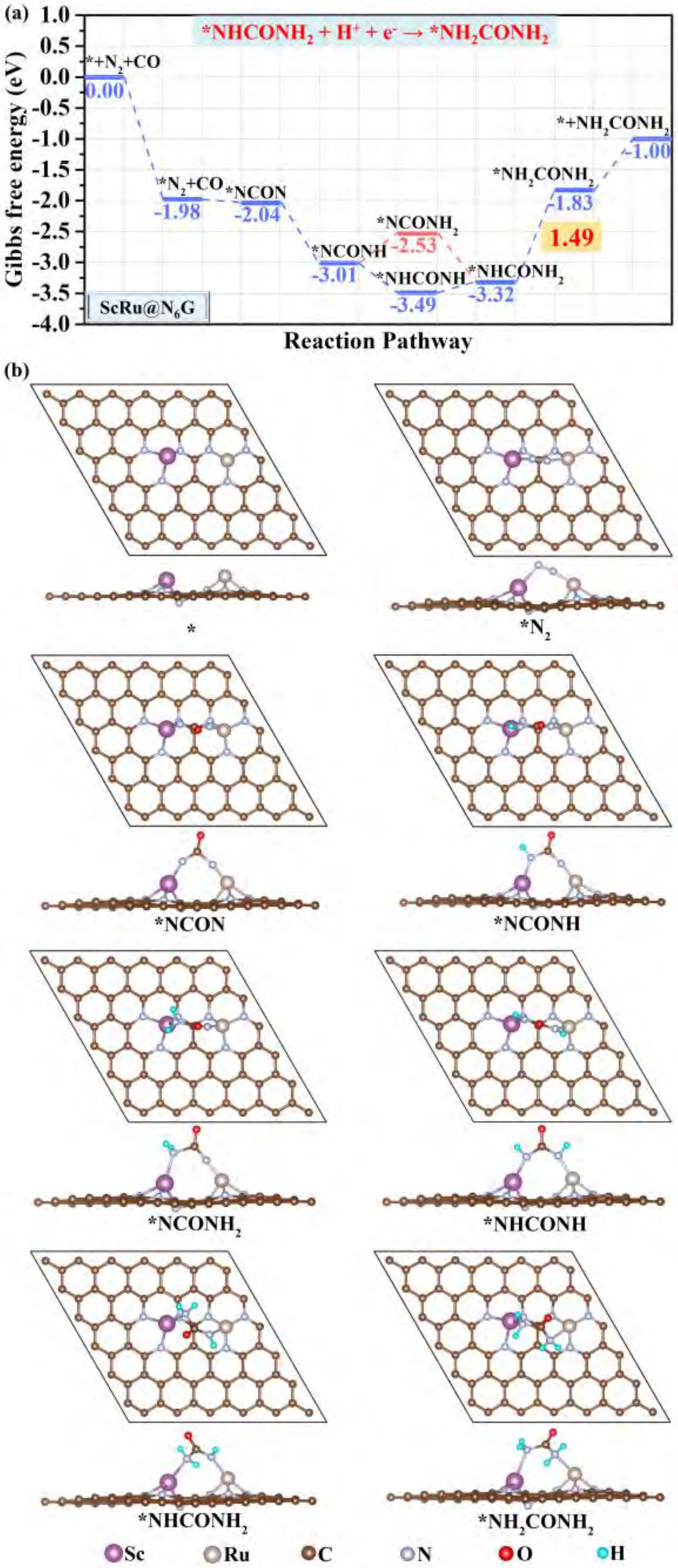

Figure S26. (a) Gibbs free energy diagram for urea production on the ScRu@N_6G system. (b) Optimized structures of various intermediates along the hydrogenation pathway of urea production on the ScRu@N_6G system.

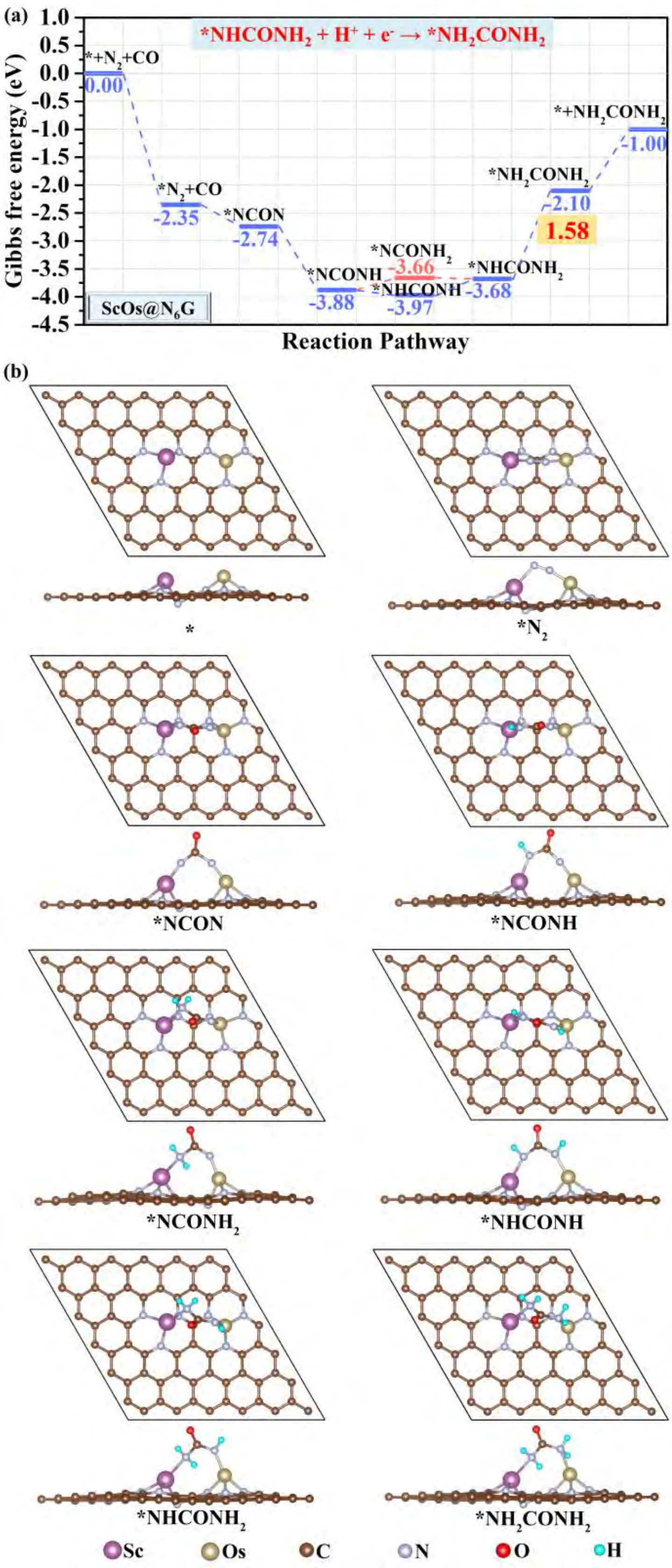

Figure S27. (a) Gibbs free energy diagram for urea production on the ScOs@N_6G system. (b) Optimized structures of various intermediates along the hydrogenation pathway of urea production on the ScOs@N_6G system.

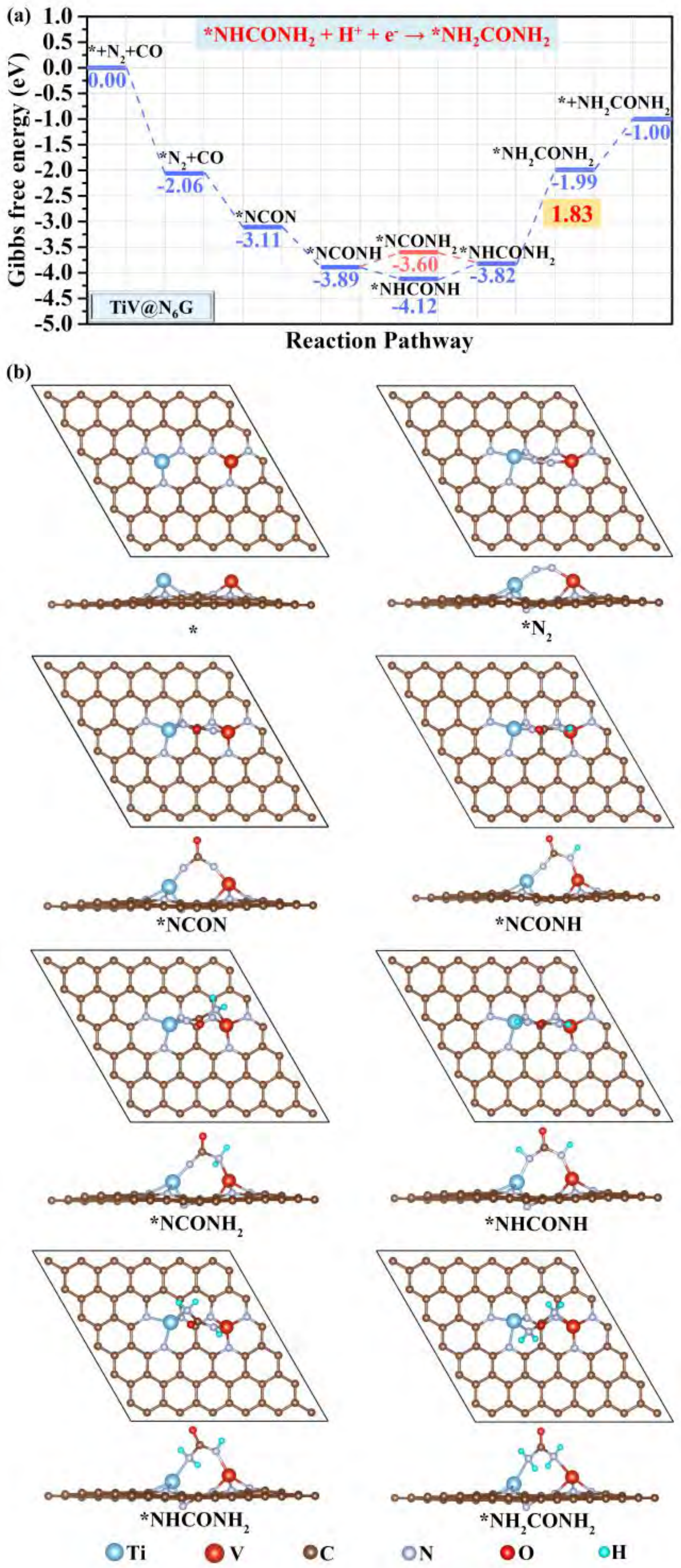

Figure S28. (a) Gibbs free energy diagram for urea production on the TiV@N_6G system. (b) Optimized structures of various intermediates along the hydrogenation pathway of urea production on the TiV@N_6G system.

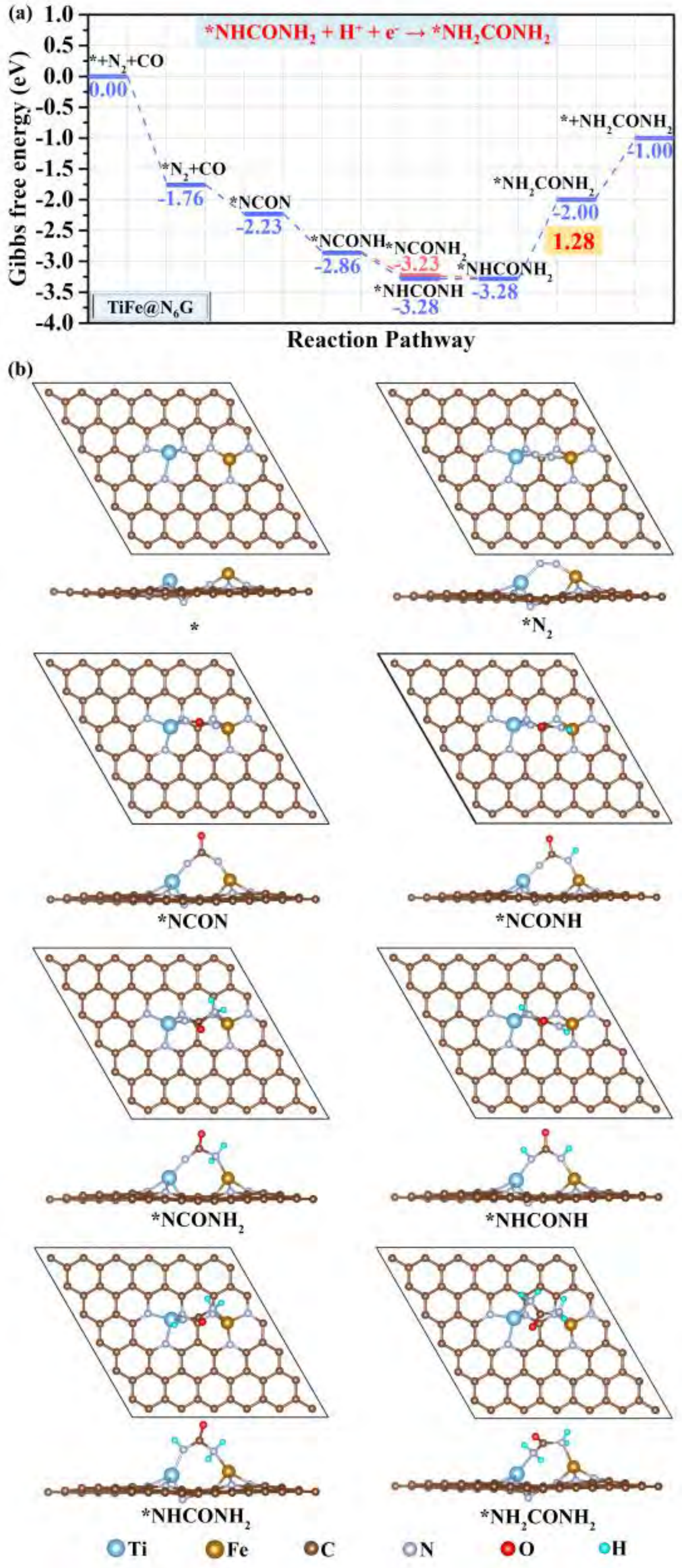

Figure S29. (a) Gibbs free energy diagram for urea production on the TiFe@N_6G system. (b) Optimized structures of various intermediates along the hydrogenation pathway of urea production on the TiFe@N_6G system.

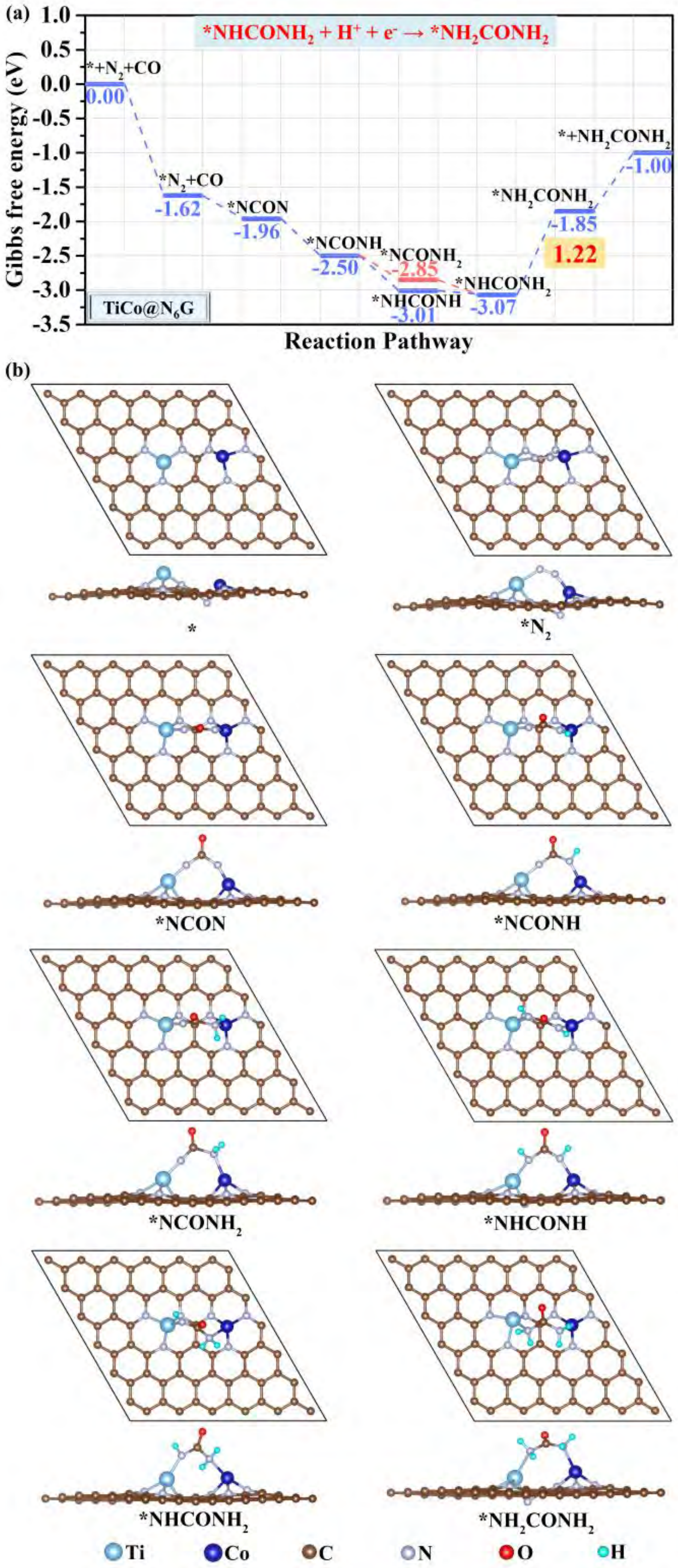

Figure S30. (a) Gibbs free energy diagram for urea production on the TiCo@N_6G system. (b) Optimized structures of various intermediates along the hydrogenation pathway of urea production on the TiCo@N_6G system.

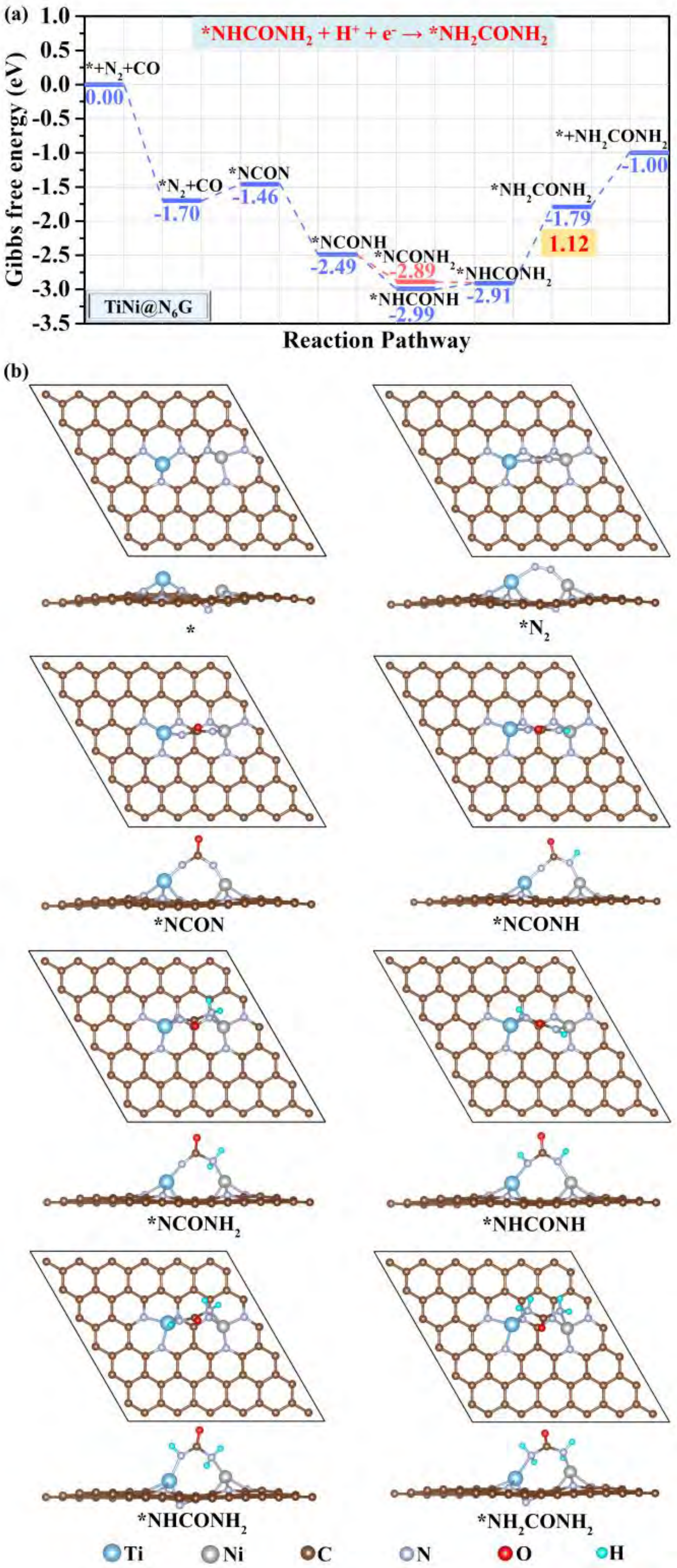

Figure S31. (a) Gibbs free energy diagram for urea production on the TiNi@N_6G system. (b) Optimized structures of various intermediates along the hydrogenation pathway of urea production on the TiNi@N_6G system.

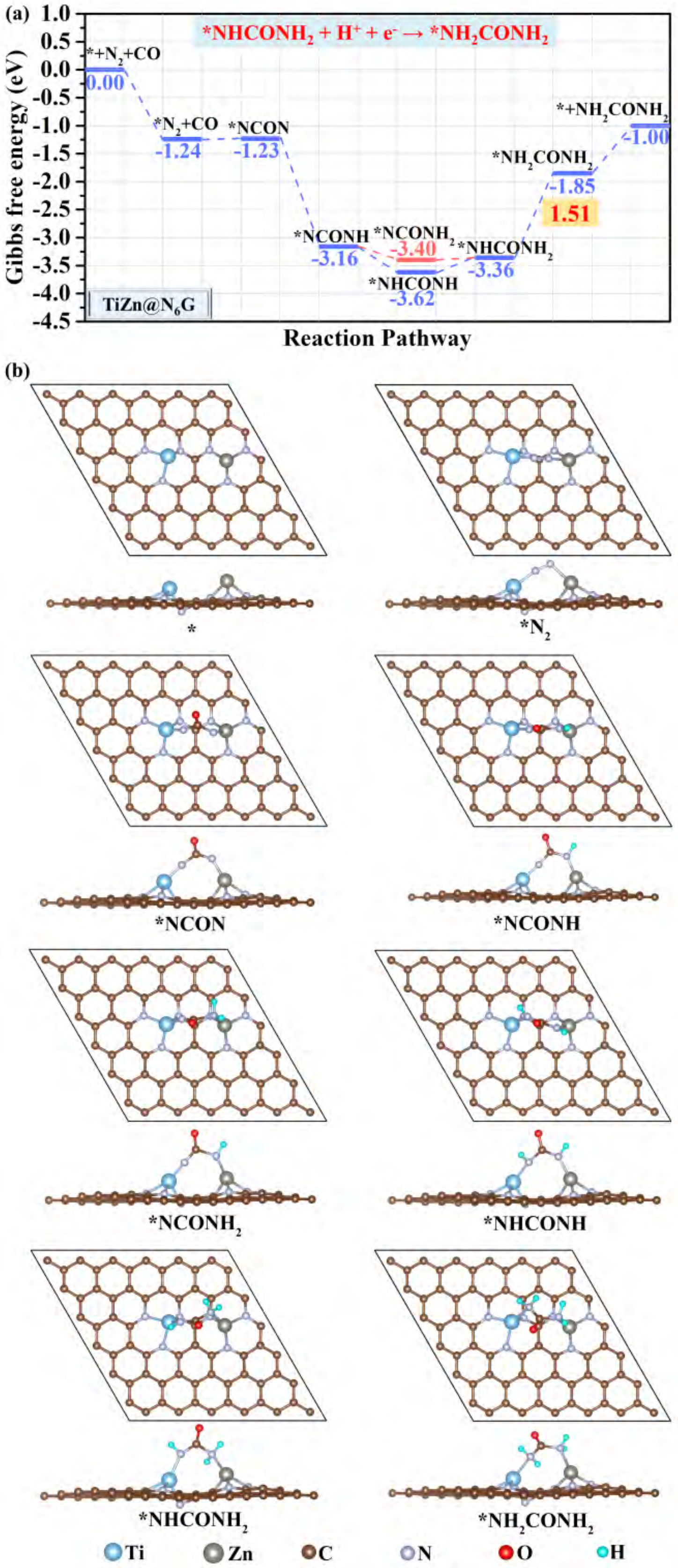

Figure S32. (a) Gibbs free energy diagram for urea production on the TiZn@N_6G system. (b) Optimized structures of various intermediates along the hydrogenation pathway of urea production on the TiZn@N_6G system.

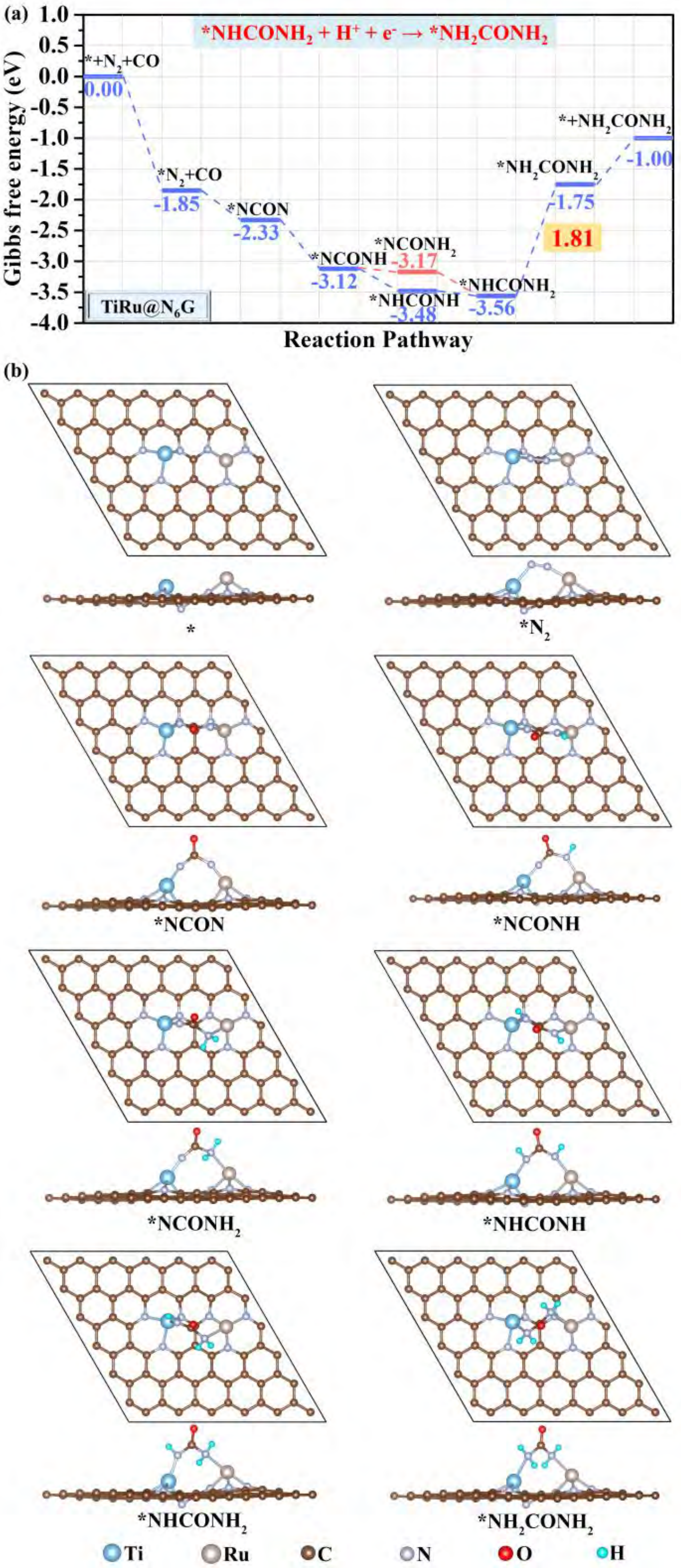

Figure S33. (a) Gibbs free energy diagram for urea production on the TiRu@N_6G system. (b) Optimized structures of various intermediates along the hydrogenation pathway of urea production on the TiRu@N_6G system.

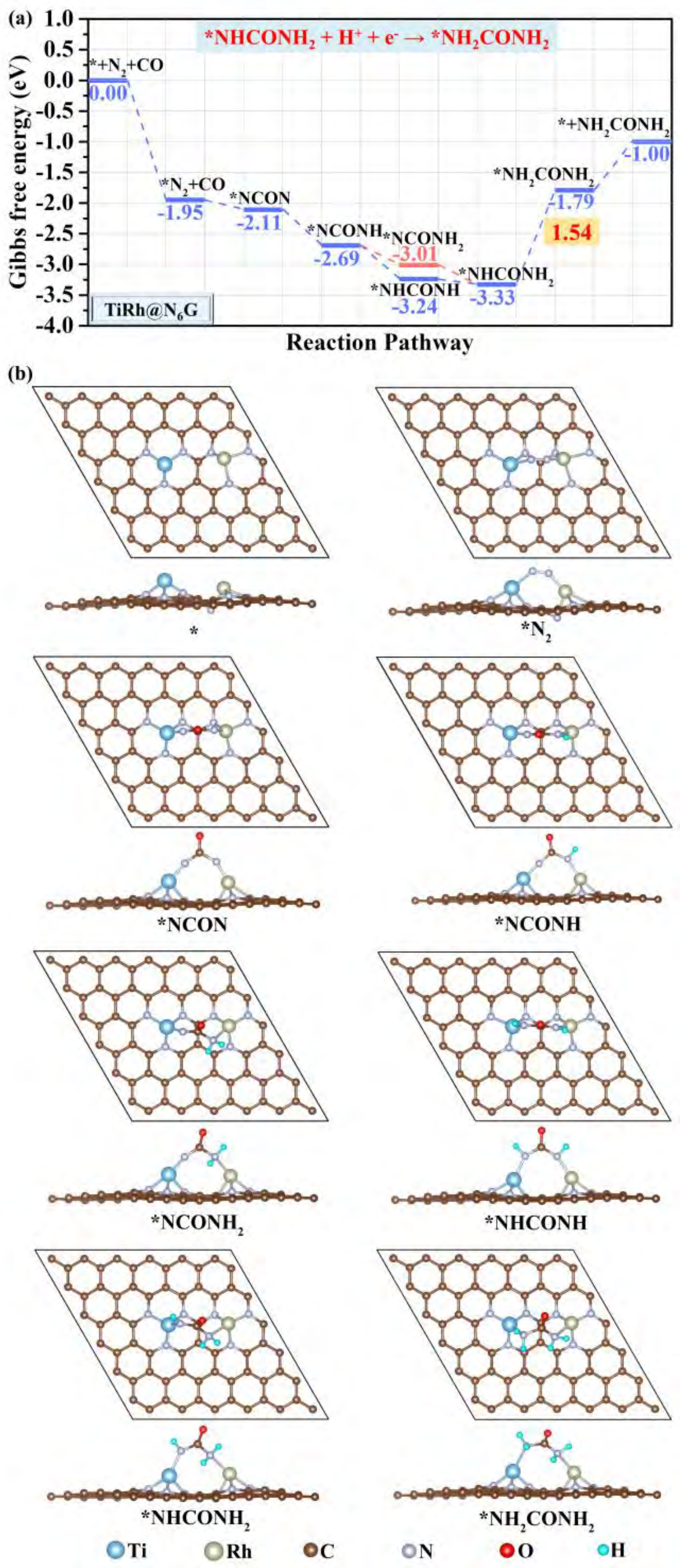

Figure S34. (a) Gibbs free energy diagram for urea production on the TiRh@N_6G system. (b) Optimized structures of various intermediates along the hydrogenation pathway of urea production on the TiRh@N_6G system.

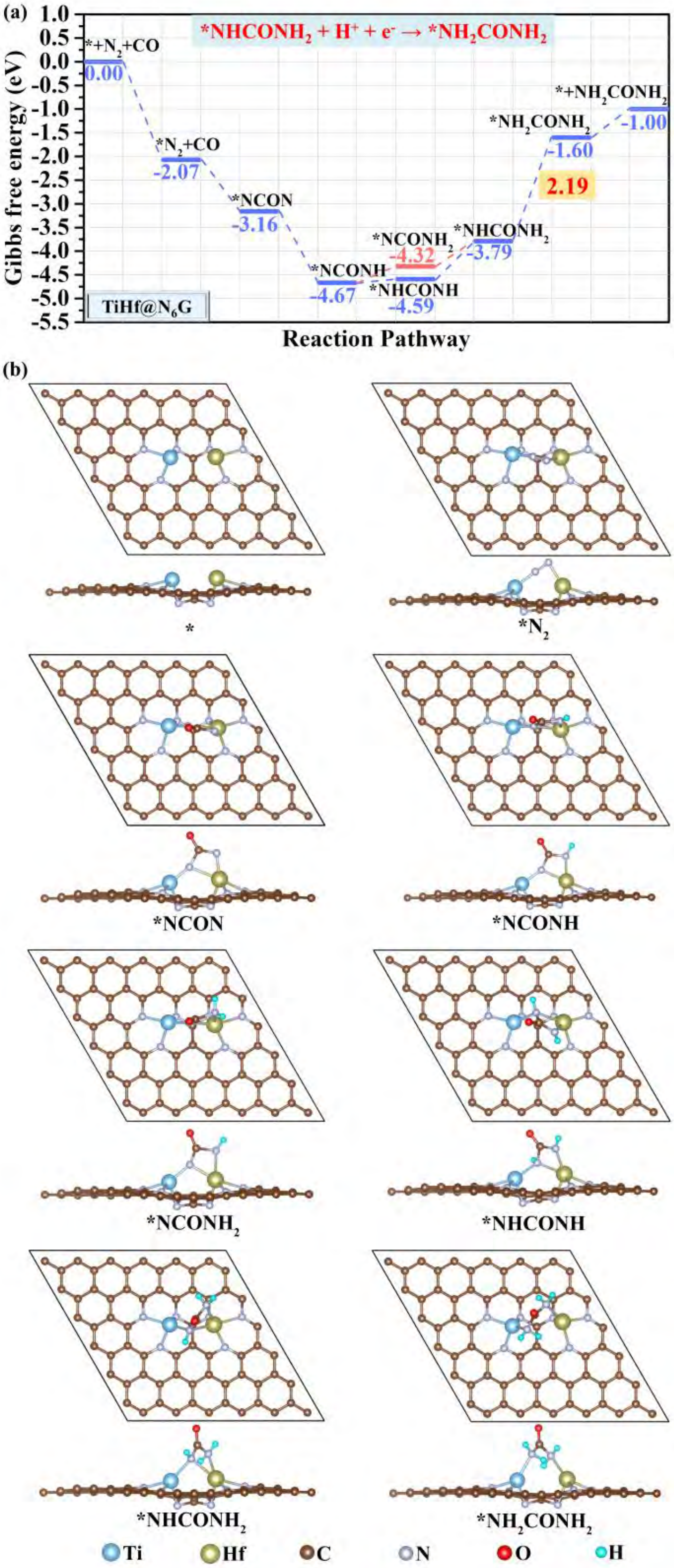

Figure S35. (a) Gibbs free energy diagram for urea production on the TiHf@N$_6$G system. (b) Optimized structures of various intermediates along the hydrogenation pathway of urea production on the TiHf@N$_6$G system.

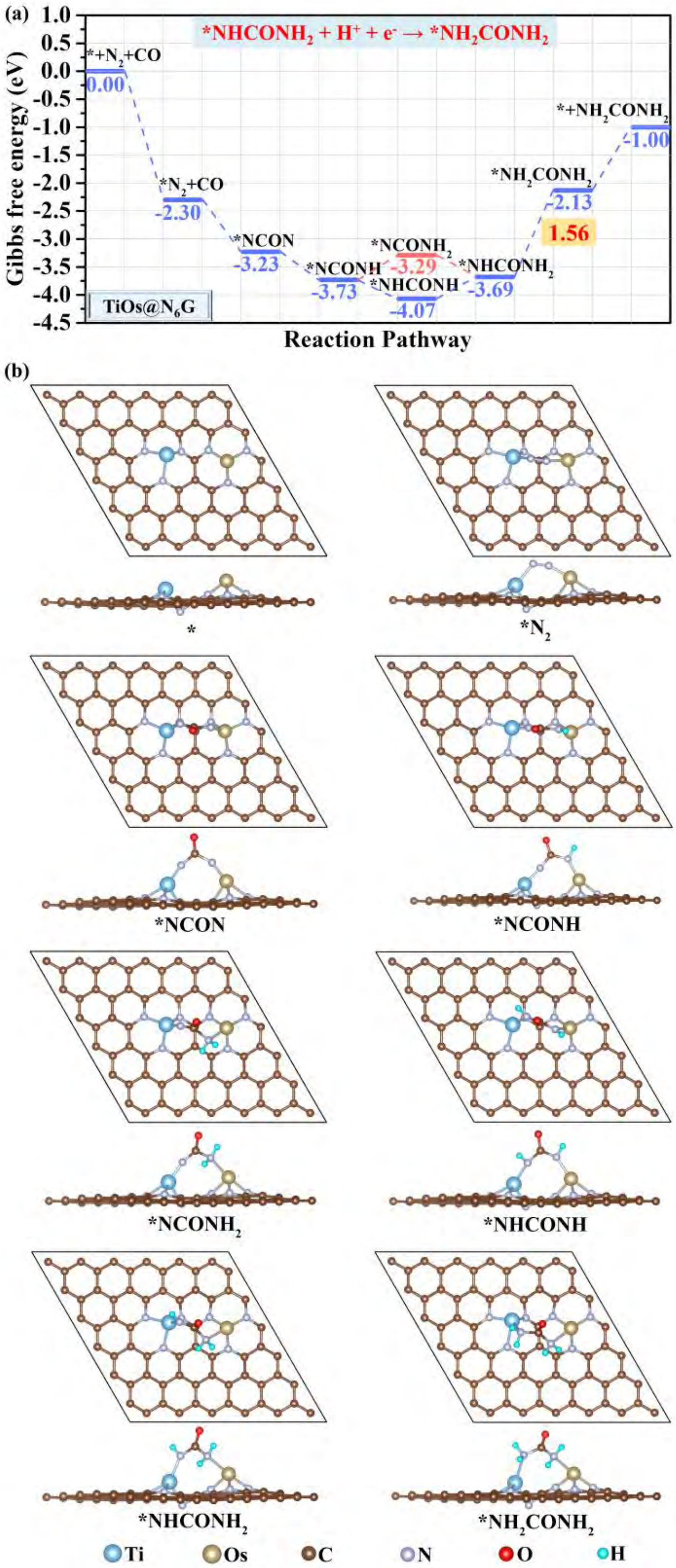

Figure S36. (a) Gibbs free energy diagram for urea production on the TiOs@N_6G system. (b) Optimized structures of various intermediates along the hydrogenation pathway of urea production on the TiOs@N_6G system.

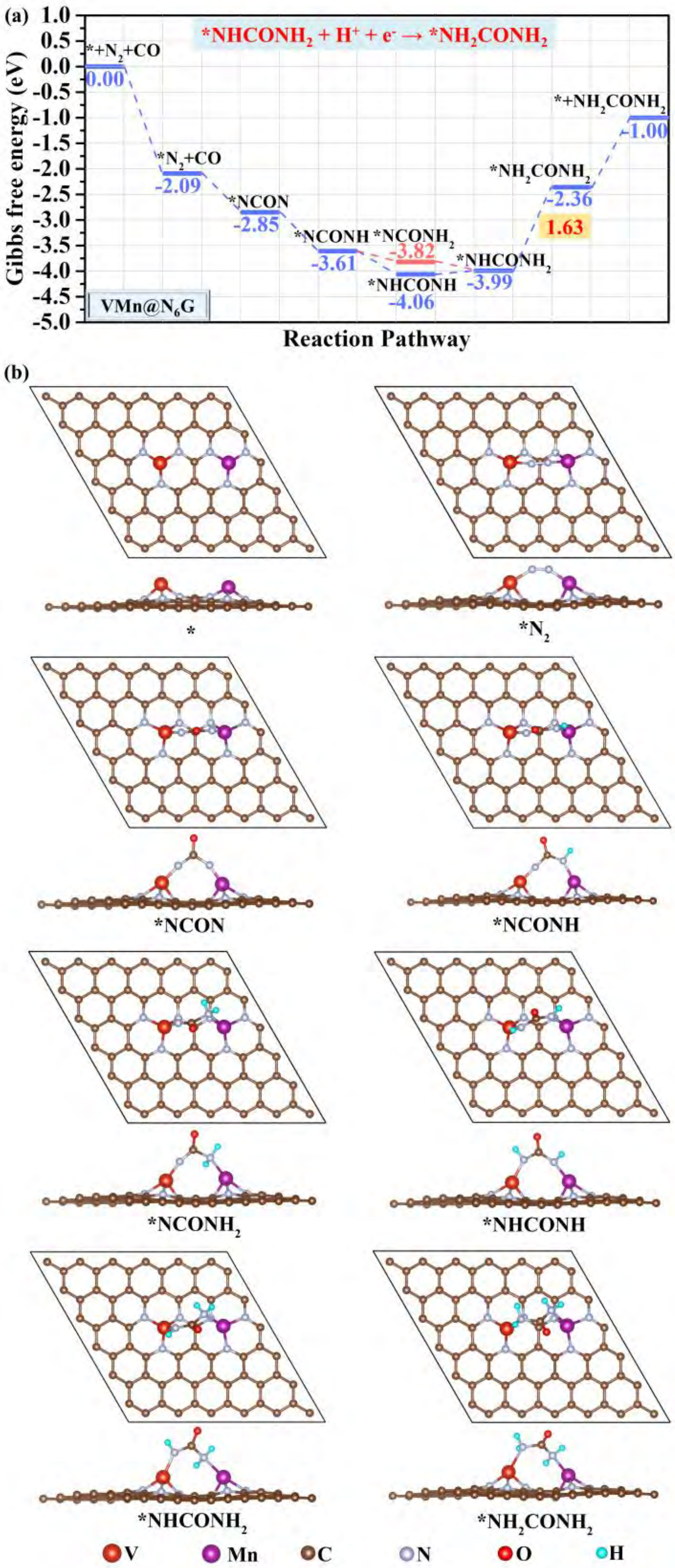

Figure S37. (a) Gibbs free energy diagram for urea production on the VMn@N_6G system. (b) Optimized structures of various intermediates along the hydrogenation pathway of urea production on the VMn@N_6G system.

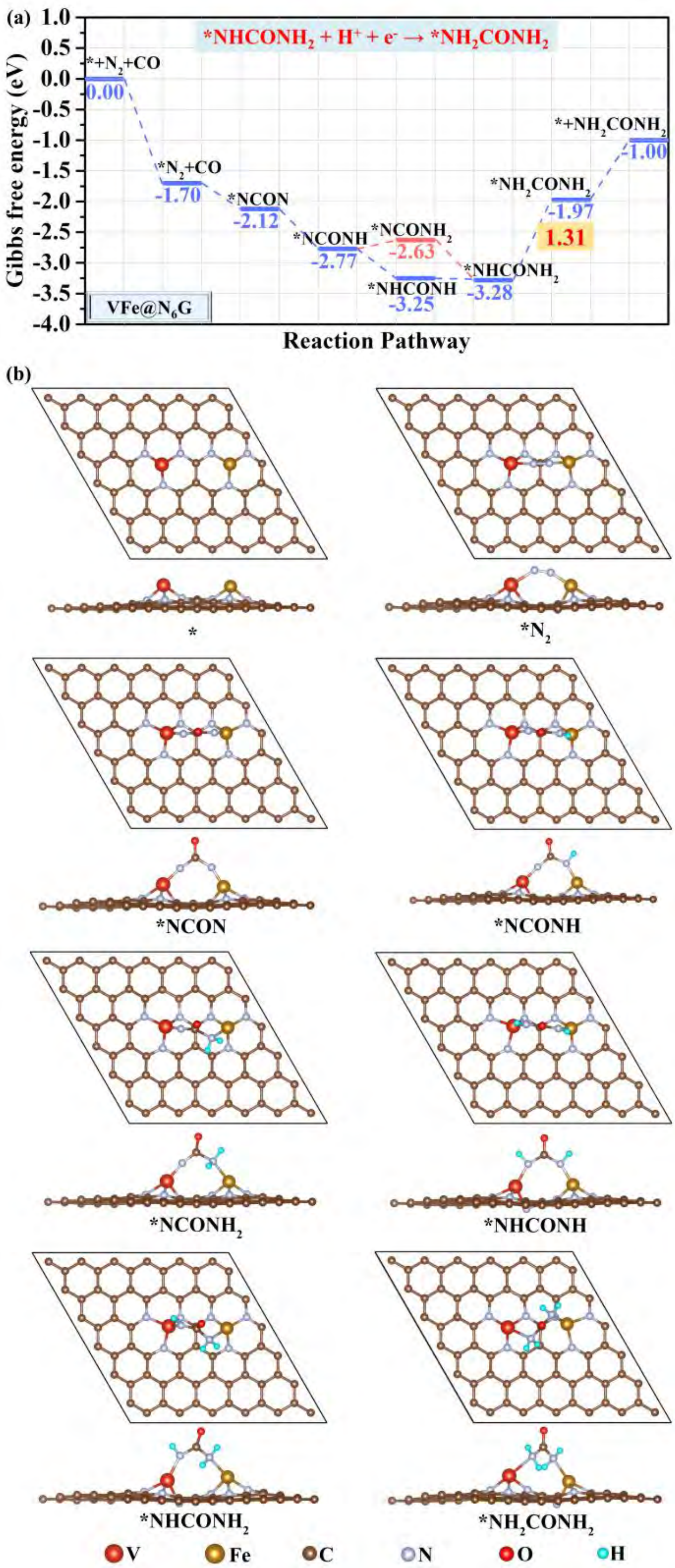

Figure S38. (a) Gibbs free energy diagram for urea production on the VFe@N_6G system. (b) Optimized structures of various intermediates along the hydrogenation pathway of urea production on the VFe@N_6G system.

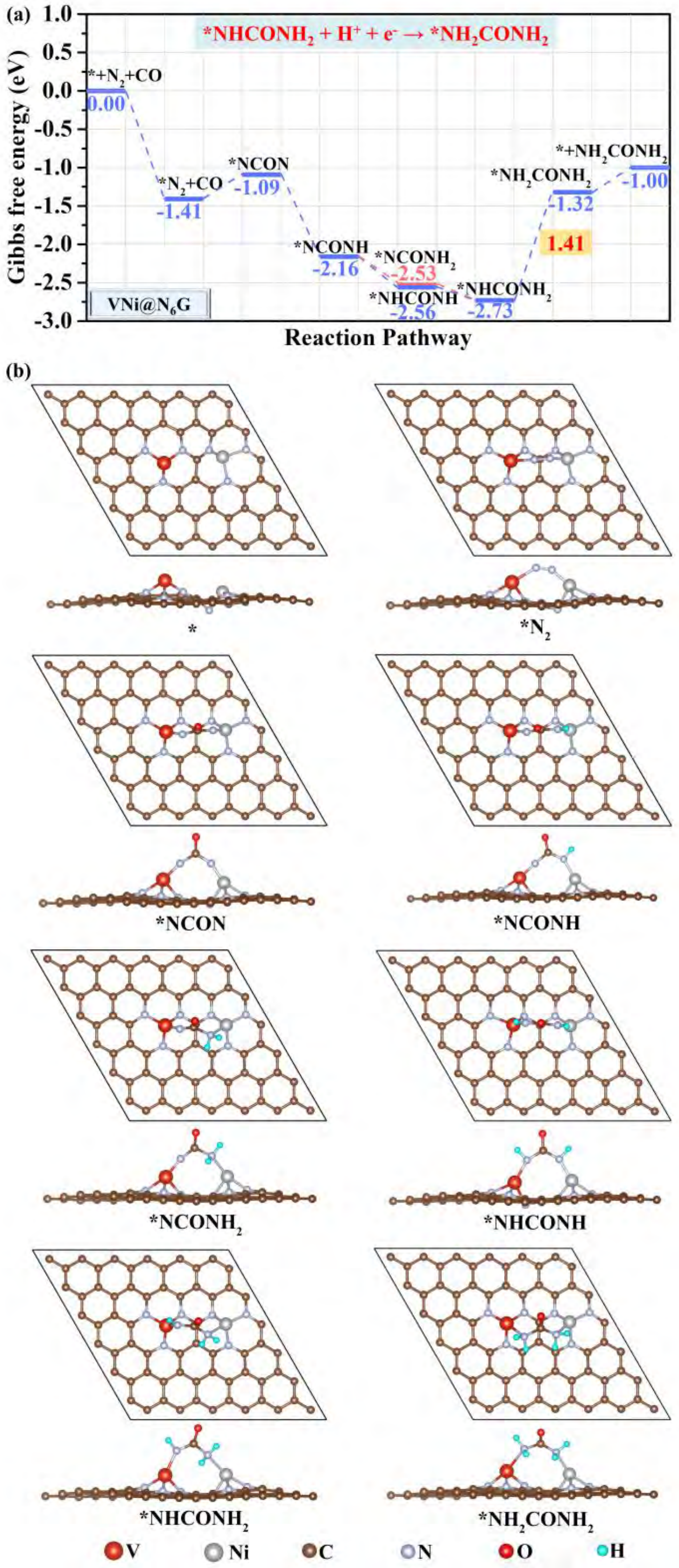

Figure S39. (a) Gibbs free energy diagram for urea production on the VNi@N_6G system. (b) Optimized structures of various intermediates along the hydrogenation pathway of urea production on the VNi@N_6G system.

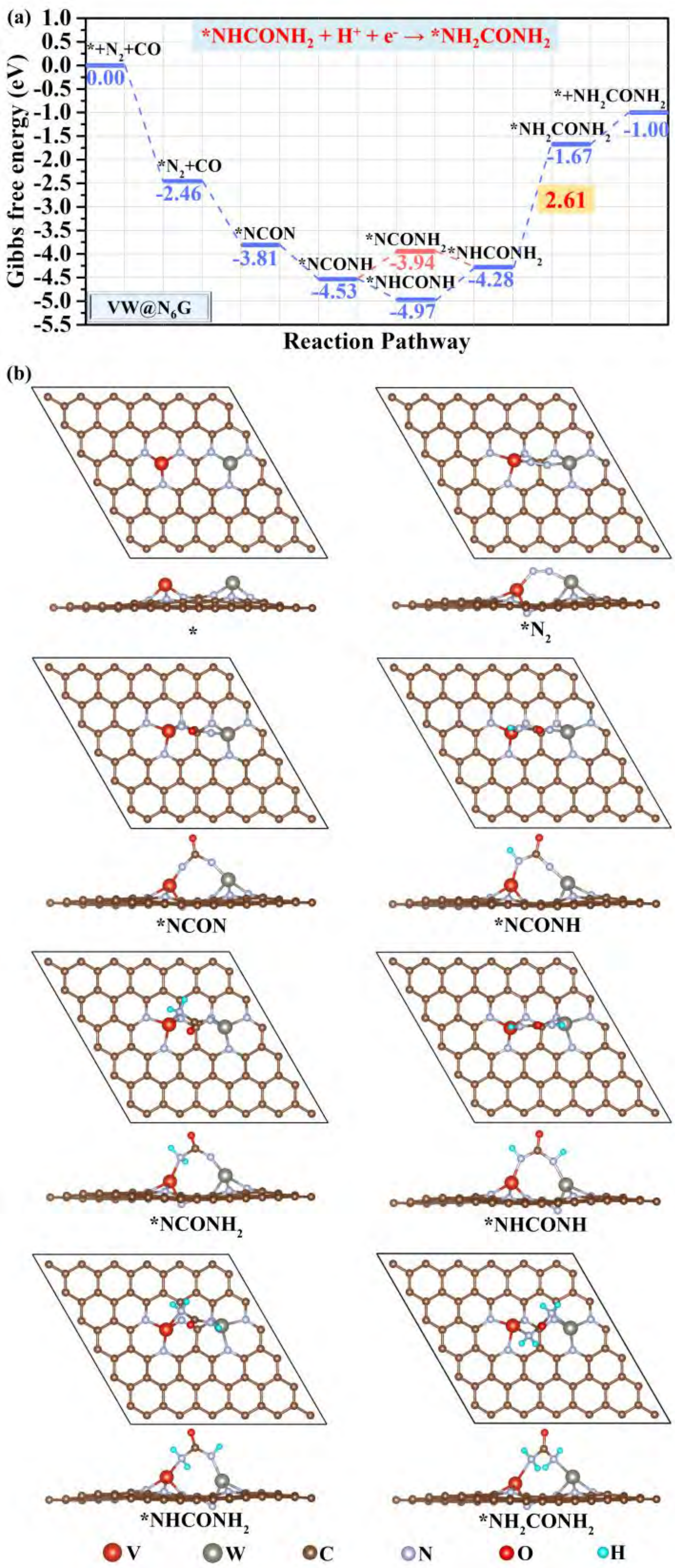

Figure S40. (a) Gibbs free energy diagram for urea production on the VW@N_6G system. (b) Optimized structures of various intermediates along the hydrogenation pathway of urea production on the VW@N_6G system.

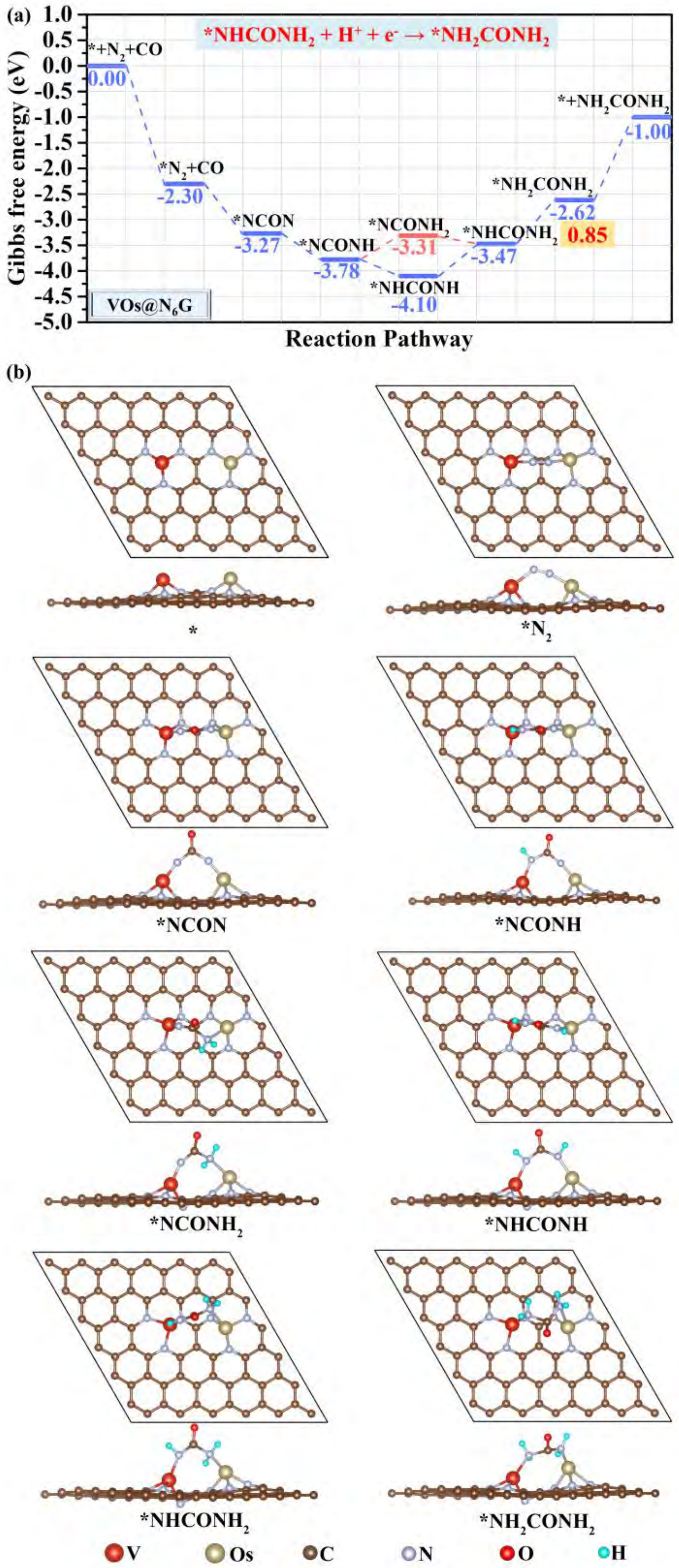

Figure S41. (a) Gibbs free energy diagram for urea production on the VOs@N_6G system. (b) Optimized structures of various intermediates along the hydrogenation pathway of urea production on the VOs@N_6G system.

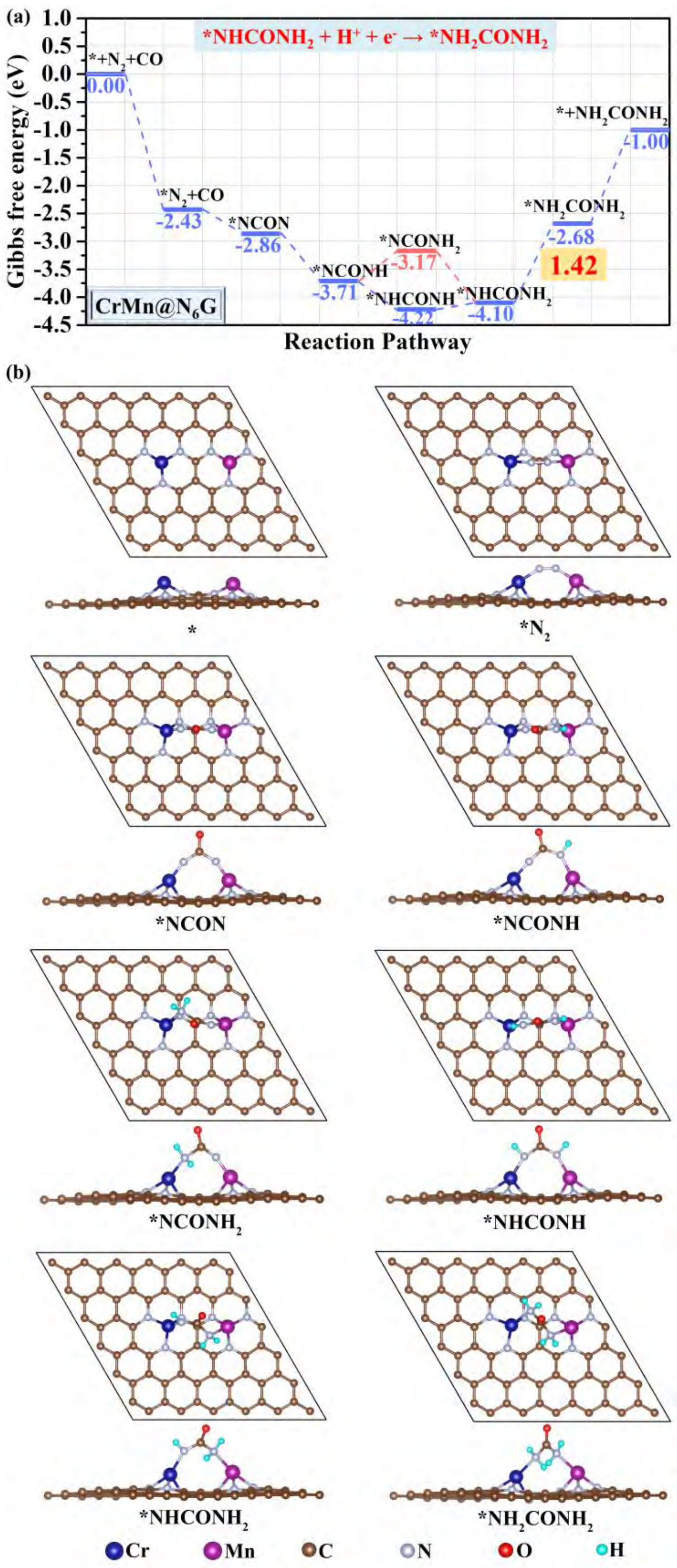

Figure S42. (a) Gibbs free energy diagram for urea production on the CrMn@N_6G system. (b) Optimized structures of various intermediates along the hydrogenation pathway of urea production on the CrMn@N_6G system.

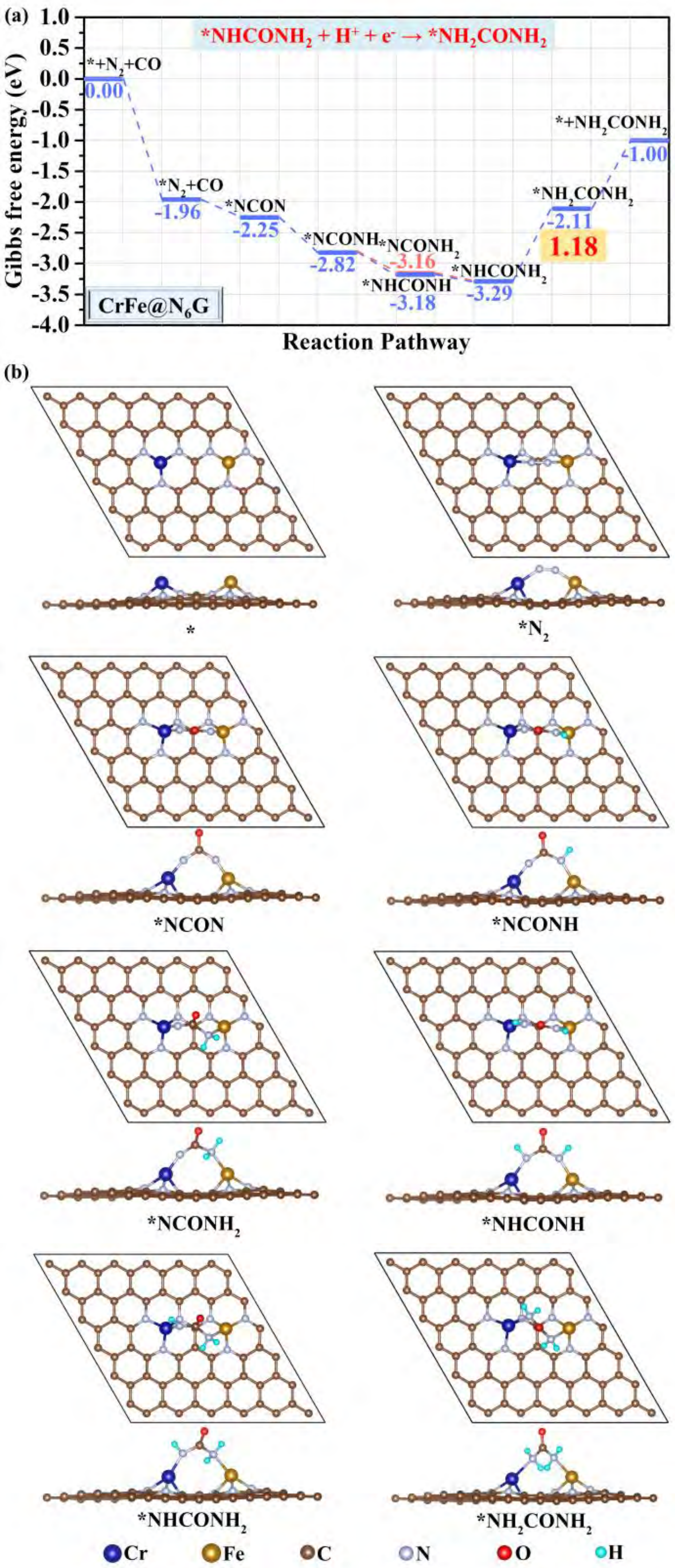

Figure S43. (a) Gibbs free energy diagram for urea production on the CrFe@N_6G system. (b) Optimized structures of various intermediates along the hydrogenation pathway of urea production on the CrFe@N_6G system.

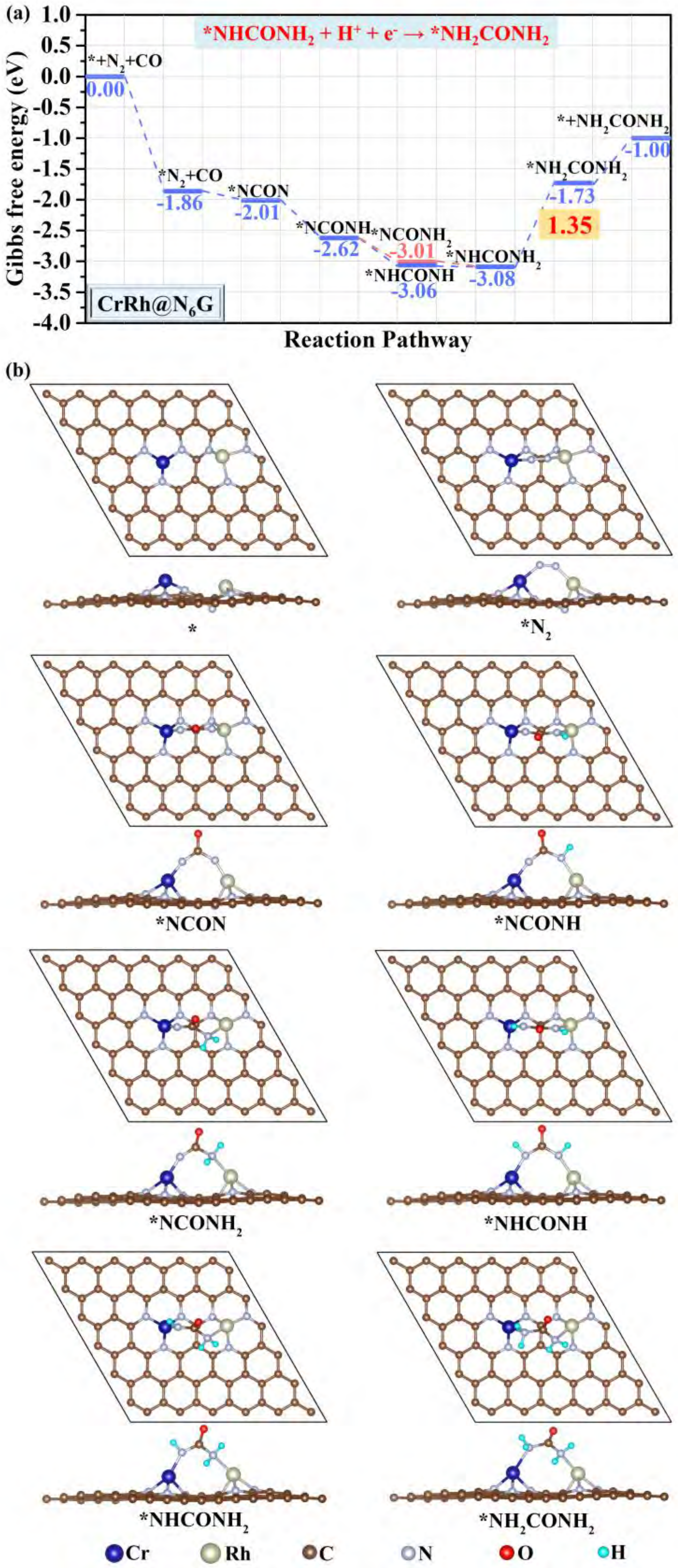

Figure S44. (a) Gibbs free energy diagram for urea production on the CrRh@N_6G system. (b) Optimized structures of various intermediates along the hydrogenation pathway of urea production on the CrRh@N_6G system.

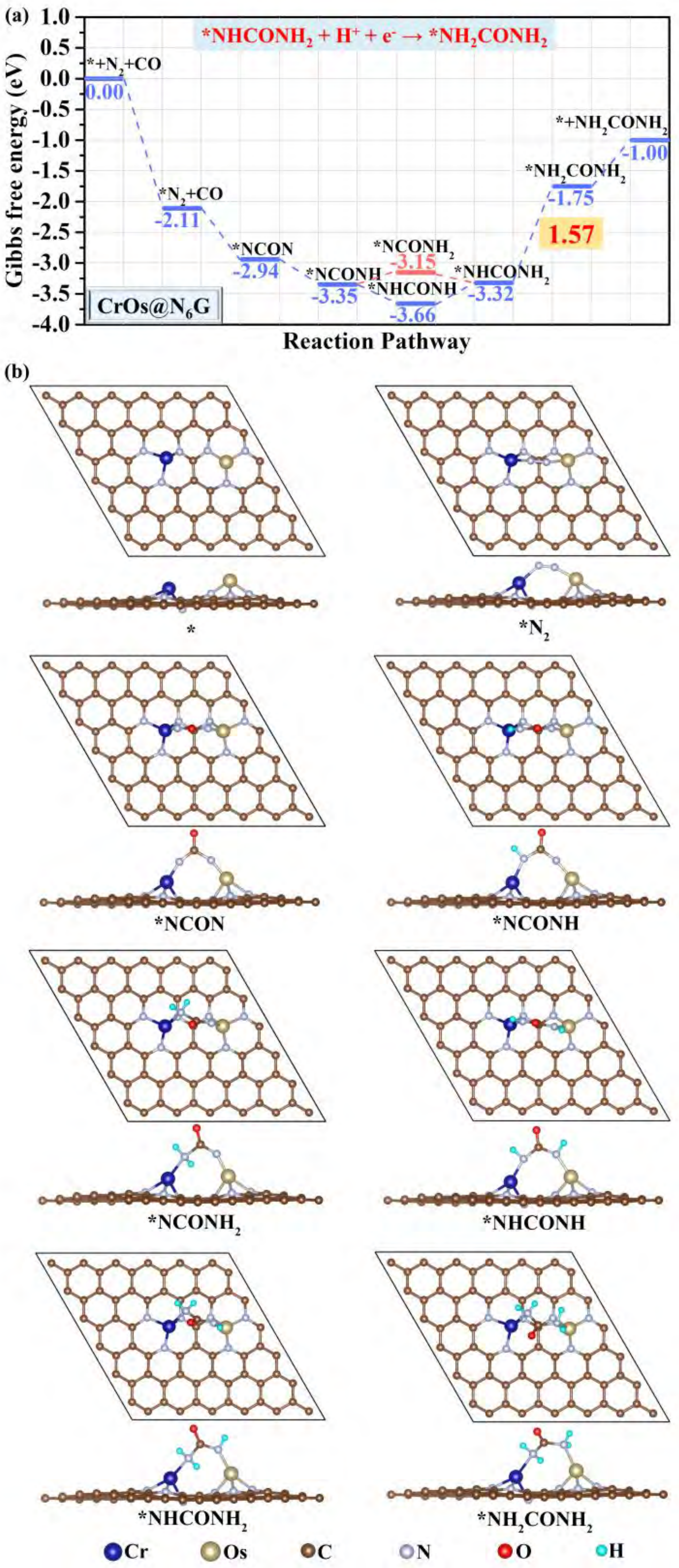

Figure S45. (a) Gibbs free energy diagram for urea production on the CrOs@N$_6$G system. (b) Optimized structures of various intermediates along the hydrogenation pathway of urea production on the CrOs@N$_6$G system.

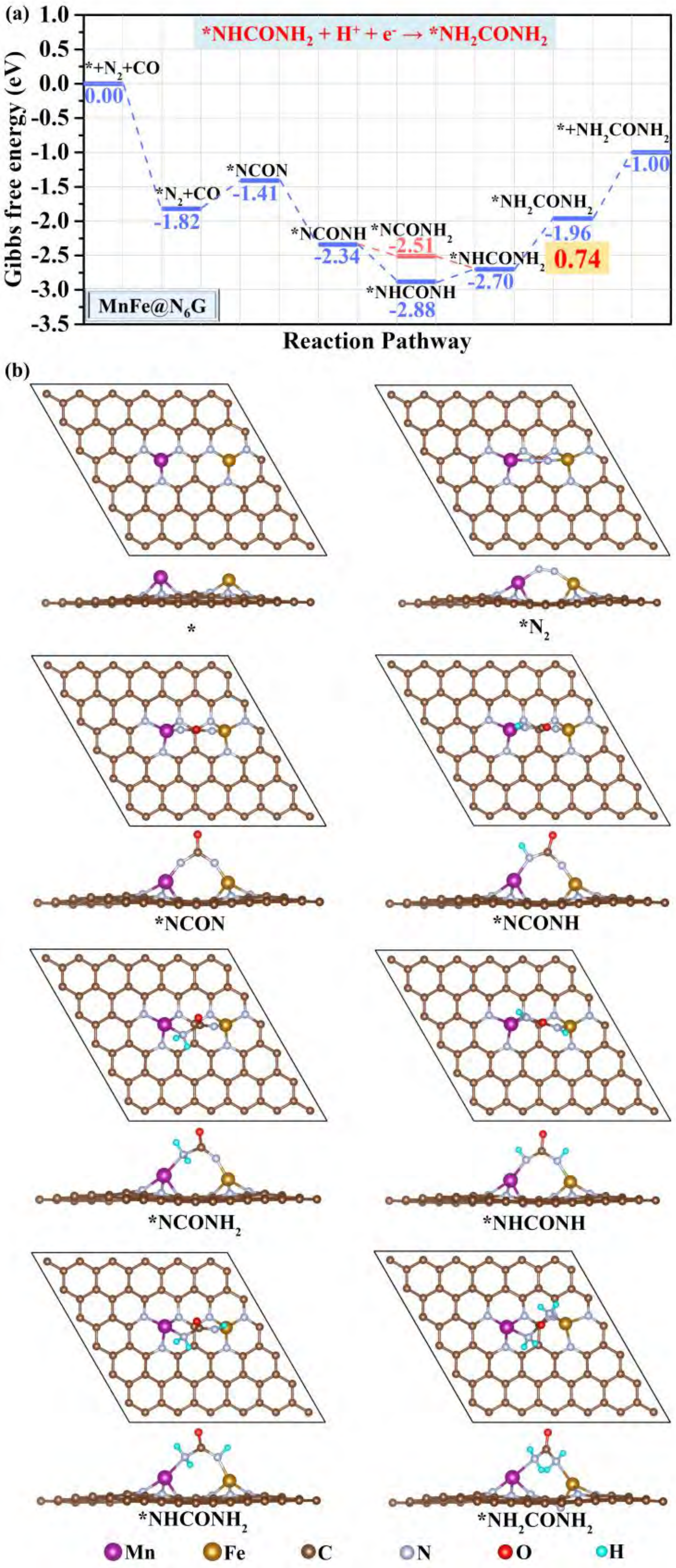

Figure S46. (a) Gibbs free energy diagram for urea production on the MnFe@N_6G system. (b) Optimized structures of various intermediates along the hydrogenation pathway of urea production on the MnFe@N_6G system.

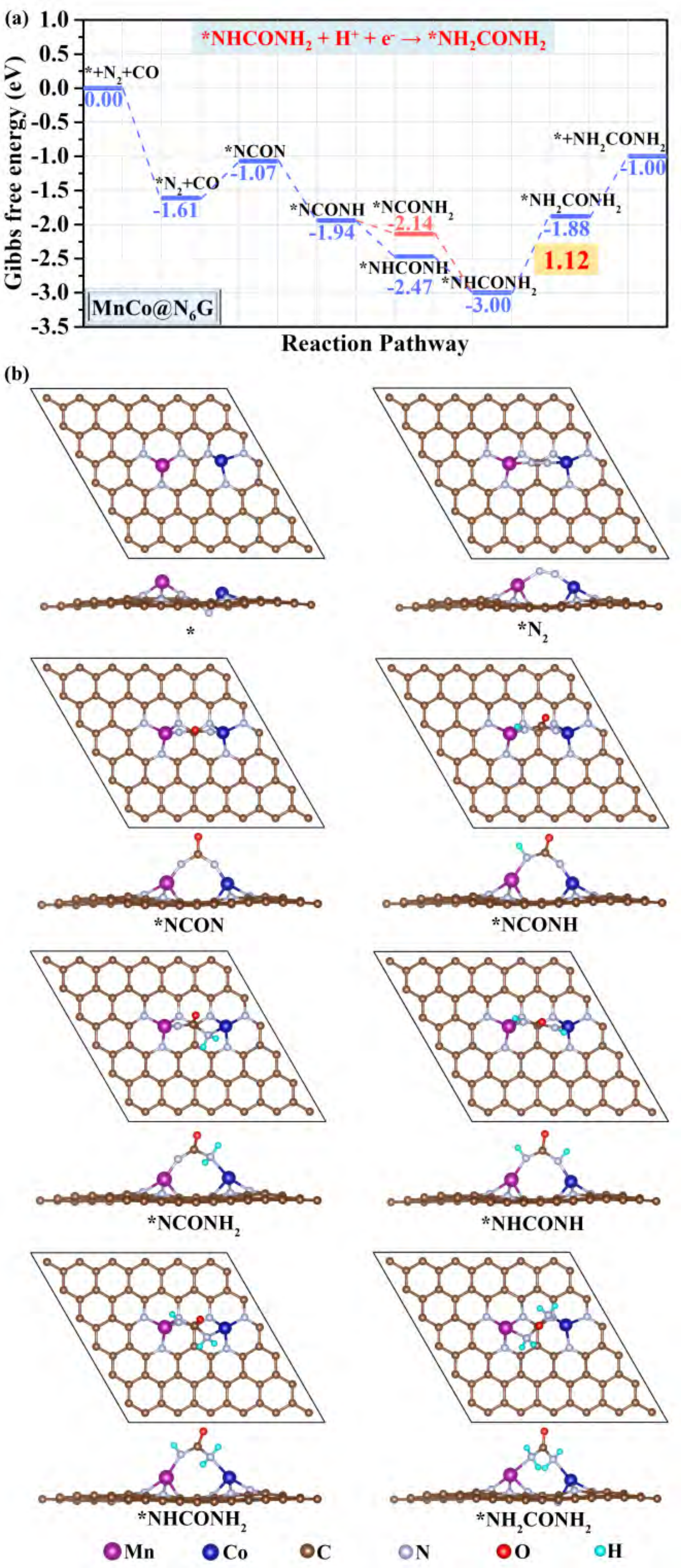

Figure S47. (a) Gibbs free energy diagram for urea production on the MnCo@N_6G system. (b) Optimized structures of various intermediates along the hydrogenation pathway of urea production on the MnCo@N_6G system.

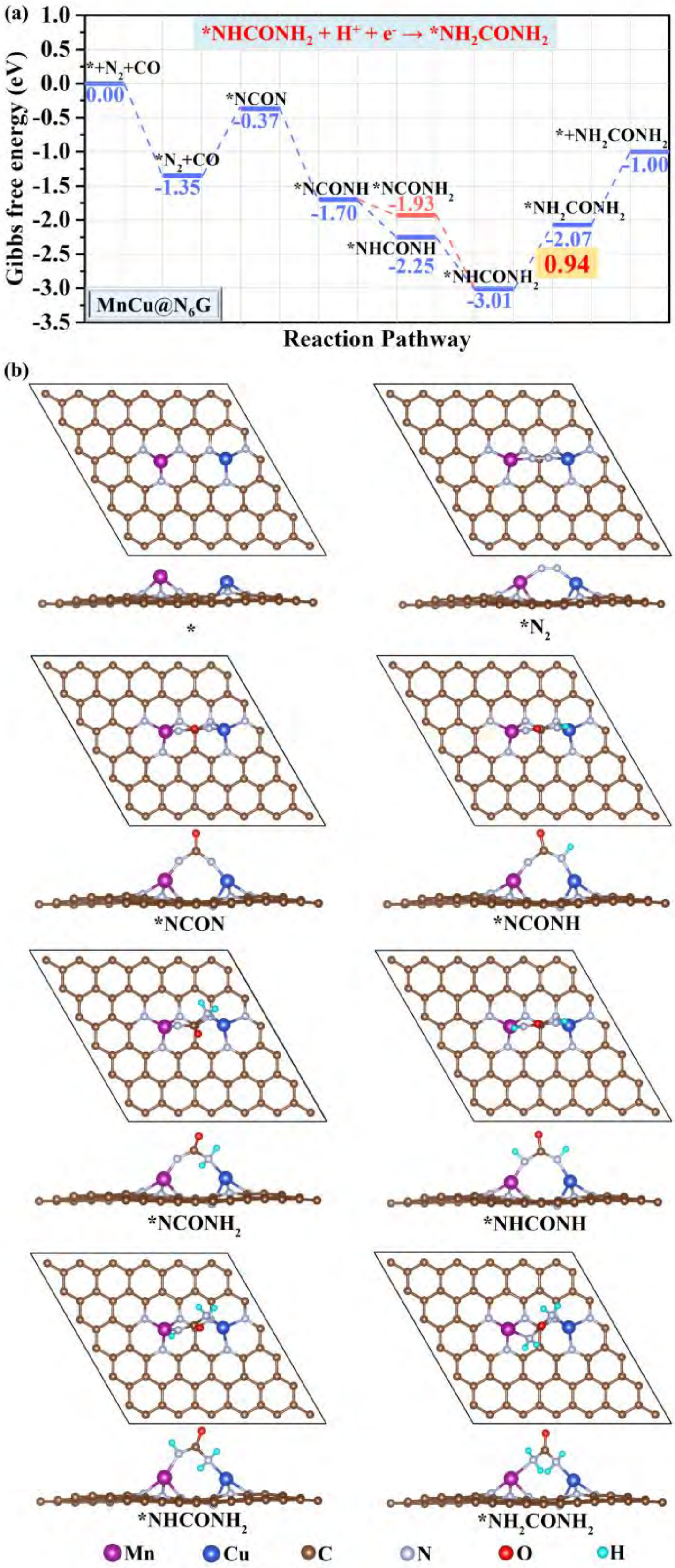

Figure S48. (a) Gibbs free energy diagram for urea production on the MnCu@N_6G system. (b) Optimized structures of various intermediates along the hydrogenation pathway of urea production on the MnCu@N_6G system.

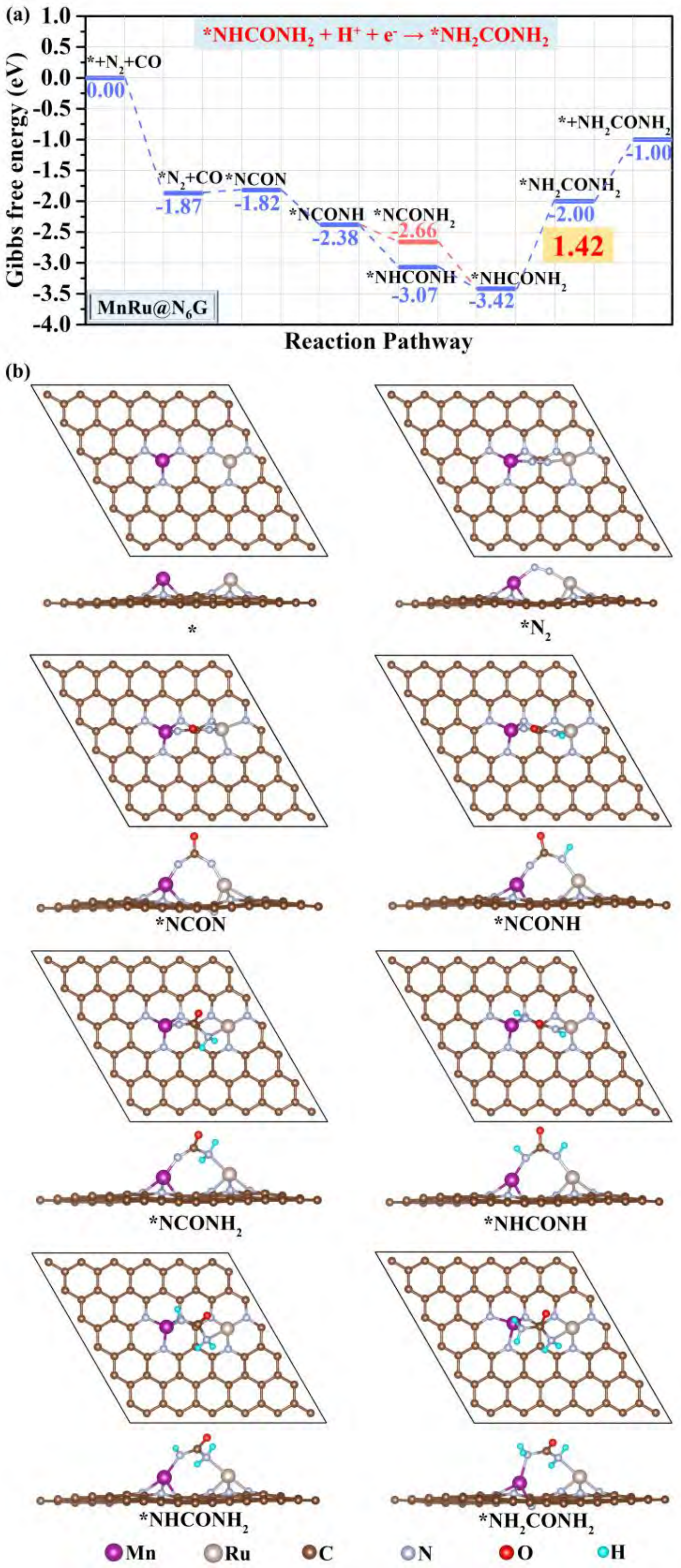

Figure S49. (a) Gibbs free energy diagram for urea production on the MnRu@N_6G system. (b) Optimized structures of various intermediates along the hydrogenation pathway of urea production on the MnRu@N_6G system.

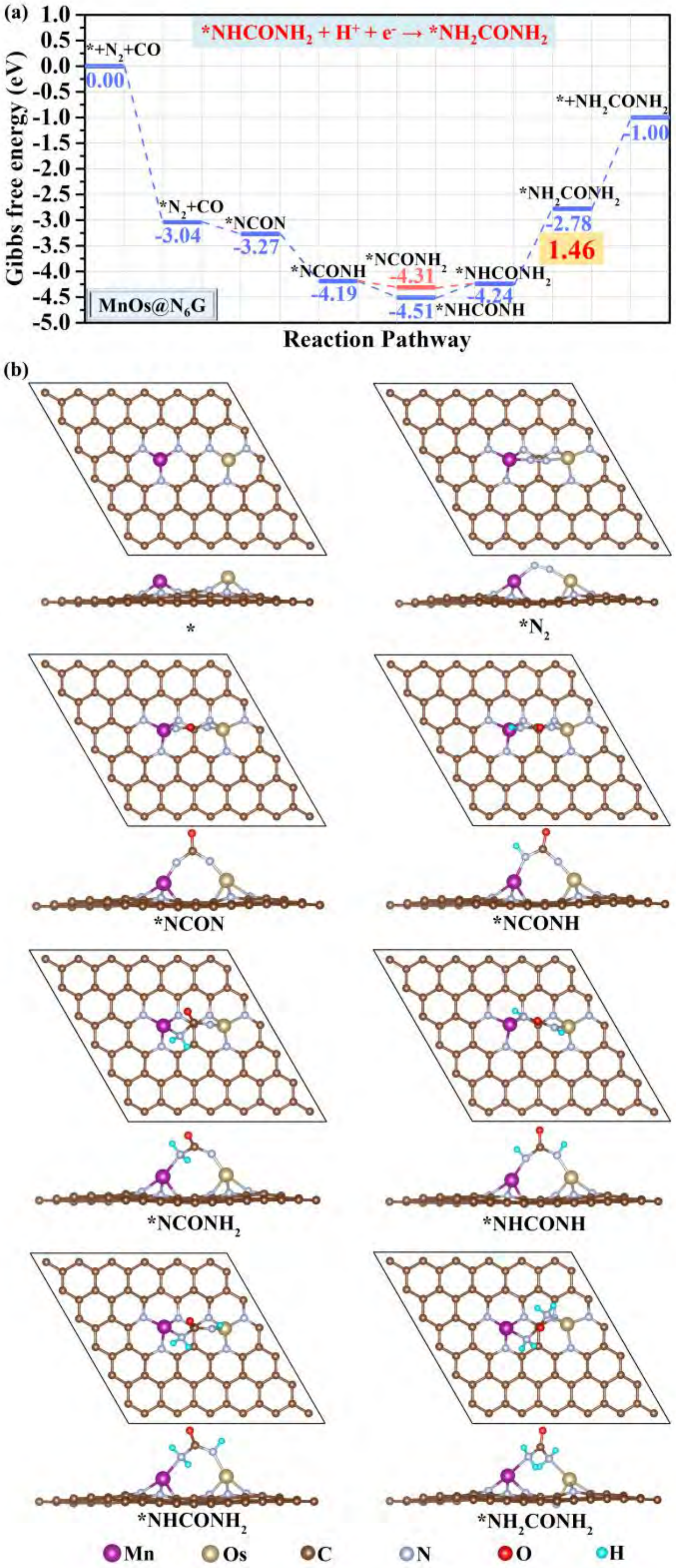

Figure S50. (a) Gibbs free energy diagram for urea production on the MnOs@N_6G system. (b) Optimized structures of various intermediates along the hydrogenation pathway of urea production on the MnOs@N_6G system.

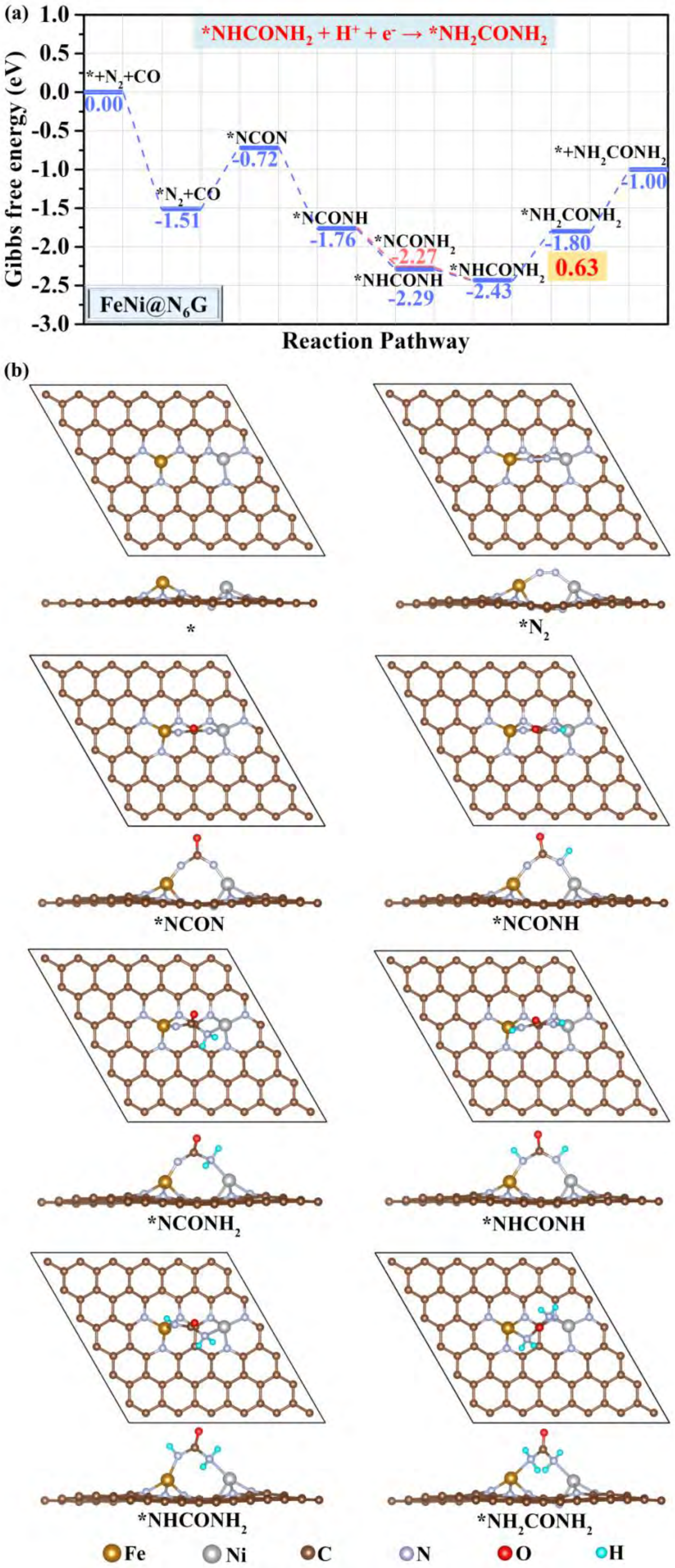

Figure S51. (a) Gibbs free energy diagram for urea production on the FeNi@N_6G system. (b) Optimized structures of various intermediates along the hydrogenation pathway of urea production on the FeNi@N_6G system.

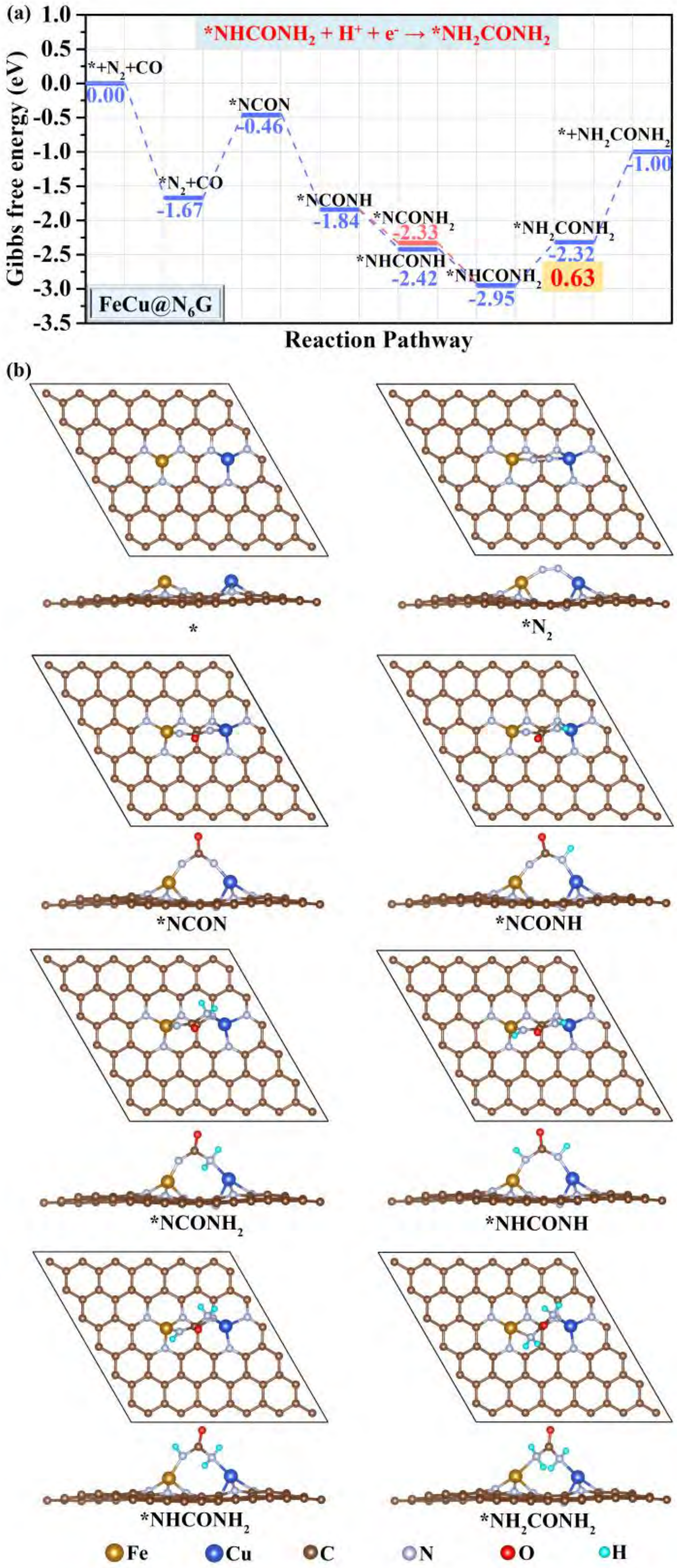

Figure S52. (a) Gibbs free energy diagram for urea production on the FeCu@N_6G system. (b) Optimized structures of various intermediates along the hydrogenation pathway of urea production on the FeCu@N_6G system.

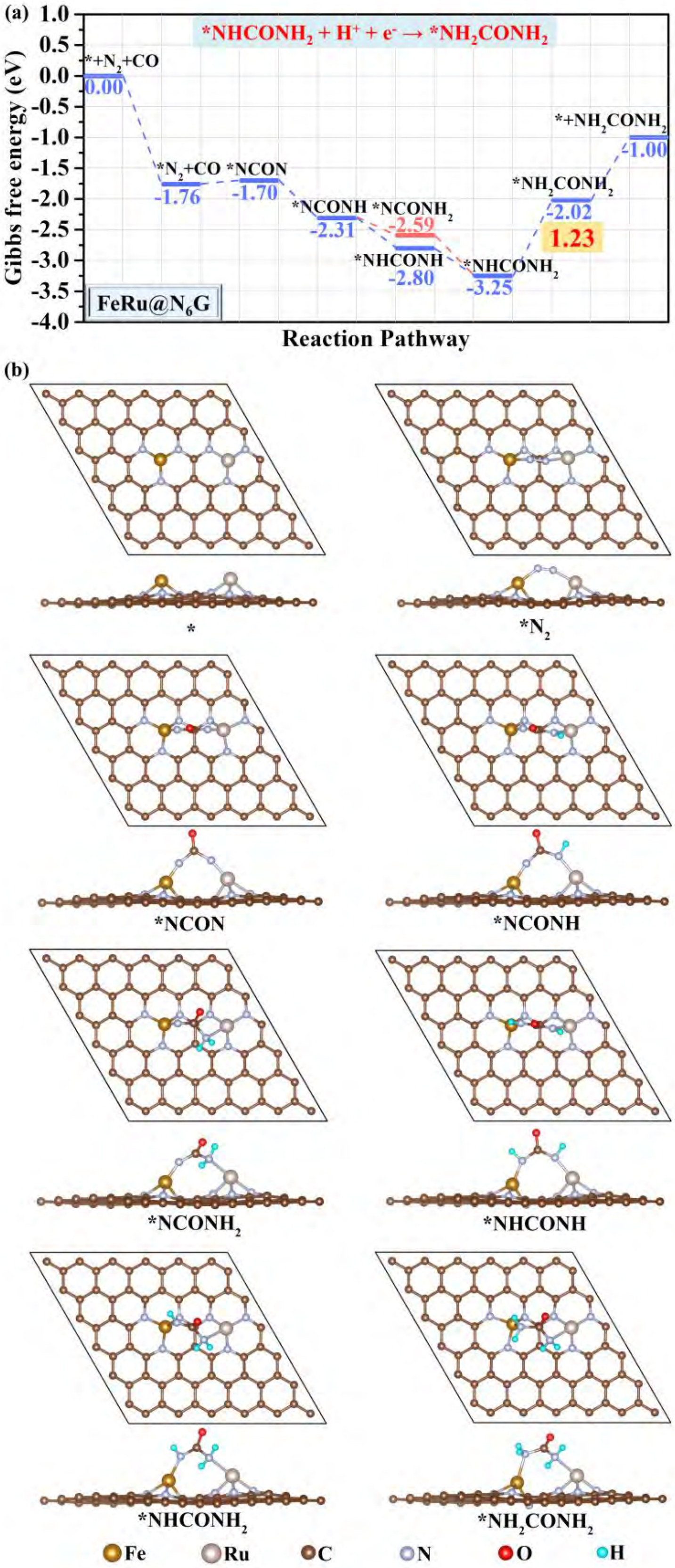

Figure S53. (a) Gibbs free energy diagram for urea production on the FeRu@N$_6$G system. (b) Optimized structures of various intermediates along the hydrogenation pathway of urea production on the FeRu@N$_6$G system.

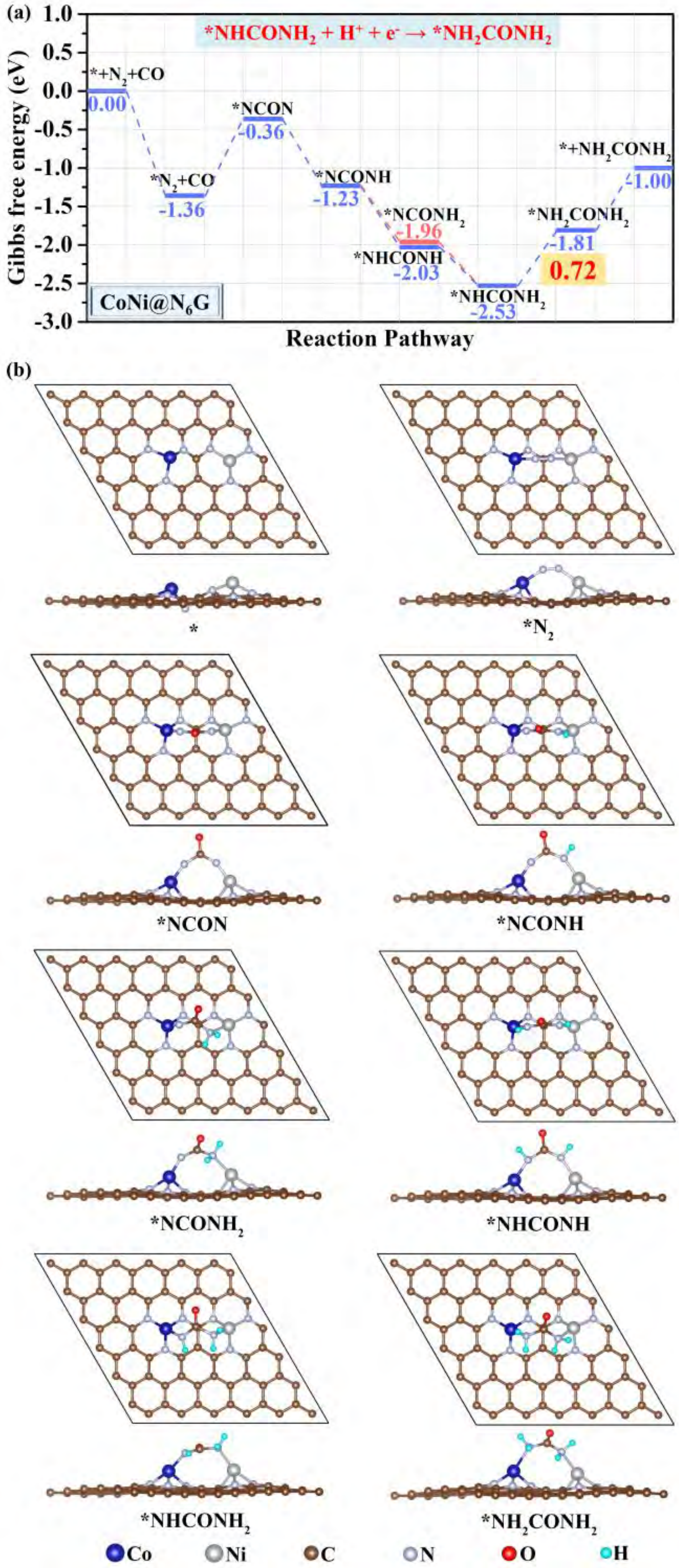

Figure S54. (a) Gibbs free energy diagram for urea production on the CoNi@N_6G system. (b) Optimized structures of various intermediates along the hydrogenation pathway of urea production on the CoNi@N_6G system.

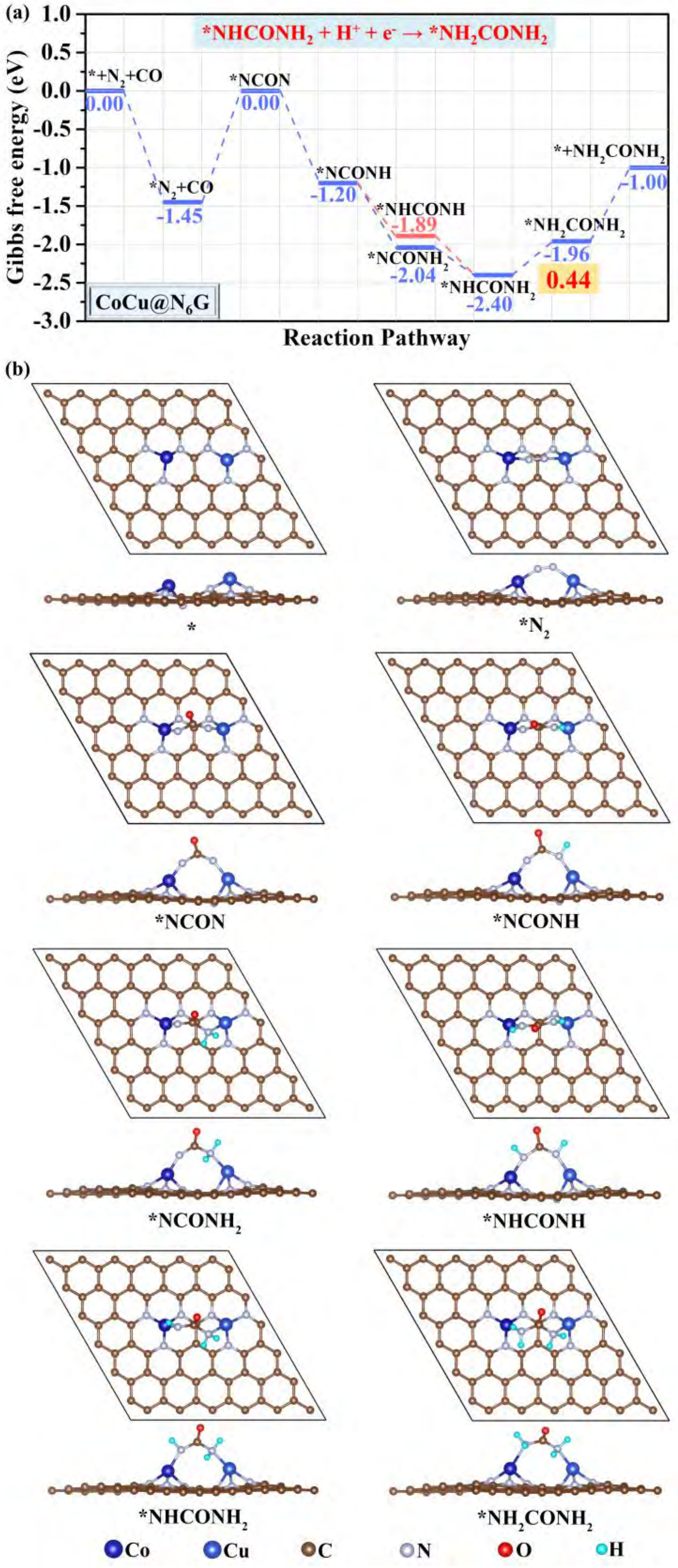

Figure S55. (a) Gibbs free energy diagram for urea production on the CoCu@N_6G system. (b) Optimized structures of various intermediates along the hydrogenation pathway of urea production on the CoCu@N_6G system.

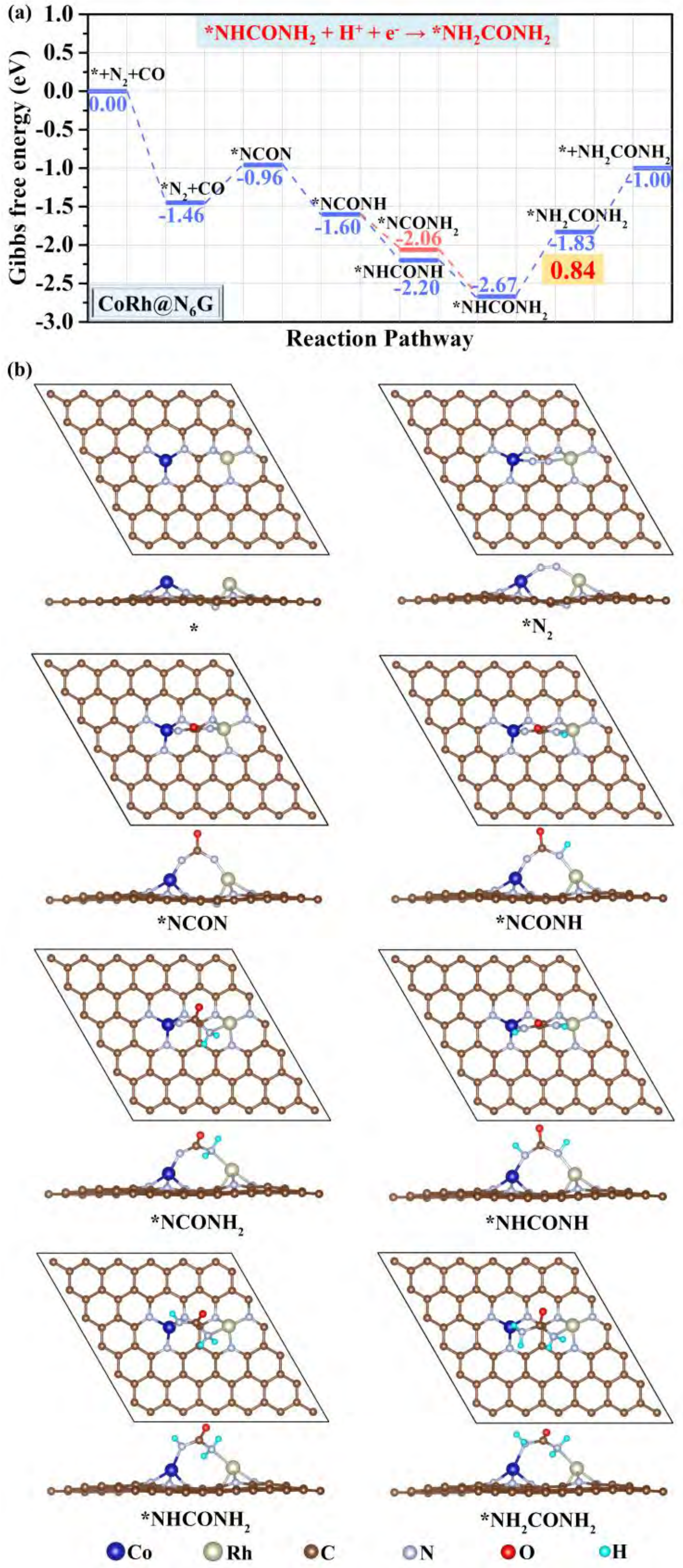

Figure S56. (a) Gibbs free energy diagram for urea production on the CoRh@N$_6$G system. (b) Optimized structures of various intermediates along the hydrogenation pathway of urea production on the CoRh@N$_6$G system.

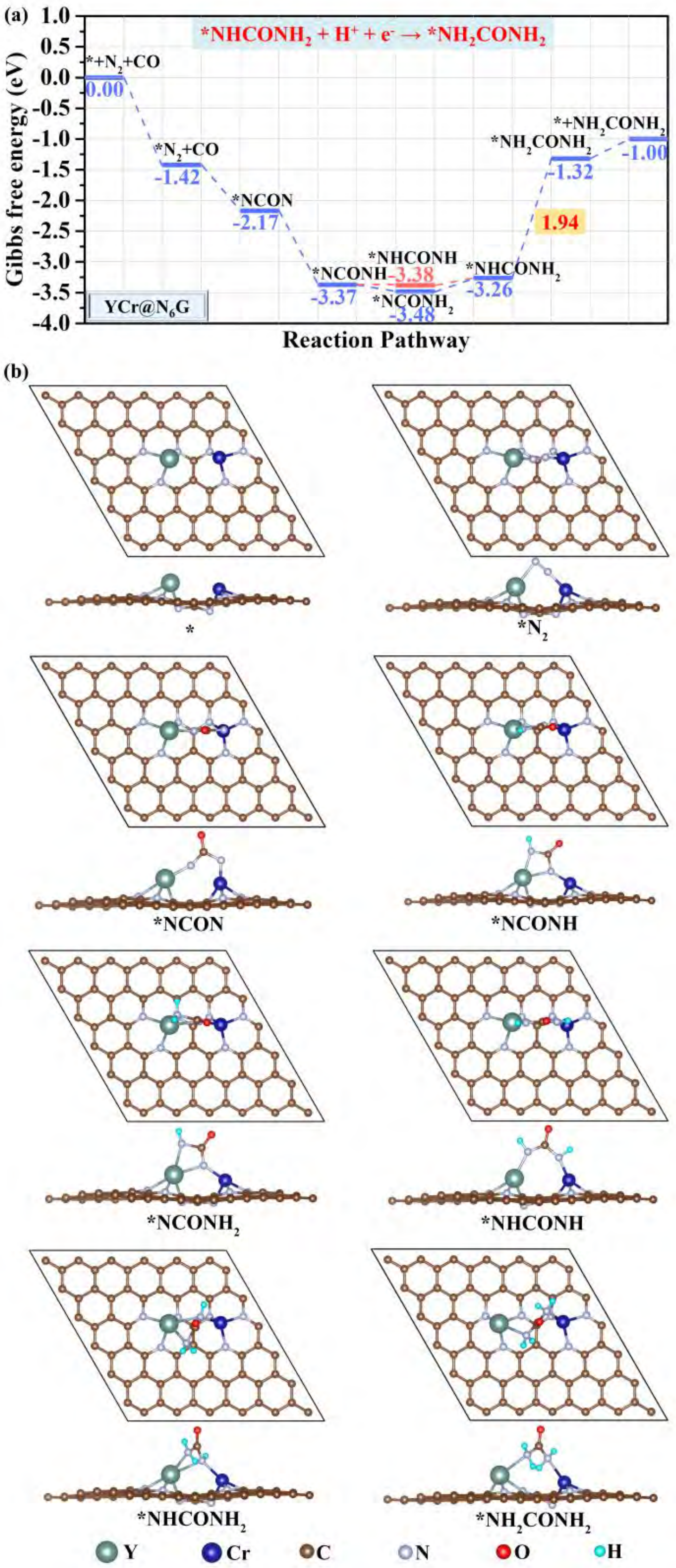

Figure S57. (a) Gibbs free energy diagram for urea production on the YCr@N_6G system. (b) Optimized structures of various intermediates along the hydrogenation pathway of urea production on the YCr@N_6G system.

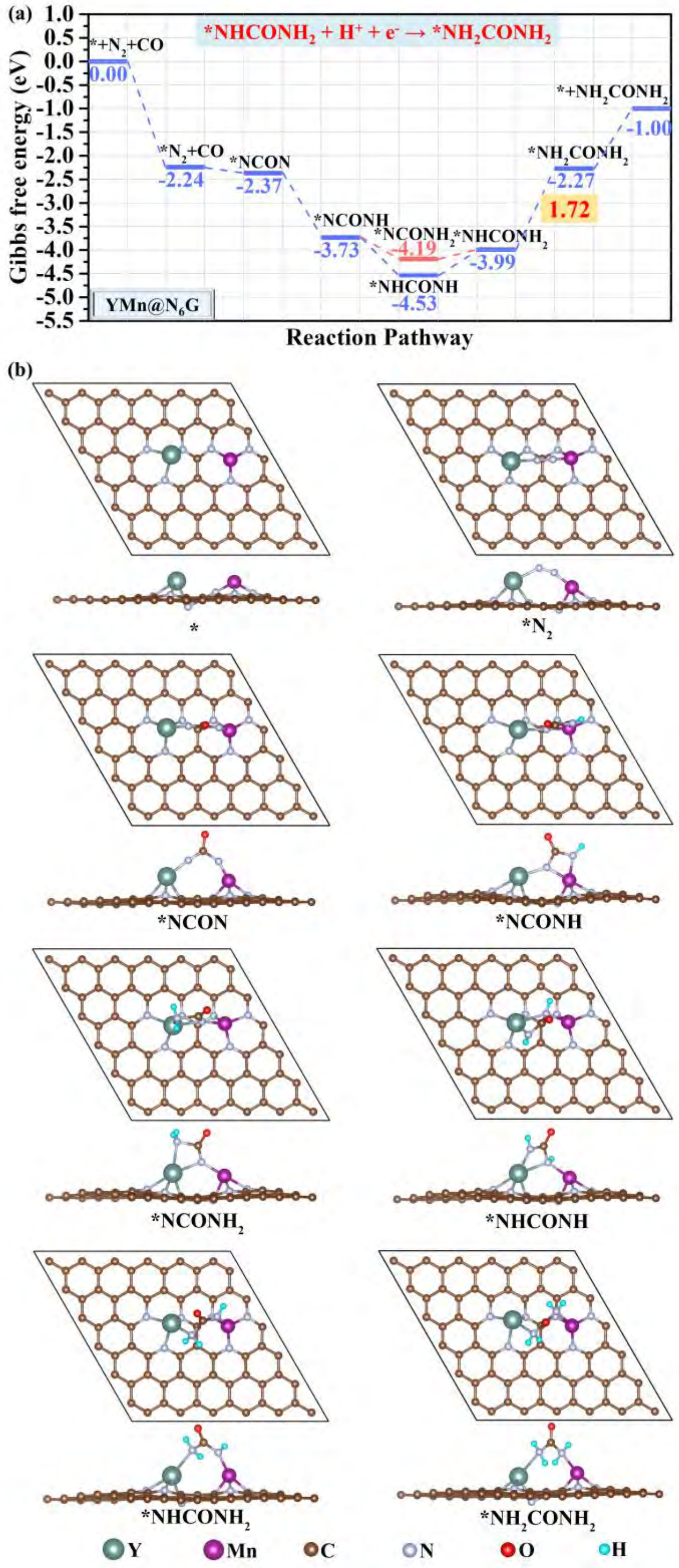

Figure S58. (a) Gibbs free energy diagram for urea production on the YMn@N_6G system. (b) Optimized structures of various intermediates along the hydrogenation pathway of urea production on the YMn@N_6G system.

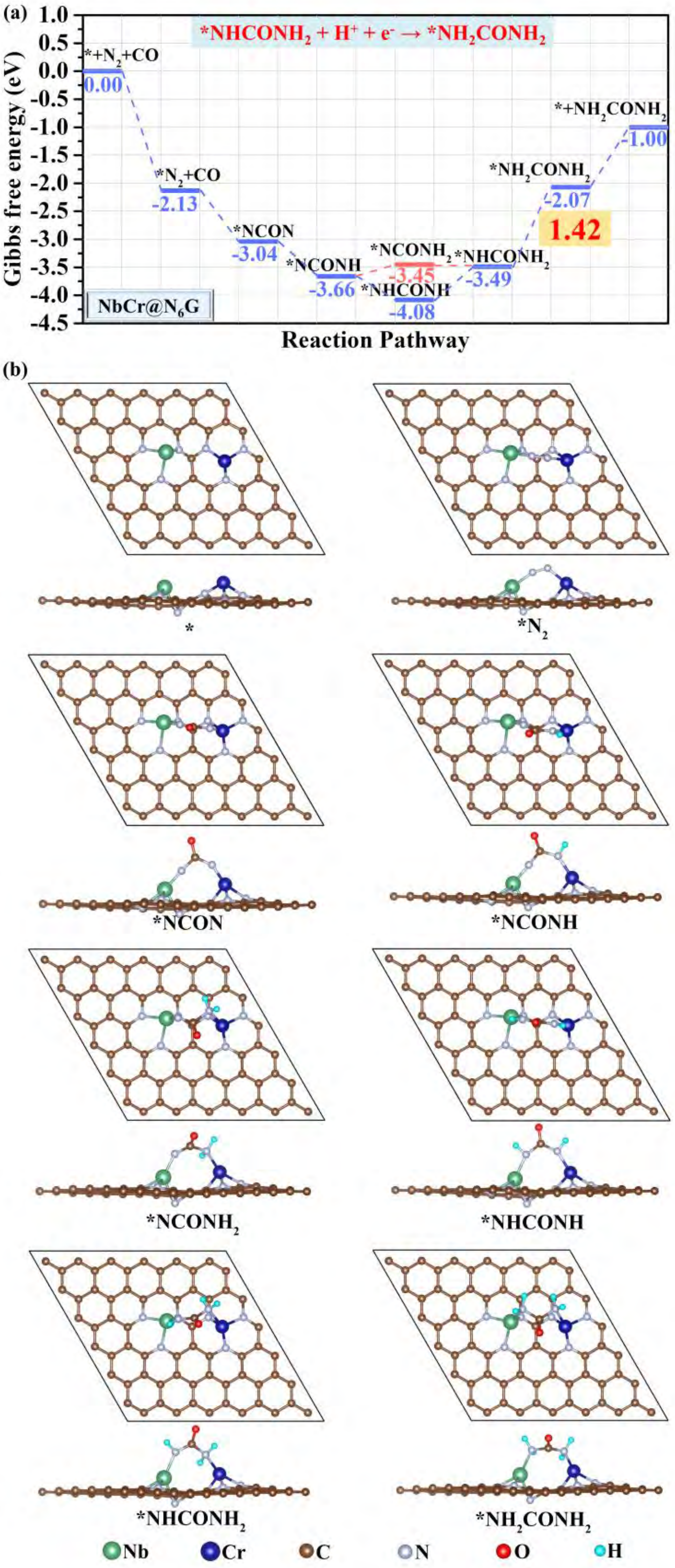

Figure S59. (a) Gibbs free energy diagram for urea production on the NbCr@N_6G system. (b) Optimized structures of various intermediates along the hydrogenation pathway of urea production on the NbCr@N_6G system.

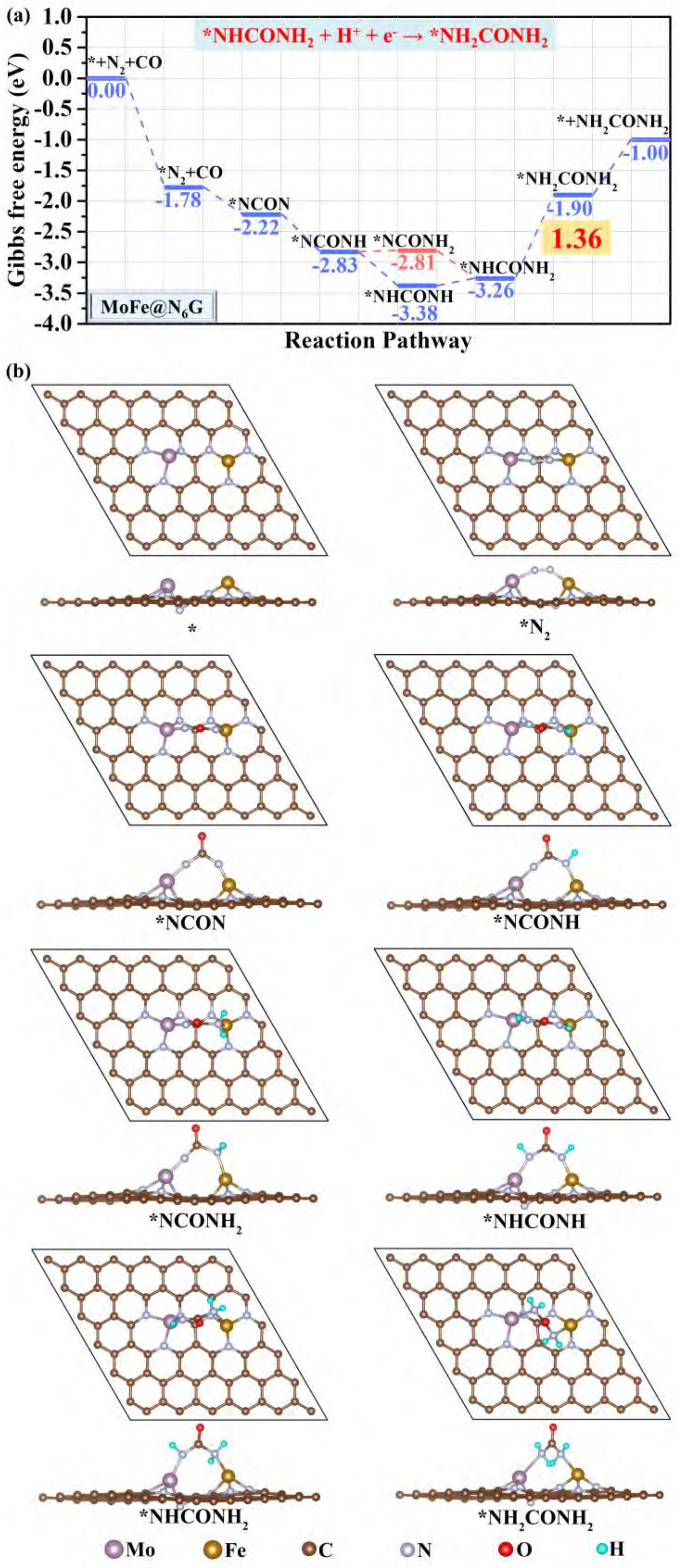

Figure S60. (a) Gibbs free energy diagram for urea production on the MoFe@N_6G system. (b) Optimized structures of various intermediates along the hydrogenation pathway of urea production on the MoFe@N_6G system.

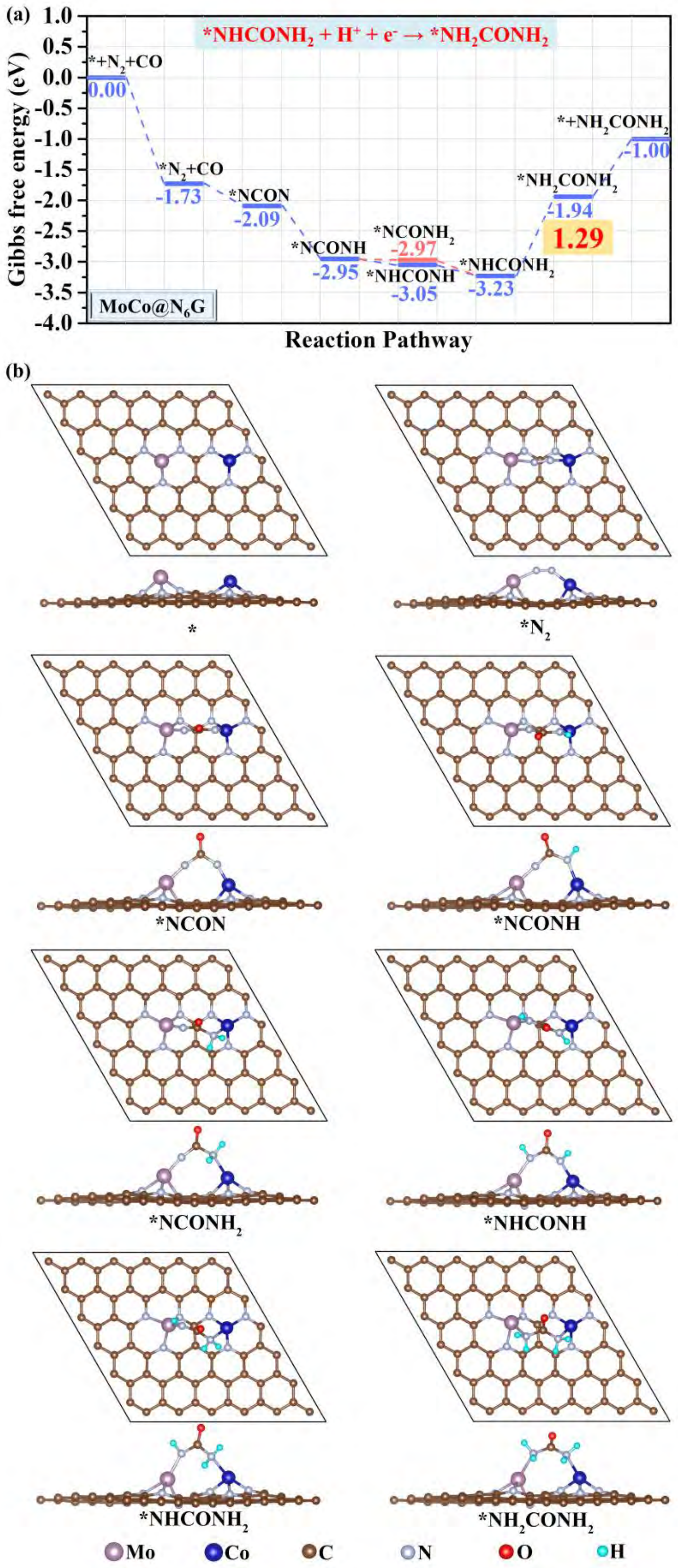

Figure S61. (a) Gibbs free energy diagram for urea production on the MoCo@N_6G system. (b) Optimized structures of various intermediates along the hydrogenation pathway of urea production on the MoCo@N_6G system.

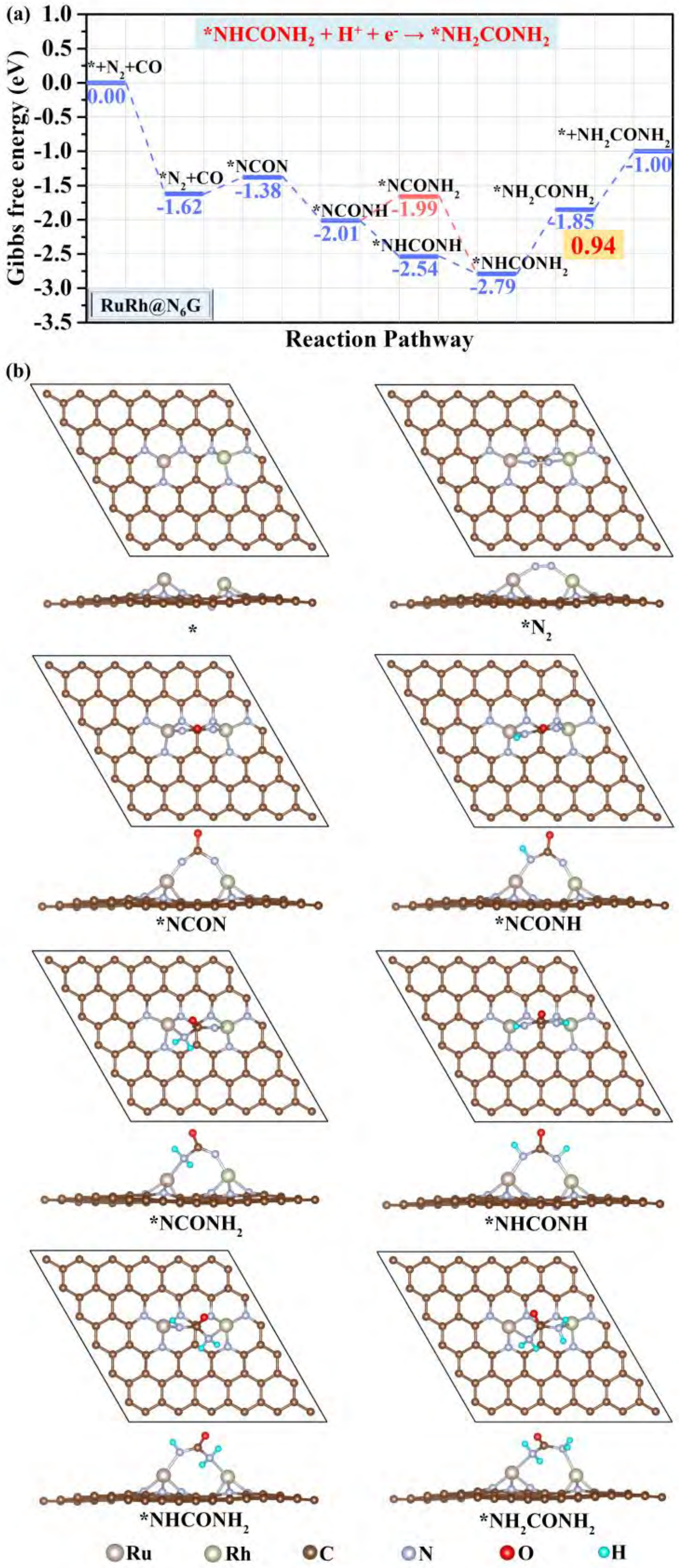

Figure S62. (a) Gibbs free energy diagram for urea production on the RuRh@N_6G system. (b) Optimized structures of various intermediates along the hydrogenation pathway of urea production on the RuRh@N_6G system.

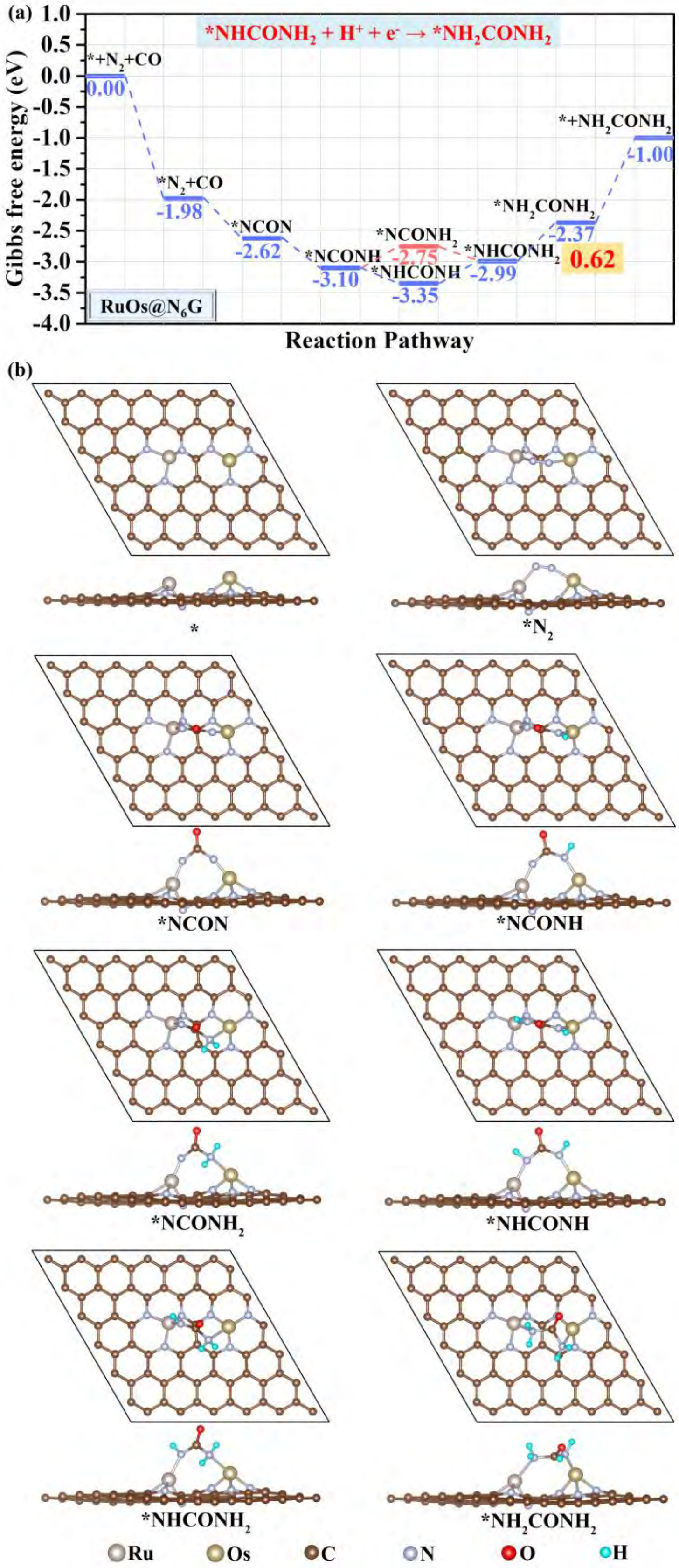

Figure S63. (a) Gibbs free energy diagram for urea production on the RuOs@N_6G system. (b) Optimized structures of various intermediates along the hydrogenation pathway of urea production on the RuOs@N_6G system.

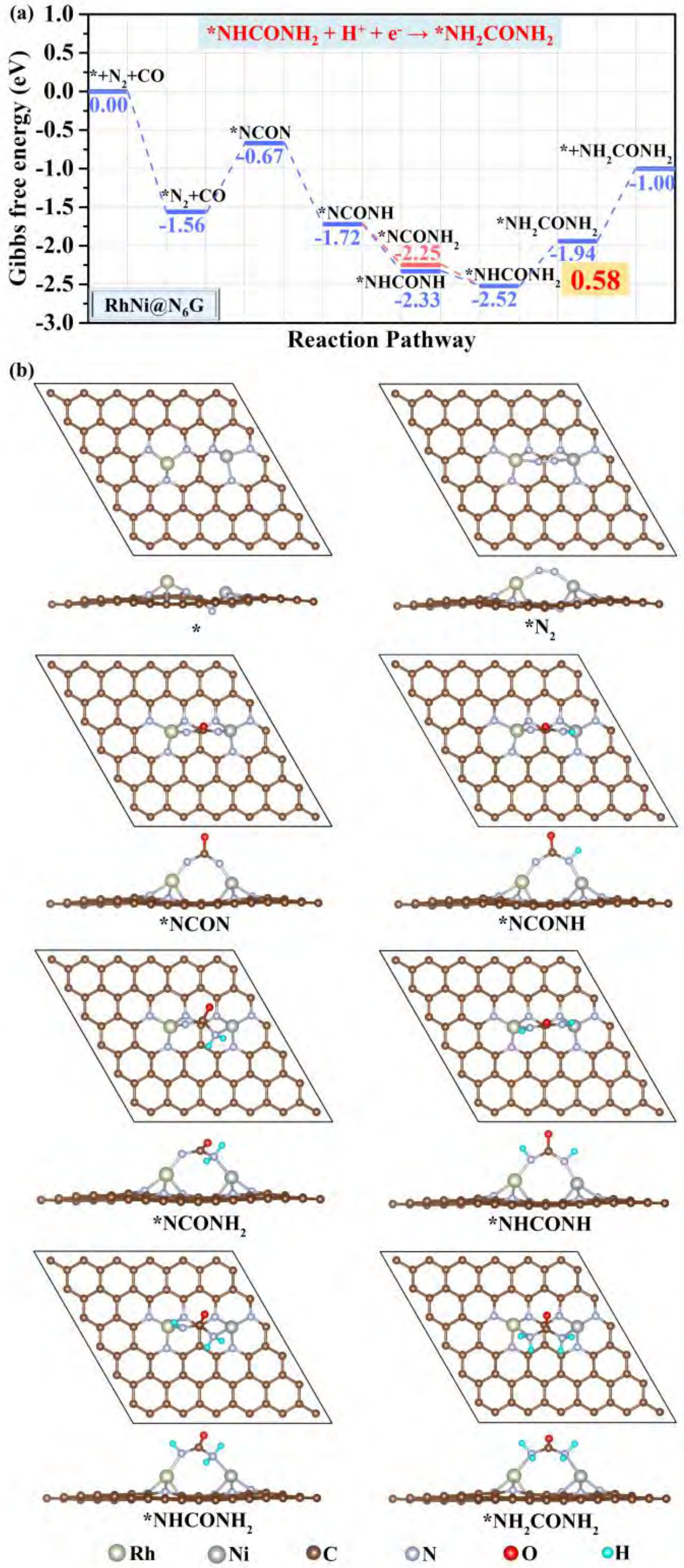

Figure S64. (a) Gibbs free energy diagram for urea production on the RhNi@N_6G system. (b) Optimized structures of various intermediates along the hydrogenation pathway of urea production on the RhNi@N_6G system.

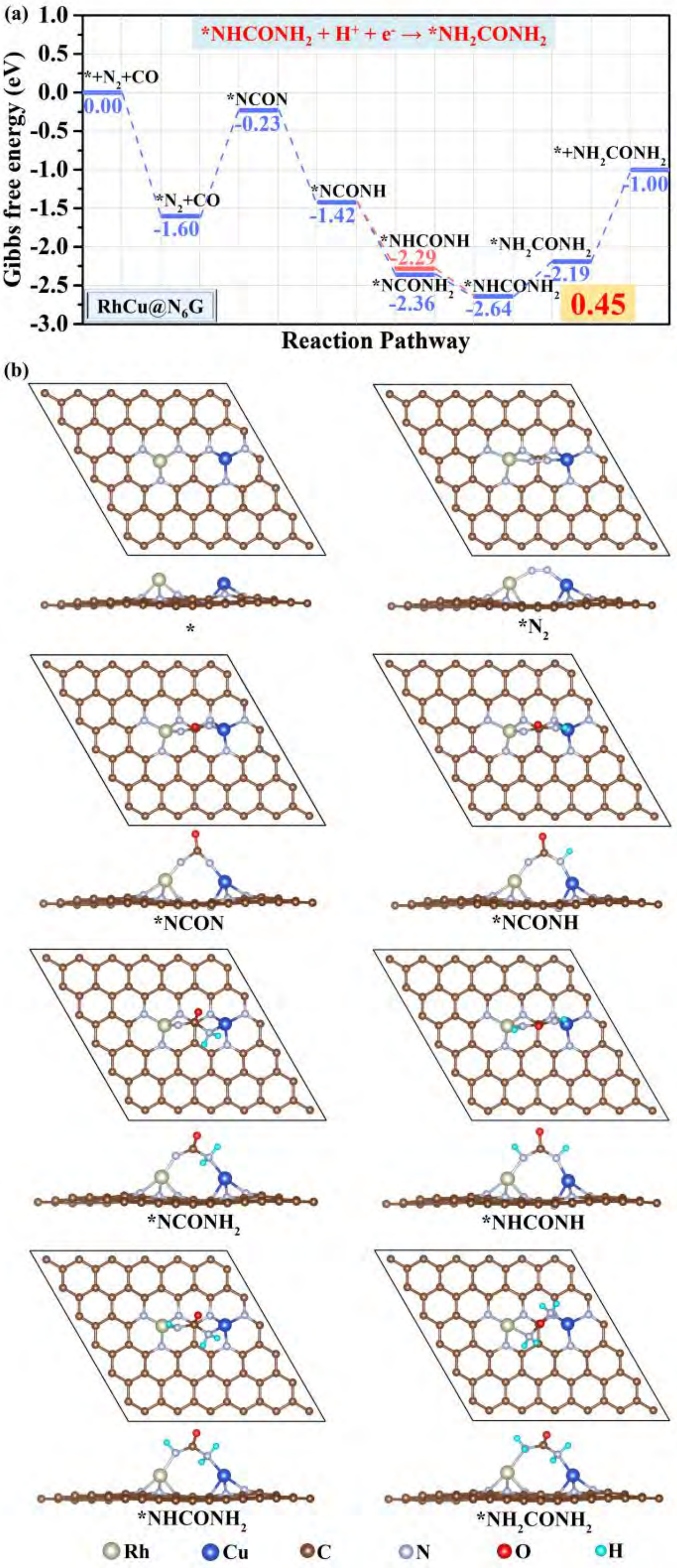

Figure S65. (a) Gibbs free energy diagram for urea production on the RhCu@N_6G system. (b) Optimized structures of various intermediates along the hydrogenation pathway of urea production on the RhCu@N_6G system.

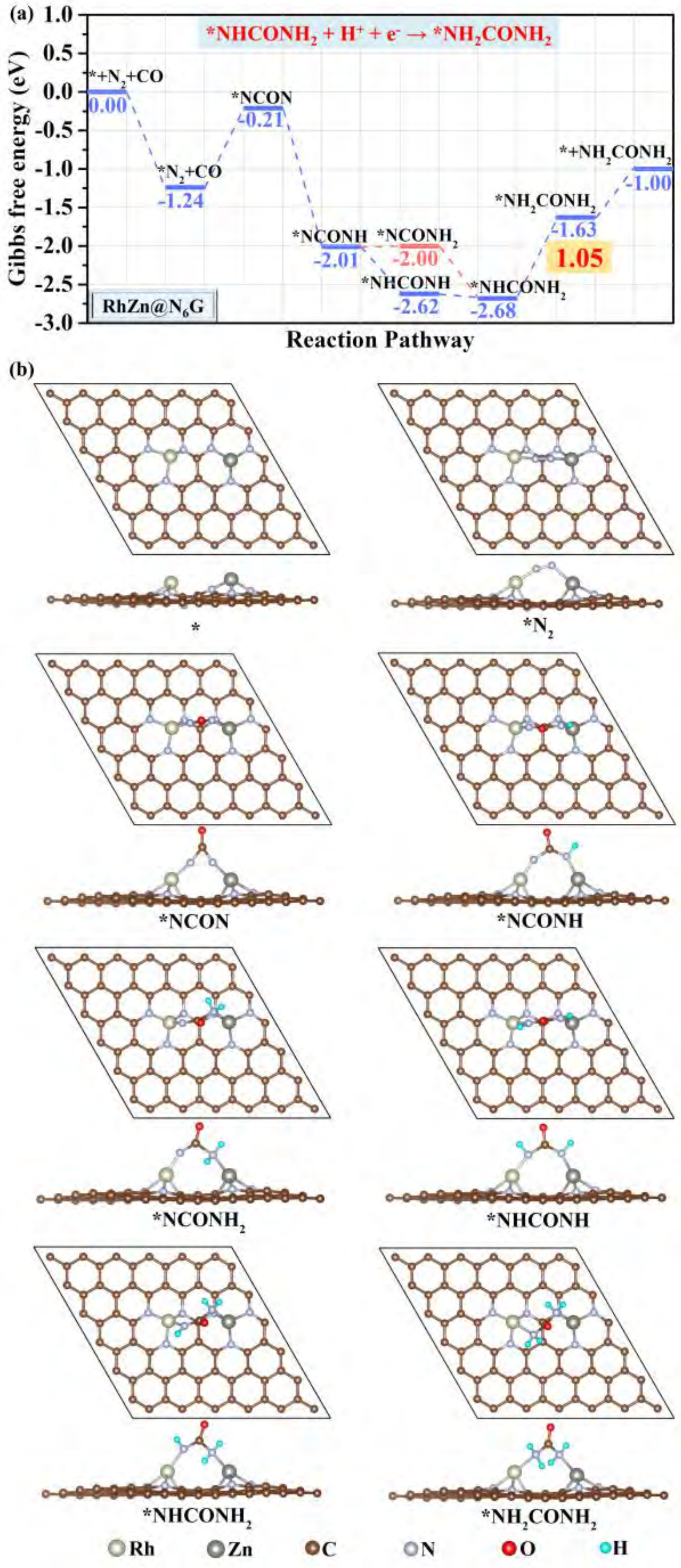

Figure S66. (a) Gibbs free energy diagram for urea production on the RhZn@N_6G system. (b) Optimized structures of various intermediates along the hydrogenation pathway of urea production on the RhZn@N_6G system.

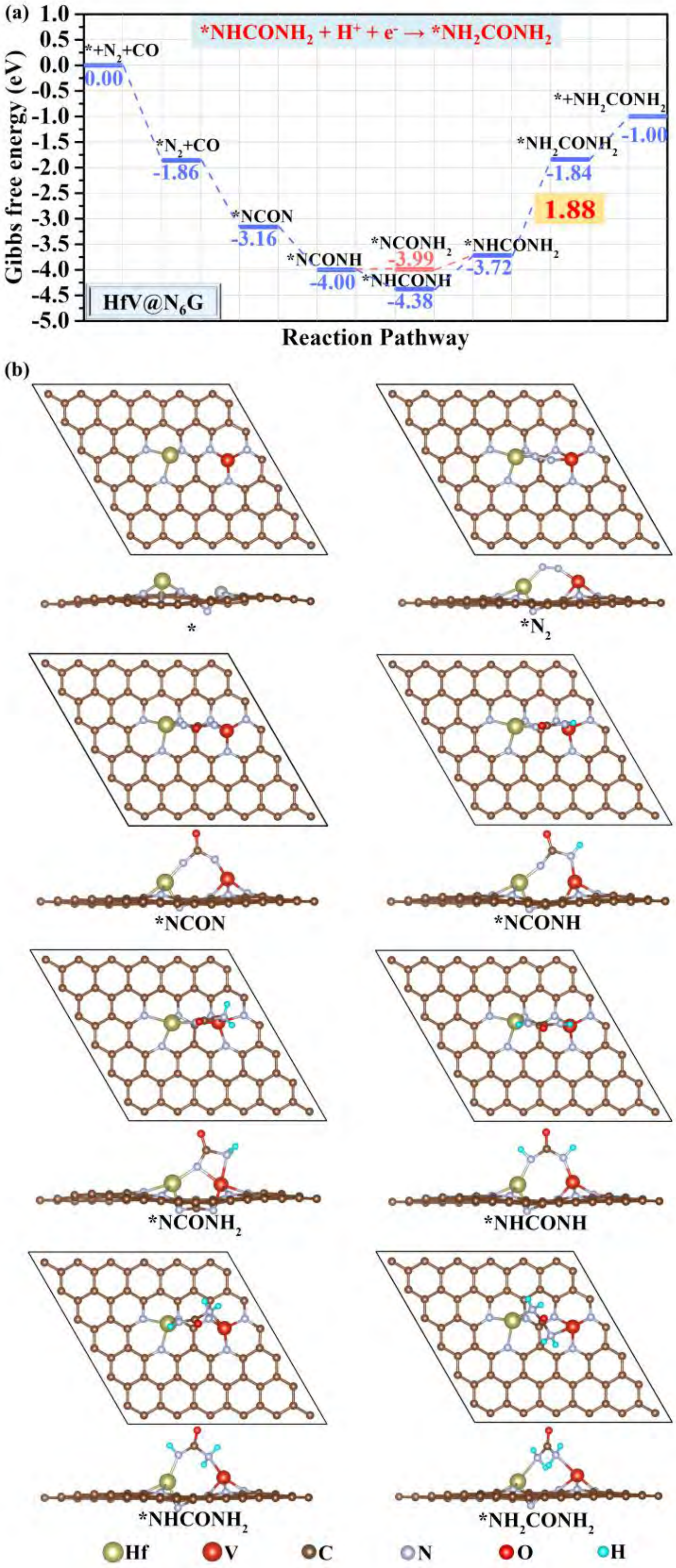

Figure S67. (a) Gibbs free energy diagram for urea production on the HfV@N$_6$G system. (b) Optimized structures of various intermediates along the hydrogenation pathway of urea production on the HfV@N$_6$G system.

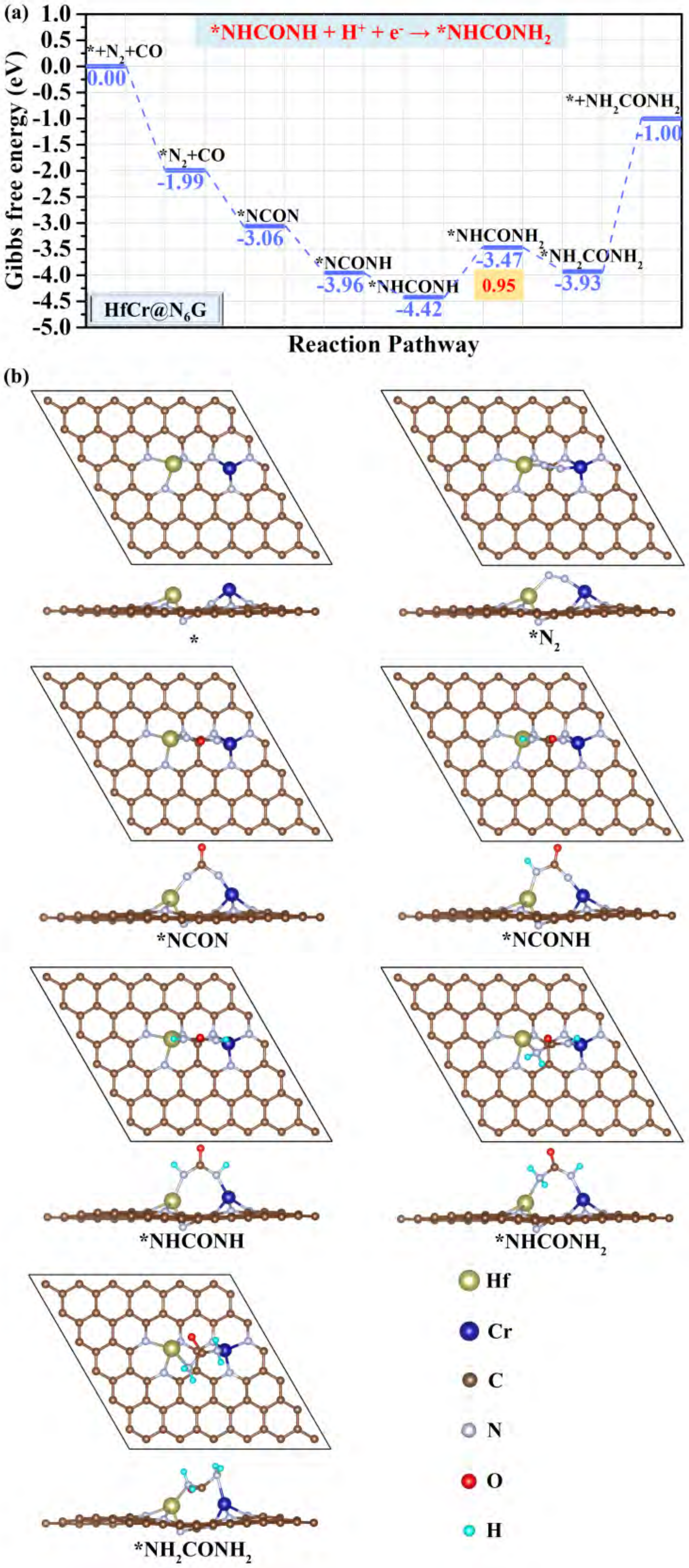

Figure S68. (a) Gibbs free energy diagram for urea production on the HfCr@N_6G system. (b) Optimized structures of various intermediates along the hydrogenation pathway of urea production on the HfCr@N_6G system.

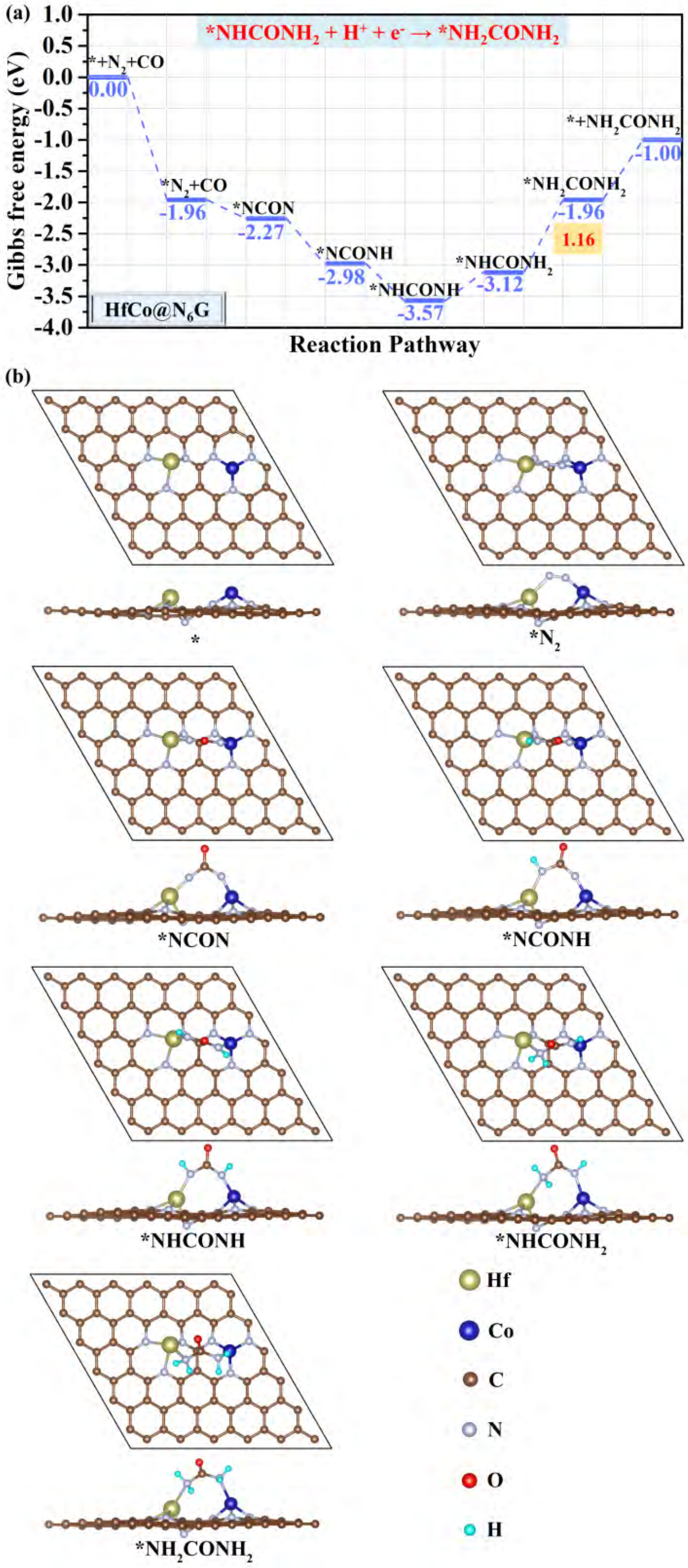

Figure S69. (a) Gibbs free energy diagram for urea production on the HfCo@N_6G system. (b) Optimized structures of various intermediates along the hydrogenation pathway of urea production on the HfCo@N_6G system.

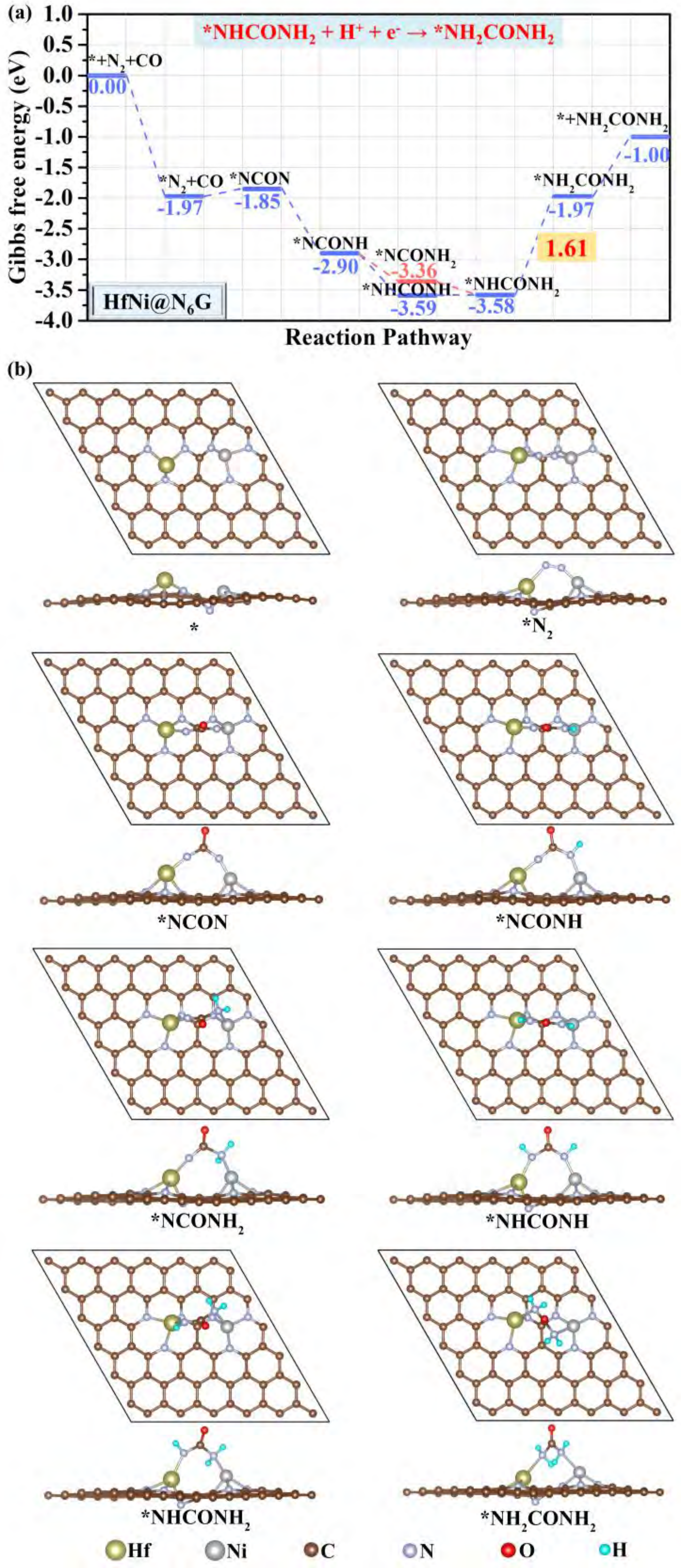

Figure S70. (a) Gibbs free energy diagram for urea production on the HfNi@N_6G system. (b) Optimized structures of various intermediates along the hydrogenation pathway of urea production on the HfNi@N_6G system.

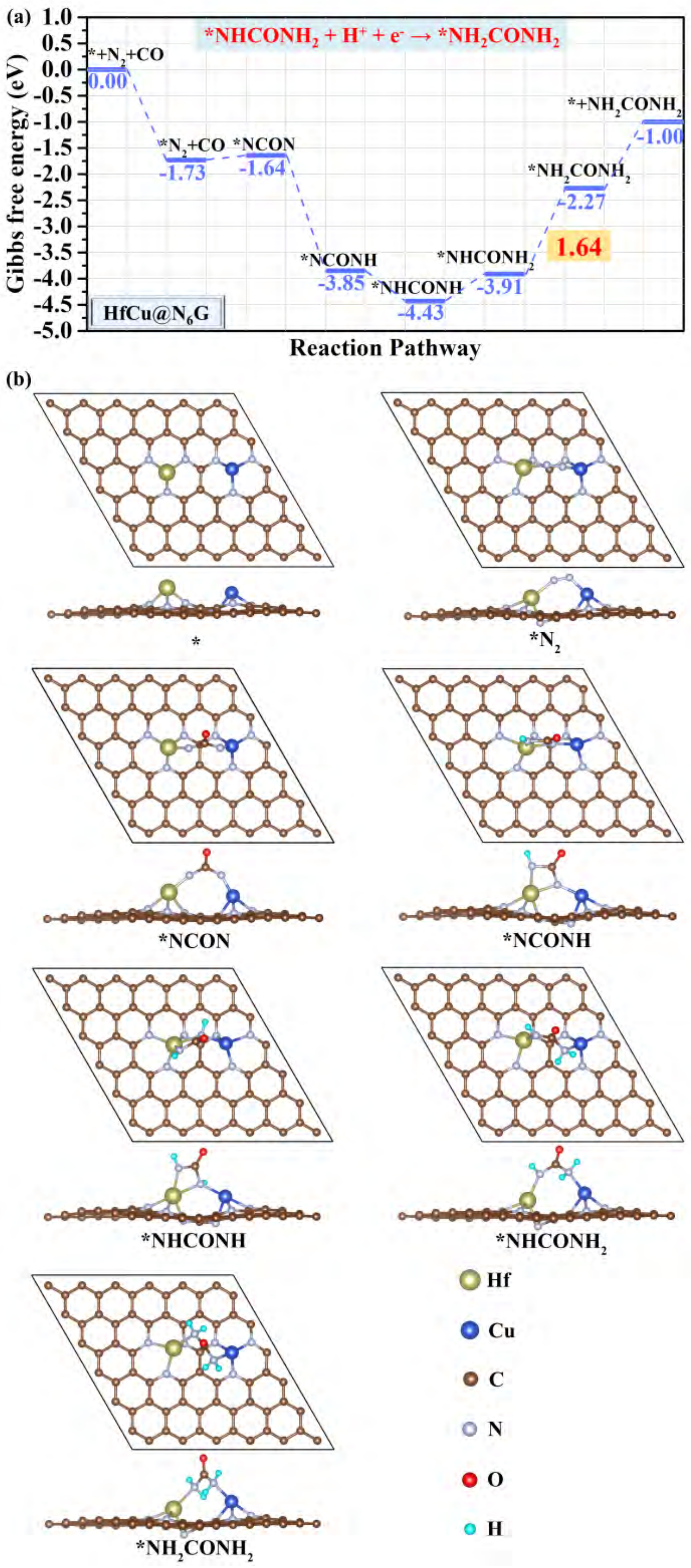

Figure S71. (a) Gibbs free energy diagram for urea production on the HfCu@N_6G system. (b) Optimized structures of various intermediates along the hydrogenation pathway of urea production on the HfCu@N_6G system.

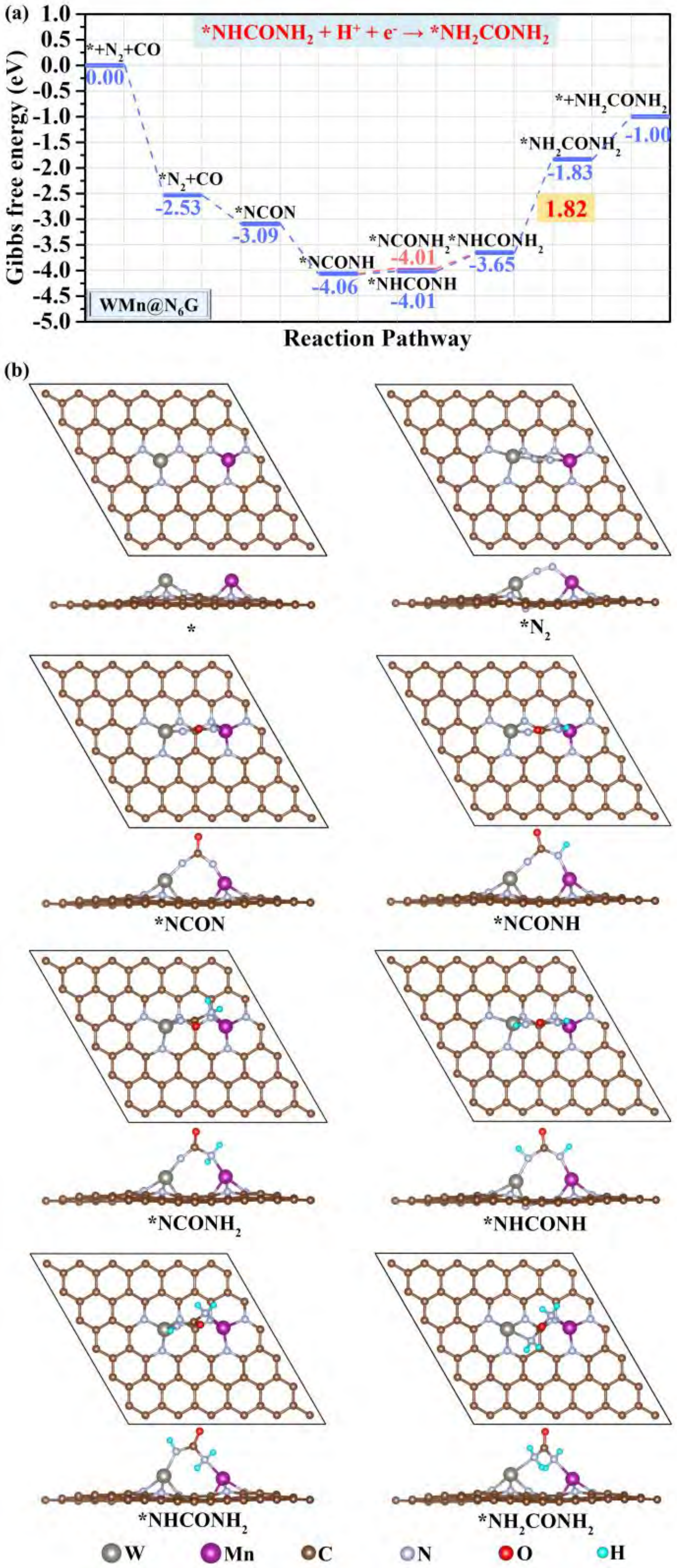

Figure S72. (a) Gibbs free energy diagram for urea production on the WMn@N_6G system. (b) Optimized structures of various intermediates along the hydrogenation pathway of urea production on the WMn@N_6G system.

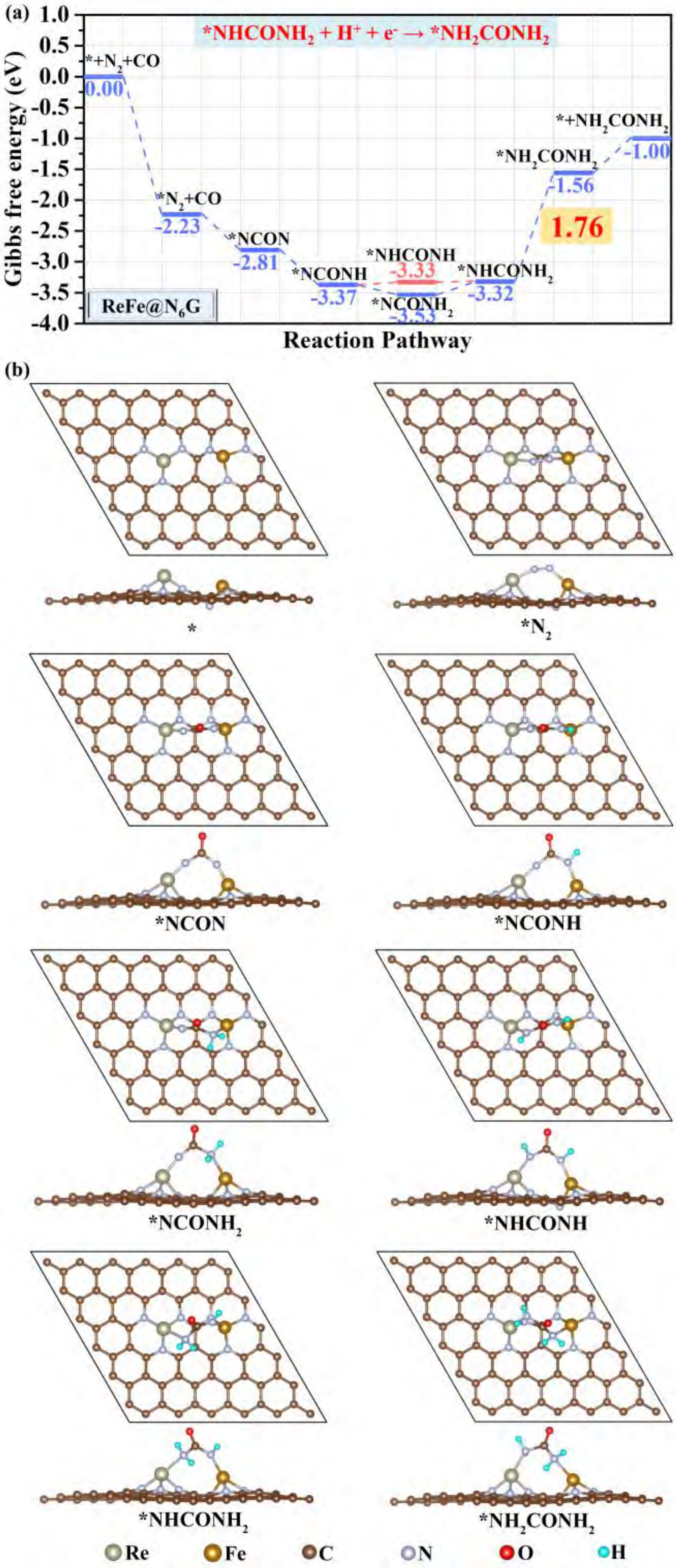

Figure S73. (a) Gibbs free energy diagram for urea production on the ReFe@N_6G system. (b) Optimized structures of various intermediates along the hydrogenation pathway of urea production on the ReFe@N_6G system.

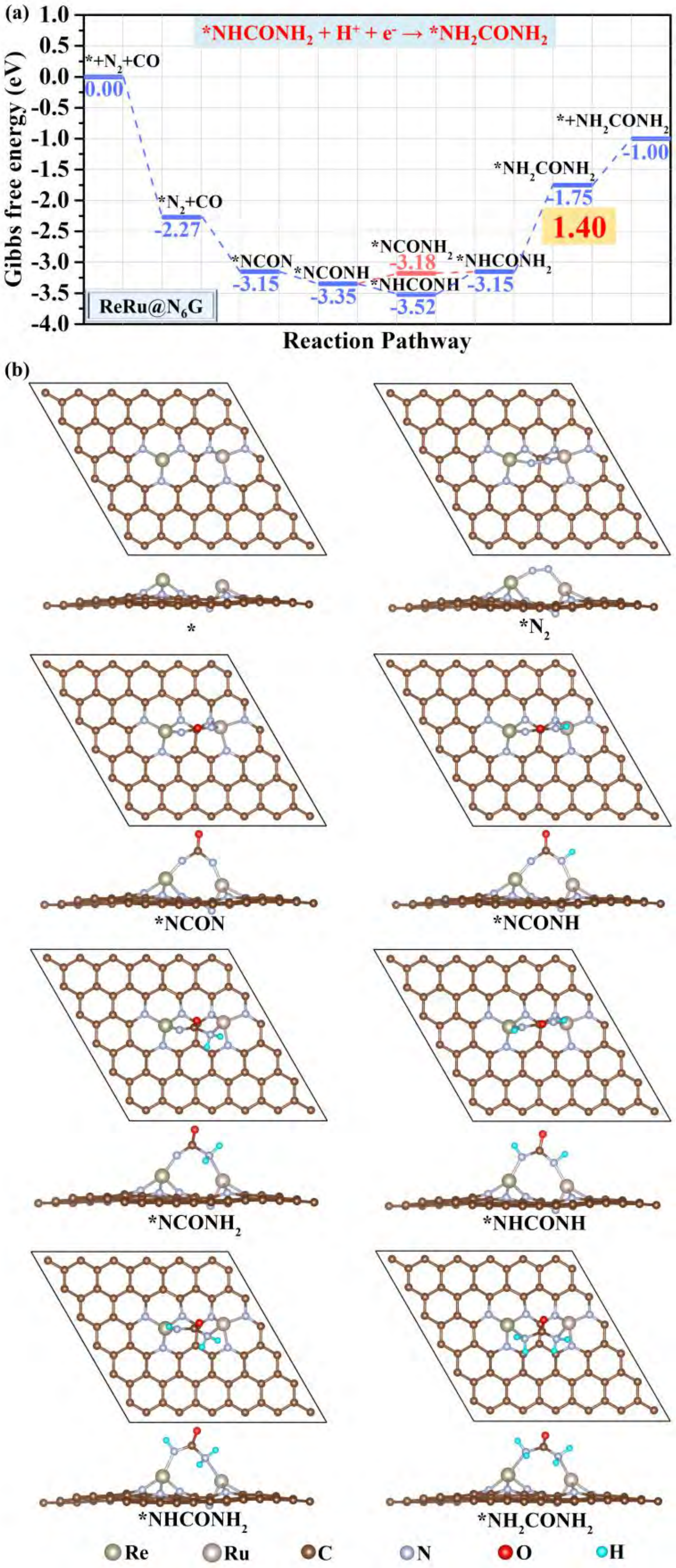

Figure S74. (a) Gibbs free energy diagram for urea production on the ReRu@N_6G system. (b) Optimized structures of various intermediates along the hydrogenation pathway of urea production on the ReRu@N_6G system.

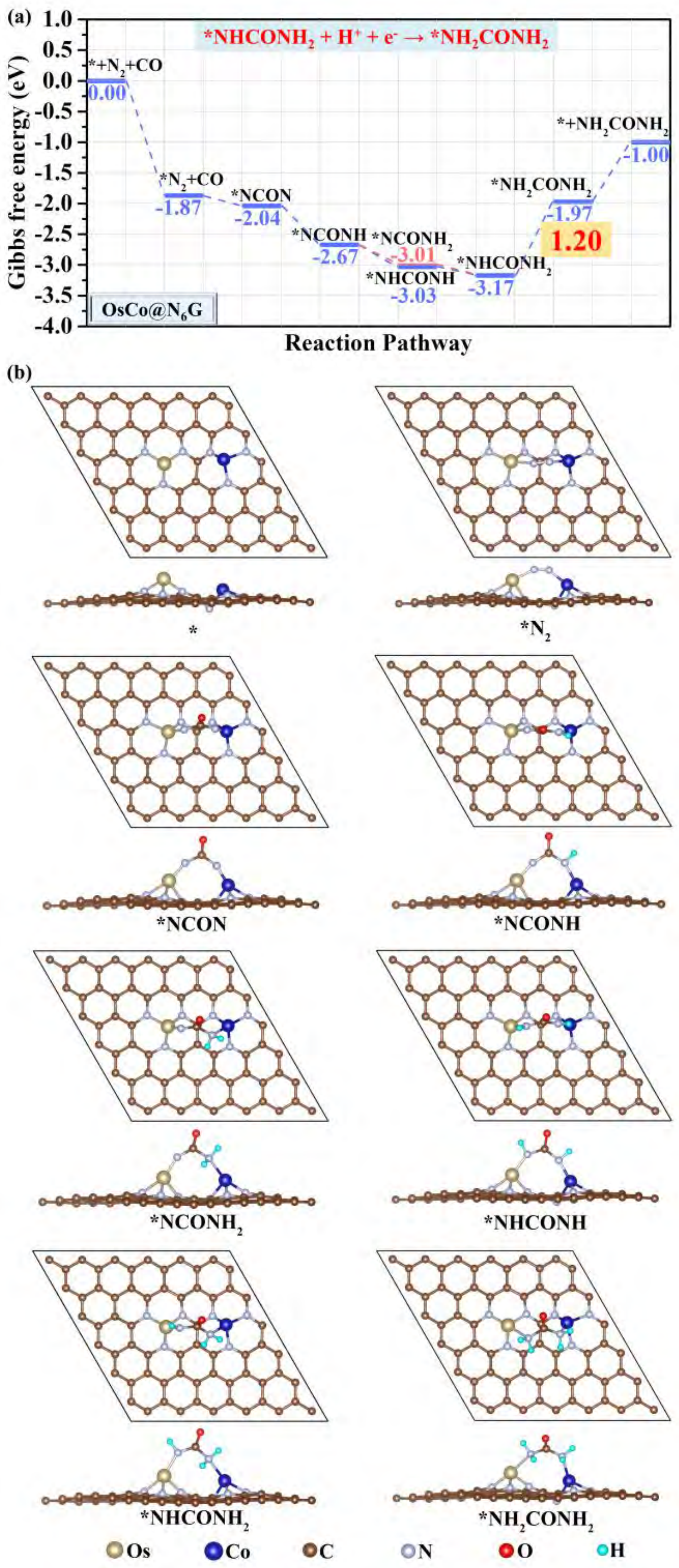

Figure S75. (a) Gibbs free energy diagram for urea production on the OsCo@N_6G system. (b) Optimized structures of various intermediates along the hydrogenation pathway of urea production on the OsCo@N_6G system.

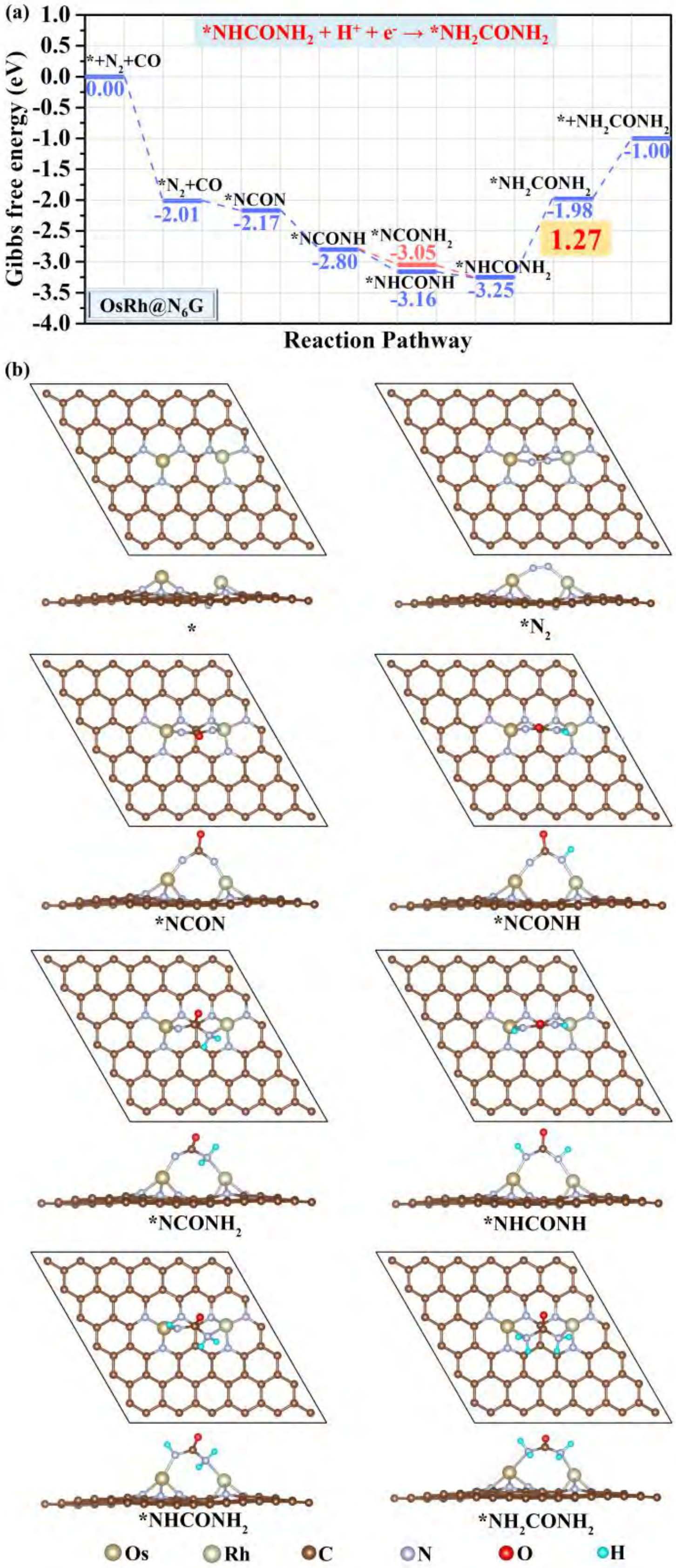

Figure S76. (a) Gibbs free energy diagram for urea production on the OsRh@N_6G system. (b) Optimized structures of various intermediates along the hydrogenation pathway of urea production on the OsRh@N_6G system.

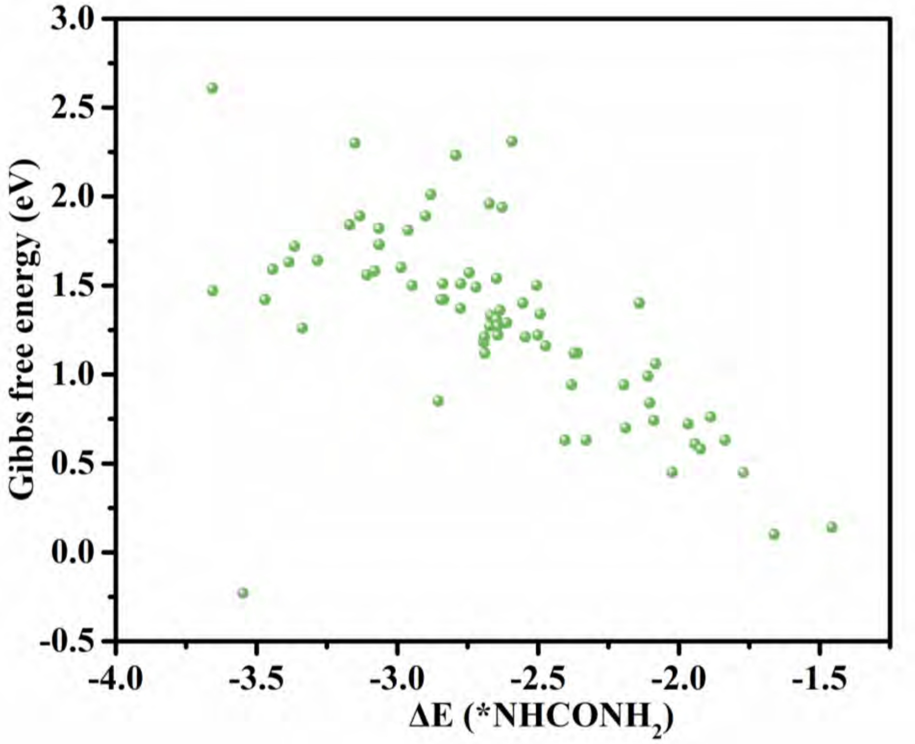

Figure S77. Calculated Gibbs free energy of $*NHCONH_2 + H^+ + e^- \rightarrow *NH_2CONH_2$ versus the adsorption energy of $*NHCONH_2$ ($\Delta E(*NHCONH_2)$). $\Delta E(*NHCONH_2) = E(*NHCONH_2) - E* - E(N_2CONH_2) + 1/2E(H_2)$.

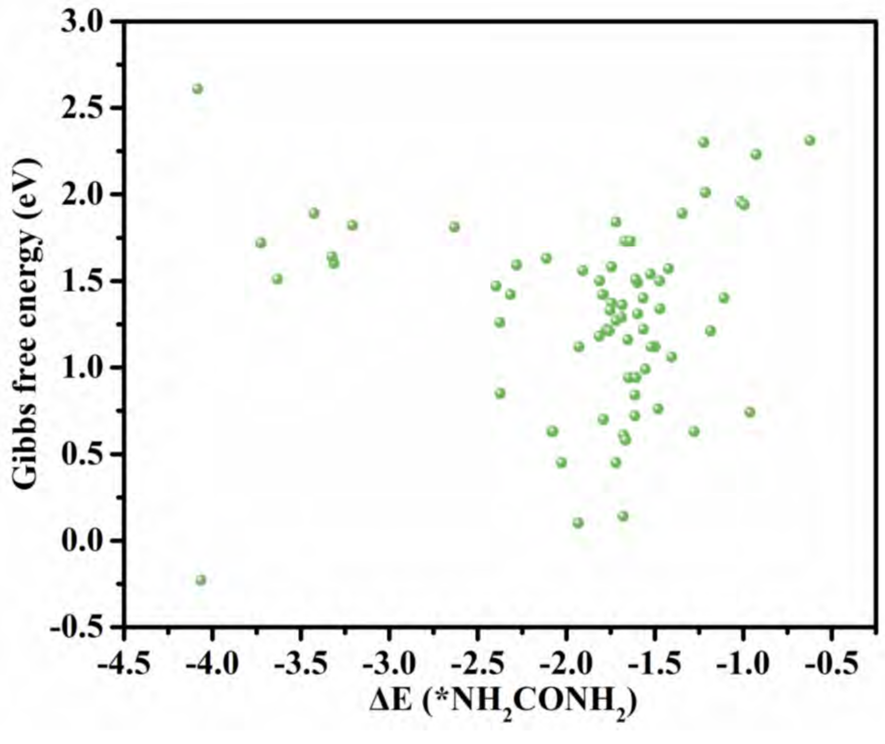

Figure S78. Calculated Gibbs free energy of $*NHCONH_2 + H^+ + e^- \rightarrow *NH_2CONH_2$ versus the adsorption energy of $*NHCONH_2$ ($\Delta E(*NHCONH_2)$). $\Delta E(*NH_2CONH_2) = E(*NH_2CONH_2) - E* - E(N_2CONH_2)$.

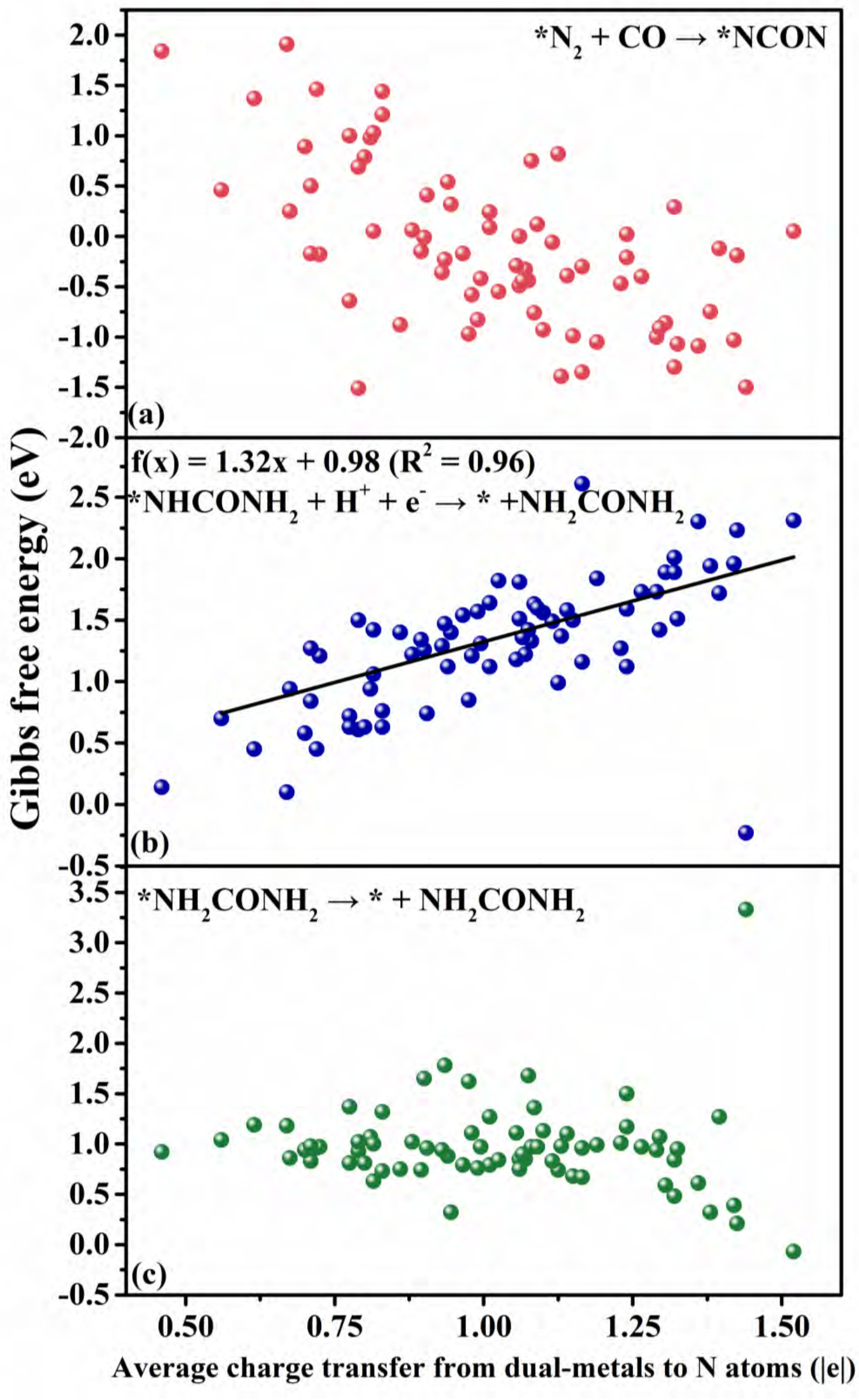

Figure S79. (a) Computed Gibbs free energy of $*N_2 + CO \rightarrow *NCON$ versus the average charge transfer from dual-metals to N atoms. (b) Calculated Gibbs free energy of $*NHCONH_2 + H^+ + e^- \rightarrow *NH_2CONH_2$ as a function of the average charge transfer from dual-metals to N atoms. (c) Calculated Gibbs free energy of $*NHCONH_2 + H^+ + e^- \rightarrow *NH_2CONH_2$ versus the average charge transfer from dual-metals to N atoms.

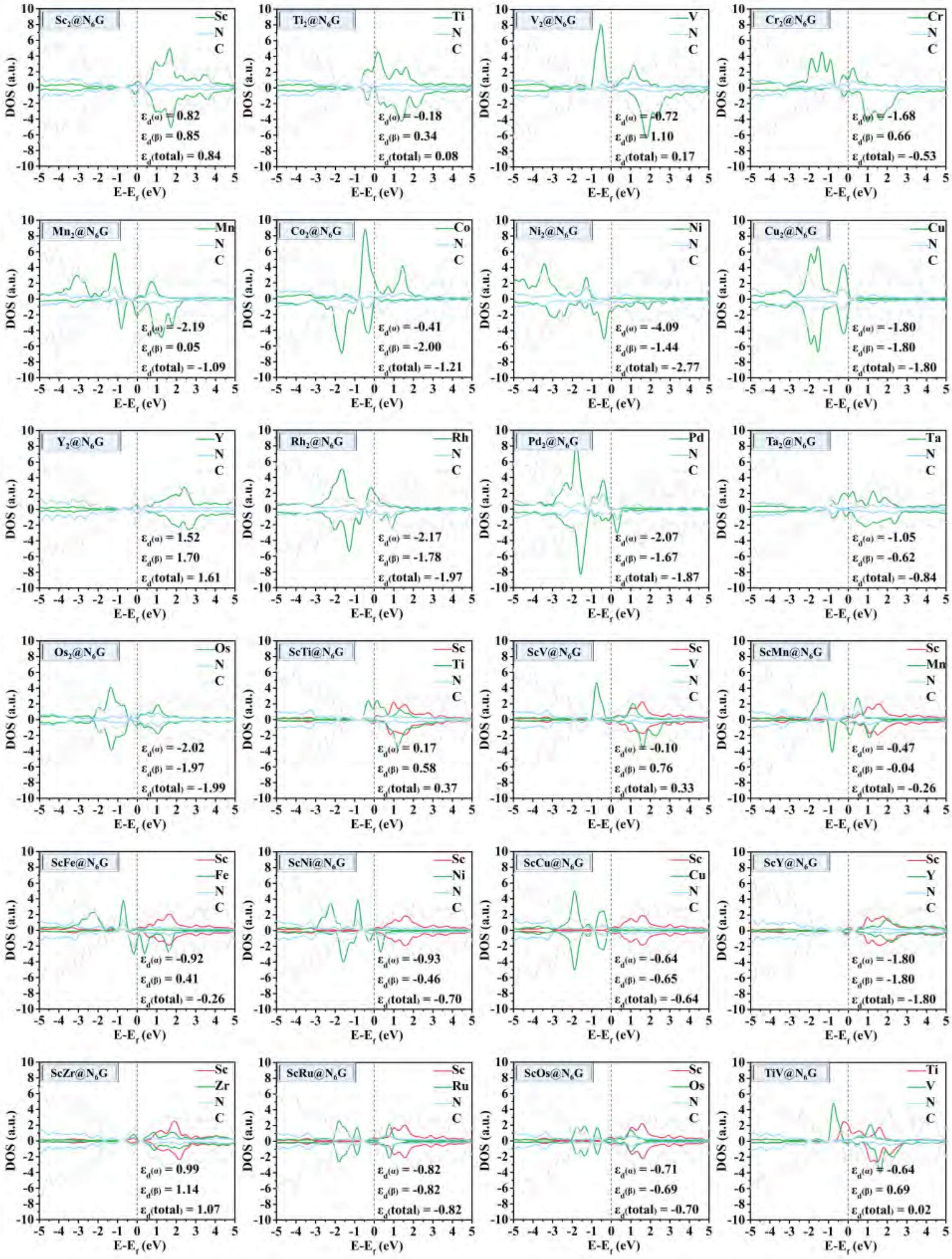

Figure S80. (a) Computed partial density of states (PDOS) of the 72 stable systems.

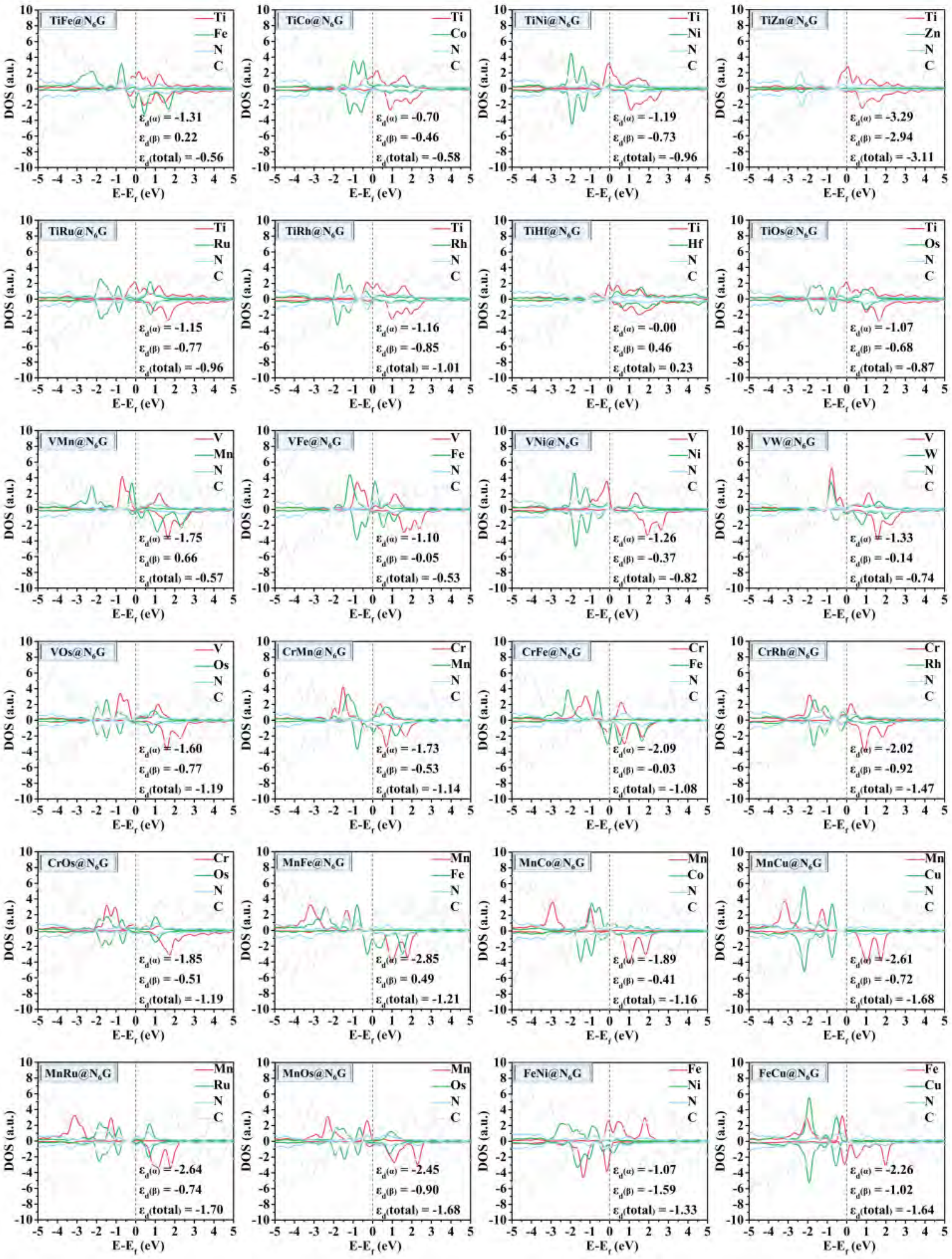

Figure S80. (b) Computed partial density of states (PDOS) of 72 stable systems.

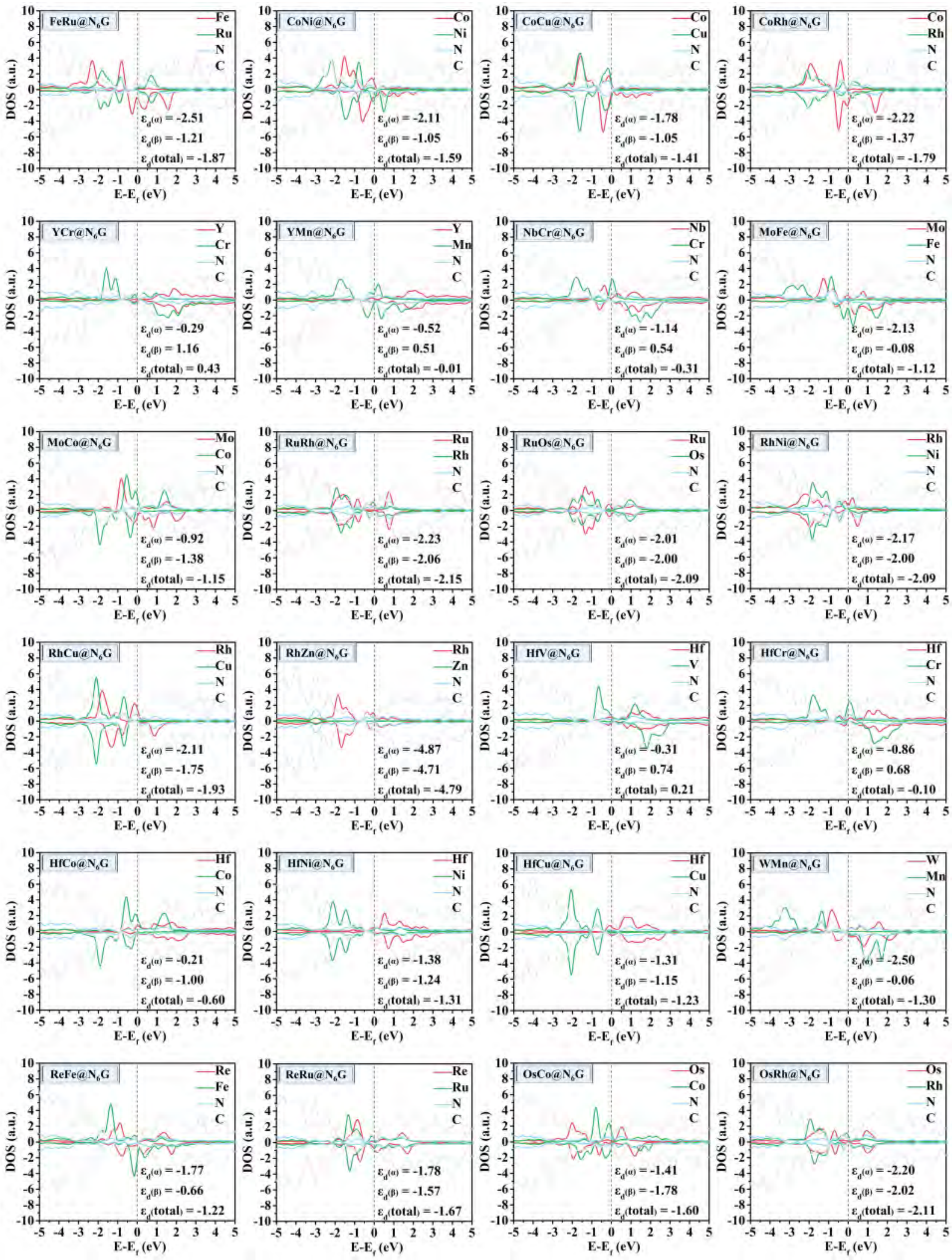

Figure S80. (c) Computed partial density of states (PDOS) of 72 stable systems.

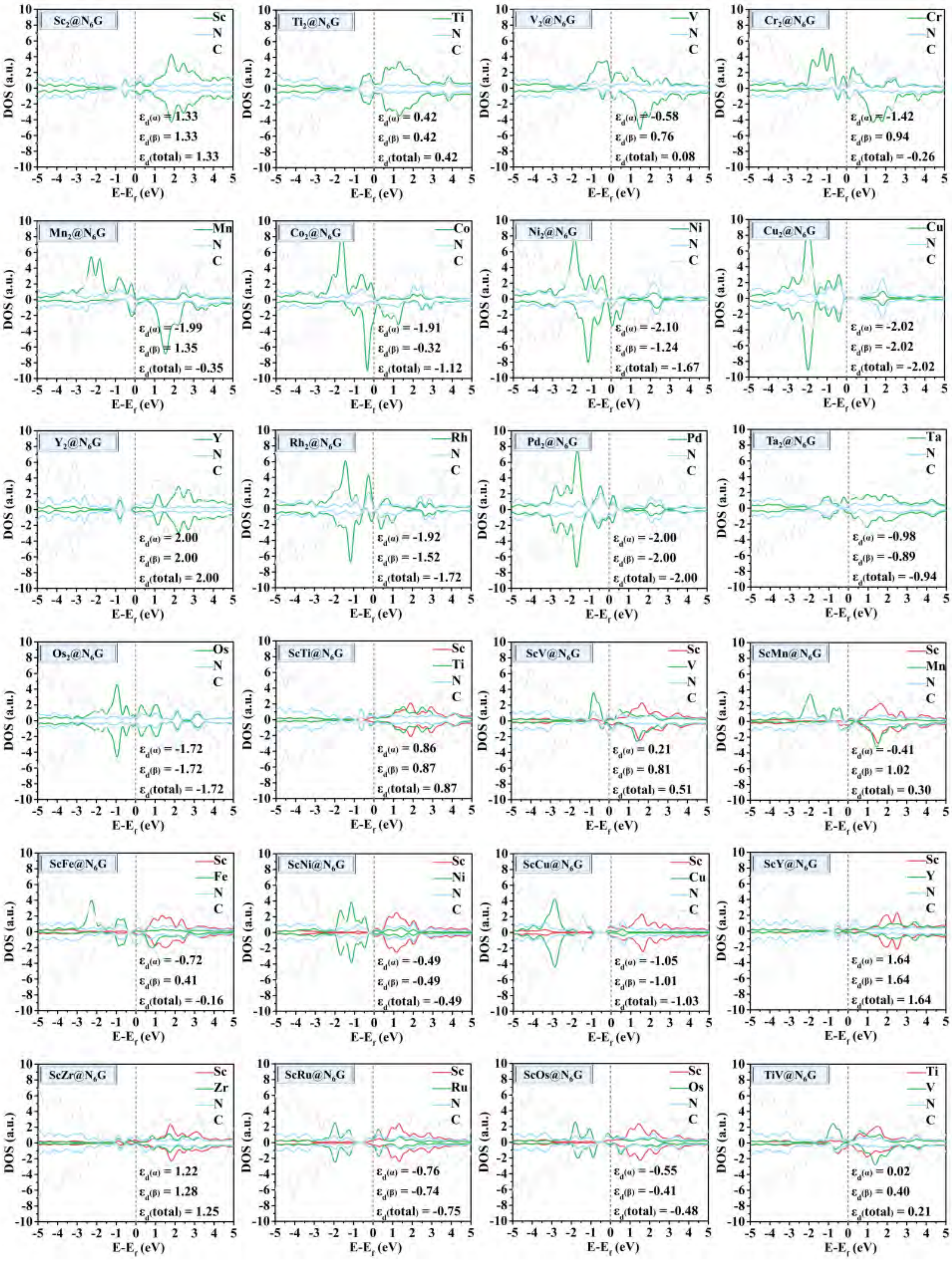

Figure S81. (a) Computed partial density of states (PDOS) of 72 stable systems with the $*N_2$ intermediate.

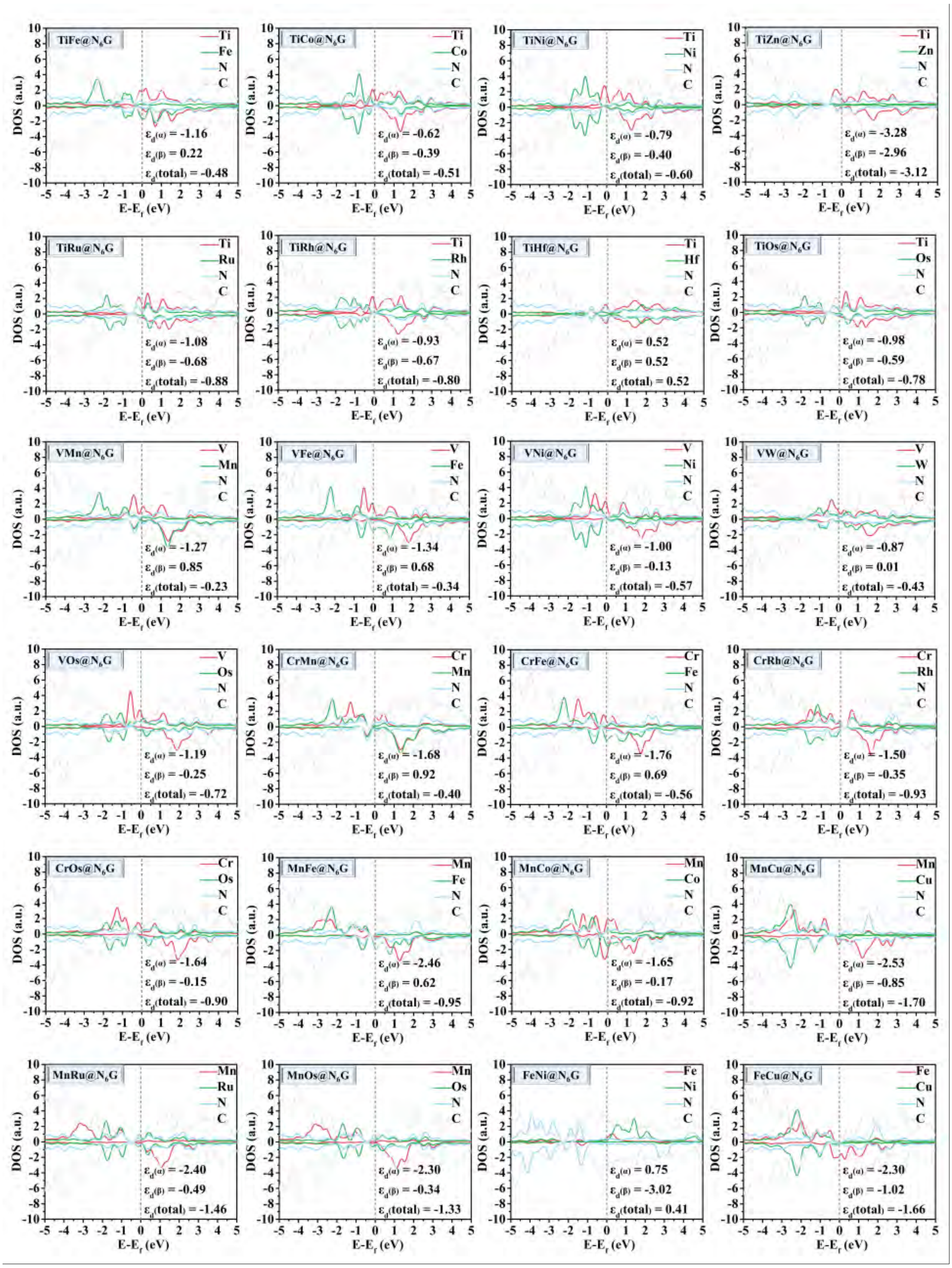

Figure S81. (b) Computed partial density of states (PDOS) of 72 stable systems with the $*N_2$ intermediate.

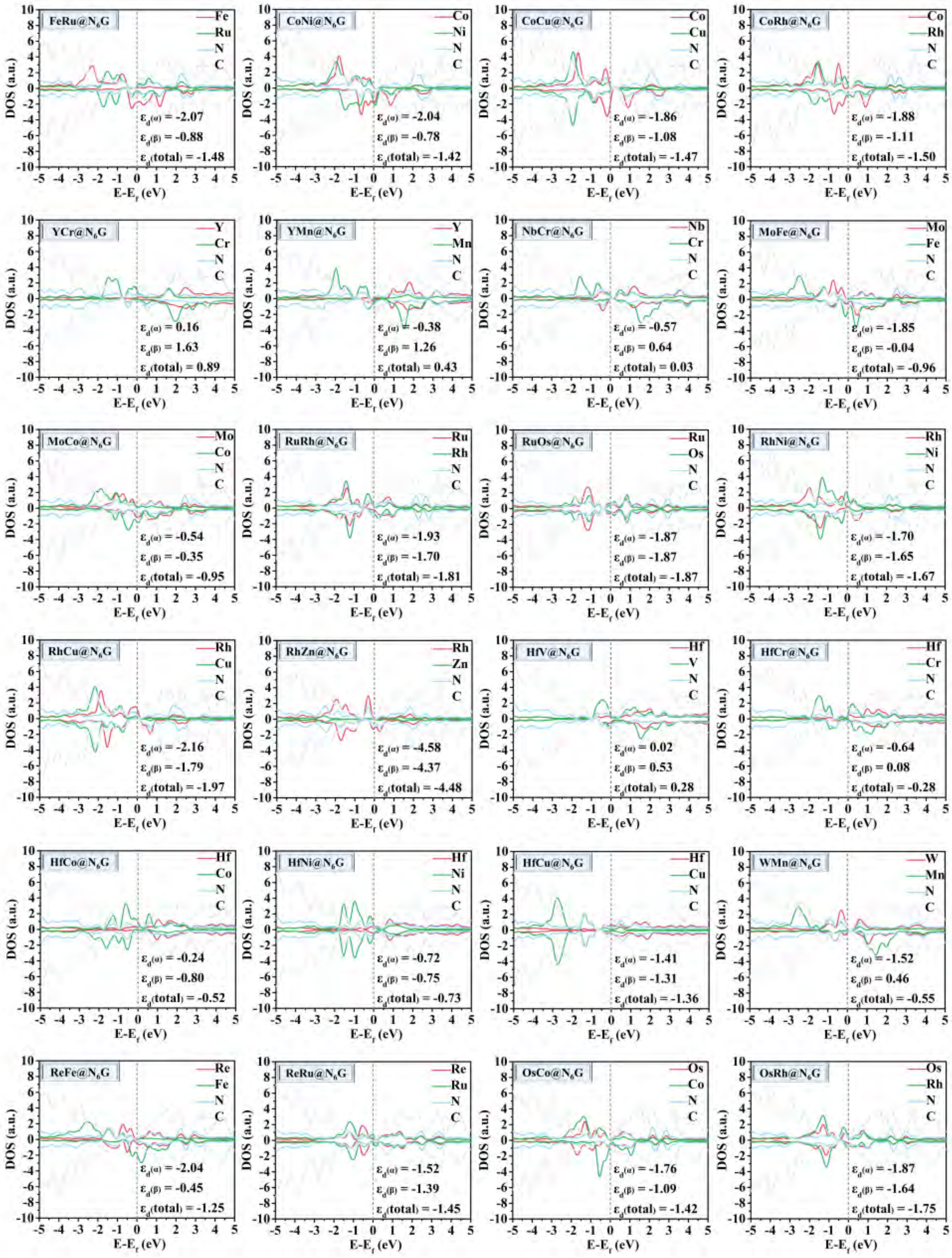

Figure S81. (c) Computed partial density of states (PDOS) of 72 stable systems with the *N_2 intermediate.

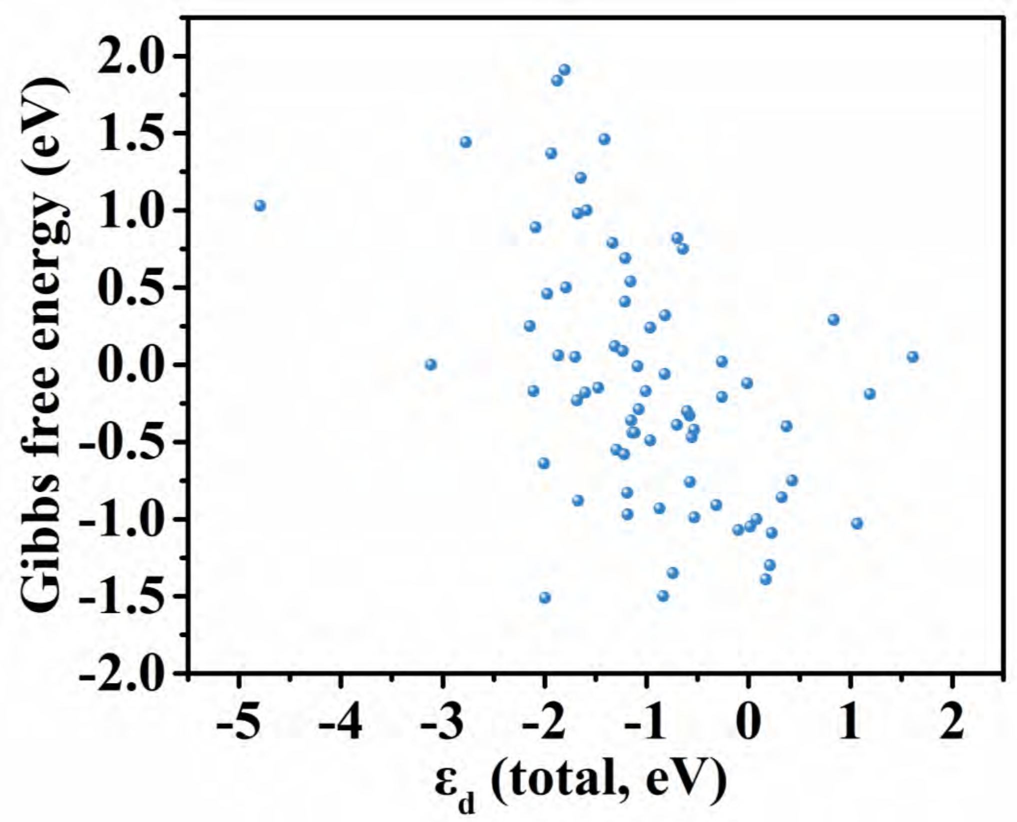

Figure S82. Calculated Gibbs free energy of $*N_2$ + CO → *NCON versus the d-band center of the total metal orbitals (ε_d(total)).

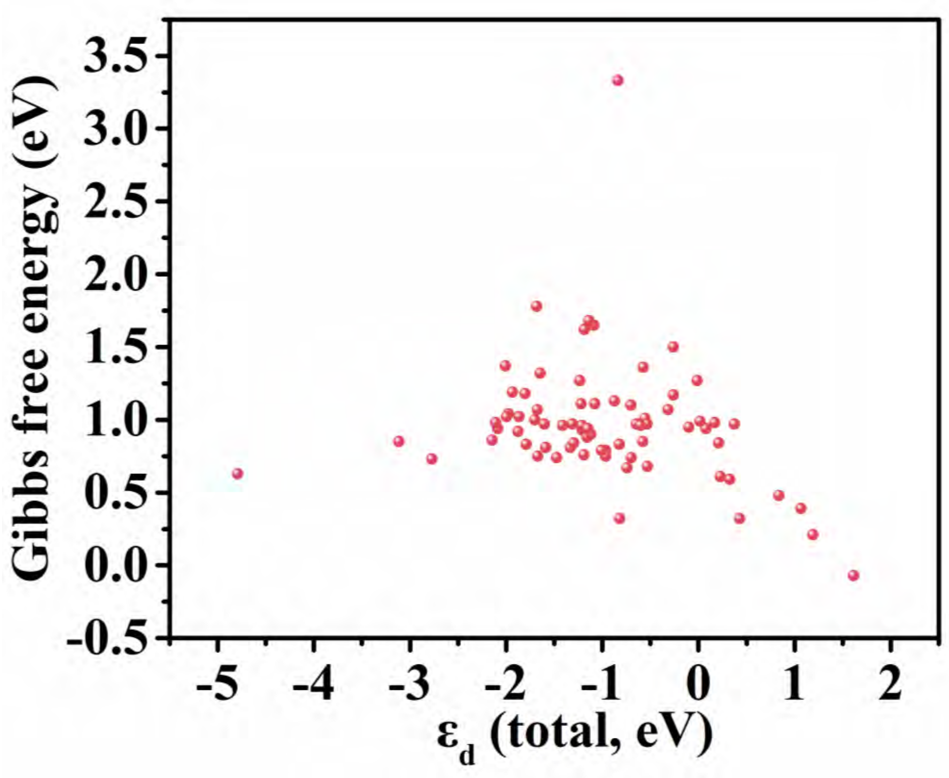

Figure S83. Calculated Gibbs free energy of $*NH_2CONH_2$ → * + NH_2CONH_2 versus the d-band center of the total metal orbitals (ε_d(total)).

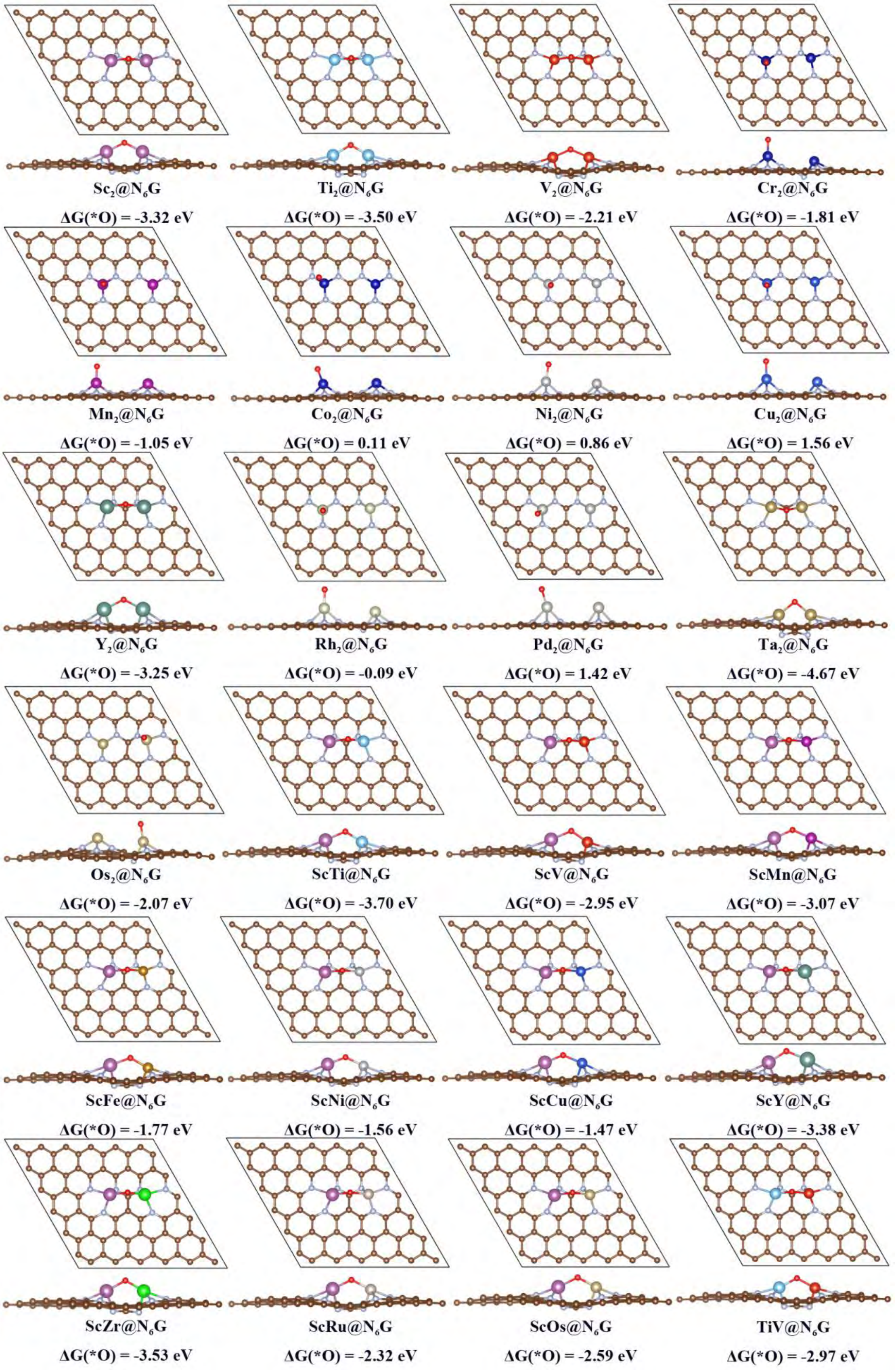

Figure S84. (a) Optimized structures and the corresponding adsorption free energies of oxygen atom (ΔG(*O)) on 72 stable systems.

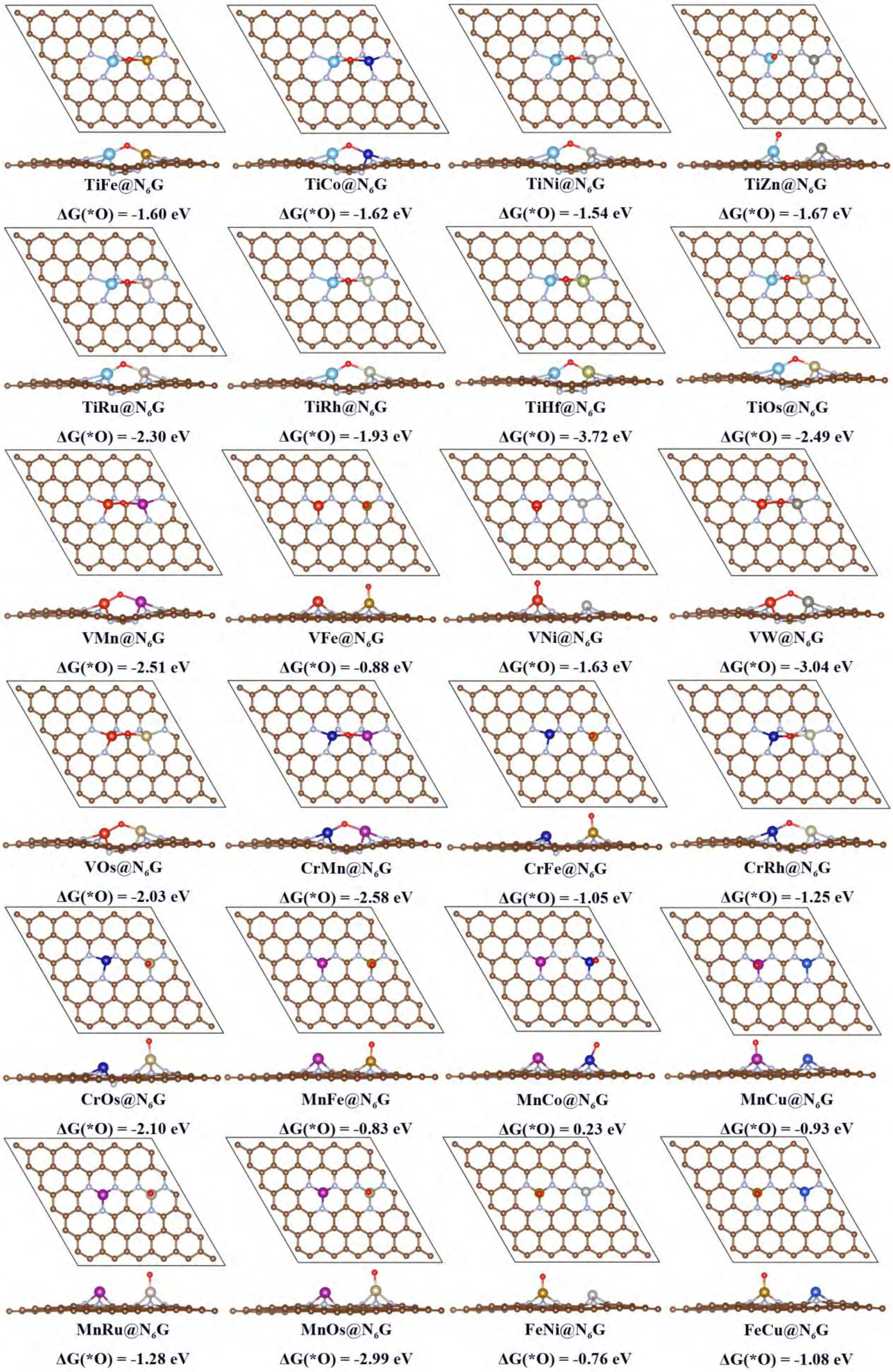

Figure S84. (b) Optimized structures and the corresponding adsorption free energies of oxygen atom (ΔG(*O)) on 72 stable systems.

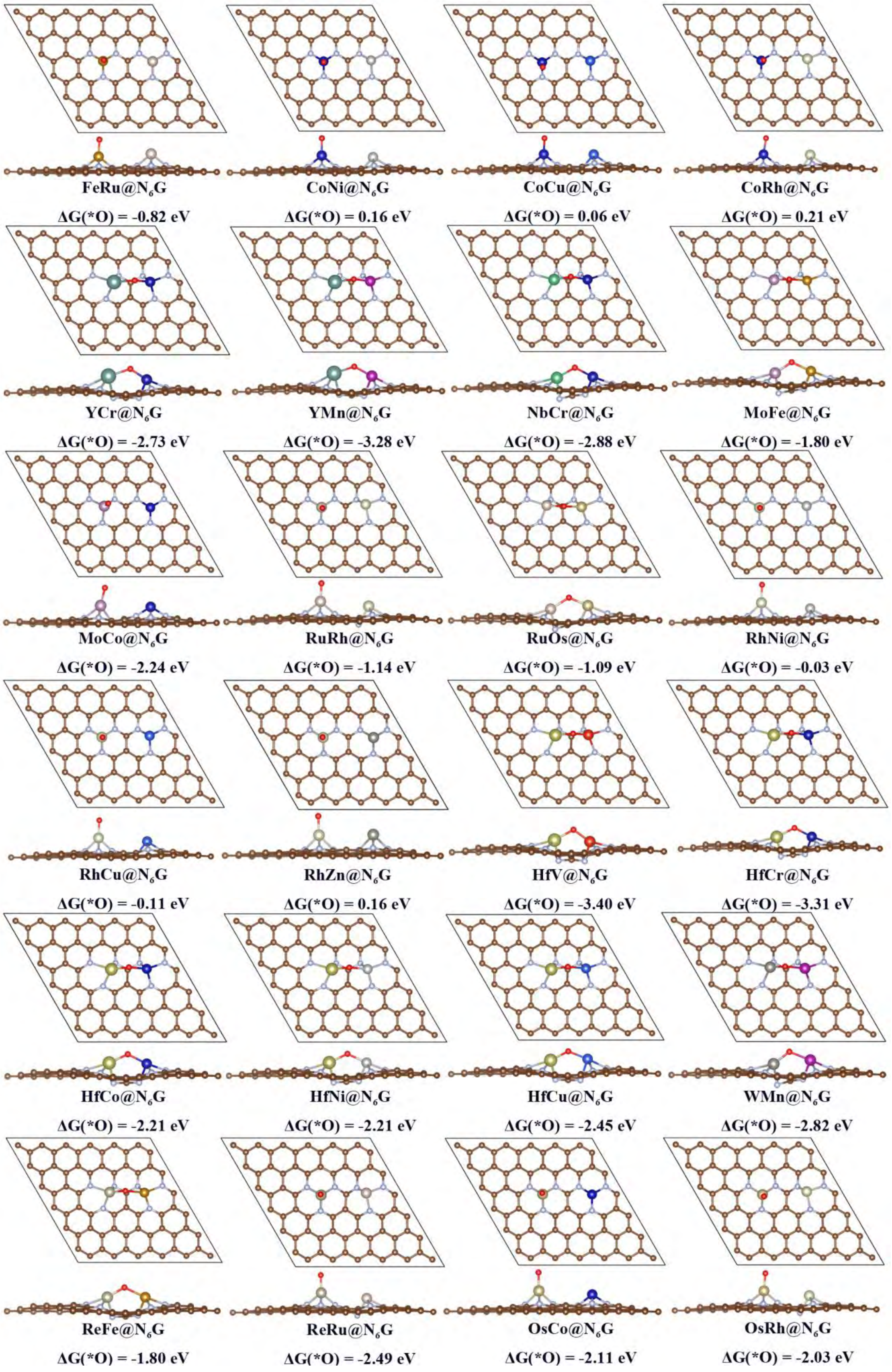

Figure S84. (c) Optimized structures and the corresponding adsorption free energies of oxygen atom (ΔG(*O)) on 72 stable systems.

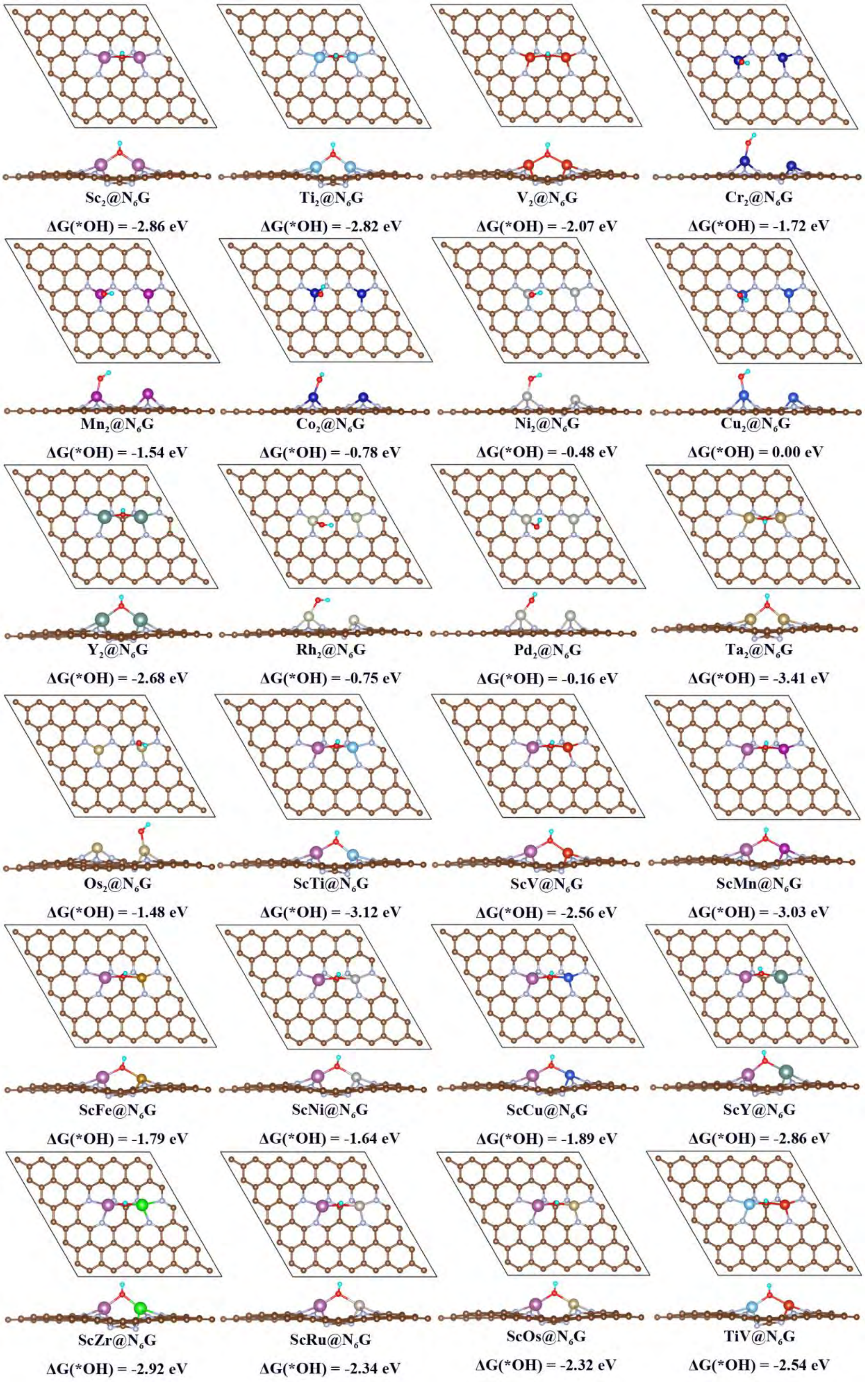

Figure S85. (a) Optimized structures and the corresponding adsorption free energies of hydroxyl (ΔG(*OH)) on 72 stable systems.

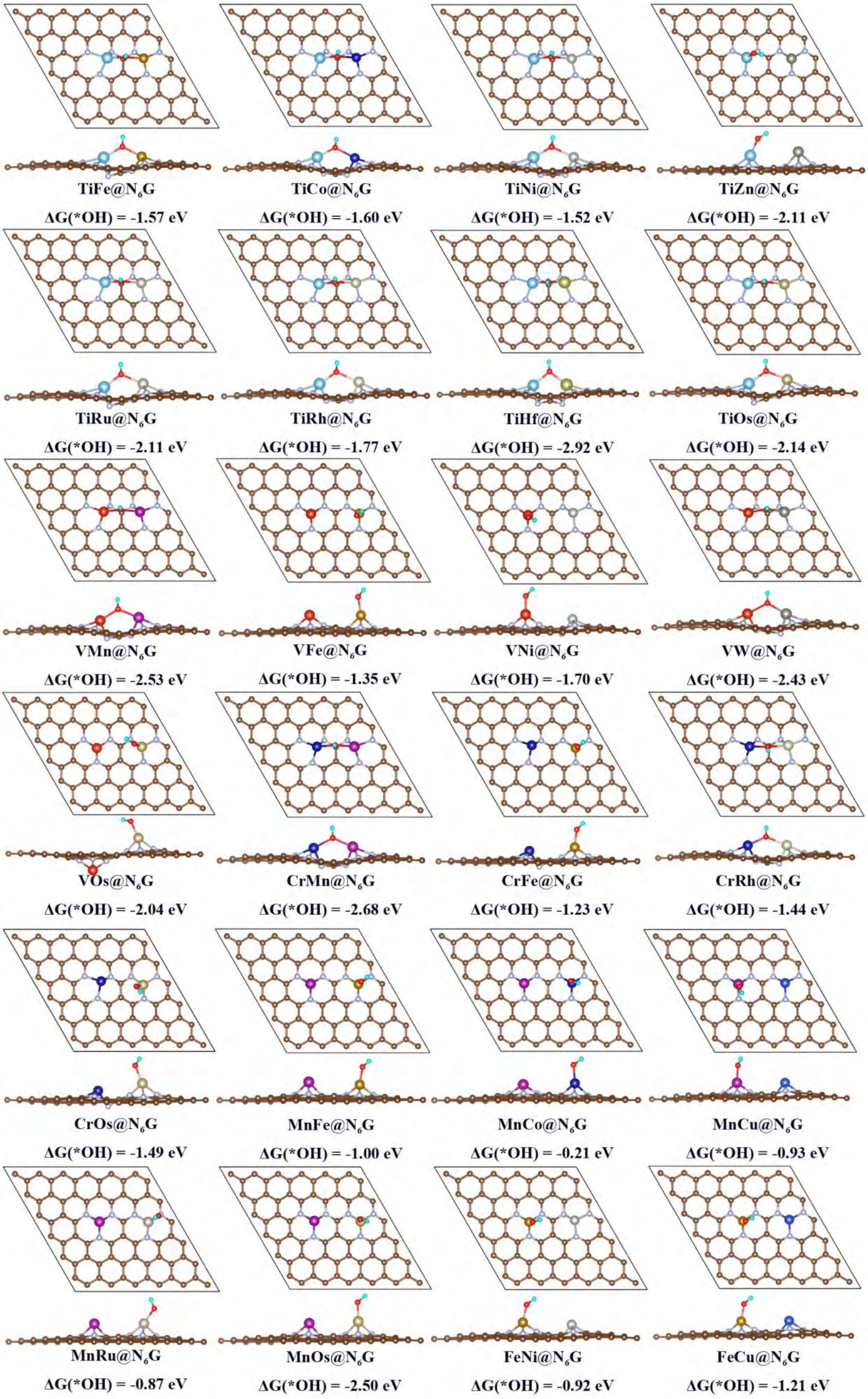

Figure S85. (b) Optimized structures and the corresponding adsorption free energies of hydroxyl (ΔG(*OH)) on 72 stable systems.

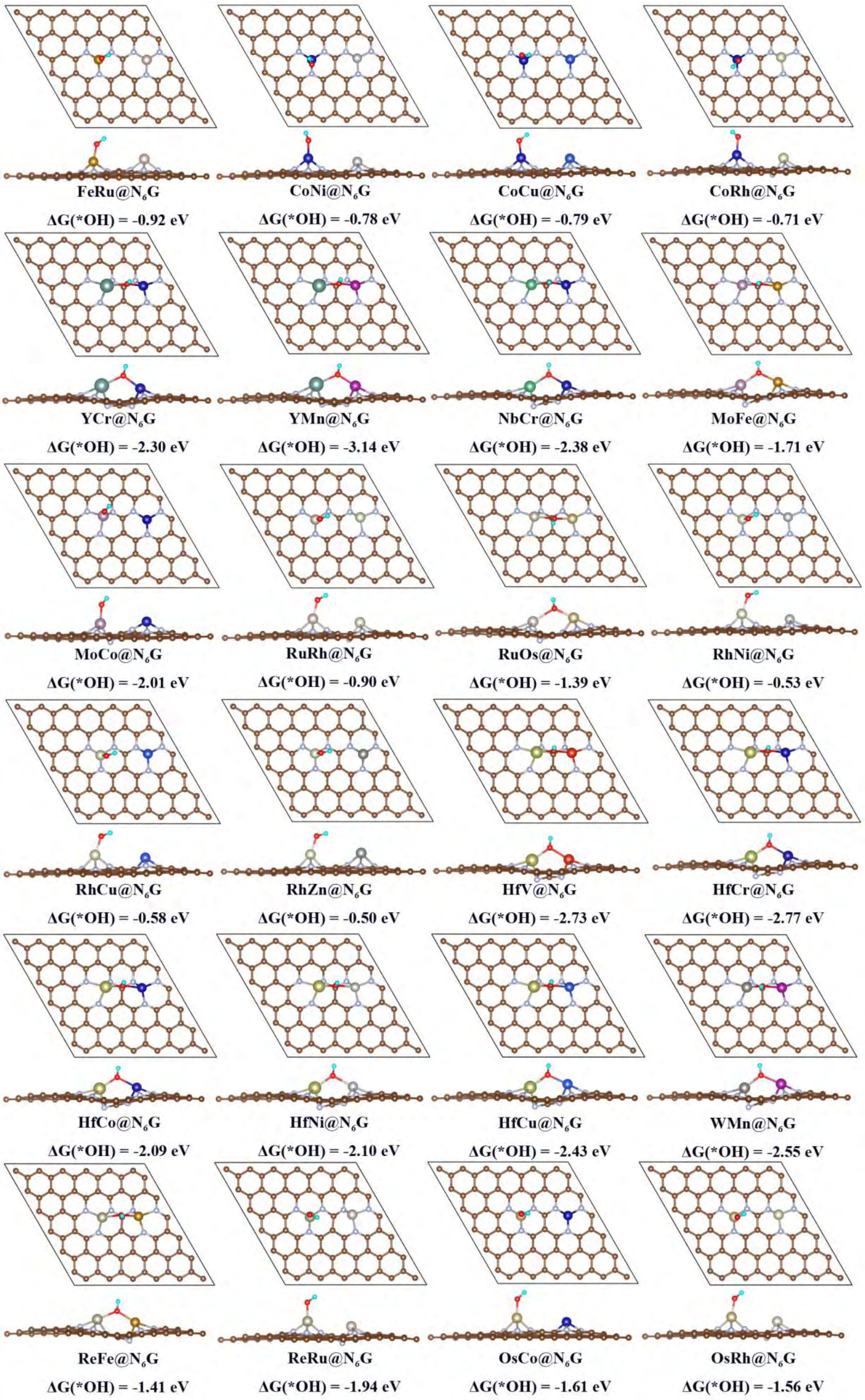

Figure S85. (c) Optimized structures and the corresponding adsorption free energies of hydroxyl (ΔG(*OH)) on 72 stable systems.

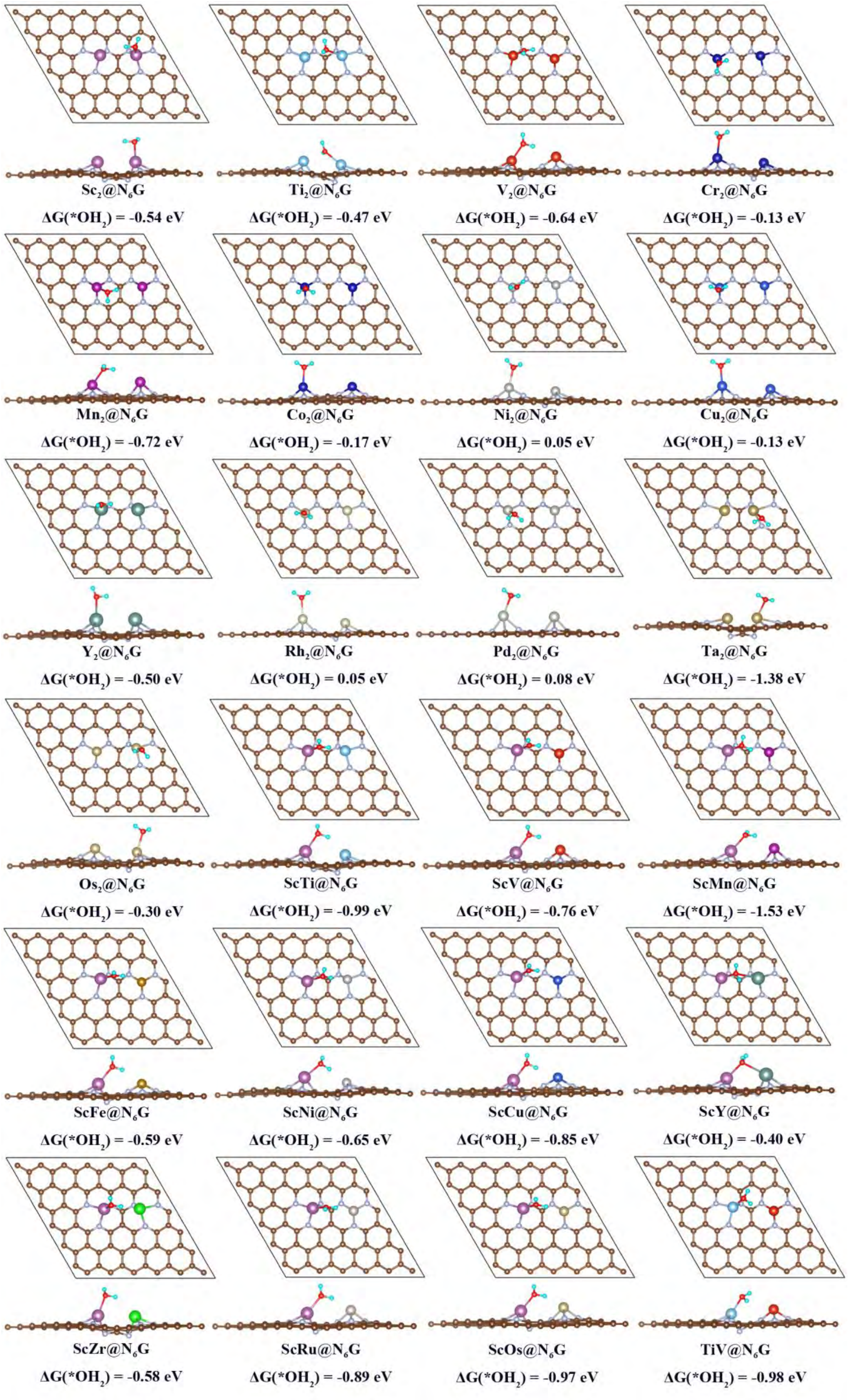

Figure S86. (a) Optimized structures and the corresponding adsorption free energies of water molecule ($\Delta G(*OH_2)$) on 72 stable systems.

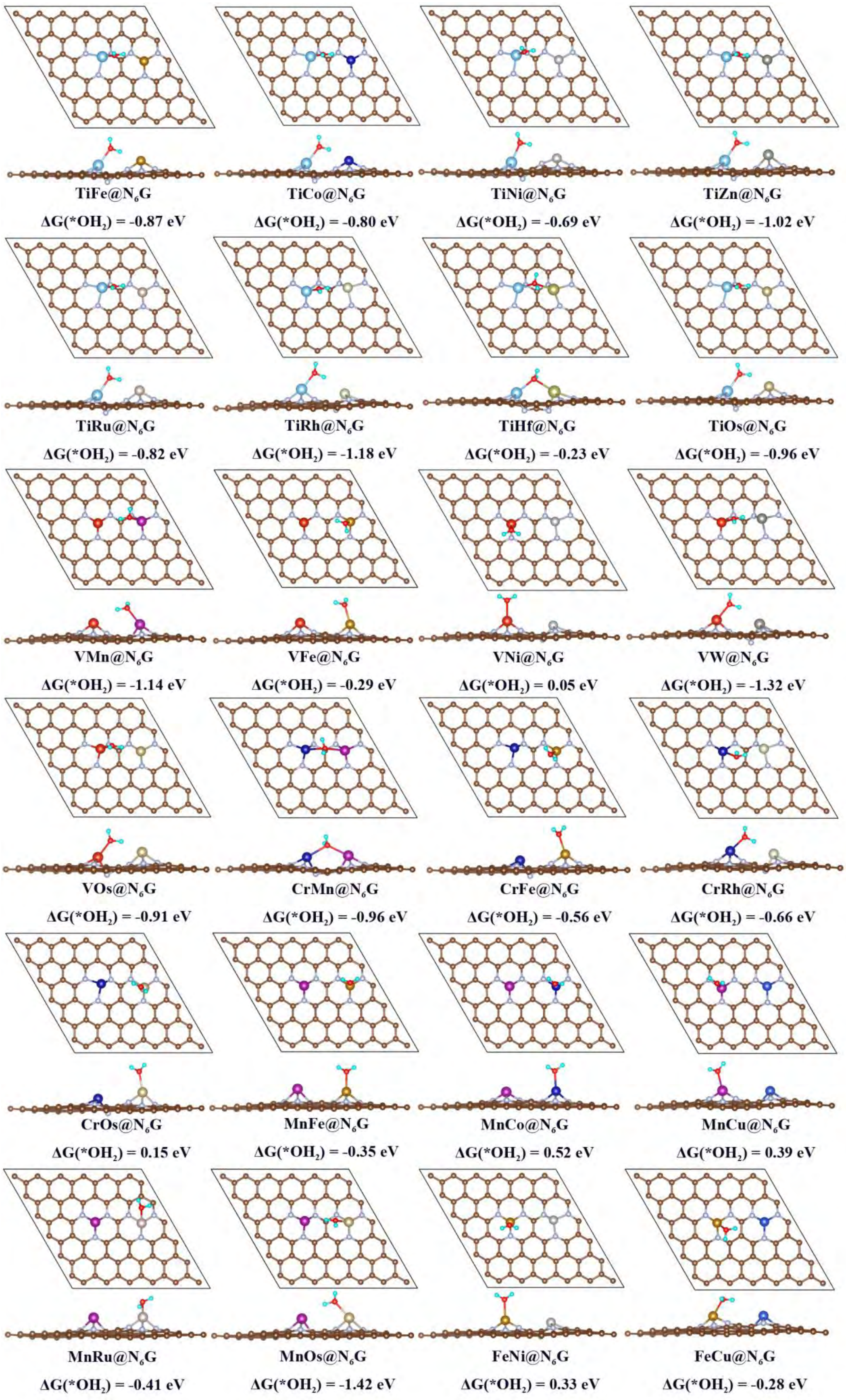

Figure S86. (b) Optimized structures and the corresponding adsorption free energies of water molecule (ΔG(*OH_2)) on 72 stable systems.

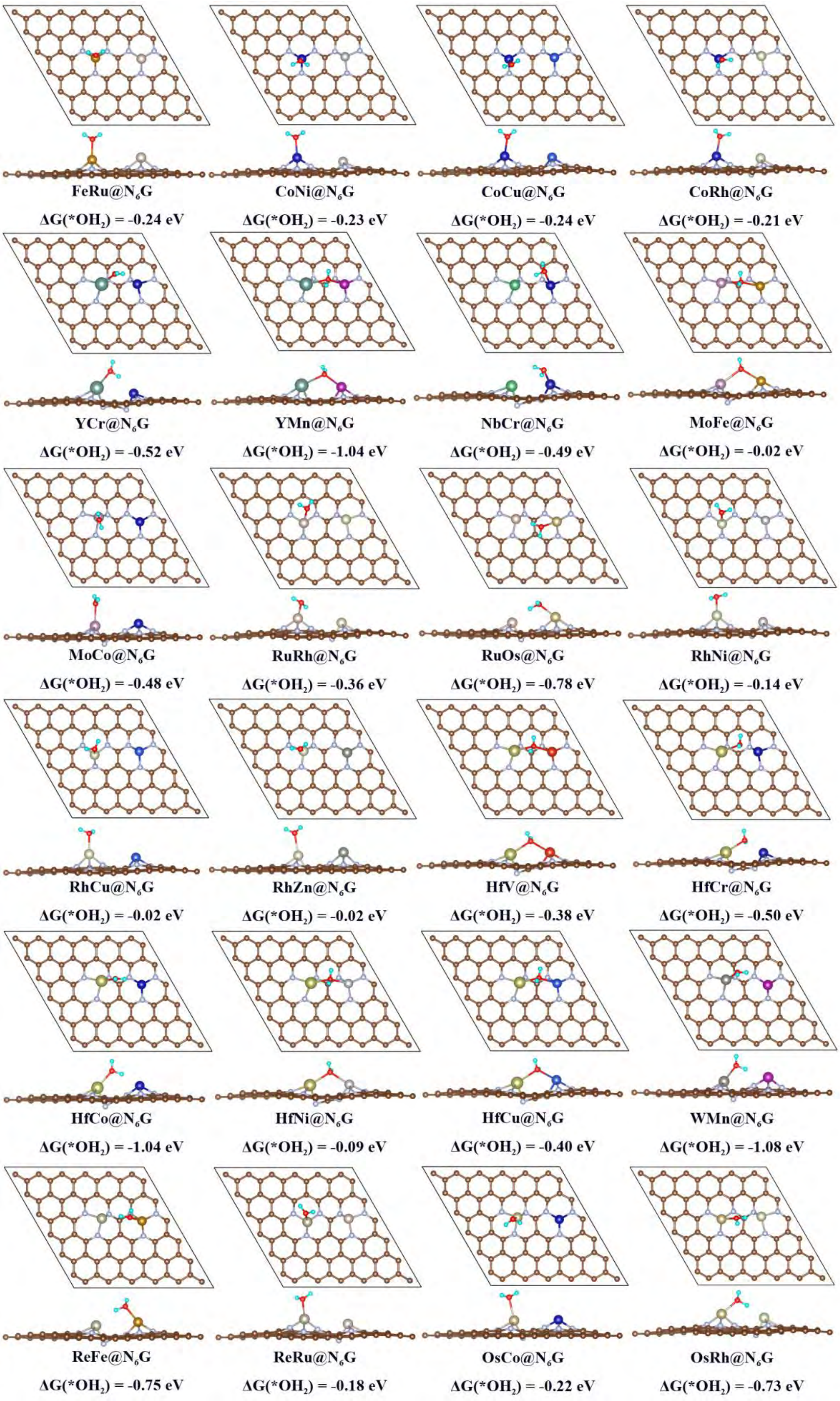

Figure S86. (c) Optimized structures and the corresponding adsorption free energies of water molecule ($\Delta G(*OH_2)$) on 72 stable systems.

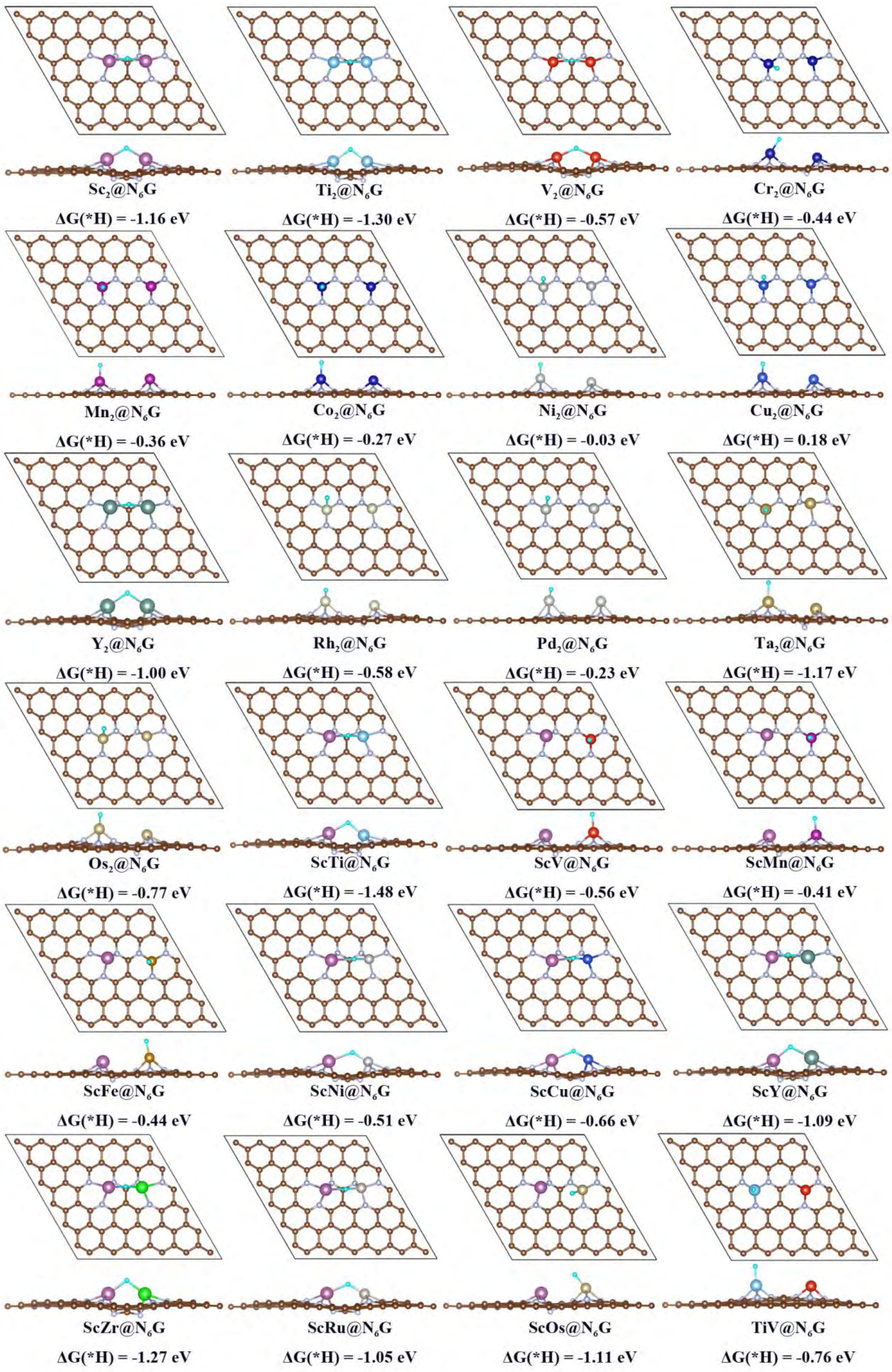

Figure S87. (a) Optimized structures and the corresponding adsorption free energies of hydrogen atom (ΔG(*H)) on 72 stable systems.

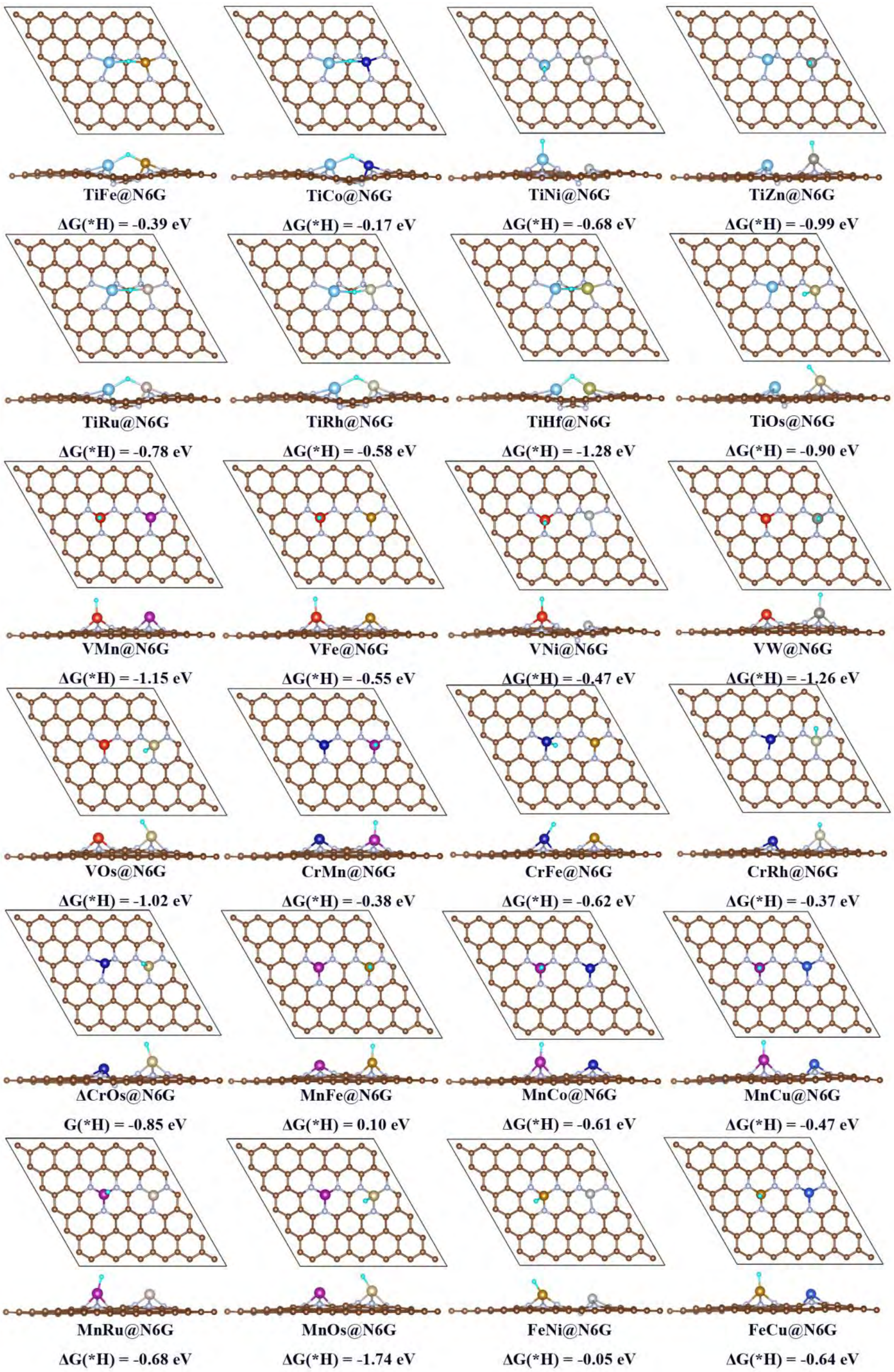

Figure S87. (b) Optimized structures and the corresponding adsorption free energies of hydrogen atom (ΔG(*H)) on 72 stable systems.

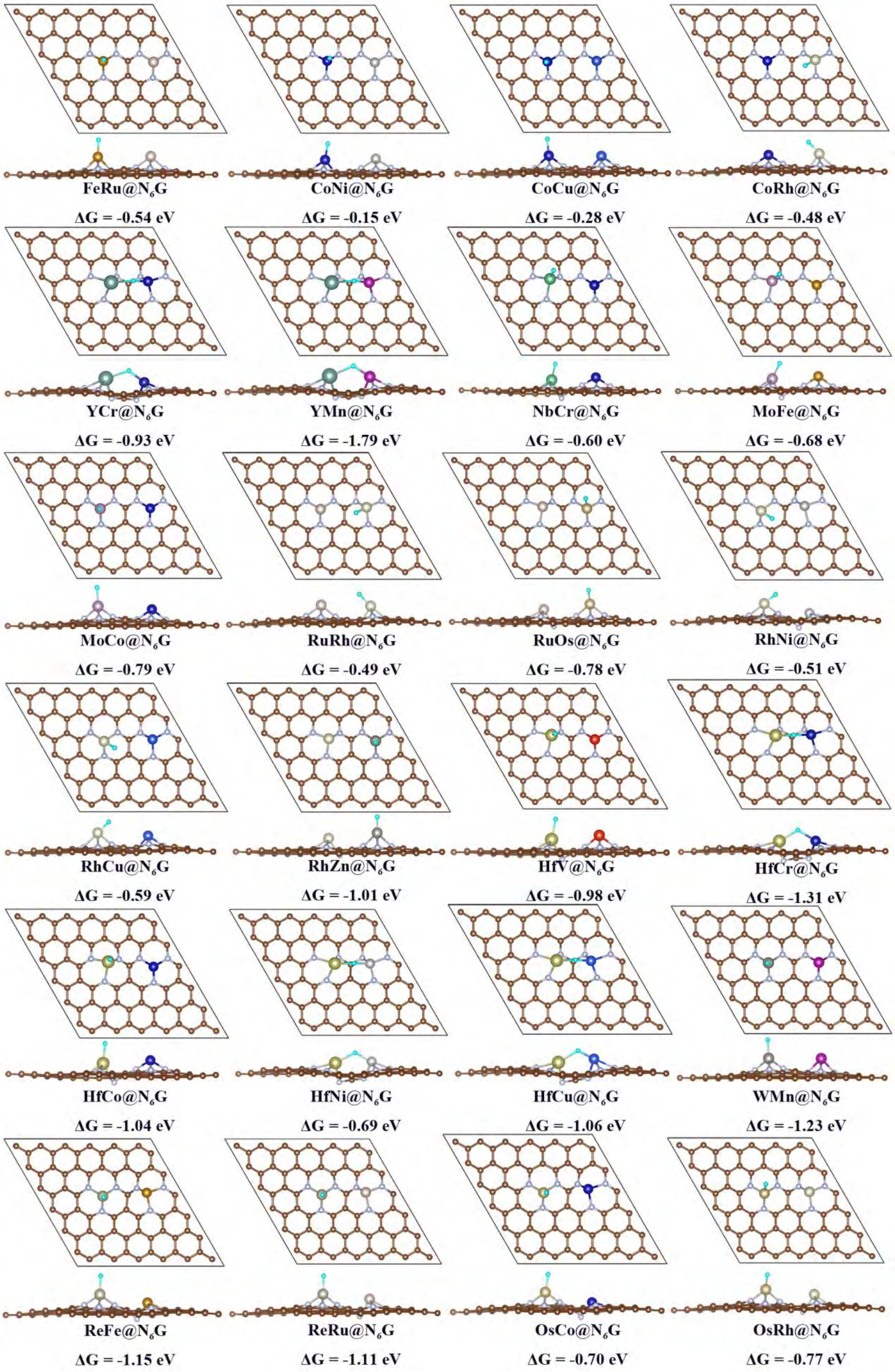

Figure S87. (c) Optimized structures and the corresponding adsorption free energies of hydrogen atom (ΔG(*H)) on 72 stable systems.

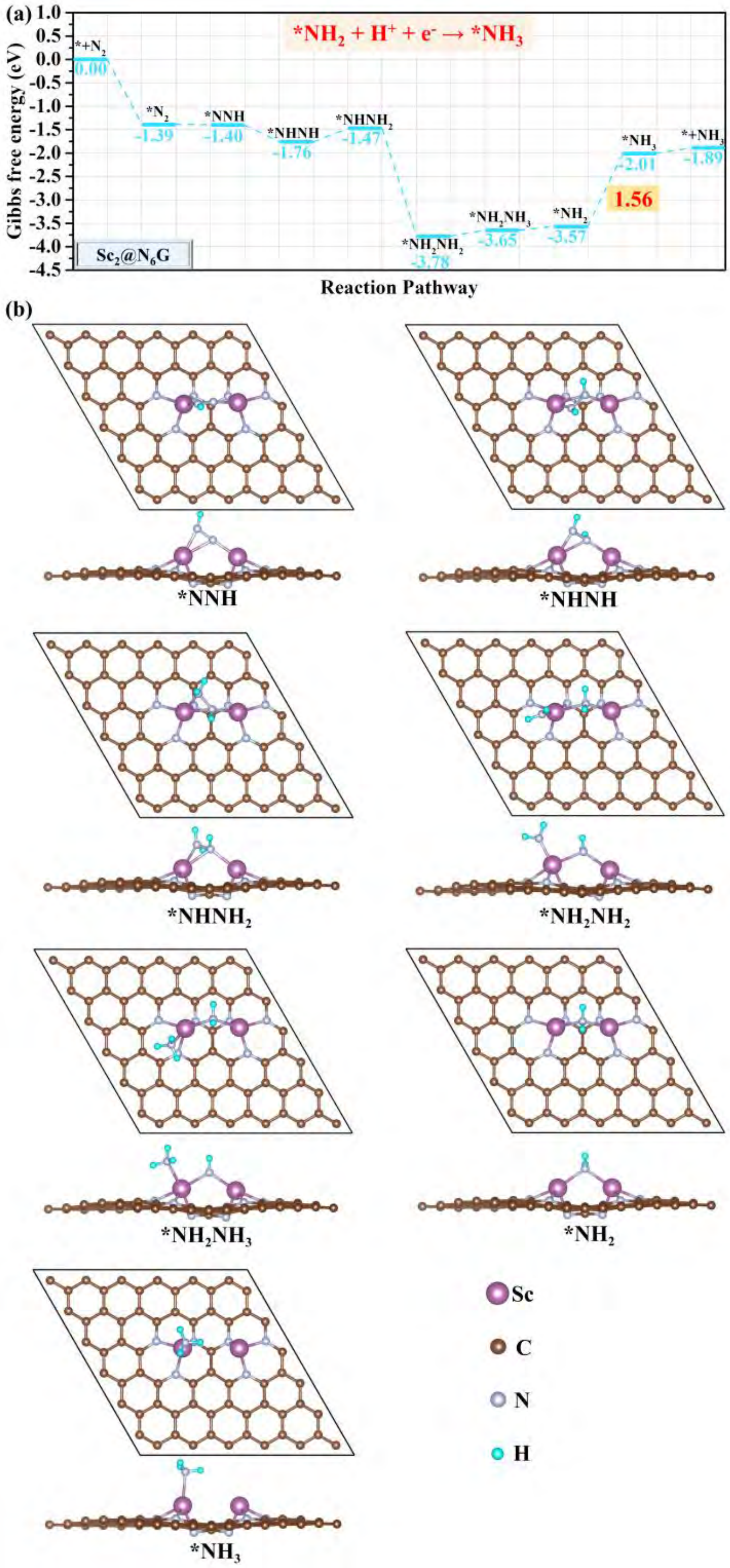

Figure S88. (a) Gibbs free energy diagram for N_2 reduction to NH_3 production on the $Sc_2@N_6G$ system. (b) Optimized structures of various intermediates along the hydrogenation pathway of N_2 reduction to NH_3 production on the $Sc_2@N_6G$ system.

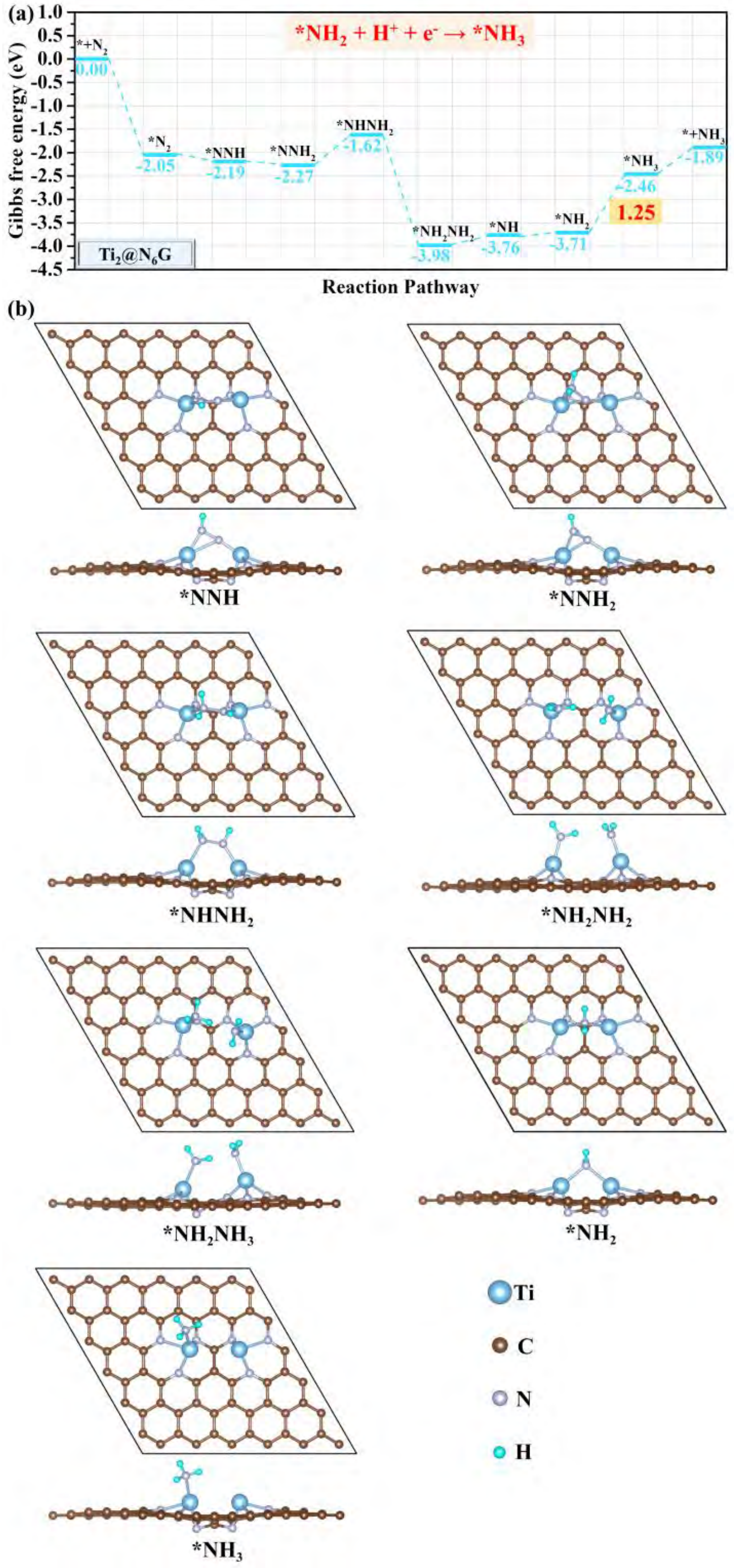

Figure S89. (a) Gibbs free energy diagram for N_2 reduction to NH_3 production on the $Ti_2@N_6G$ system. (b) Optimized structures of various intermediates along the hydrogenation pathway of N_2 reduction to NH_3 production on the $Ti_2@N_6G$ system.

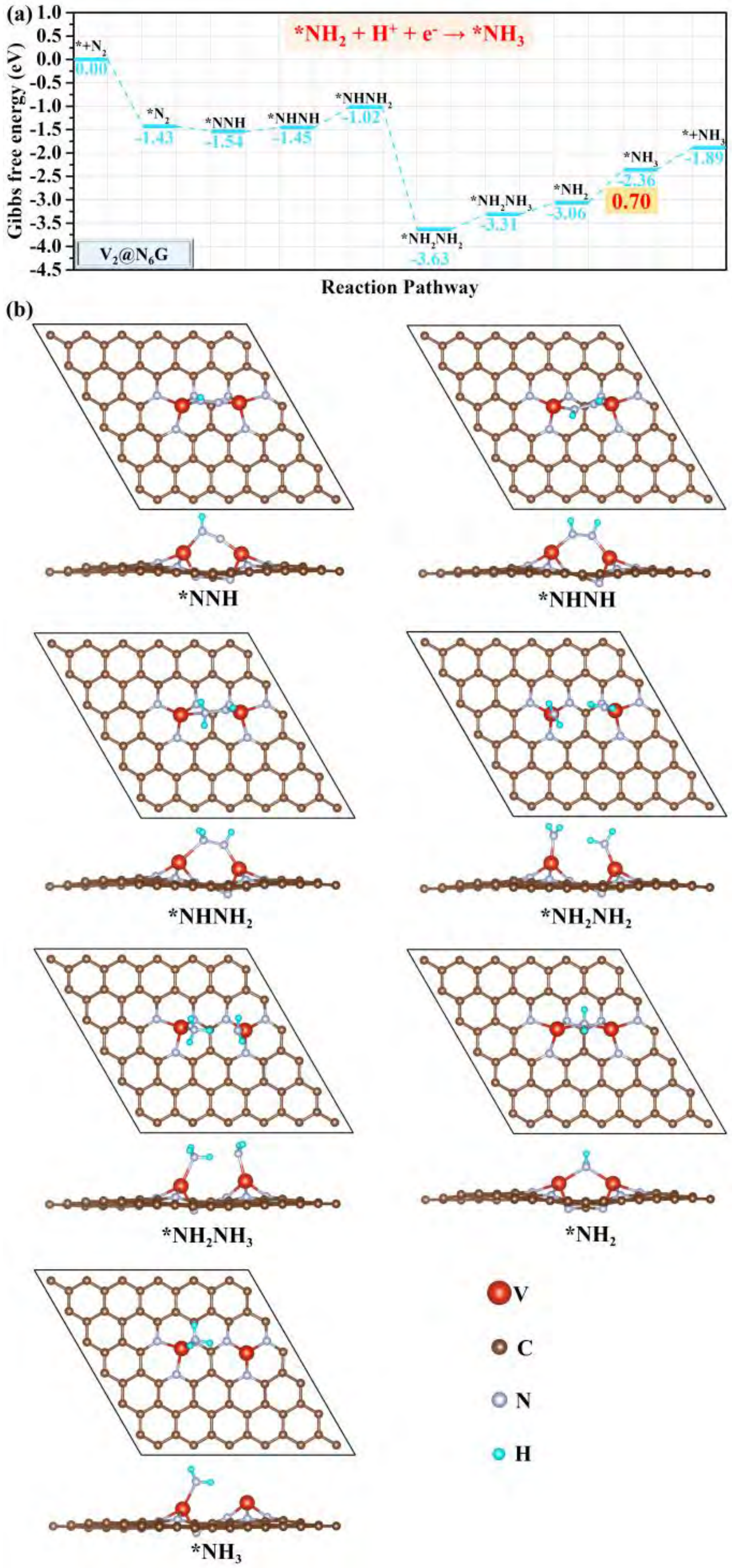

Figure S90. (a) Gibbs free energy diagram for N_2 reduction to NH_3 production on the V_2@N_6G system. (b) Optimized structures of various intermediates along the hydrogenation pathway of N_2 reduction to NH_3 production on the V_2@N_6G system.

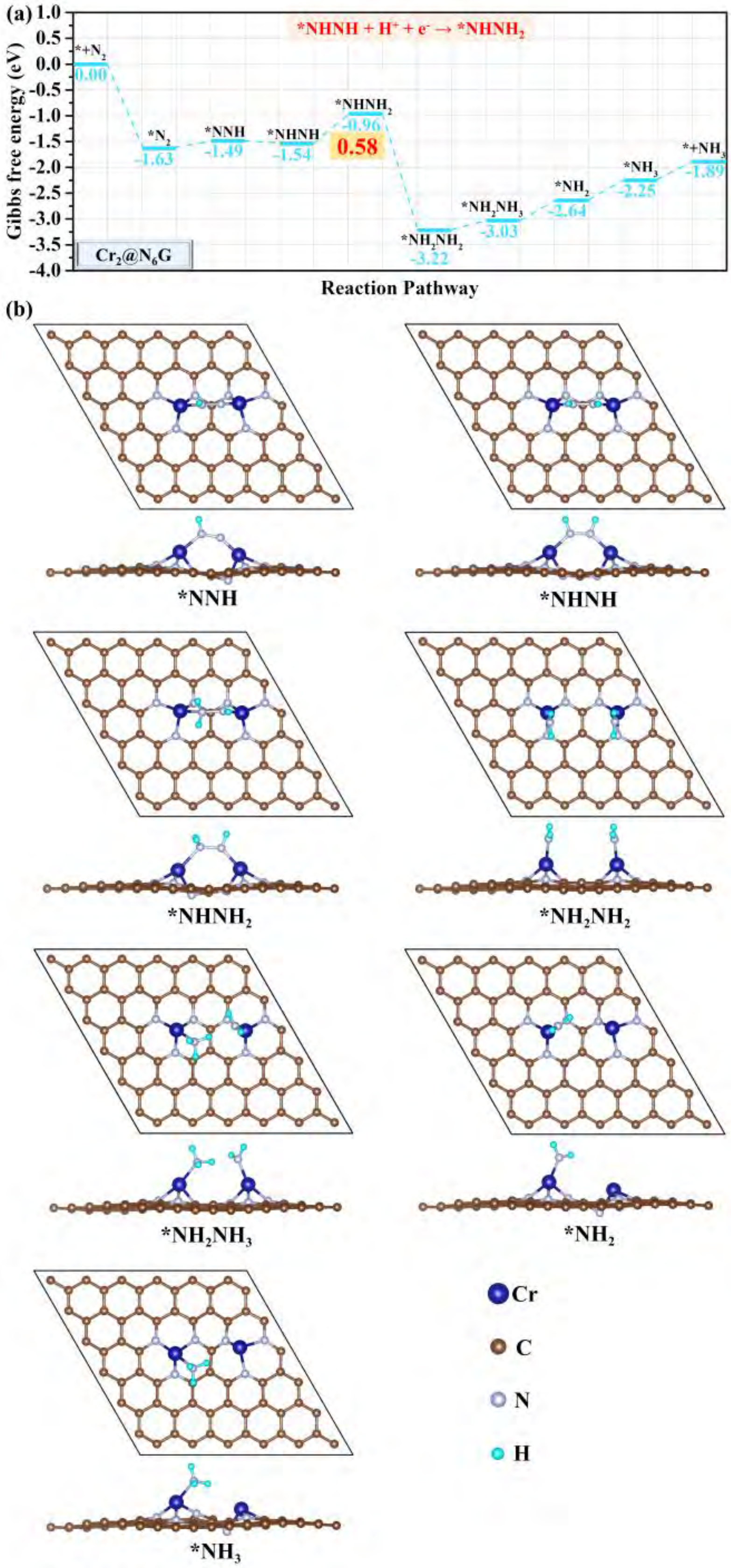

Figure S91. (a) Gibbs free energy diagram for N_2 reduction to NH_3 production on the $Cr_2@N_6G$ system. (b) Optimized structures of various intermediates along the hydrogenation pathway of N_2 reduction to NH_3 production on the $Cr_2@N_6G$ system.

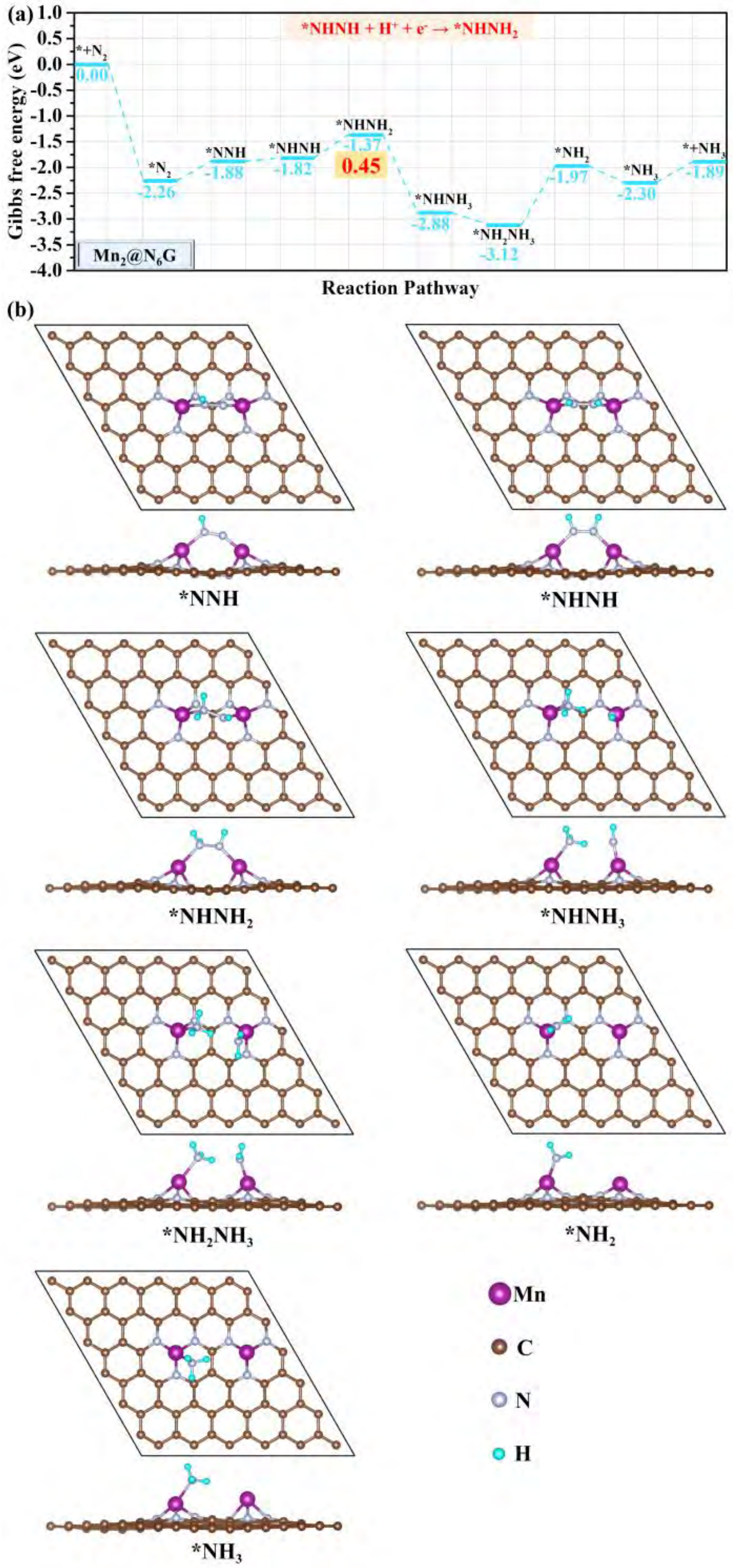

Figure S92. (a) Gibbs free energy diagram for N_2 reduction to NH_3 production on the $Mn_2@N_6G$ system. (b) Optimized structures of various intermediates along the hydrogenation pathway of N_2 reduction to NH_3 production on the $Mn_2@N_6G$ system.

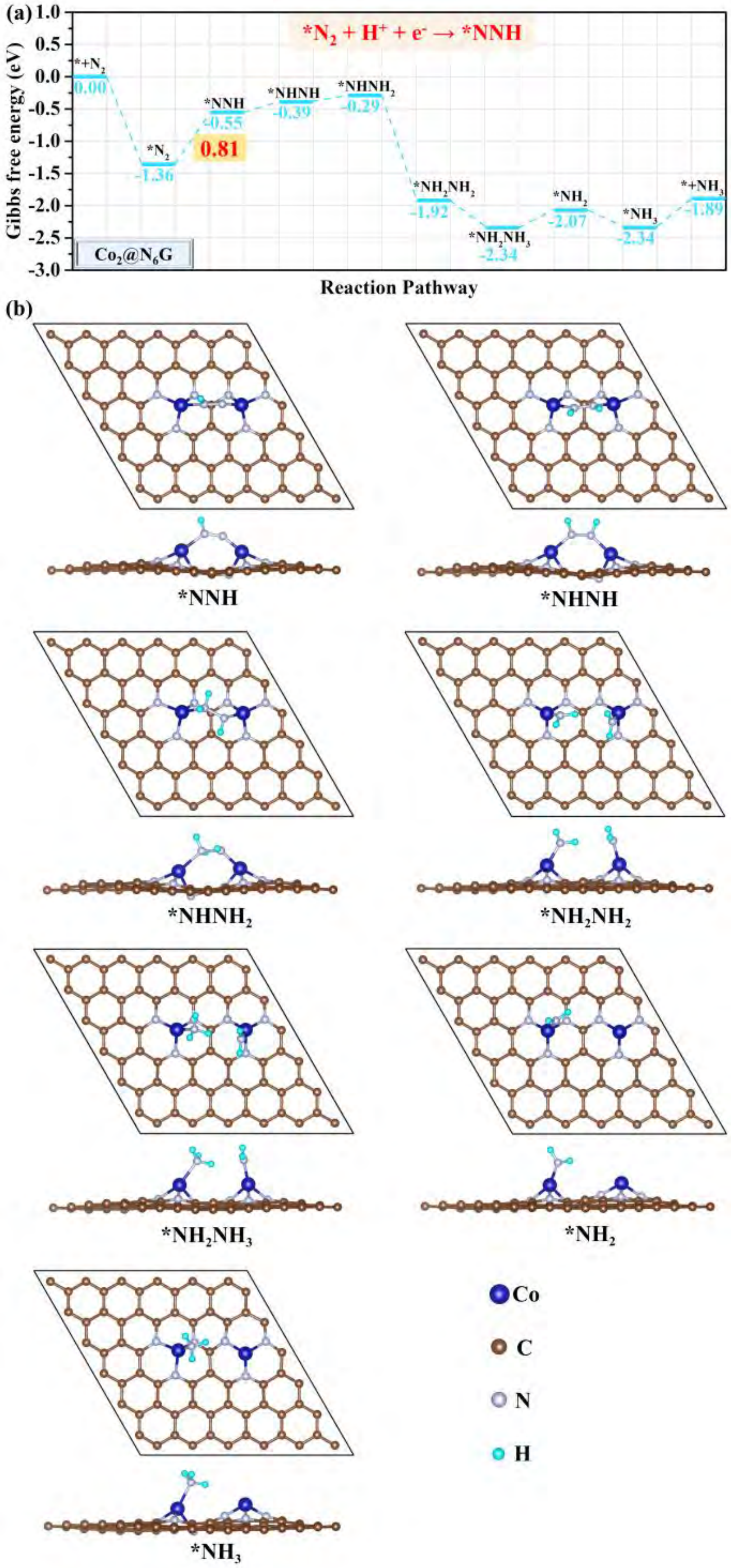

Figure S93. (a) Gibbs free energy diagram for N_2 reduction to NH_3 production on the $Co_2@N_6G$ system. (b) Optimized structures of various intermediates along the hydrogenation pathway of N_2 reduction to NH_3 production on the $Co_2@N_6G$ system.

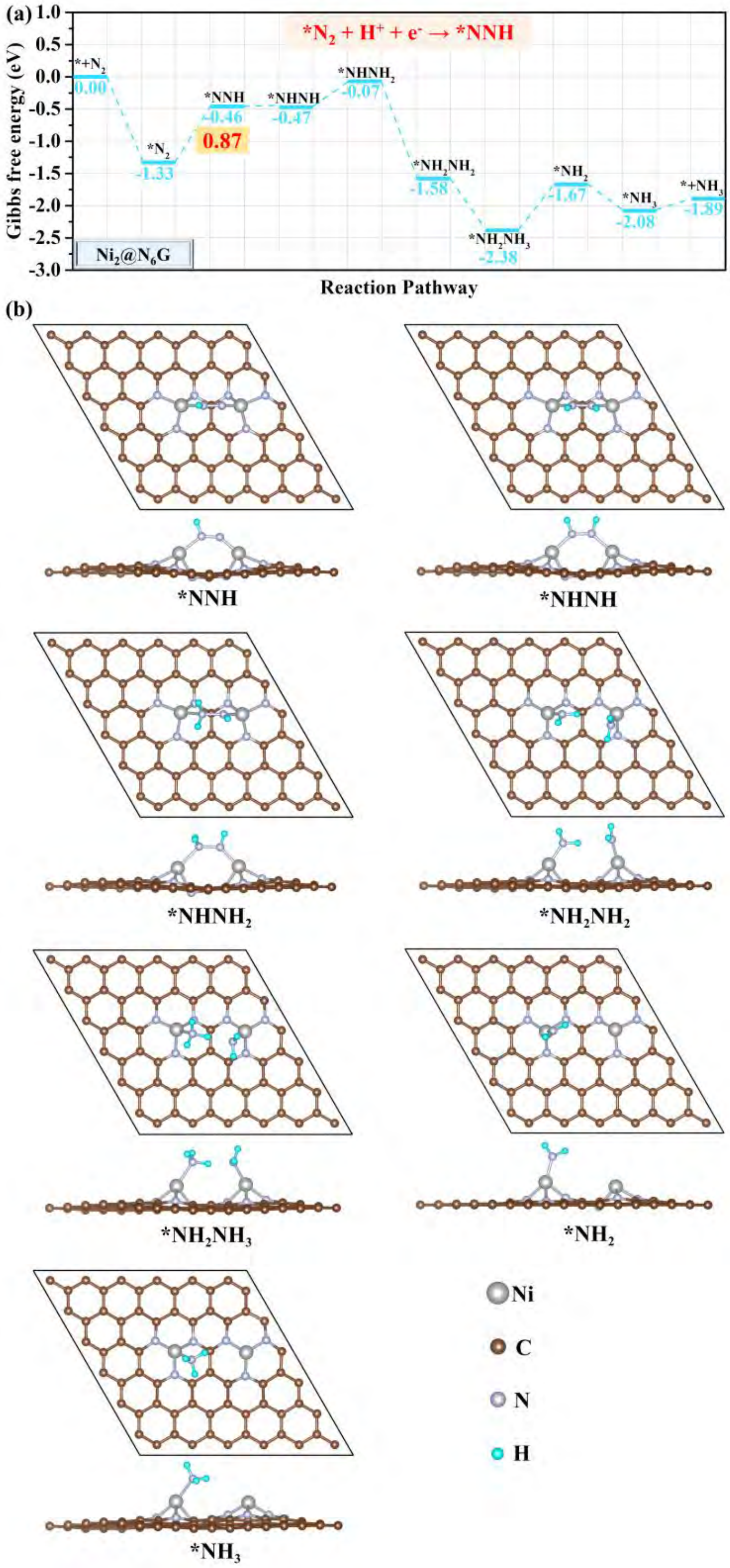

Figure S94. (a) Gibbs free energy diagram for N_2 reduction to NH_3 production on the Ni_2@N_6G system. (b) Optimized structures of various intermediates along the hydrogenation pathway of N_2 reduction to NH_3 production on the Ni_2@N_6G system.

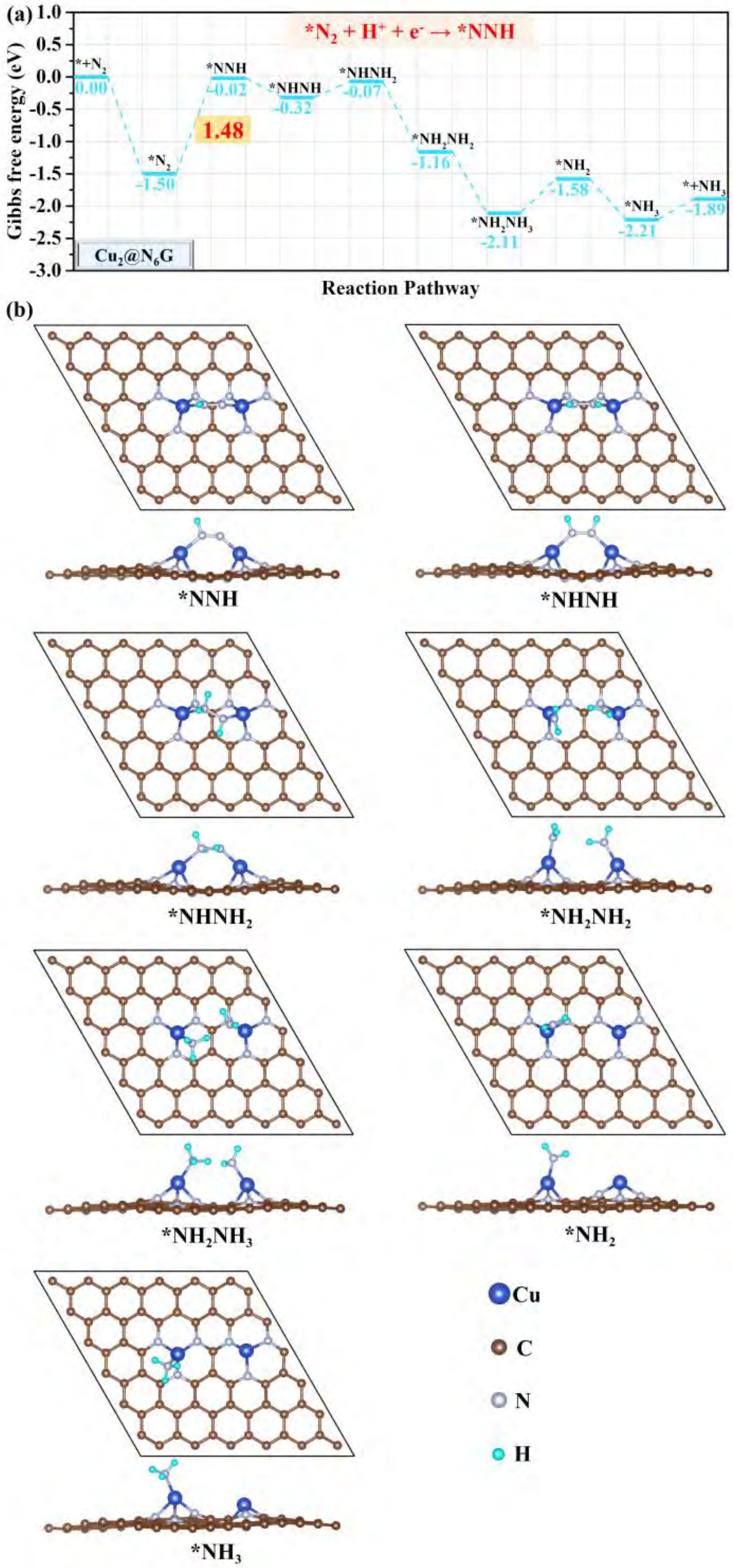

Figure S95. (a) Gibbs free energy diagram for N_2 reduction to NH_3 production on the $Cu_2@N_6G$ system. (b) Optimized structures of various intermediates along the hydrogenation pathway of N_2 reduction to NH_3 production on the $Cu_2@N_6G$ system.

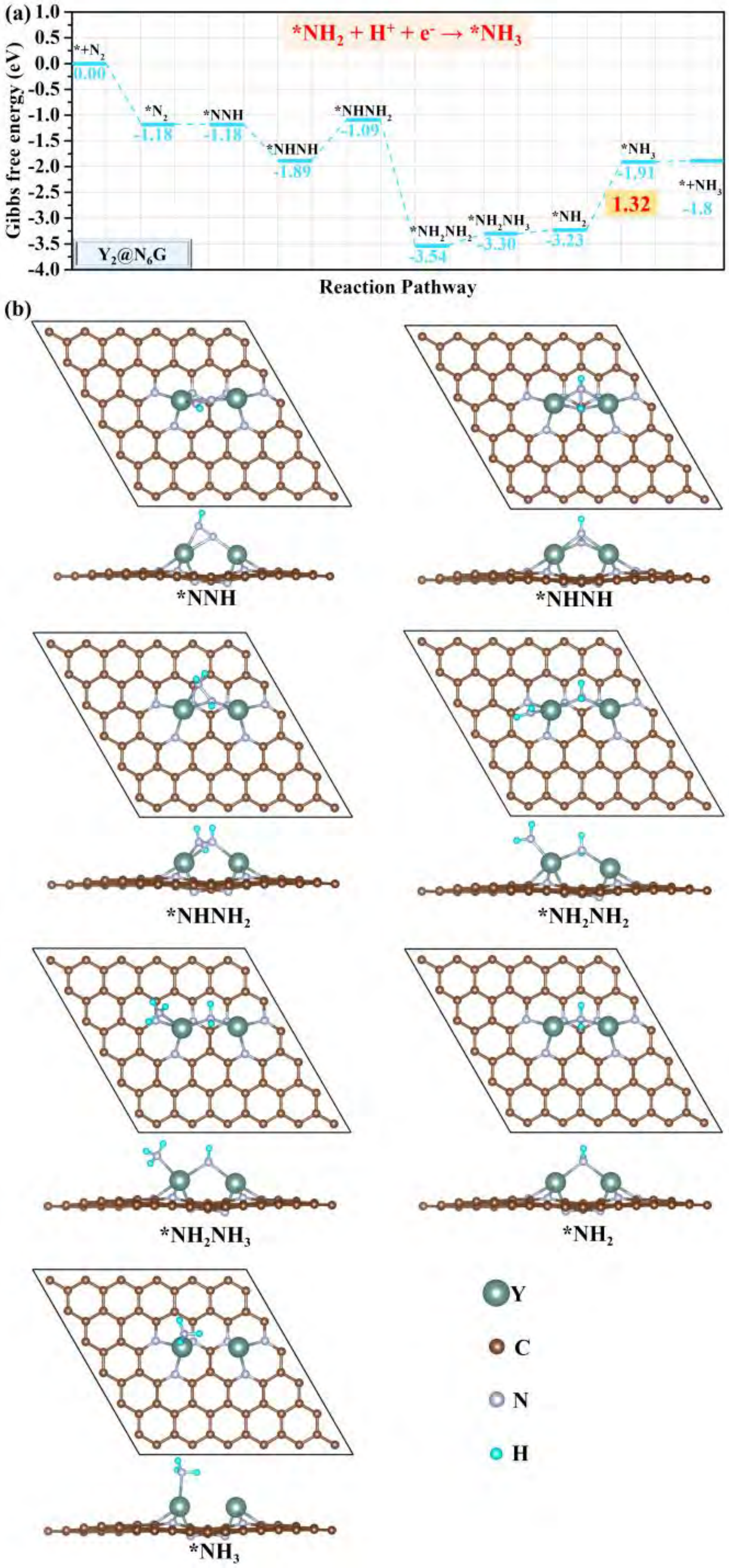

Figure S96. (a) Gibbs free energy diagram for N_2 reduction to NH_3 production on the Y_2@N_6G system. (b) Optimized structures of various intermediates along the hydrogenation pathway of N_2 reduction to NH_3 production on the Y_2@N_6G system.

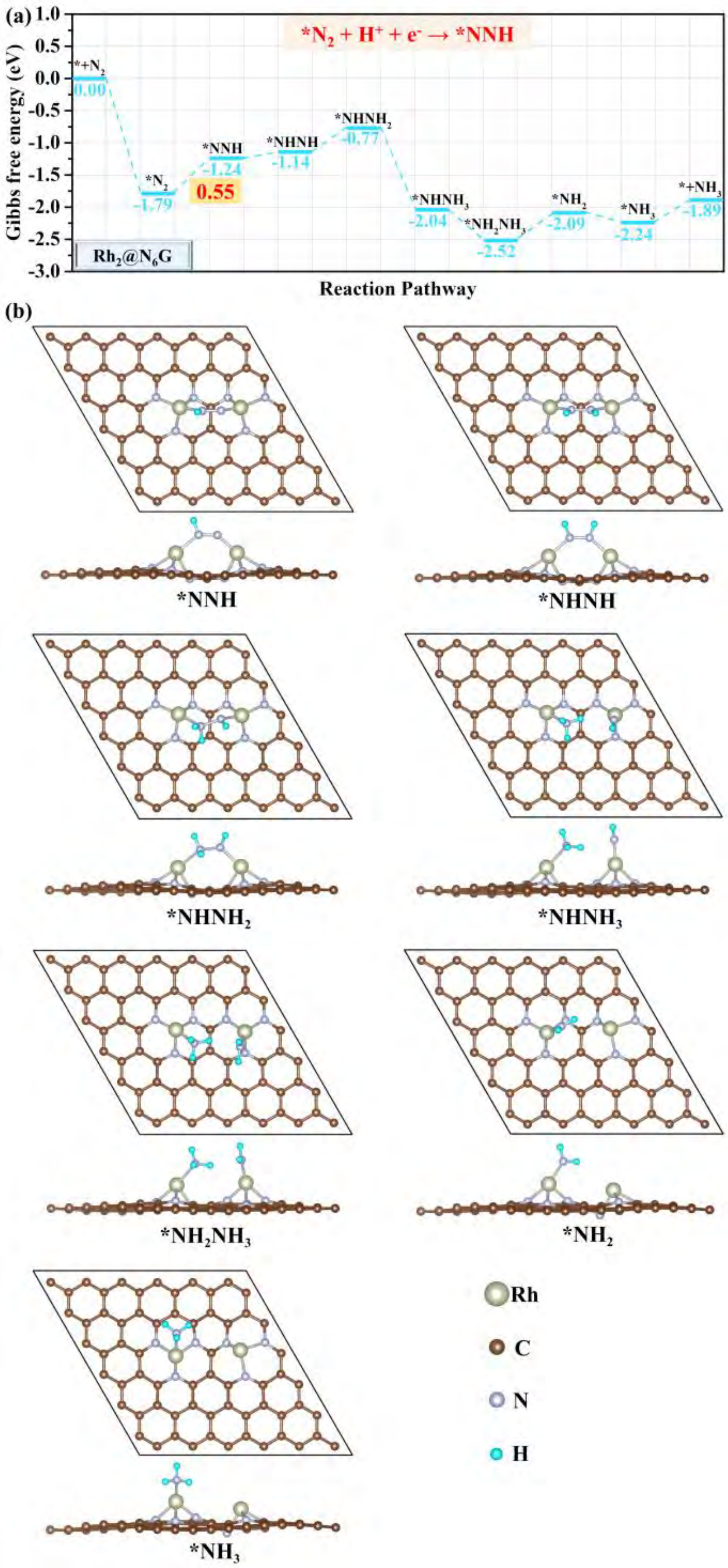

Figure S97. (a) Gibbs free energy diagram for N_2 reduction to NH_3 production on the Rh_2@N_6G system. (b) Optimized structures of various intermediates along the hydrogenation pathway of N_2 reduction to NH_3 production on the Rh_2@N_6G system.

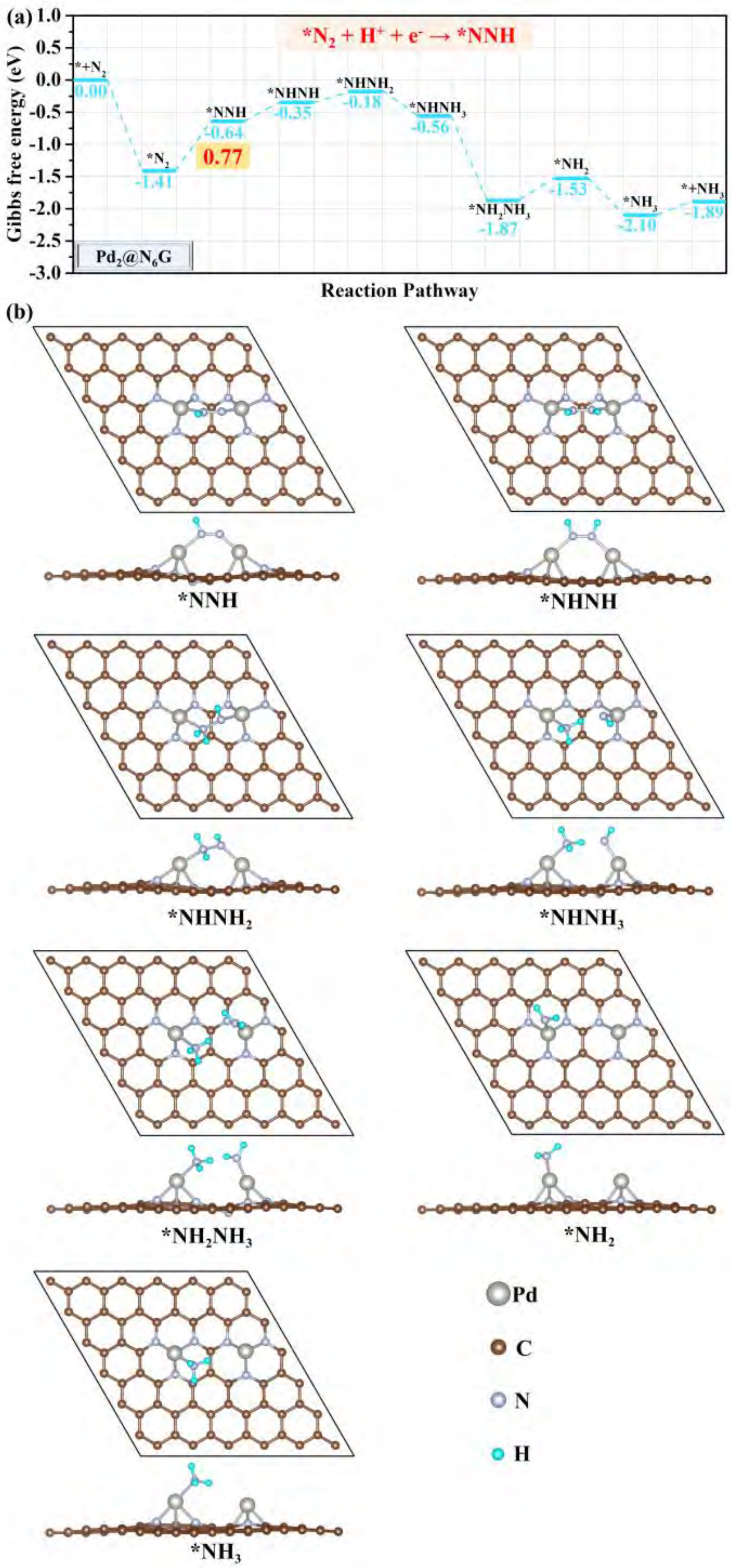

Figure S98. (a) Gibbs free energy diagram for N_2 reduction to NH_3 production on the $Pd_2@N_6G$ system. (b) Optimized structures of various intermediates along the hydrogenation pathway of N_2 reduction to NH_3 production on the $Pd_2@N_6G$ system.

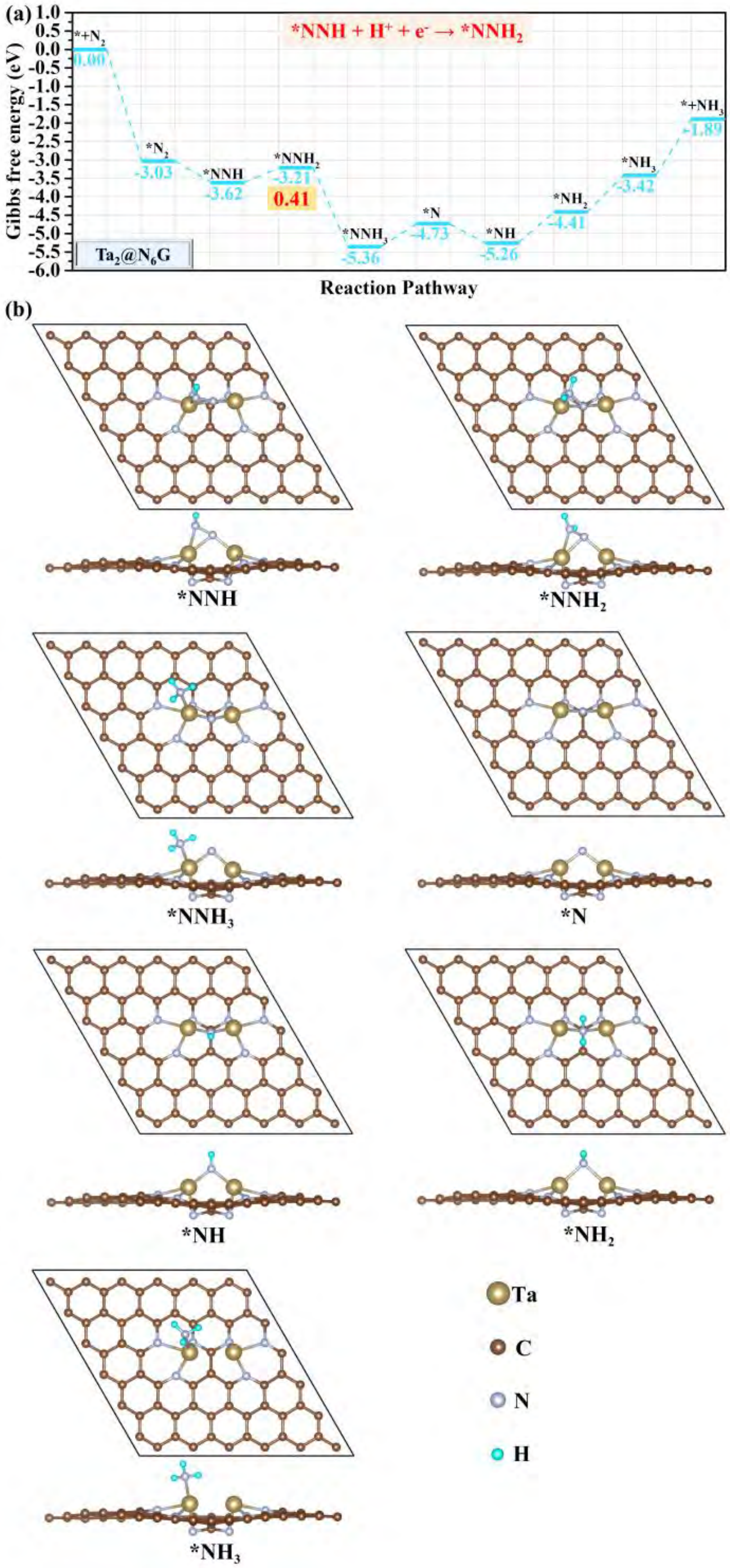

Figure S99. (a) Gibbs free energy diagram for N_2 reduction to NH_3 production on the $Ta_2@N_6G$ system. (b) Optimized structures of various intermediates along the hydrogenation pathway of N_2 reduction to NH_3 production on the $Ta_2@N_6G$ system.

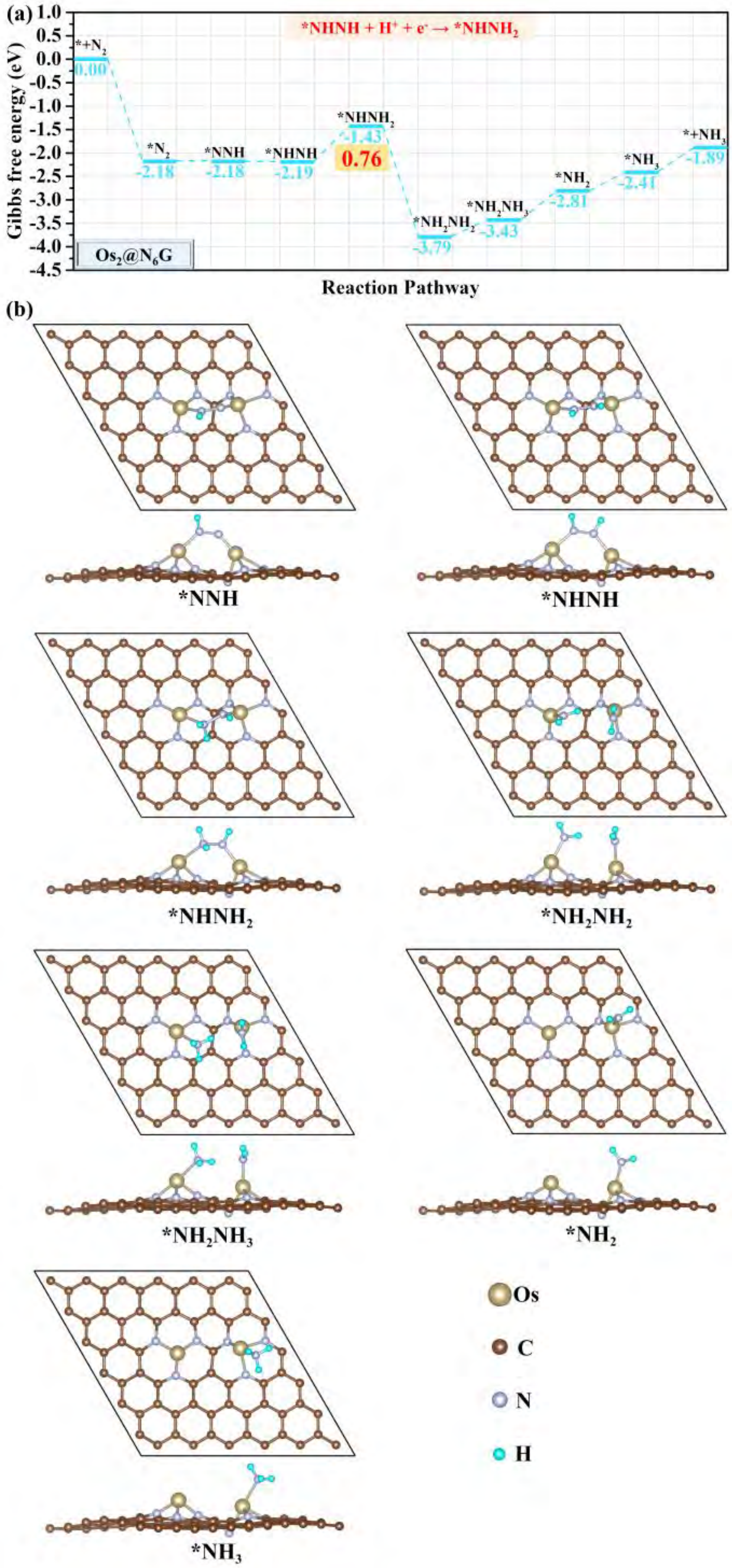

Figure S100. (a) Gibbs free energy diagram for N_2 reduction to NH_3 production on the Os_2@N_6G system. (b) Optimized structures of various intermediates along the hydrogenation pathway of N_2 reduction to NH_3 production on the Os_2@N_6G system.

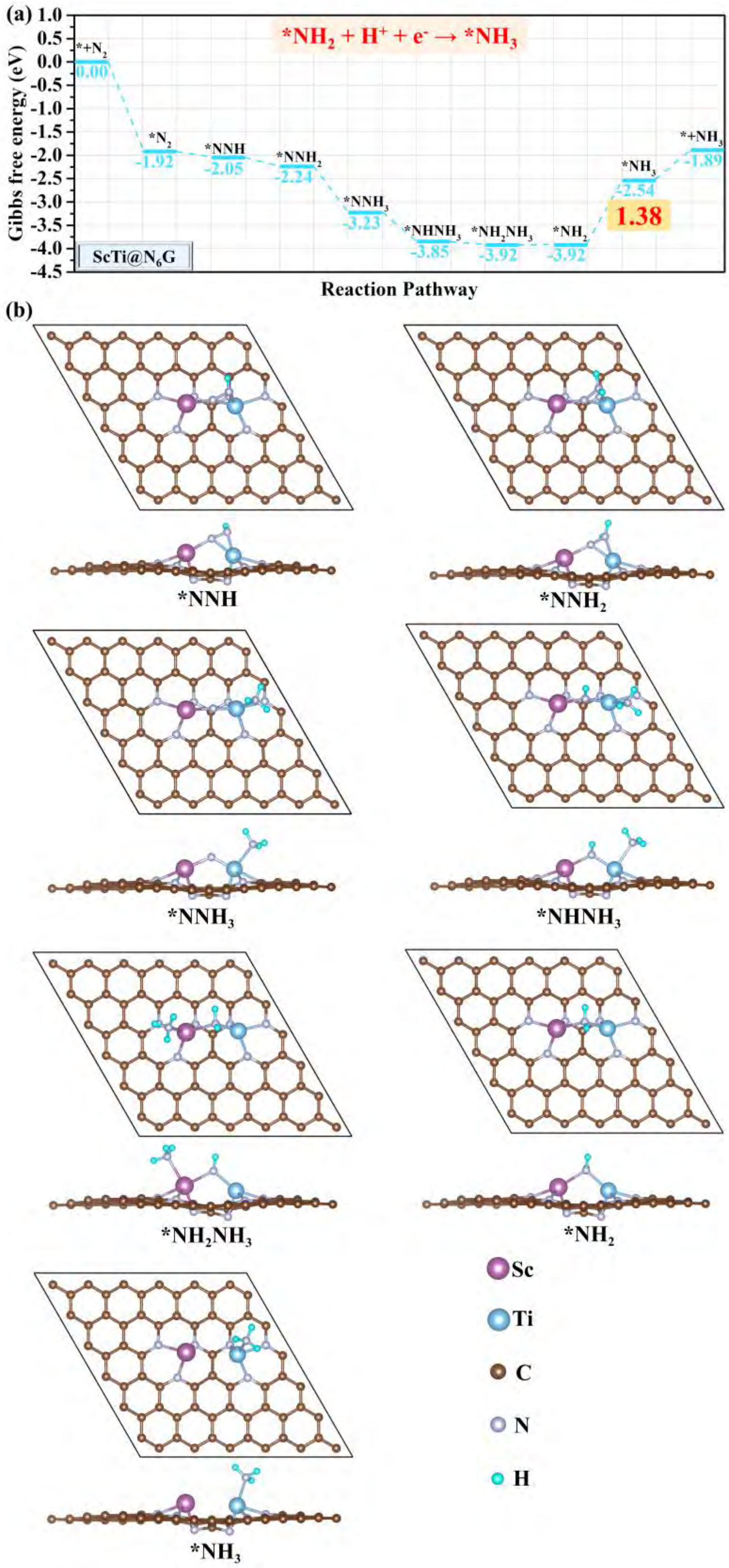

Figure S101. (a) Gibbs free energy diagram for N_2 reduction to NH_3 production on the ScTi@N_6G system. (b) Optimized structures of various intermediates along the hydrogenation pathway of N_2 reduction to NH_3 production on the ScTi@N_6G system.

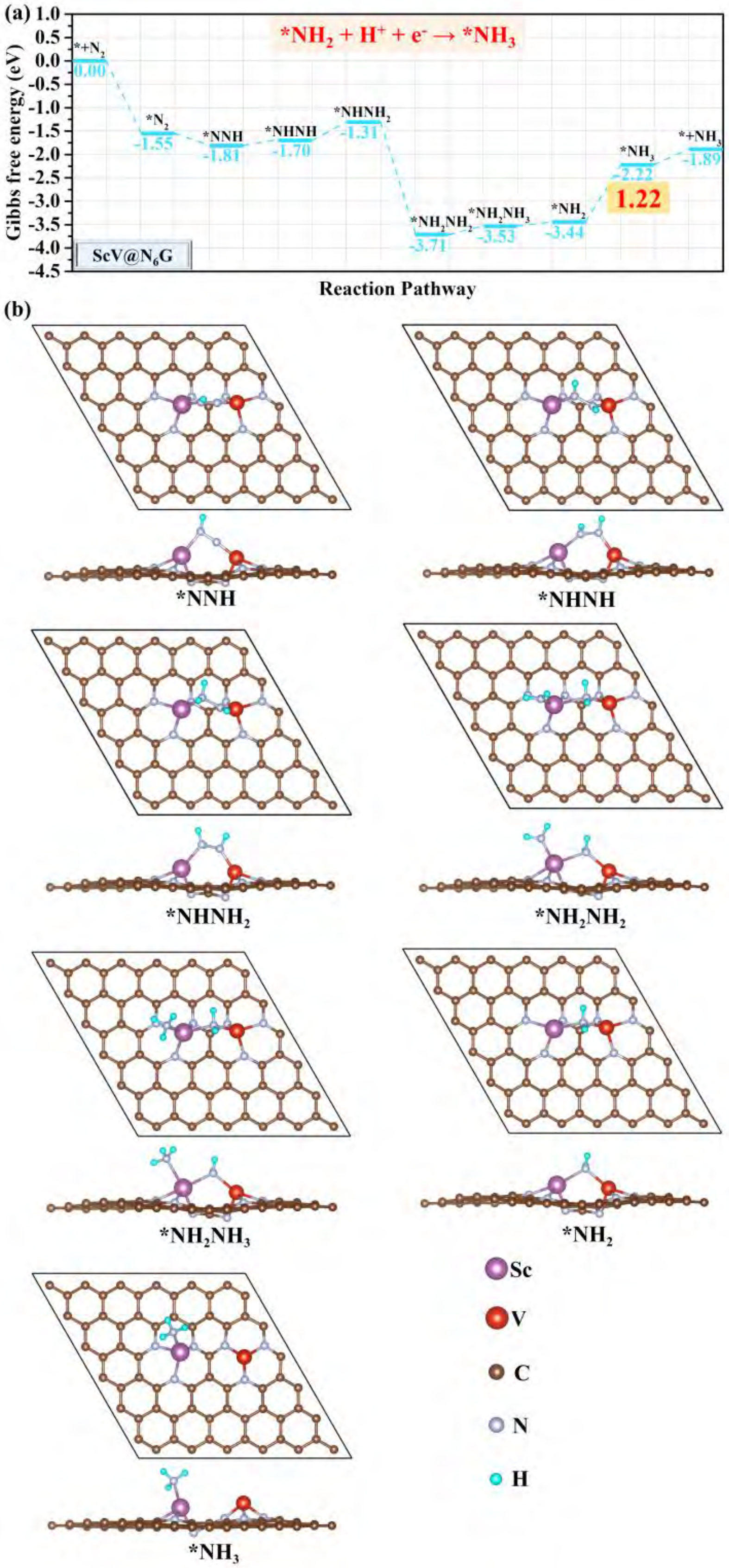

Figure S102. (a) Gibbs free energy diagram for N_2 reduction to NH_3 production on the ScV@N$_6$G system. (b) Optimized structures of various intermediates along the hydrogenation pathway of N_2 reduction to NH_3 production on the ScV@N$_6$G system.

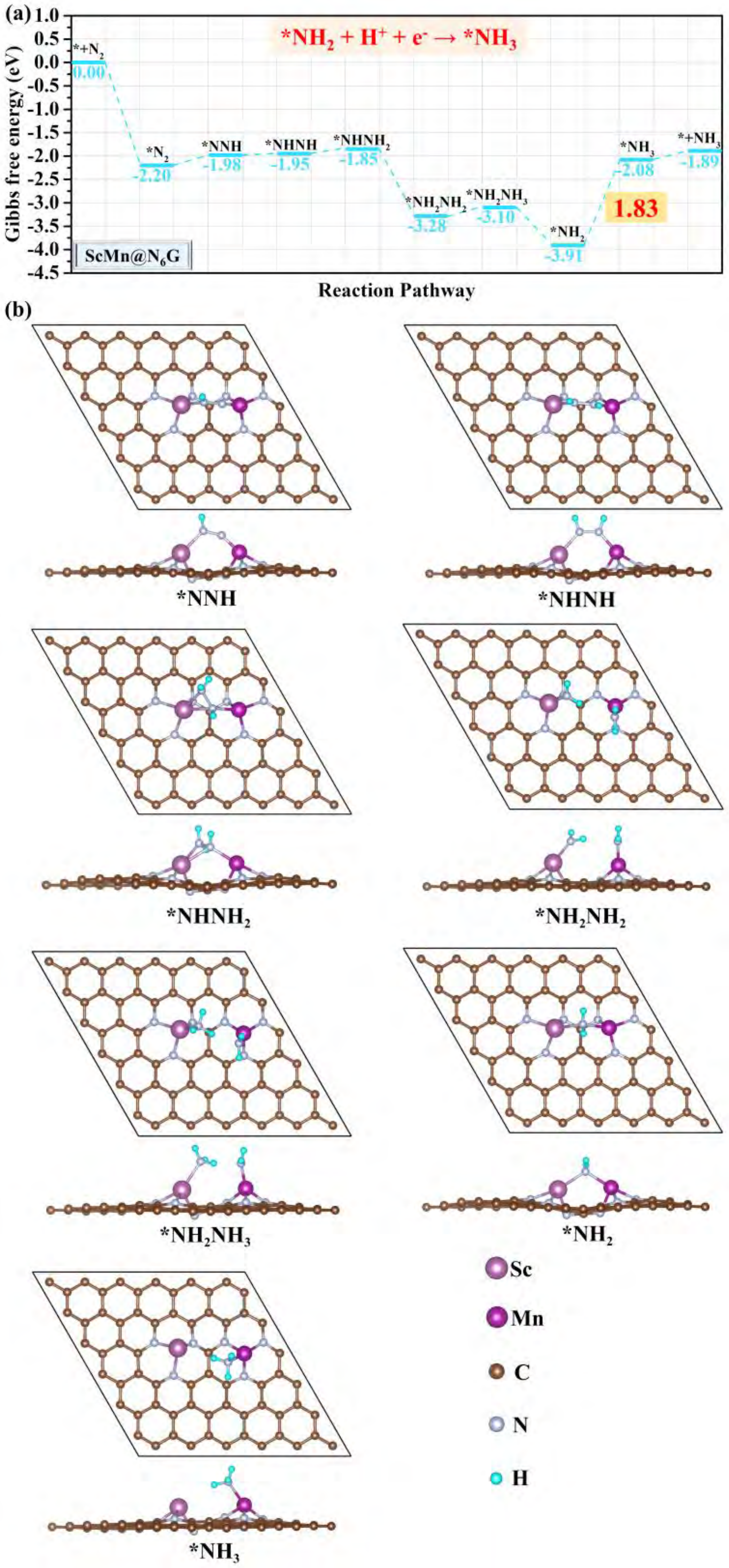

Figure S103. (a) Gibbs free energy diagram for N_2 reduction to NH_3 production on the ScMn@N_6G system. (b) Optimized structures of various intermediates along the hydrogenation pathway of N_2 reduction to NH_3 on the ScMn@N_6G system.

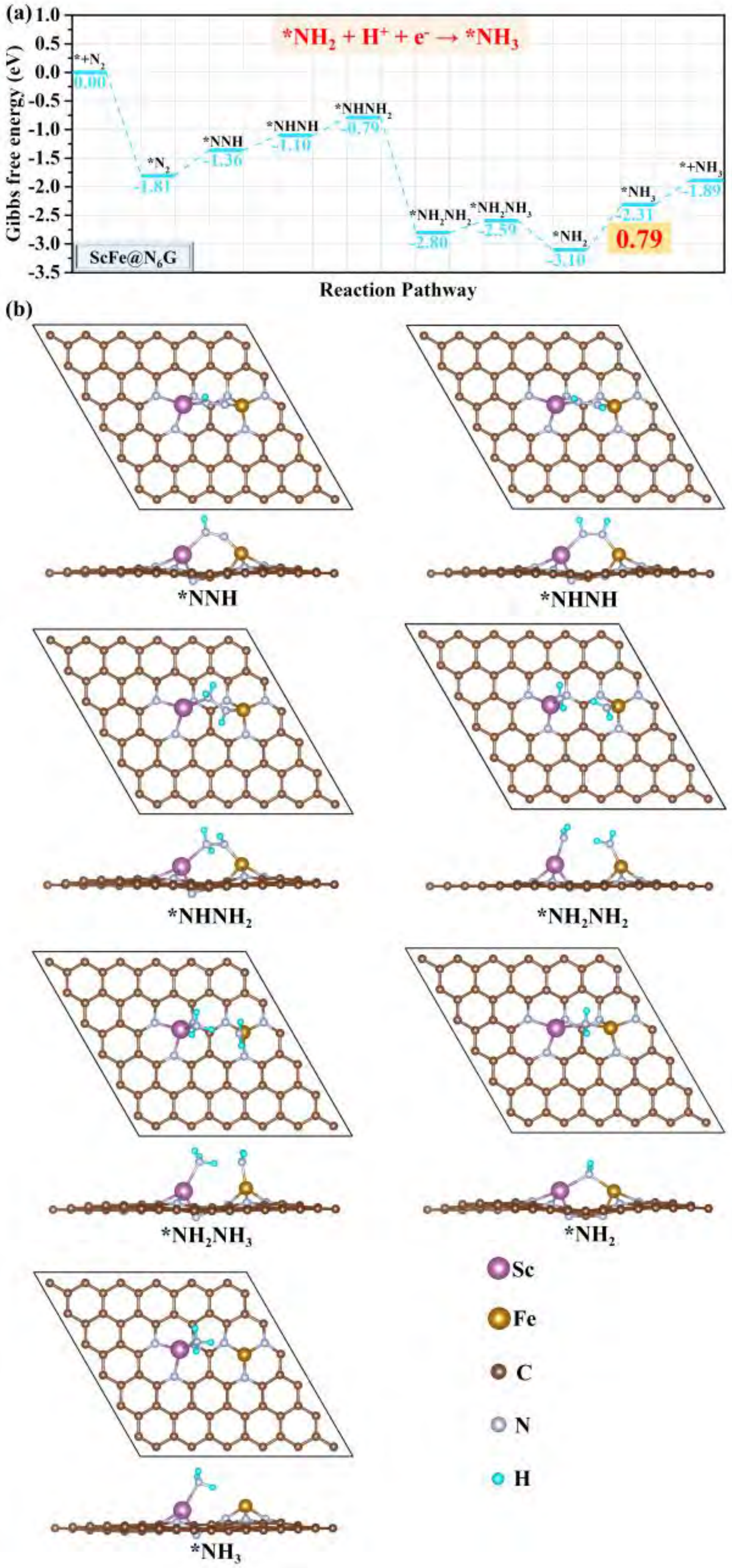

Figure S104. (a) Gibbs free energy diagram for N_2 reduction to NH_3 production on the ScFe@N_6G system. (b) Optimized structures of various intermediates along the hydrogenation pathway of N_2 reduction to NH_3 production on the ScFe@N_6G system.

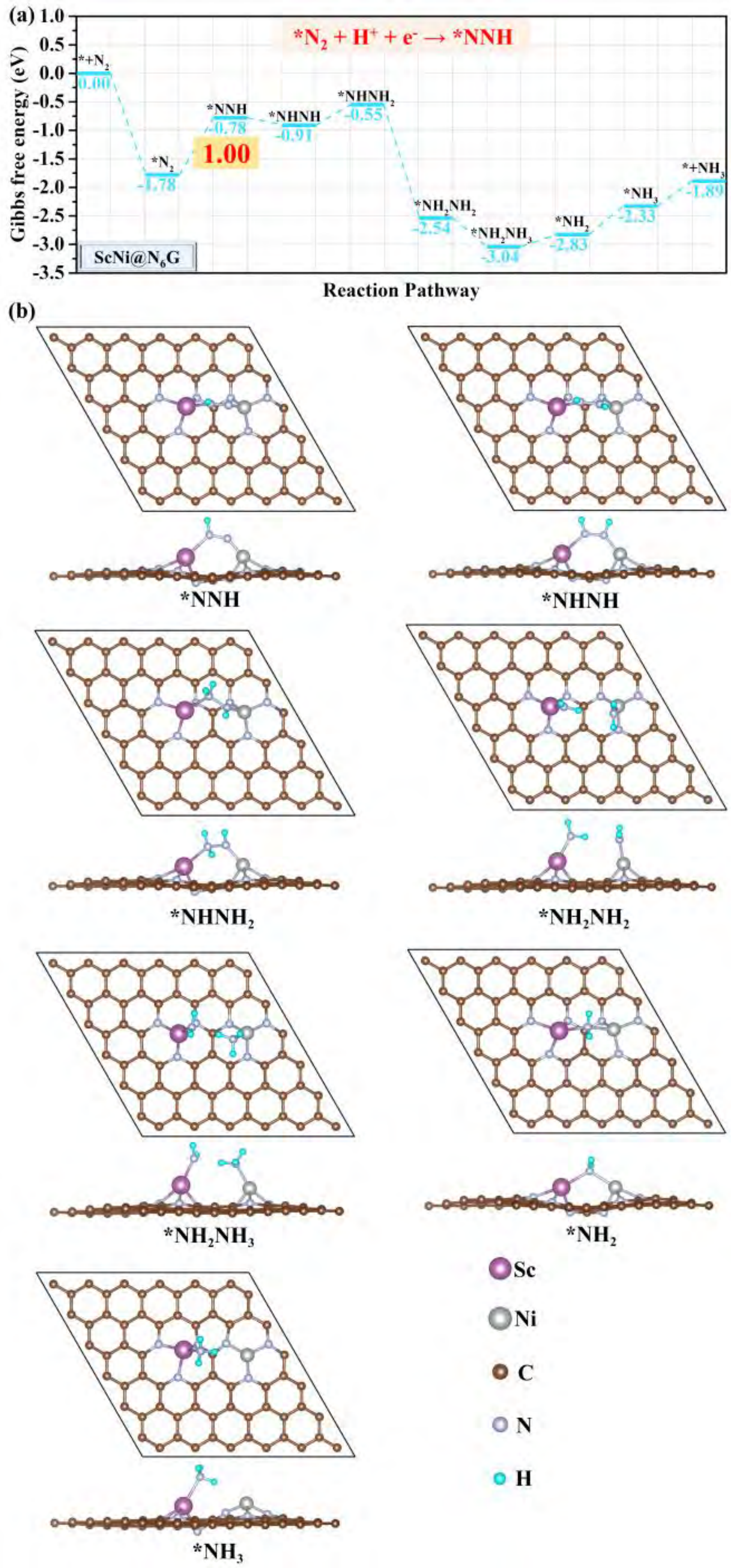

Figure S105. (a) Gibbs free energy diagram for N_2 reduction to NH_3 production on the ScNi@N_6G system. (b) Optimized structures of various intermediates along the hydrogenation pathway of N_2 reduction to NH_3 production on the ScNi@N_6G system.

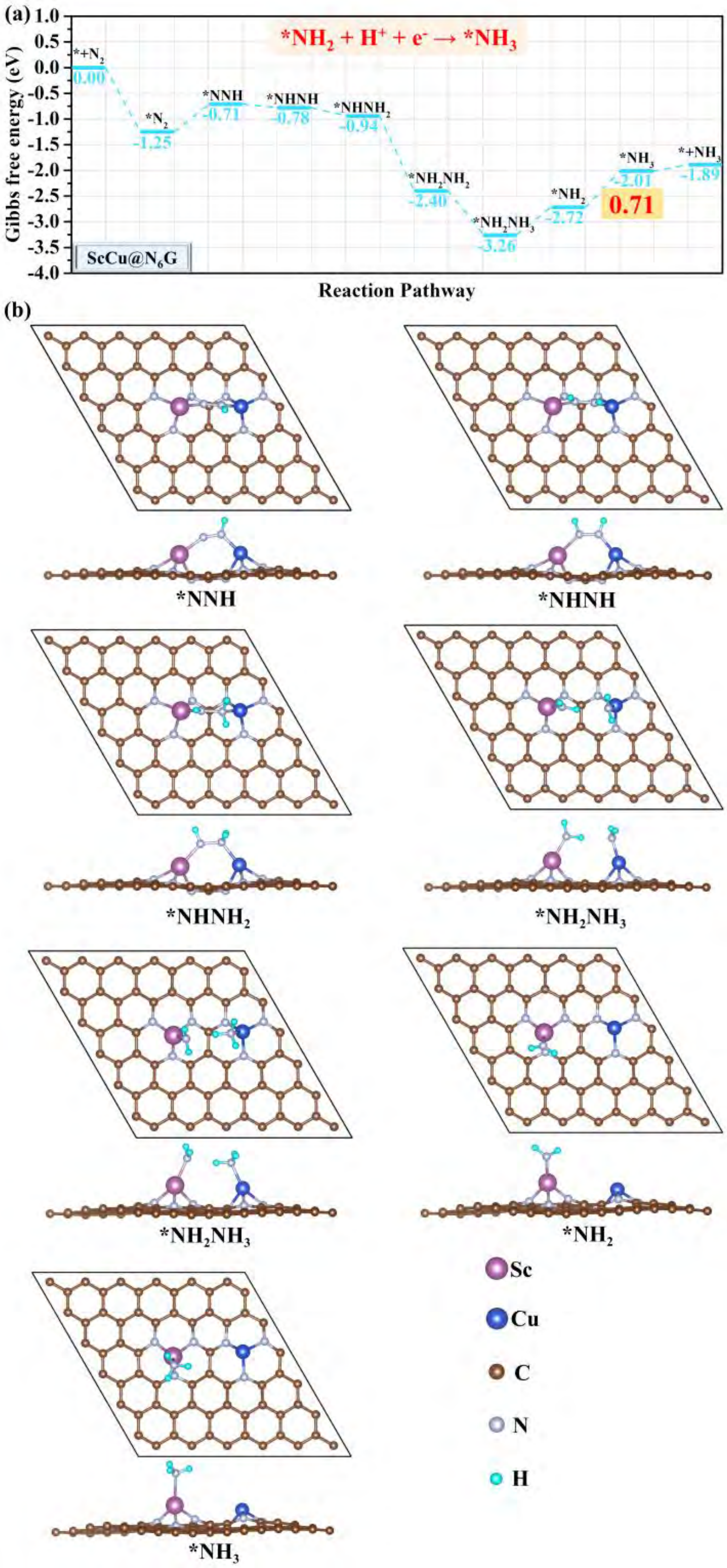

Figure S106. (a) Gibbs free energy diagram for N_2 reduction to NH_3 production on the ScCu@N_6G system. (b) Optimized structures of various intermediates along the hydrogenation pathway of N_2 reduction to NH_3 production on the ScCu@N_6G system.

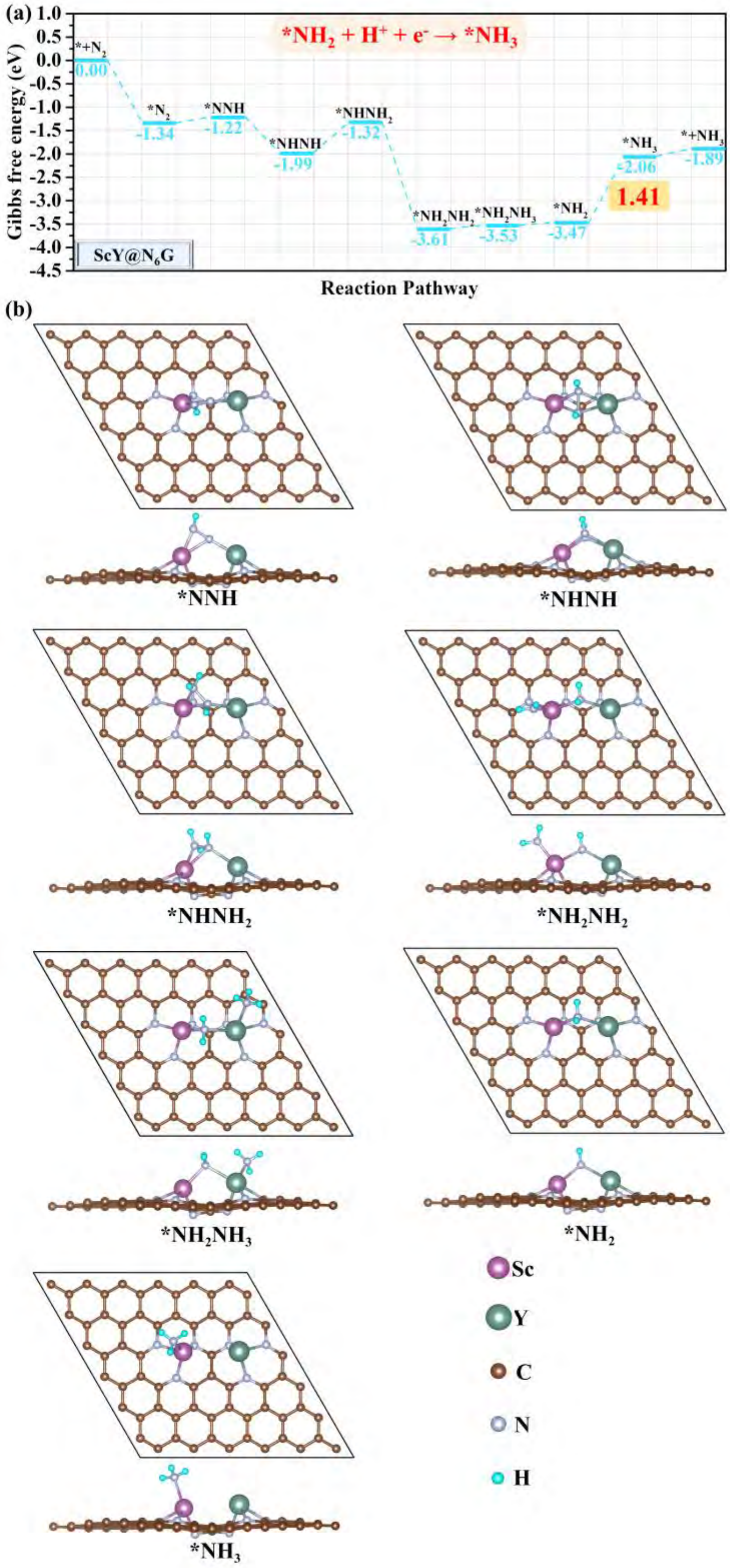

Figure S107. (a) Gibbs free energy diagram for N_2 reduction to NH_3 production on the ScY@N$_6$G system. (b) Optimized structures of various intermediates along the hydrogenation pathway of N_2 reduction to NH_3 production on the ScY@N$_6$G system.

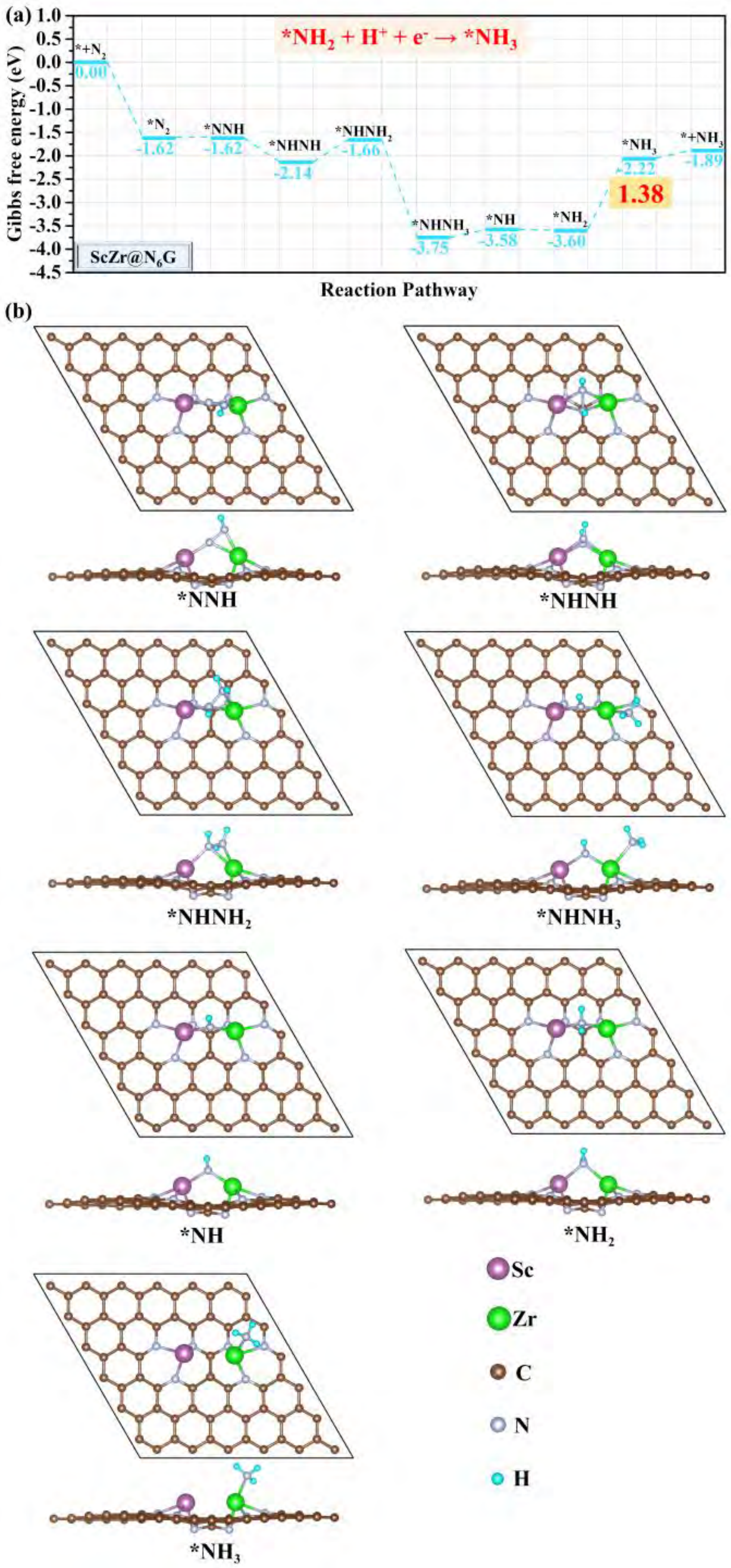

Figure S108. (a) Gibbs free energy diagram for N_2 reduction to NH_3 production on the ScZr@N_6G system. (b) Optimized structures of various intermediates along the hydrogenation pathway of N_2 reduction to NH_3 production on the ScZr@N_6G system.

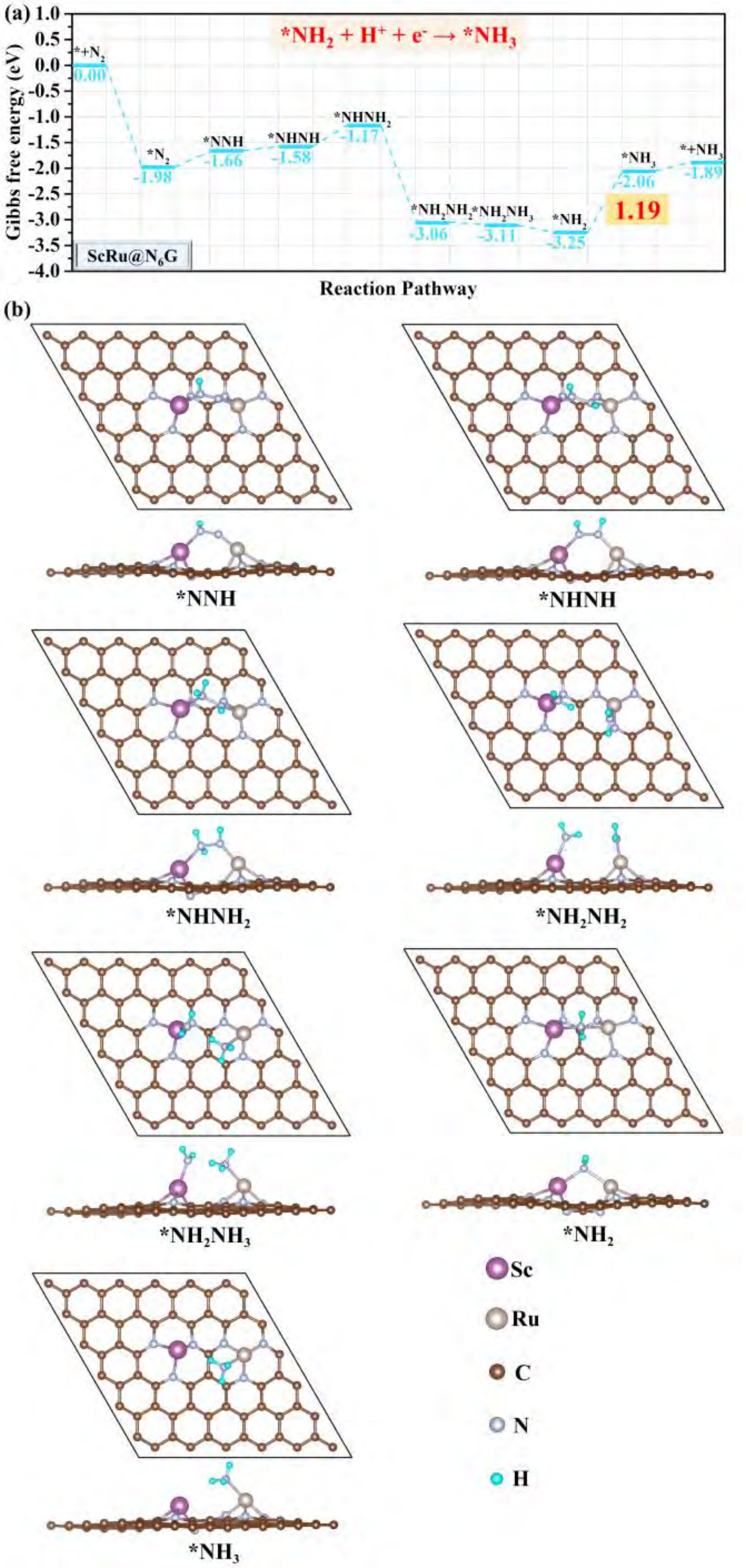

Figure S109. (a) Gibbs free energy diagram for N_2 reduction to NH_3 production on the ScRu@N_6G system. (b) Optimized structures of various intermediates along the hydrogenation pathway of N_2 reduction to NH_3 production on the ScRu@N_6G system.

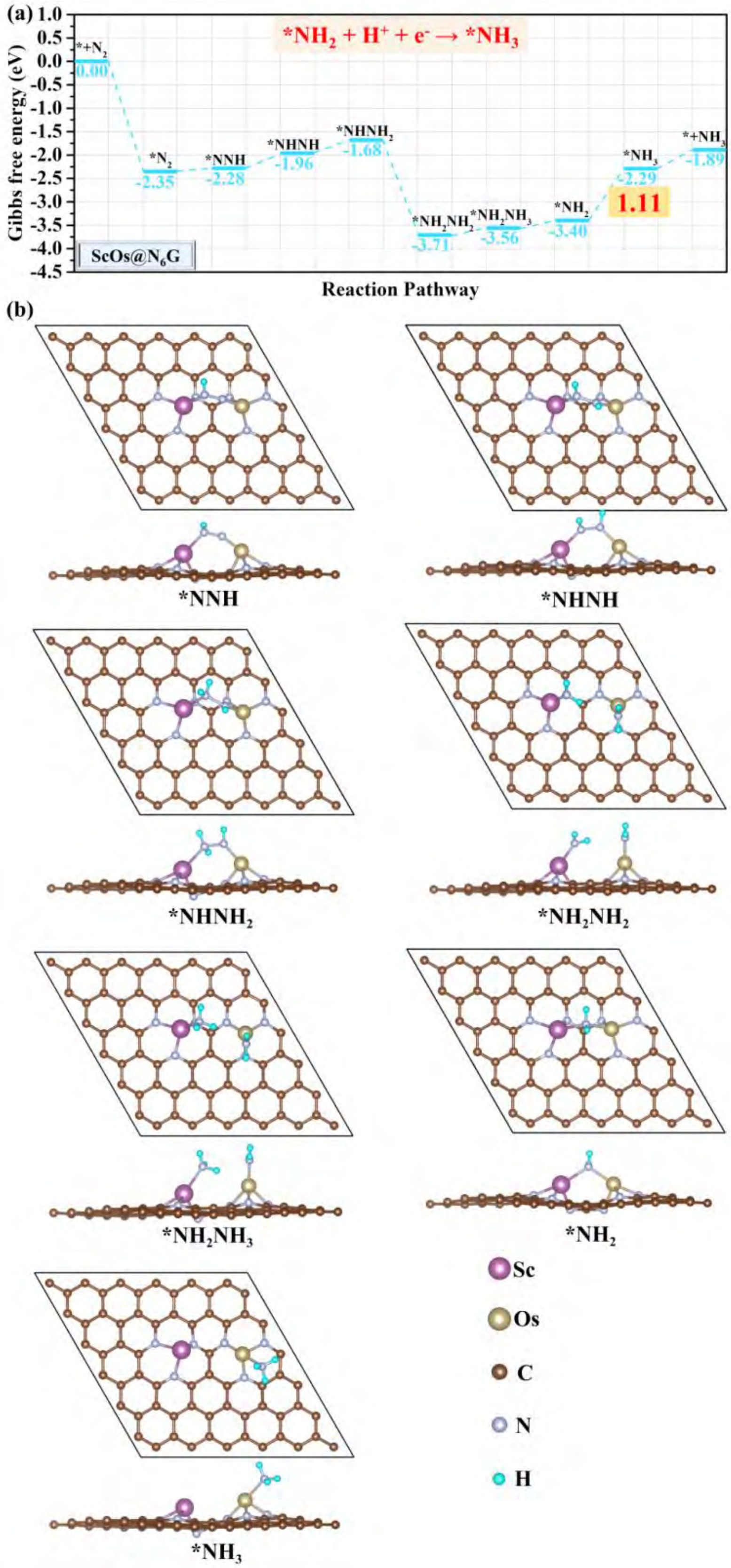

Figure S110. (a) Gibbs free energy diagram for N_2 reduction to NH_3 production on the ScOs@N_6G system. (b) Optimized structures of various intermediates along the hydrogenation pathway of N_2 reduction to NH_3 production on the ScOs@N_6G system.

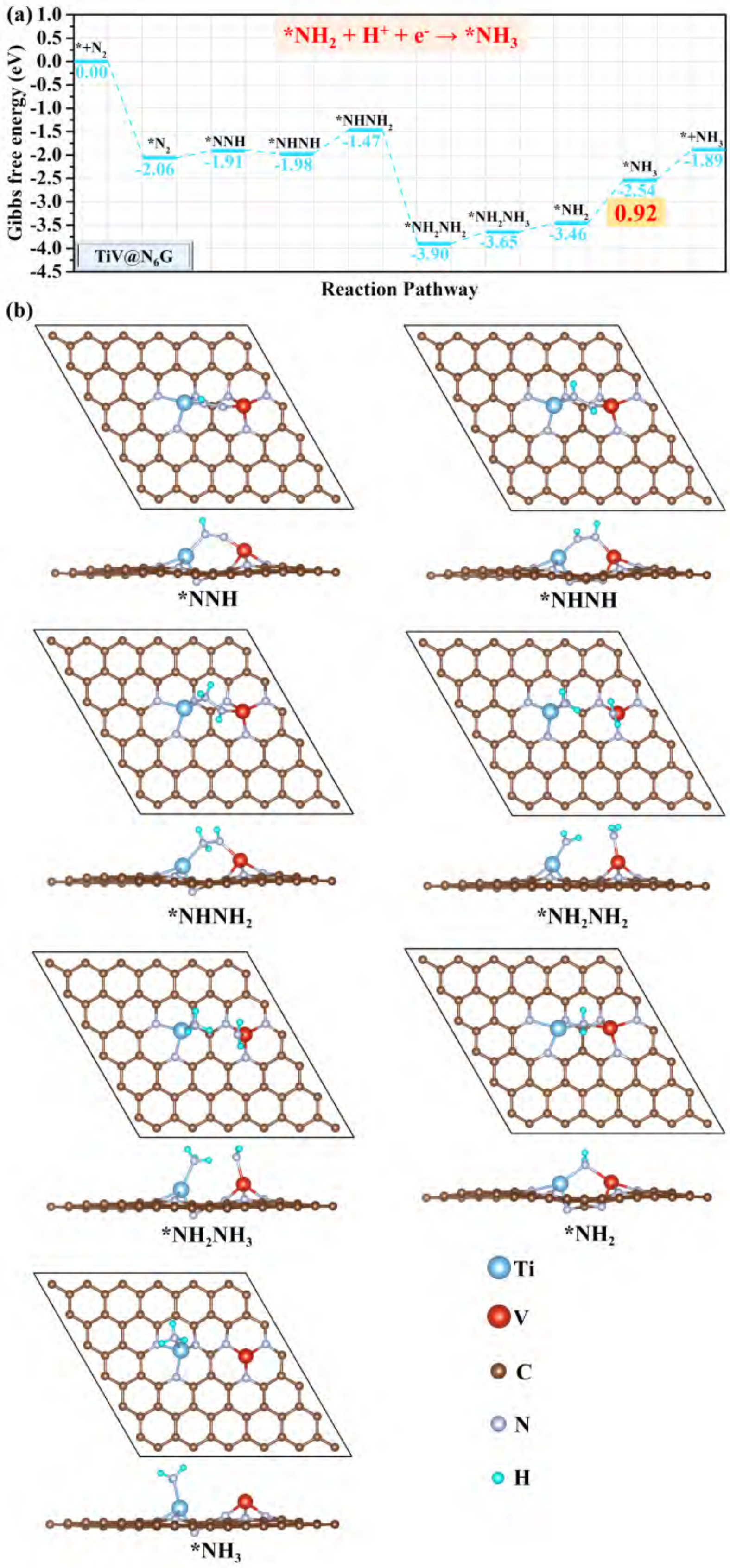

Figure S111. (a) Gibbs free energy diagram for N_2 reduction to NH_3 production on the TiV@N$_6$G system. (b) Optimized structures of various intermediates along the hydrogenation pathway of N_2 reduction to NH_3 production on the TiV@N$_6$G system.

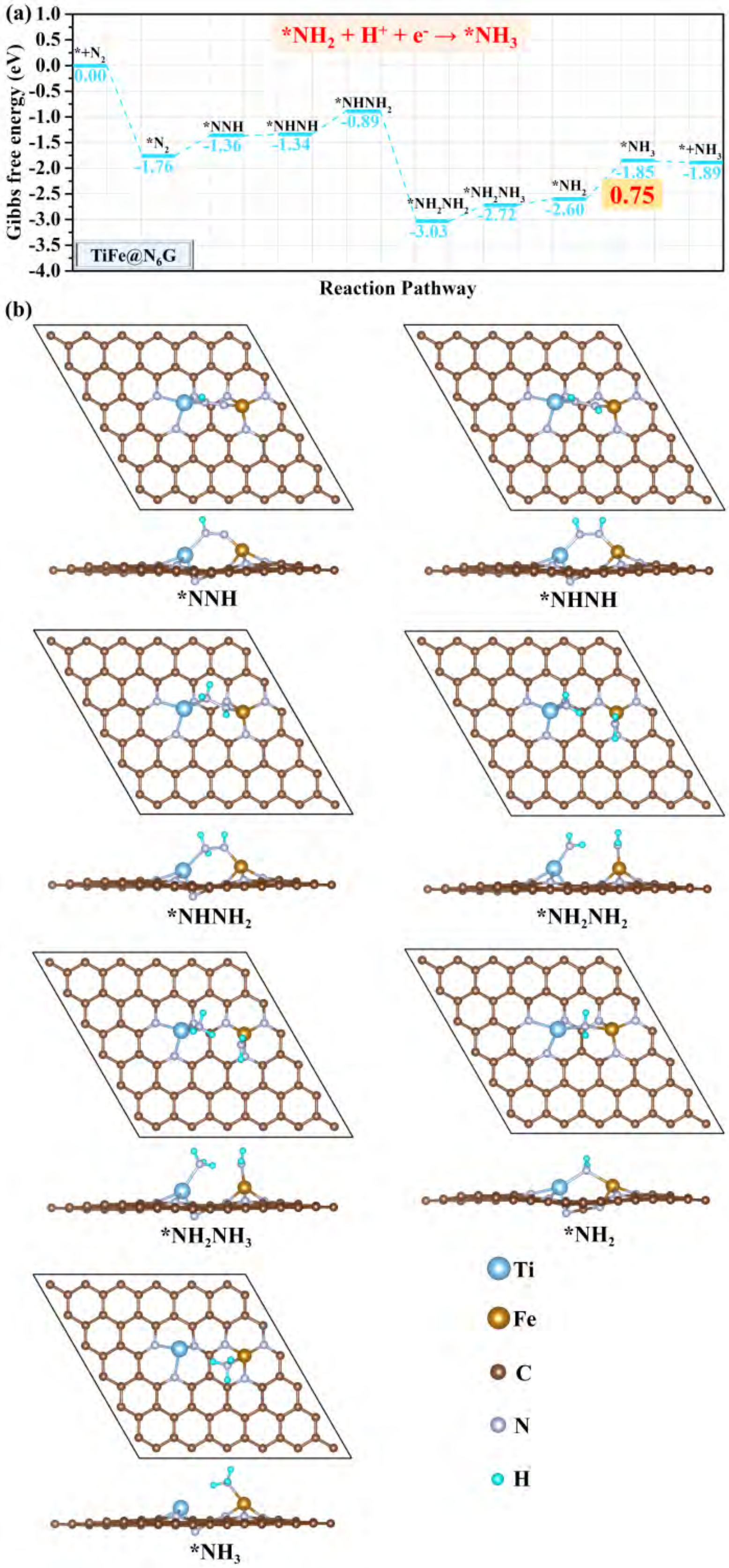

Figure S112. (a) Gibbs free energy diagram for N_2 reduction to NH_3 production on the TiFe@N$_6$G system. (b) Optimized structures of various intermediates along the hydrogenation pathway of N_2 reduction to NH_3 production on the TiFe@N$_6$G system.

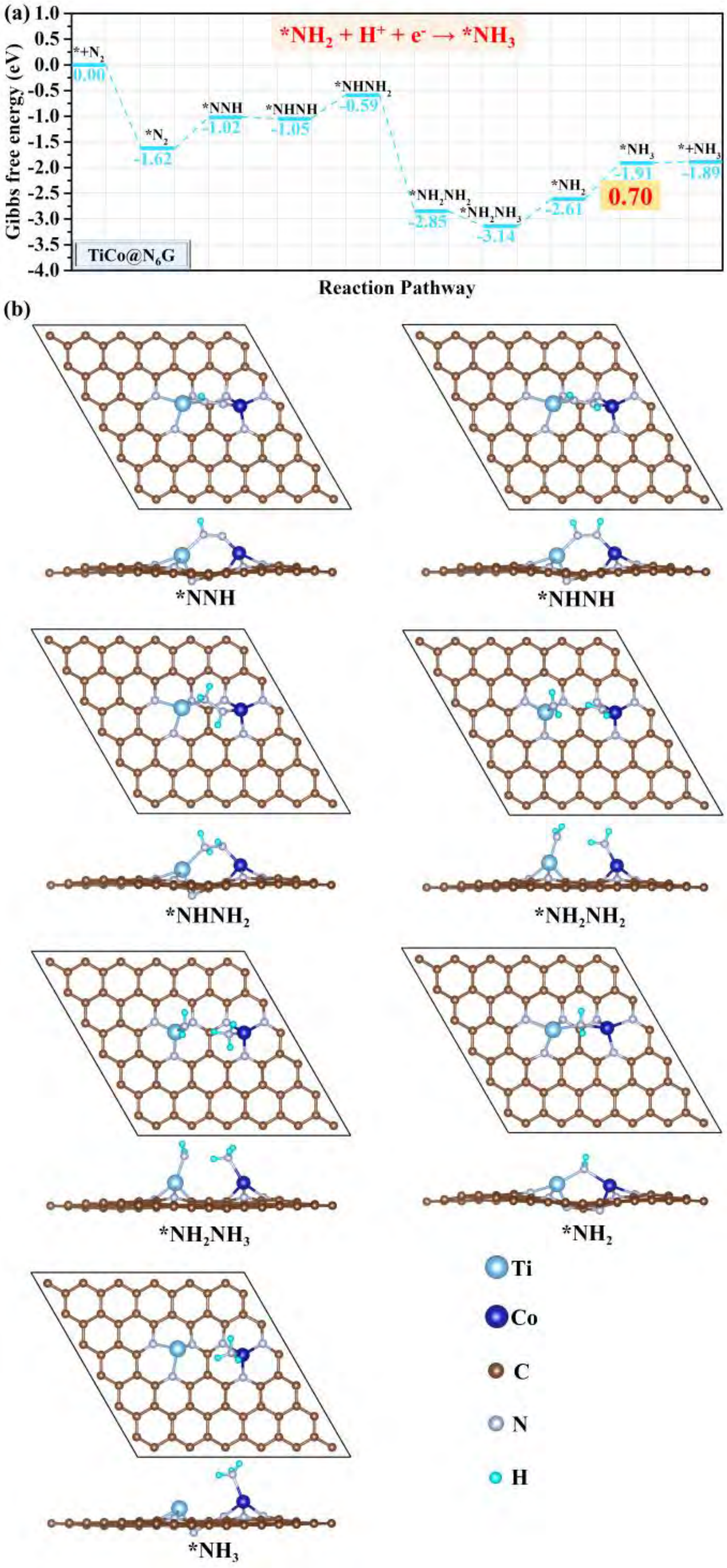

Figure S113. (a) Gibbs free energy diagram for N_2 reduction to NH_3 production on the TiCo@N_6G system. (b) Optimized structures of various intermediates along the hydrogenation pathway of N_2 reduction to NH_3 production on the TiCo@N_6G system.

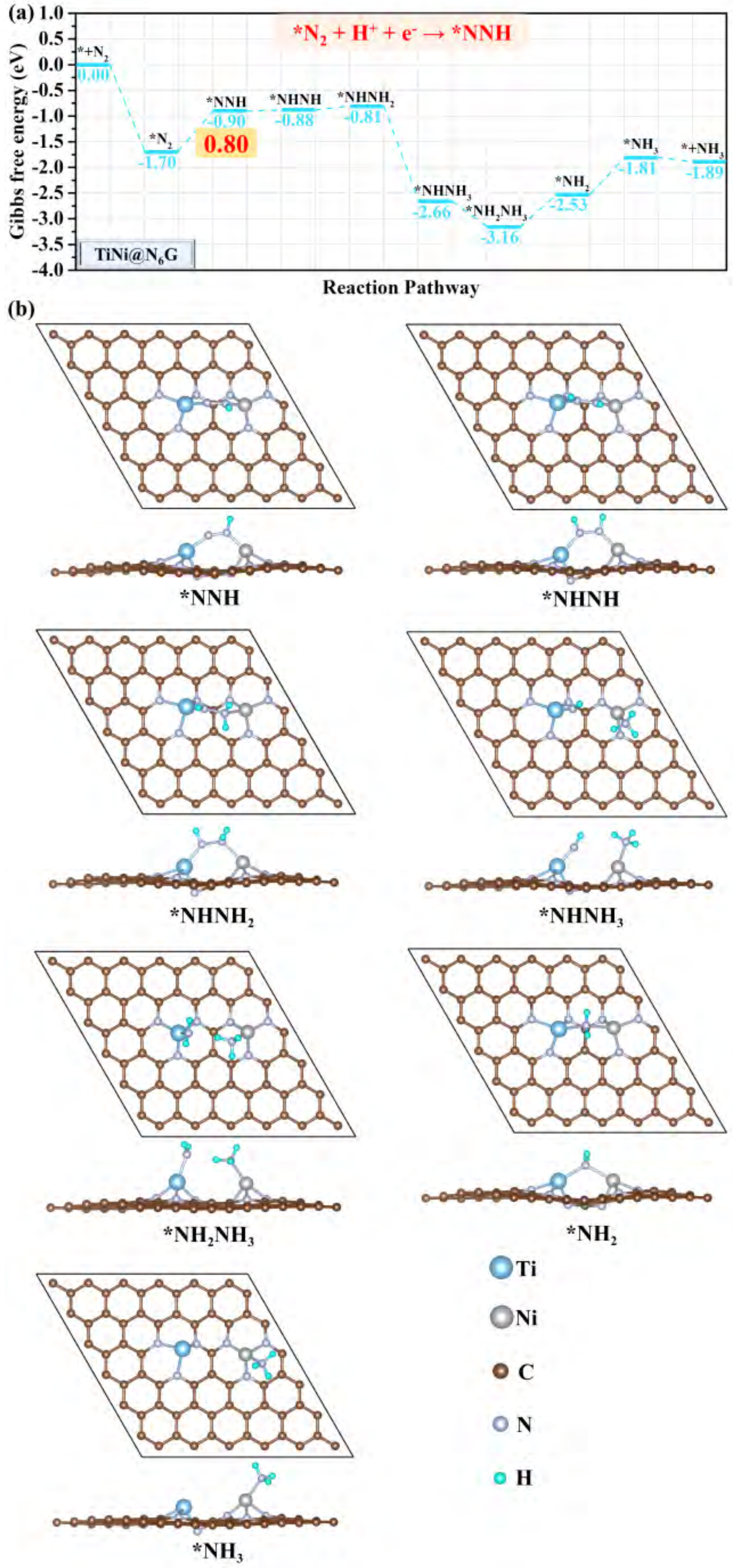

Figure S114. (a) Gibbs free energy diagram for N_2 reduction to NH_3 production on the TiNi@N_6G system. (b) Optimized structures of various intermediates along the hydrogenation pathway of N_2 reduction to NH_3 production on the TiNi@N_6G system.

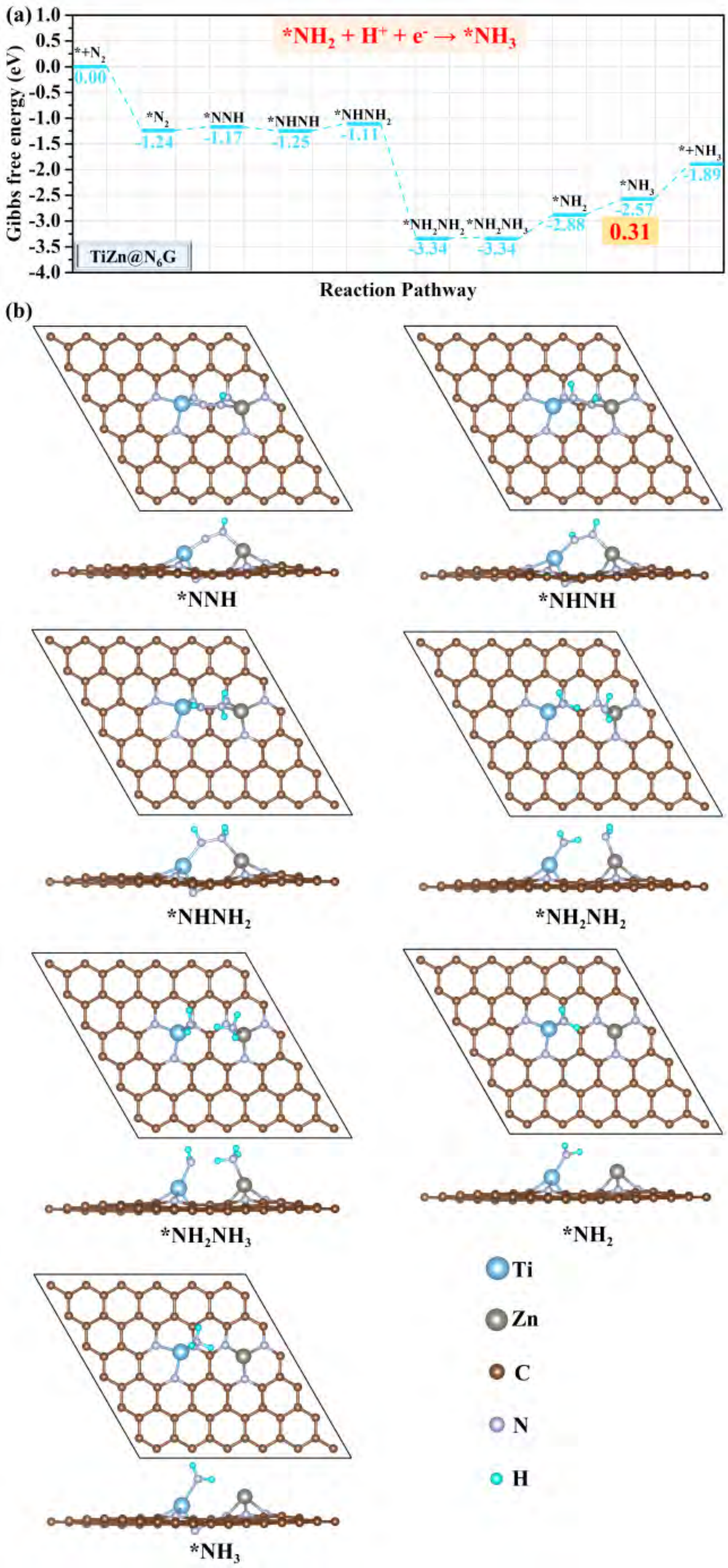

Figure S115. (a) Gibbs free energy diagram for N_2 reduction to NH_3 production on the TiZn@N_6G system. (b) Optimized structures of various intermediates along the hydrogenation pathway of N_2 reduction to NH_3 production on the TiZn@N_6G system.

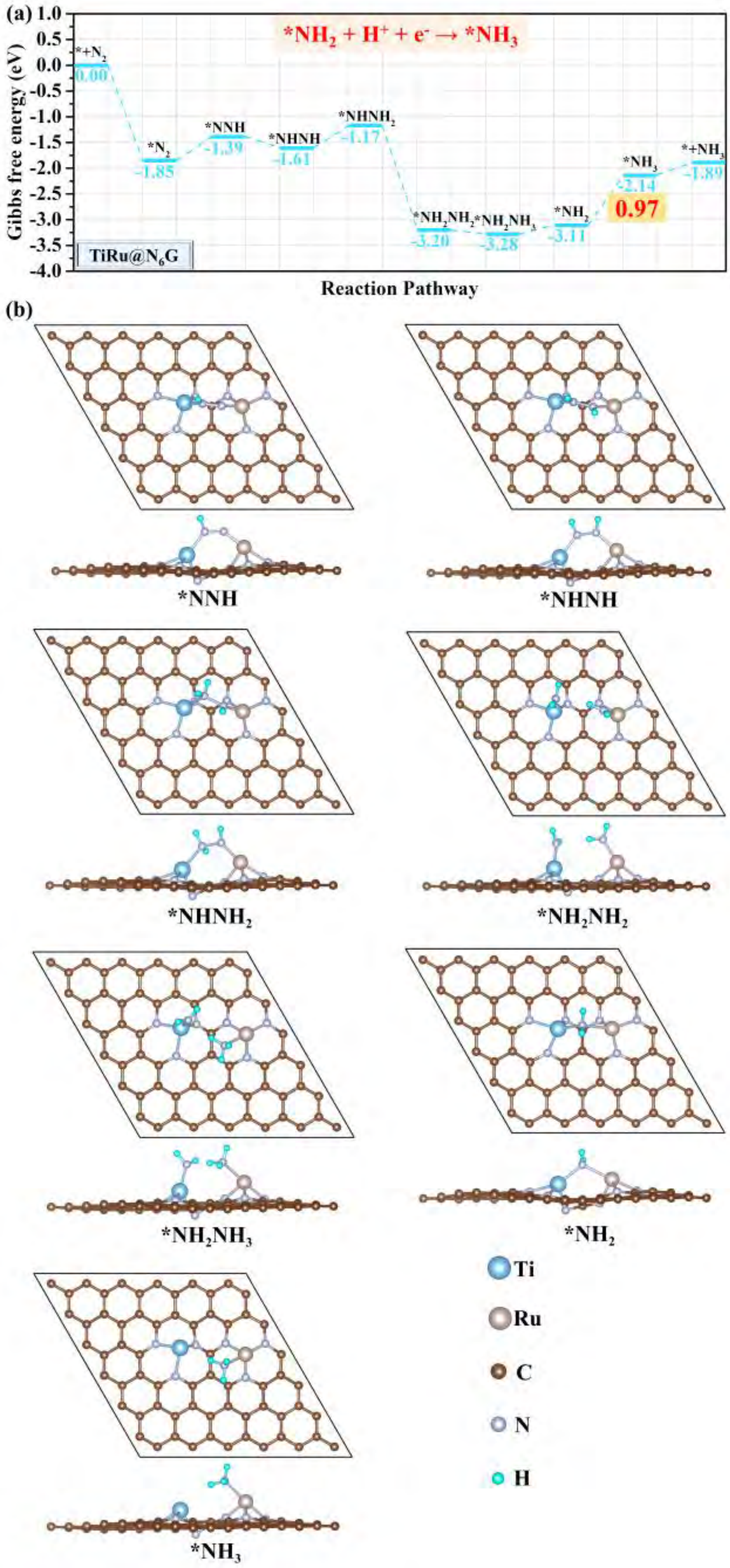

Figure S116. (a) Gibbs free energy diagram for N_2 reduction to NH_3 production on the TiRu@N_6G system. (b) Optimized structures of various intermediates along the hydrogenation pathway of N_2 reduction to NH_3 production on the TiRu@N_6G system.

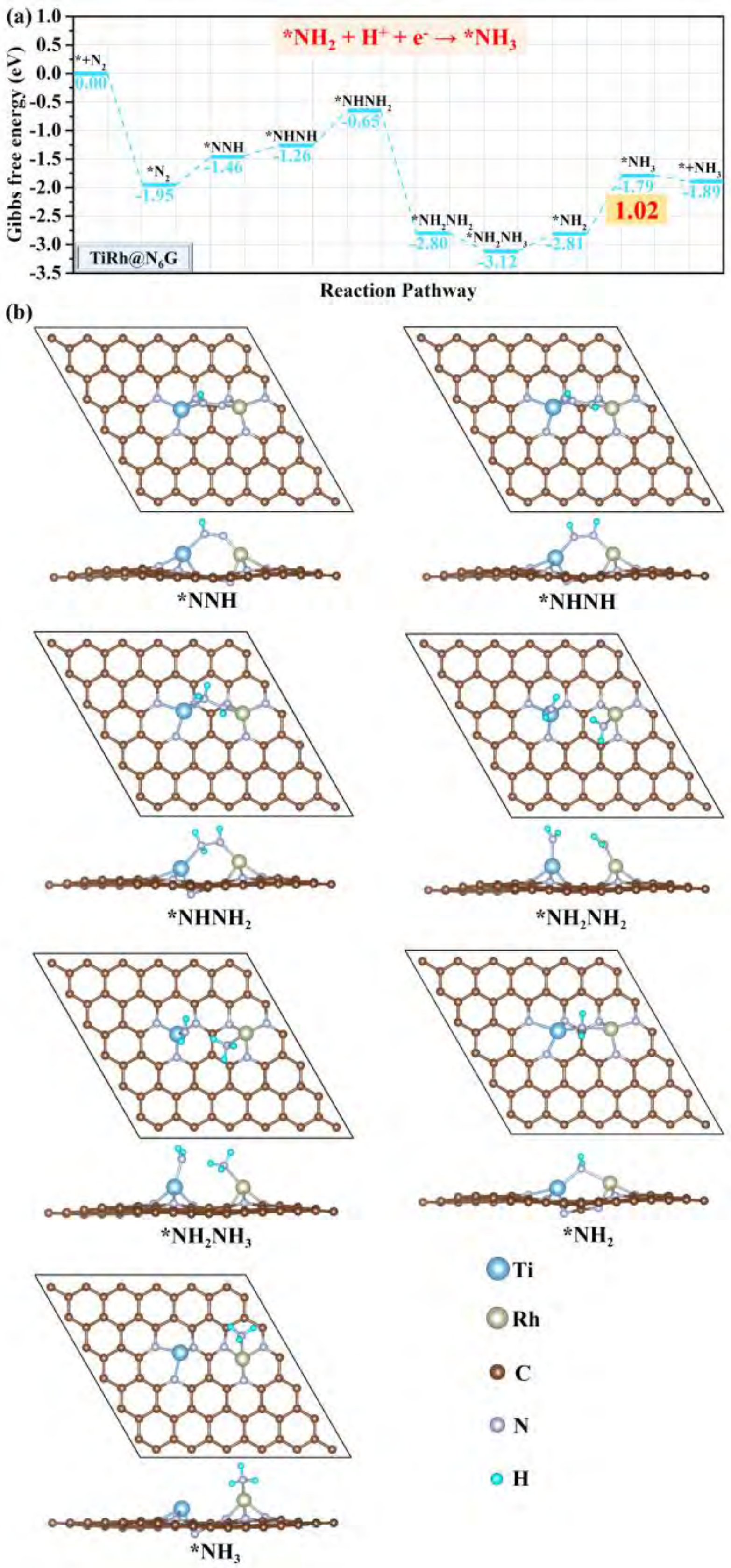

Figure S117. (a) Gibbs free energy diagram for N_2 reduction to NH_3 production on the TiRh@N_6G system. (b) Optimized structures of various intermediates along the hydrogenation pathway of N_2 reduction to NH_3 production on the TiRh@N_6G system.

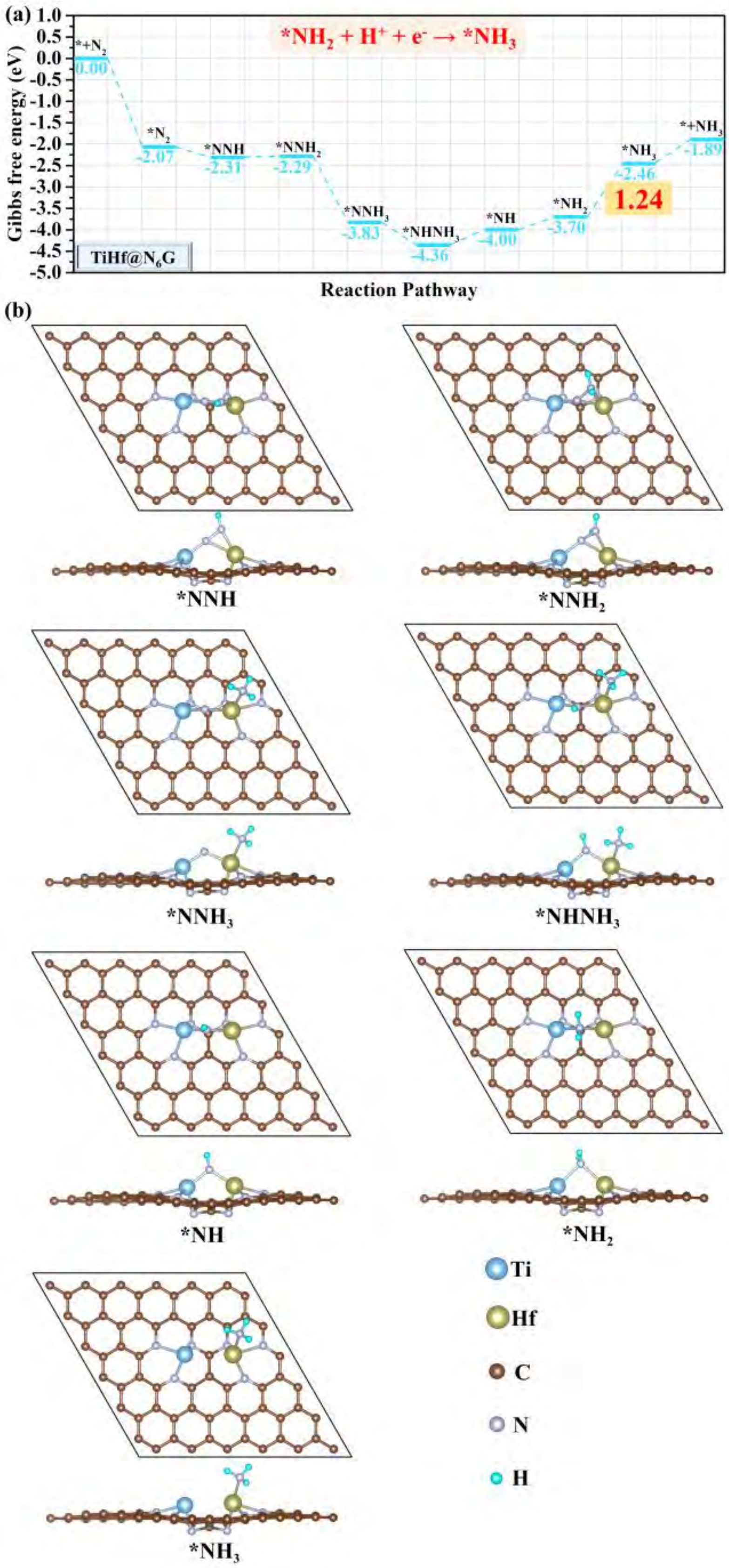

Figure S118. (a) Gibbs free energy diagram for N_2 reduction to NH_3 production on the TiHf@N_6G system. (b) Optimized structures of various intermediates along the hydrogenation pathway of N_2 reduction to NH_3 production on the TiHf@N_6G system.

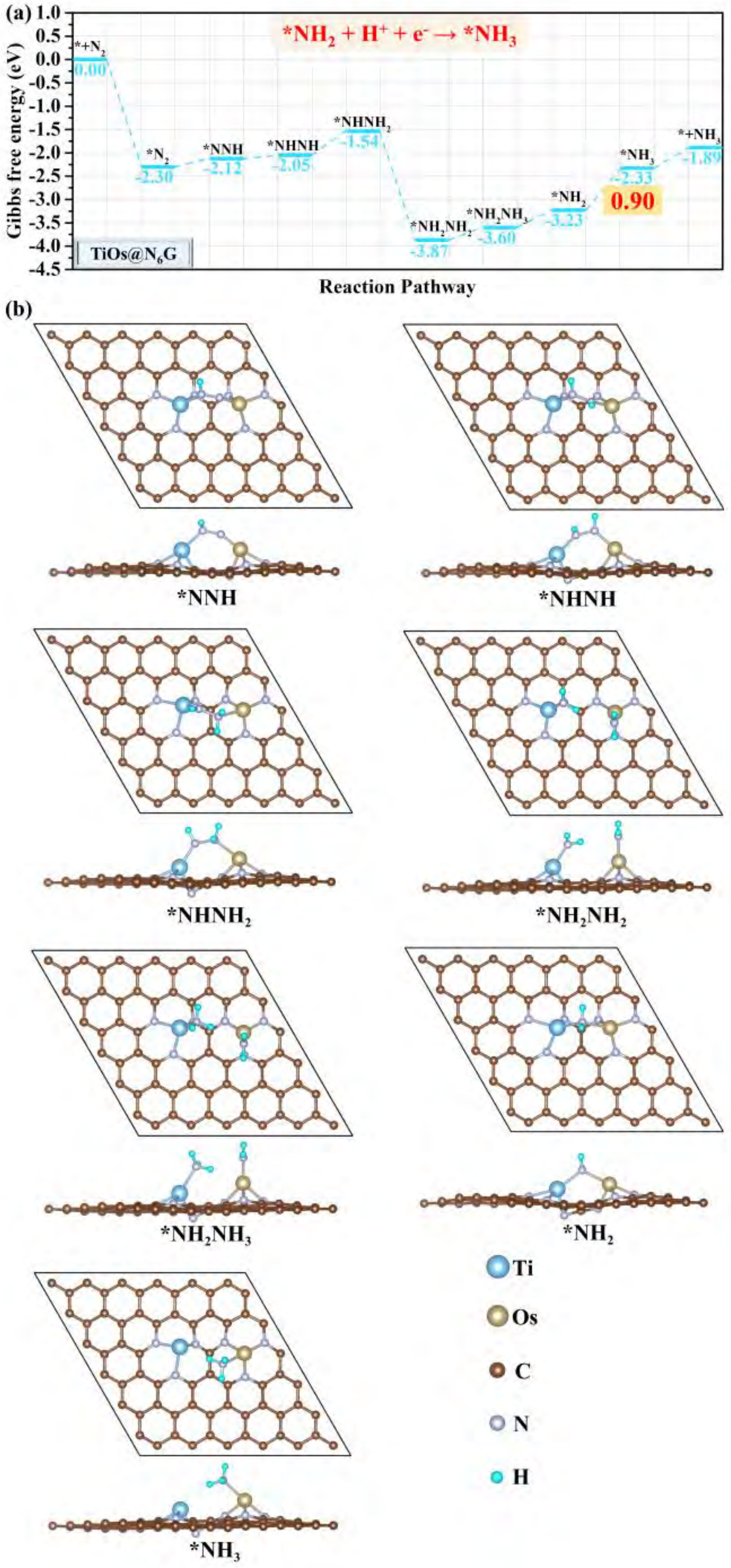

Figure S119. (a) Gibbs free energy diagram for N_2 reduction to NH_3 production on the TiOs@N_6G system. (b) Optimized structures of various intermediates along the hydrogenation pathway of N_2 reduction to NH_3 production on the TiOs@N_6G system.

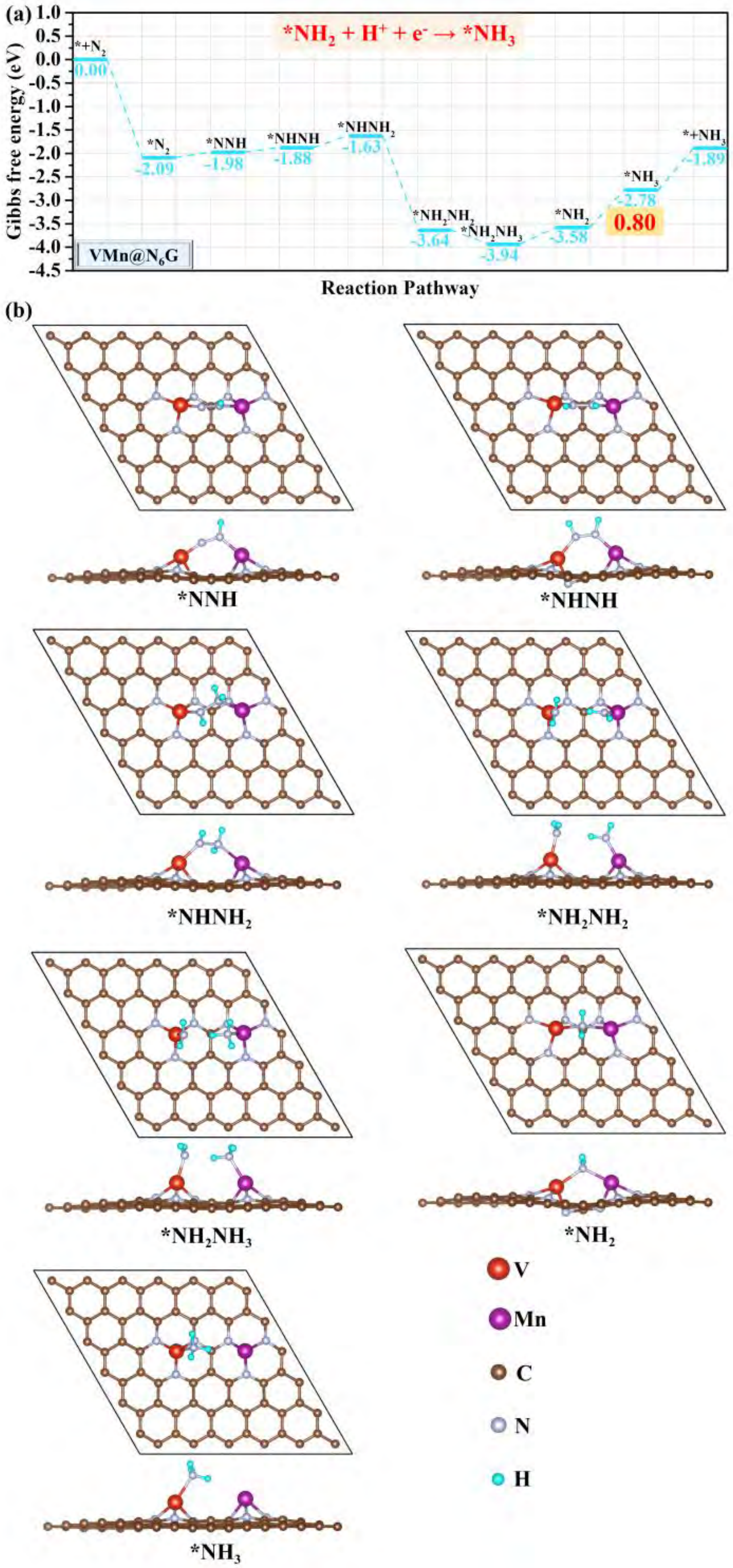

Figure S120. (a) Gibbs free energy diagram for N_2 reduction to NH_3 production on the VMn@N_6G system. (b) Optimized structures of various intermediates along the hydrogenation pathway of N_2 reduction to NH_3 production on the VMn@N_6G system.

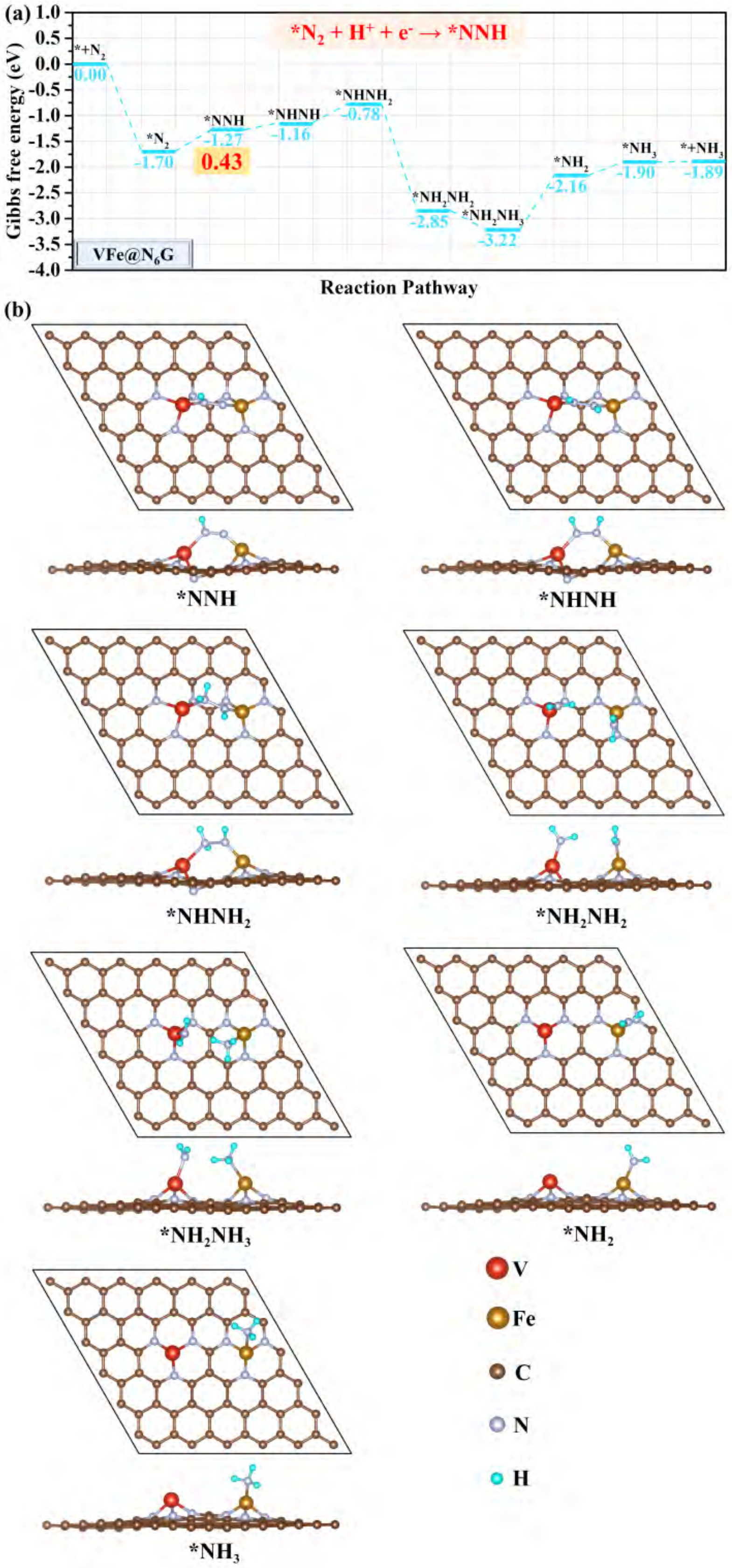

Figure S121. (a) Gibbs free energy diagram for N_2 reduction to NH_3 production on the VFe@N_6G system. (b) Optimized structures of various intermediates along the hydrogenation pathway of N_2 reduction to NH_3 production on the VFe@N_6G system.

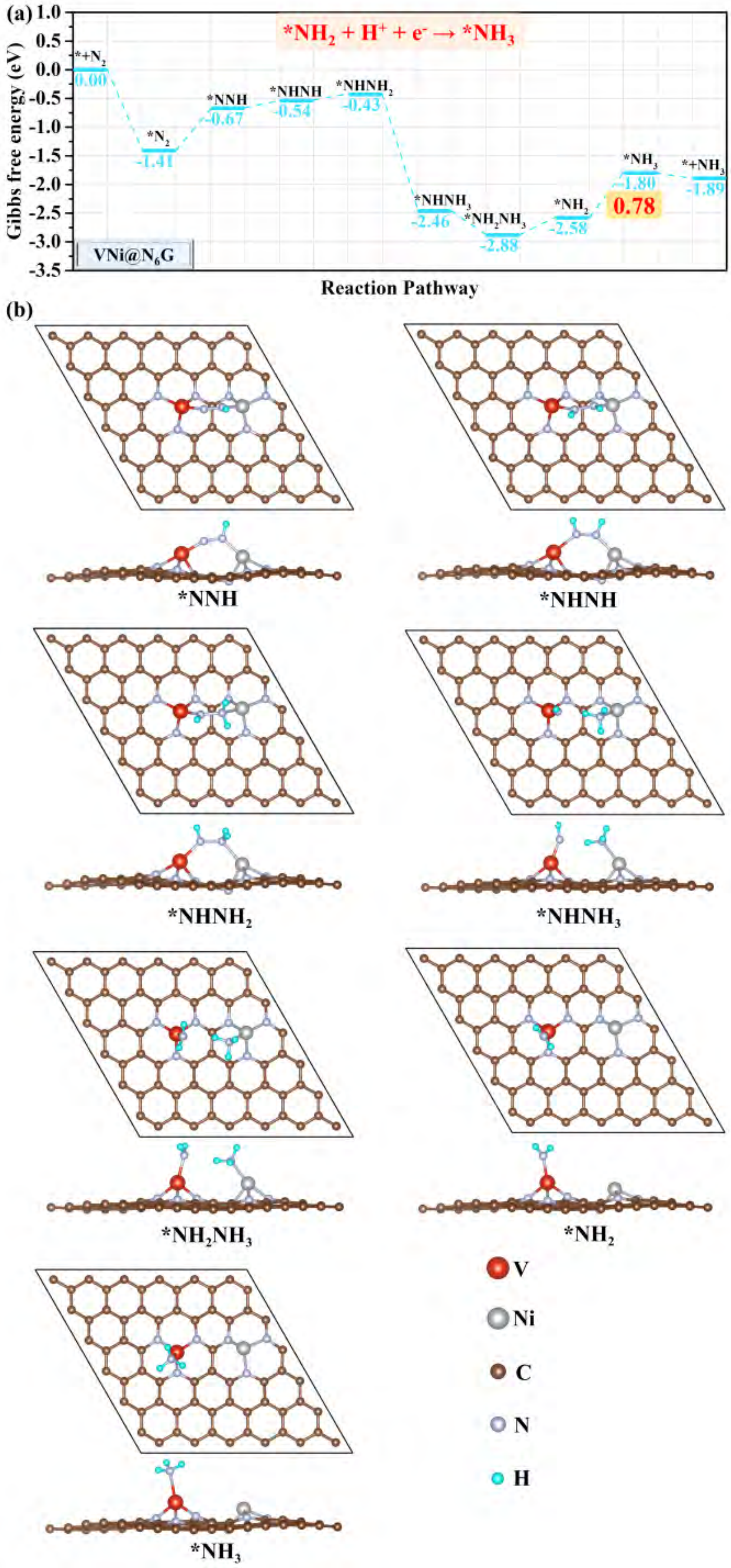

Figure S122. (a) Gibbs free energy diagram for N_2 reduction to NH_3 production on the VNi@N$_6$G system. (b) Optimized structures of various intermediates along the hydrogenation pathway of N_2 reduction to NH_3 production on the VNi@N$_6$G system.

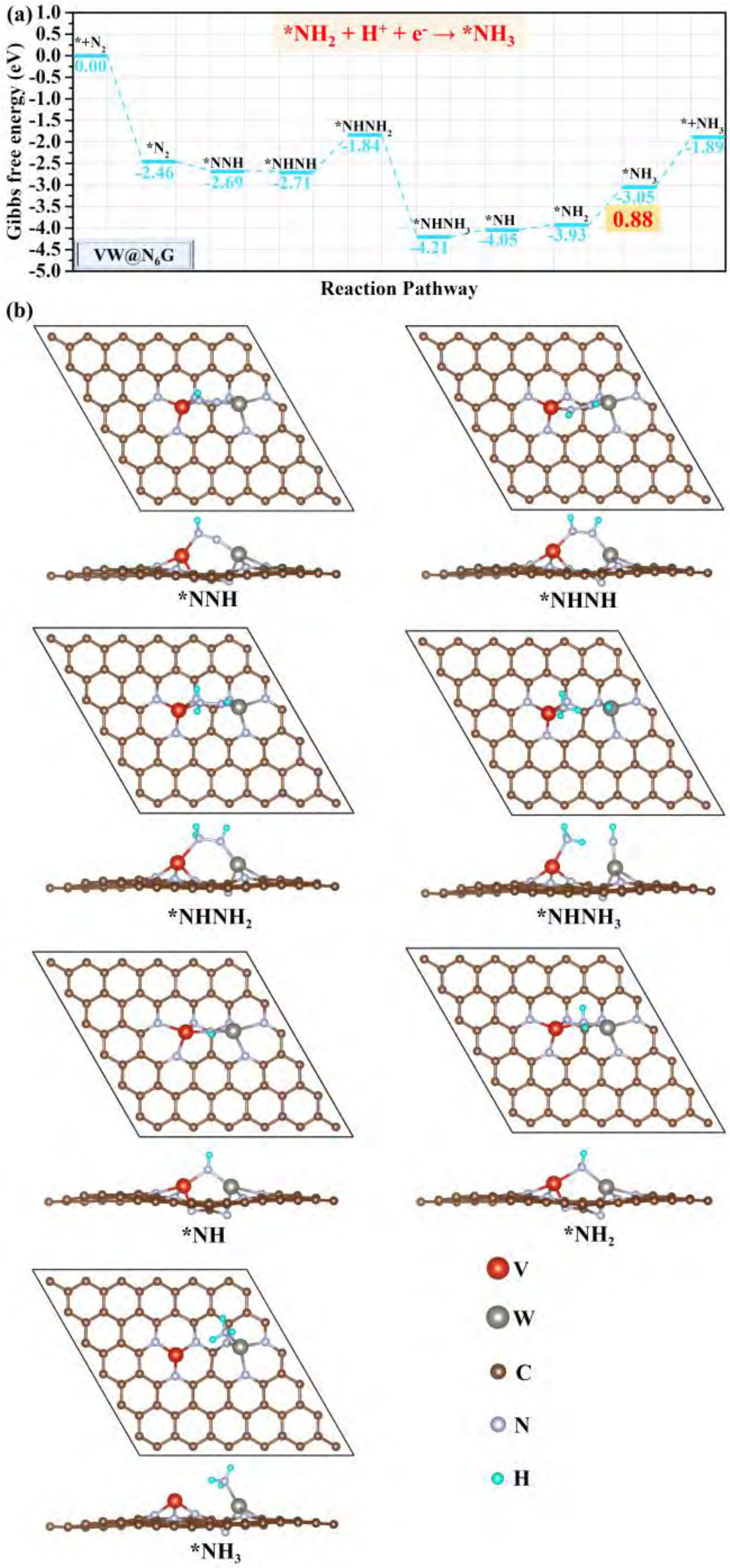

Figure S123. (a) Gibbs free energy diagram for N_2 reduction to NH_3 production on the VW@N_6G system. (b) Optimized structures of various intermediates along the hydrogenation pathway of N_2 reduction to NH_3 production on the VW@N_6G system.

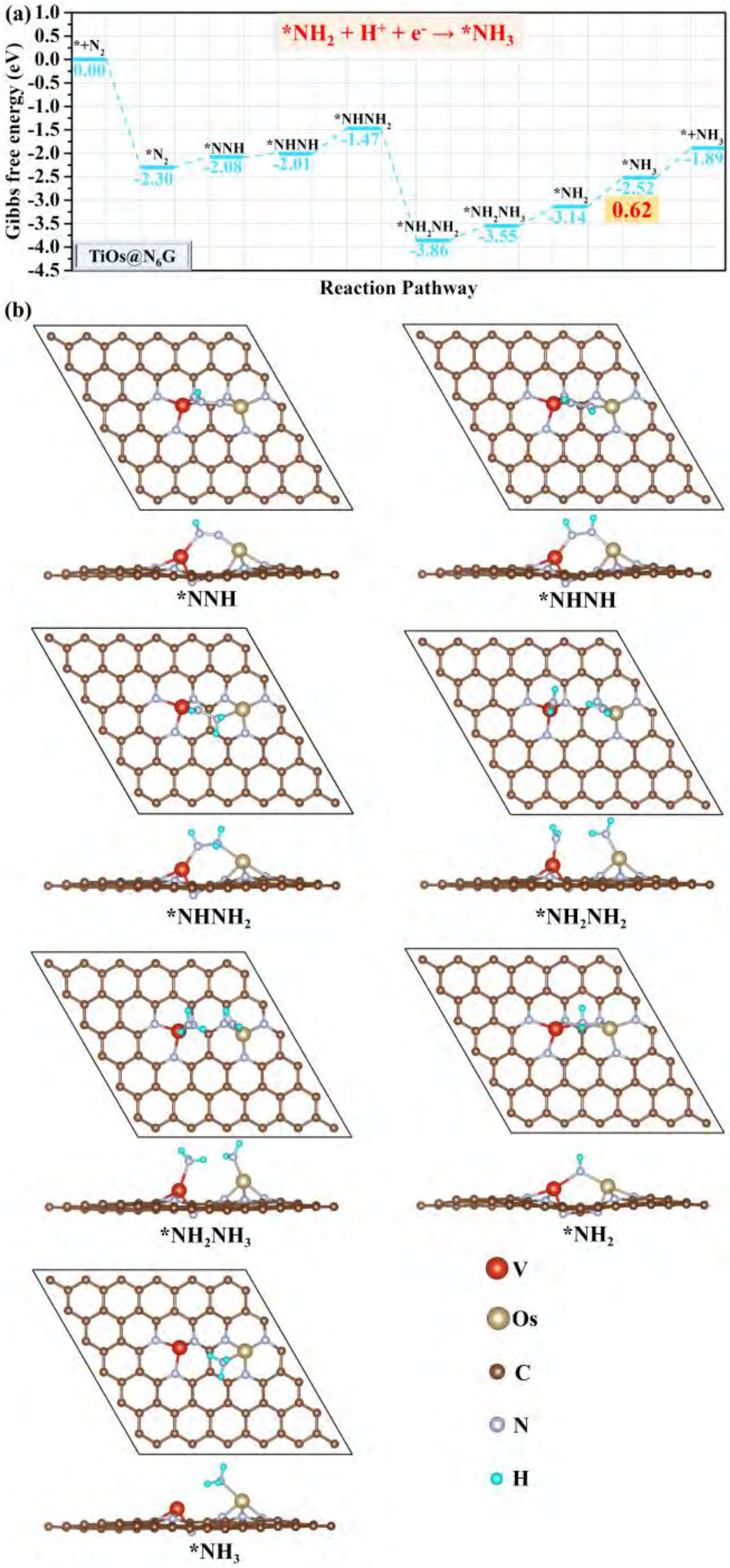

Figure S124. (a) Gibbs free energy diagram for N_2 reduction to NH_3 production on the VOs@N_6G system. (b) Optimized structures of various intermediates along the hydrogenation pathway of N_2 reduction to NH_3 production on the VOs@N_6G system.

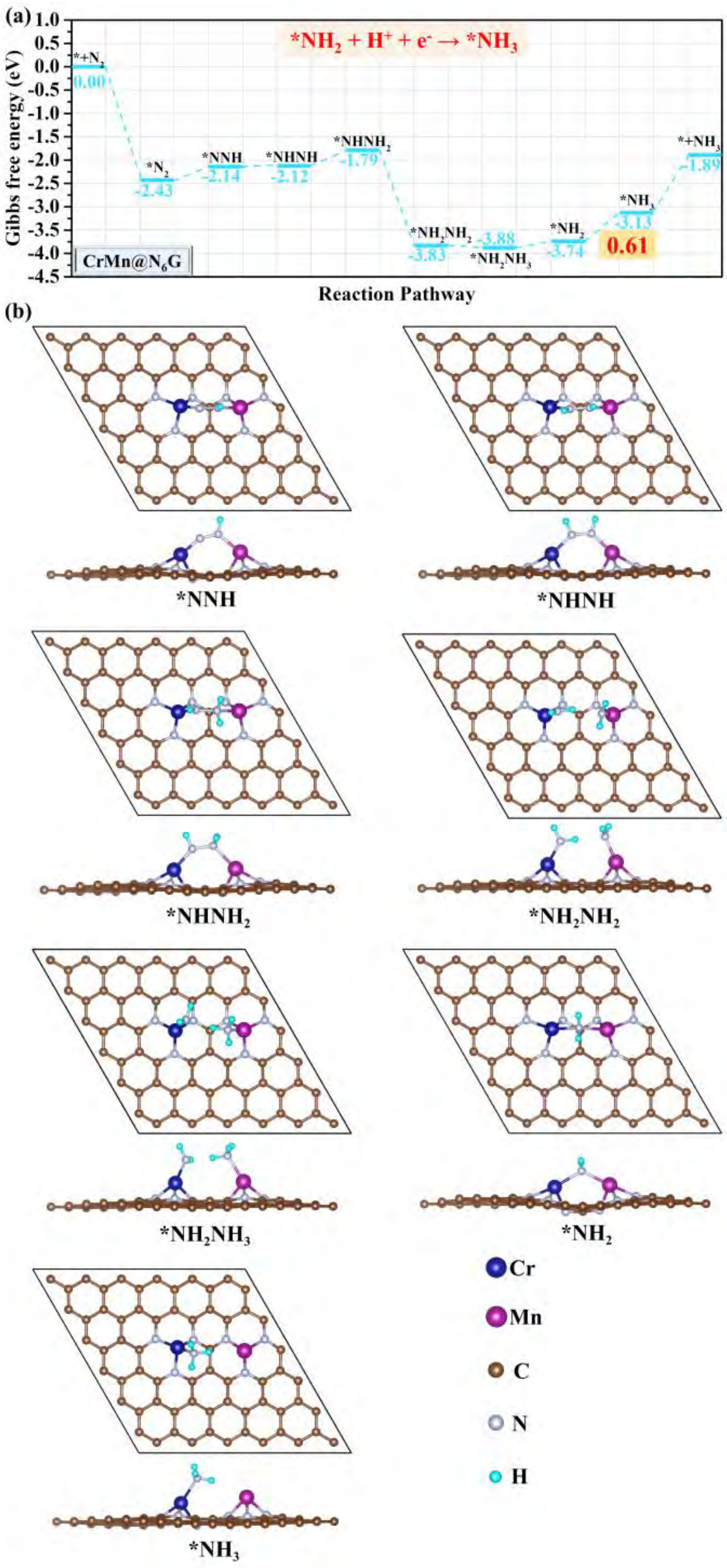

Figure S125. (a) Gibbs free energy diagram for N_2 reduction to NH_3 production on the CrMn@N_6G system. (b) Optimized structures of various intermediates along the hydrogenation pathway of N_2 reduction to NH_3 on the CrMn@N_6G system.

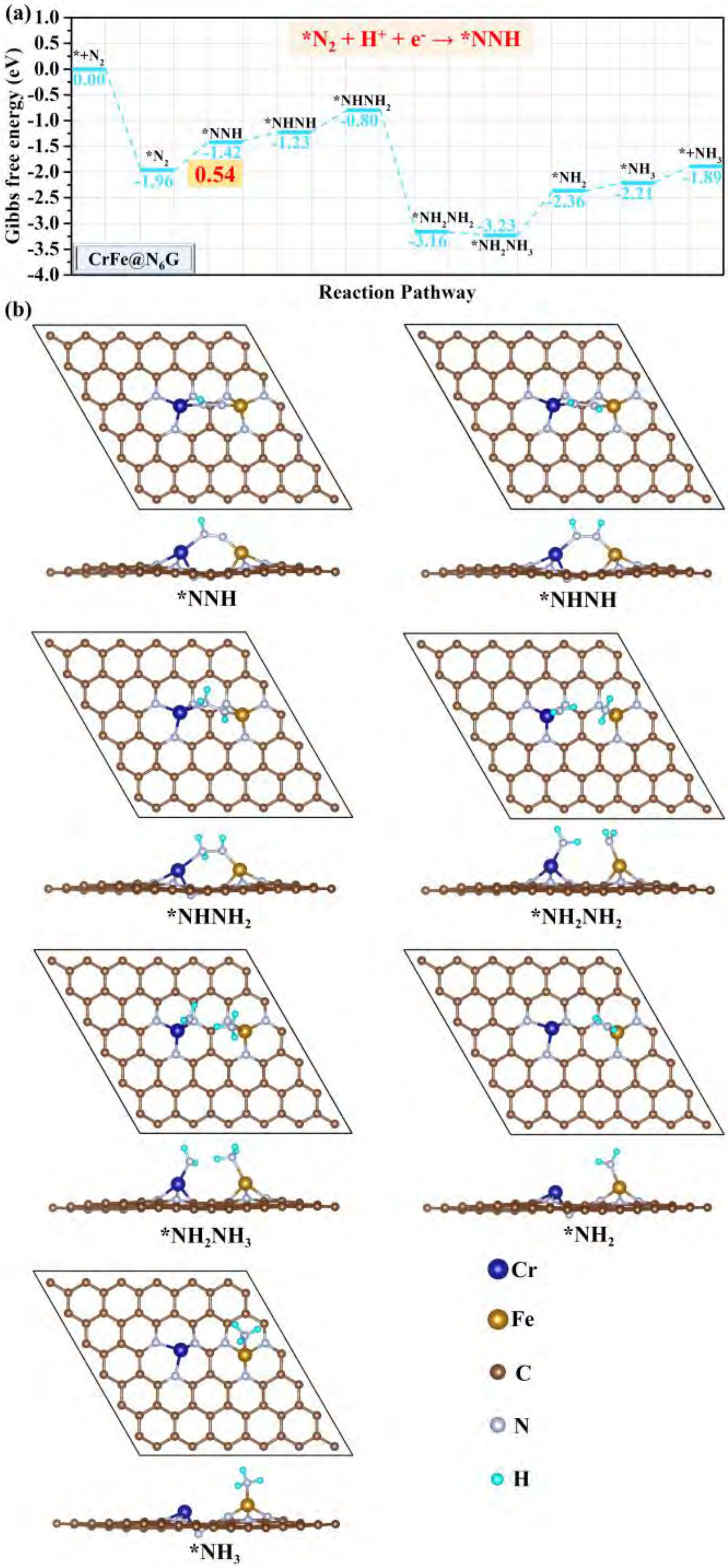

Figure S126. (a) Gibbs free energy diagram for N_2 reduction to NH_3 production on the CrFe@N_6G system. (b) Optimized structures of various intermediates along the hydrogenation pathway of N_2 reduction to NH_3 production on the CrFe@N_6G system.

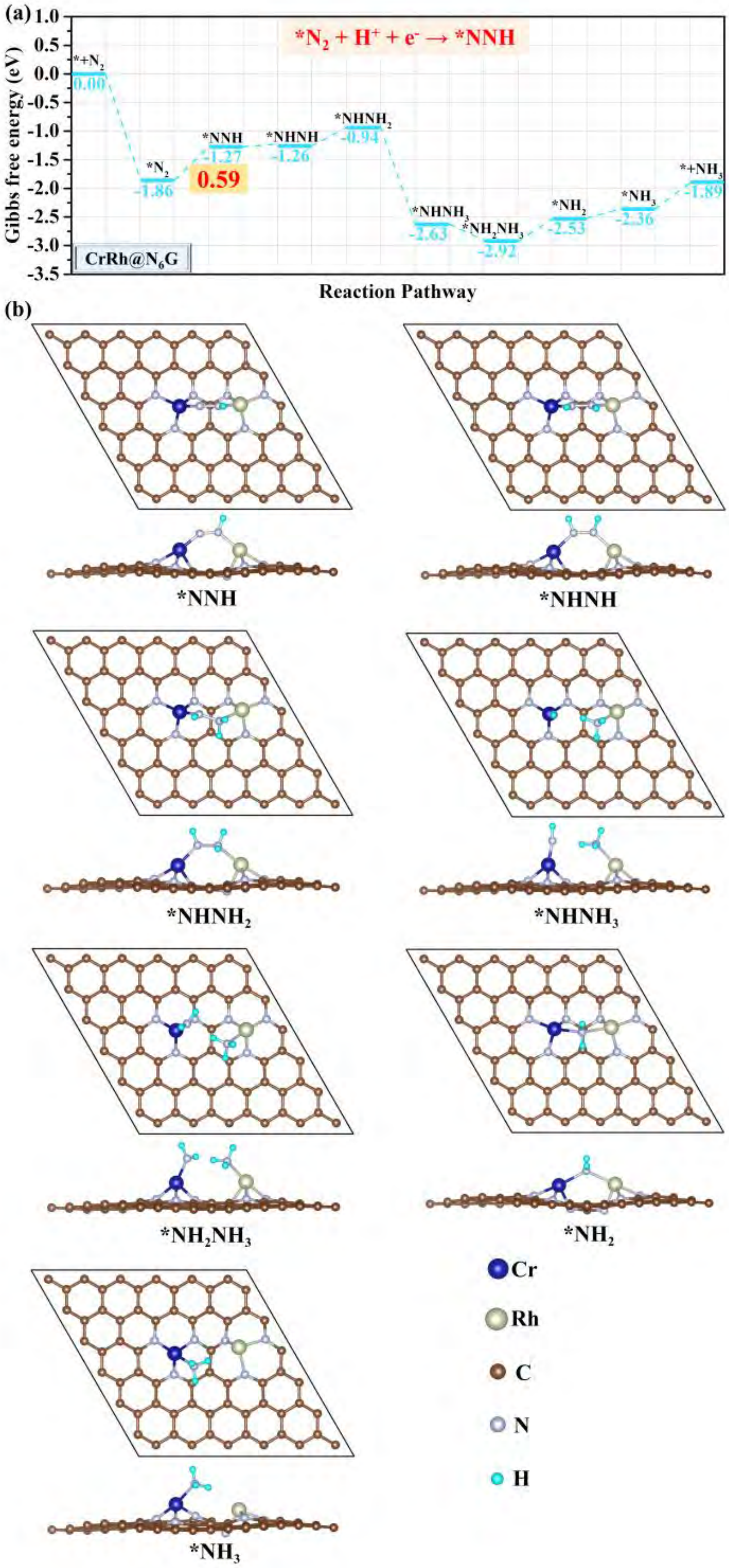

Figure S127. (a) Gibbs free energy diagram for N_2 reduction to NH_3 production on the CrRh@N_6G system. (b) Optimized structures of various intermediates along the hydrogenation pathway of N_2 reduction to NH_3 production on the CrRh@N_6G system.

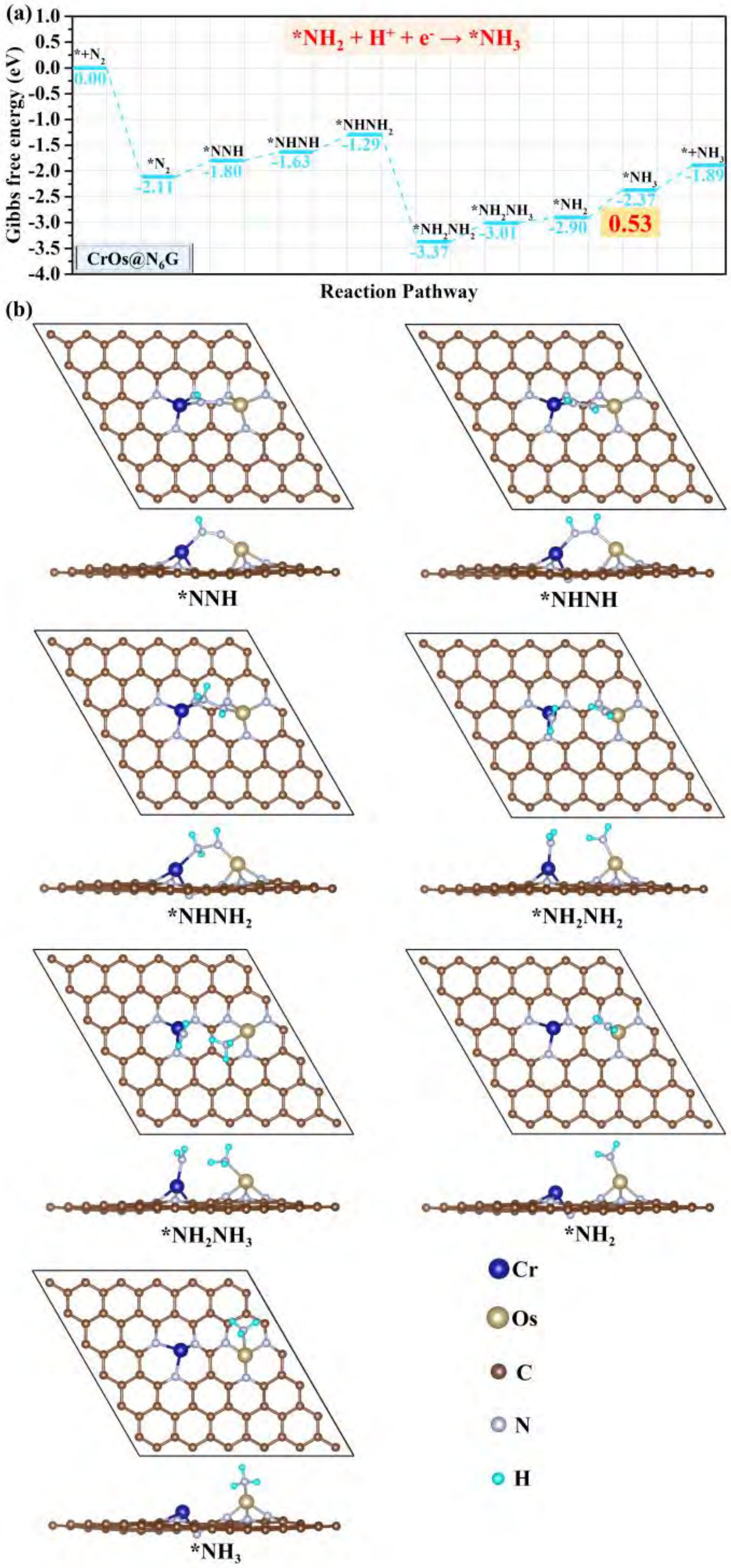

Figure S128. (a) Gibbs free energy diagram for N_2 reduction to NH_3 production on the CrOs@N_6G system. (b) Optimized structures of various intermediates along the hydrogenation pathway of N_2 reduction to NH_3 production on the CrOs@N_6G system.

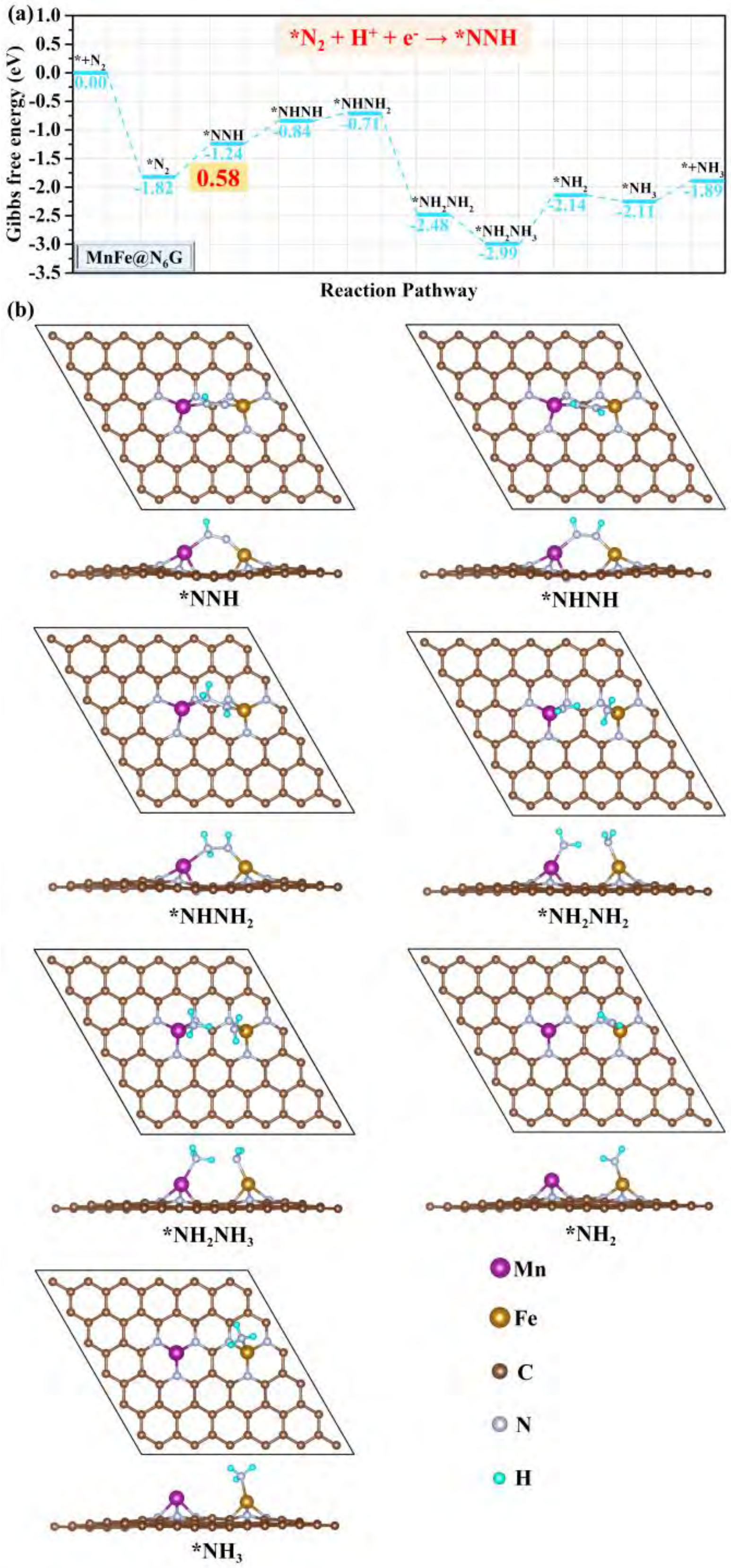

Figure S129. (a) Gibbs free energy diagram for N_2 reduction to NH_3 production on the MnFe@N_6G system. (b) Optimized structures of various intermediates along the hydrogenation pathway of N_2 reduction to NH_3 on the MnFe@N_6G system.

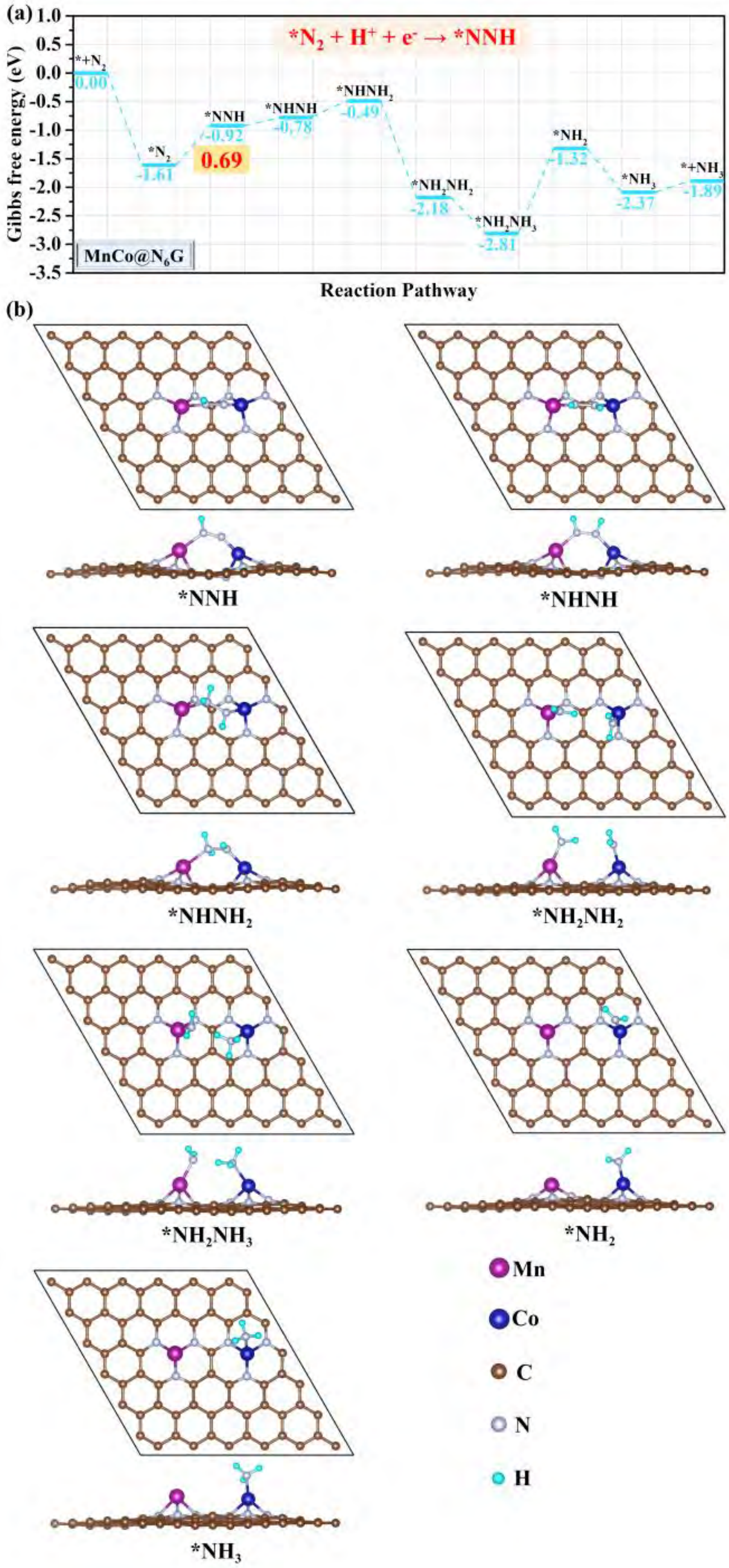

Figure S130. (a) Gibbs free energy diagram for N_2 reduction to NH_3 production on the MnCo@N_6G system. (b) Optimized structures of various intermediates along the hydrogenation pathway of N_2 reduction to NH_3 on the MnCo@N_6G system.

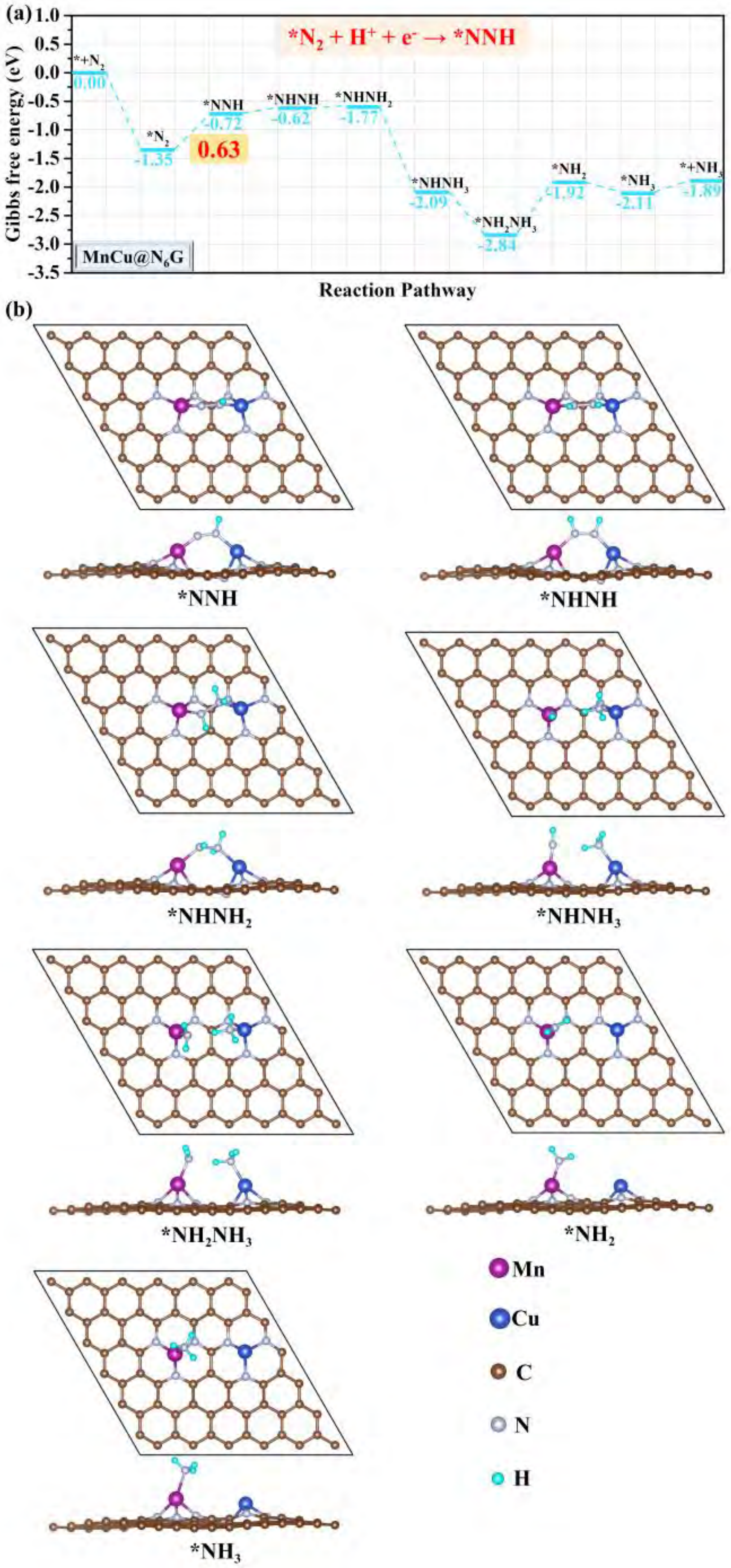

Figure S131. (a) Gibbs free energy diagram for N_2 reduction to NH_3 production on the MnCu@N_6G system. (b) Optimized structures of various intermediates along the hydrogenation pathway of N_2 reduction to NH_3 on the MnCu@N_6G system.

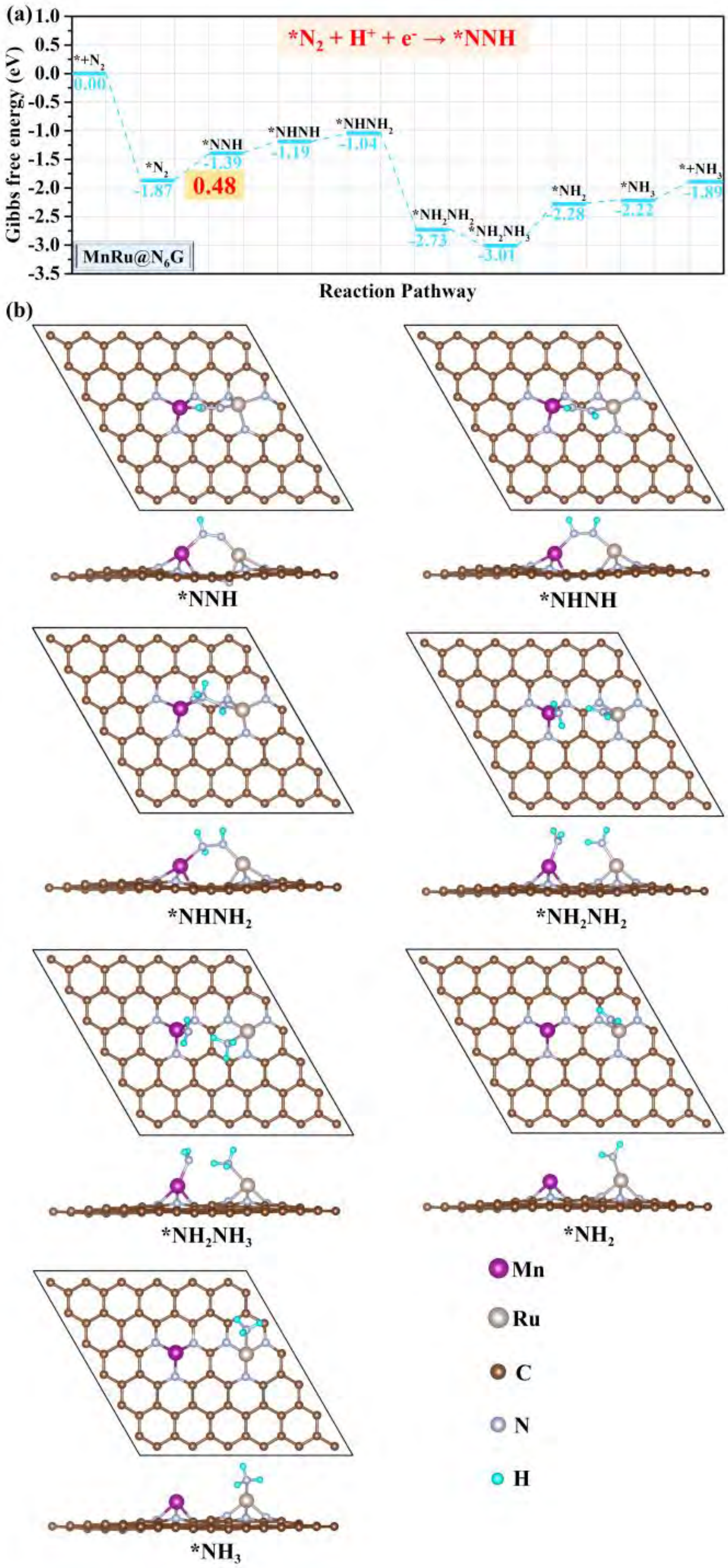

Figure S132. (a) Gibbs free energy diagram for N_2 reduction to NH_3 production on the MnRu@N_6G system. (b) Optimized structures of various intermediates along the hydrogenation pathway of N_2 reduction to NH_3 on the MnRu@N_6G system.

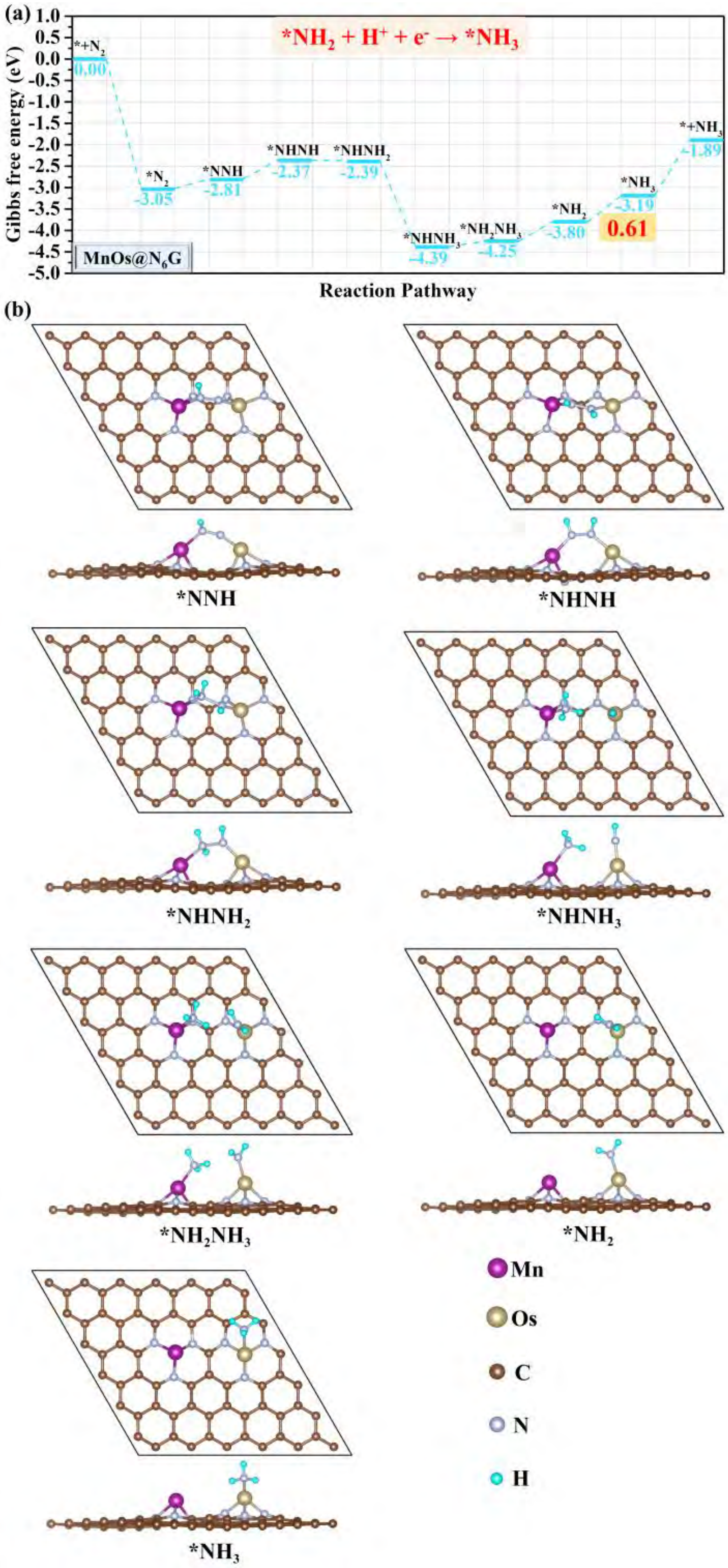

Figure S133. (a) Gibbs free energy diagram for N_2 reduction to NH_3 production on the MnOs@N_6G system. (b) Optimized structures of various intermediates along the hydrogenation pathway of N_2 reduction to NH_3 on the MnOs@N_6G system.

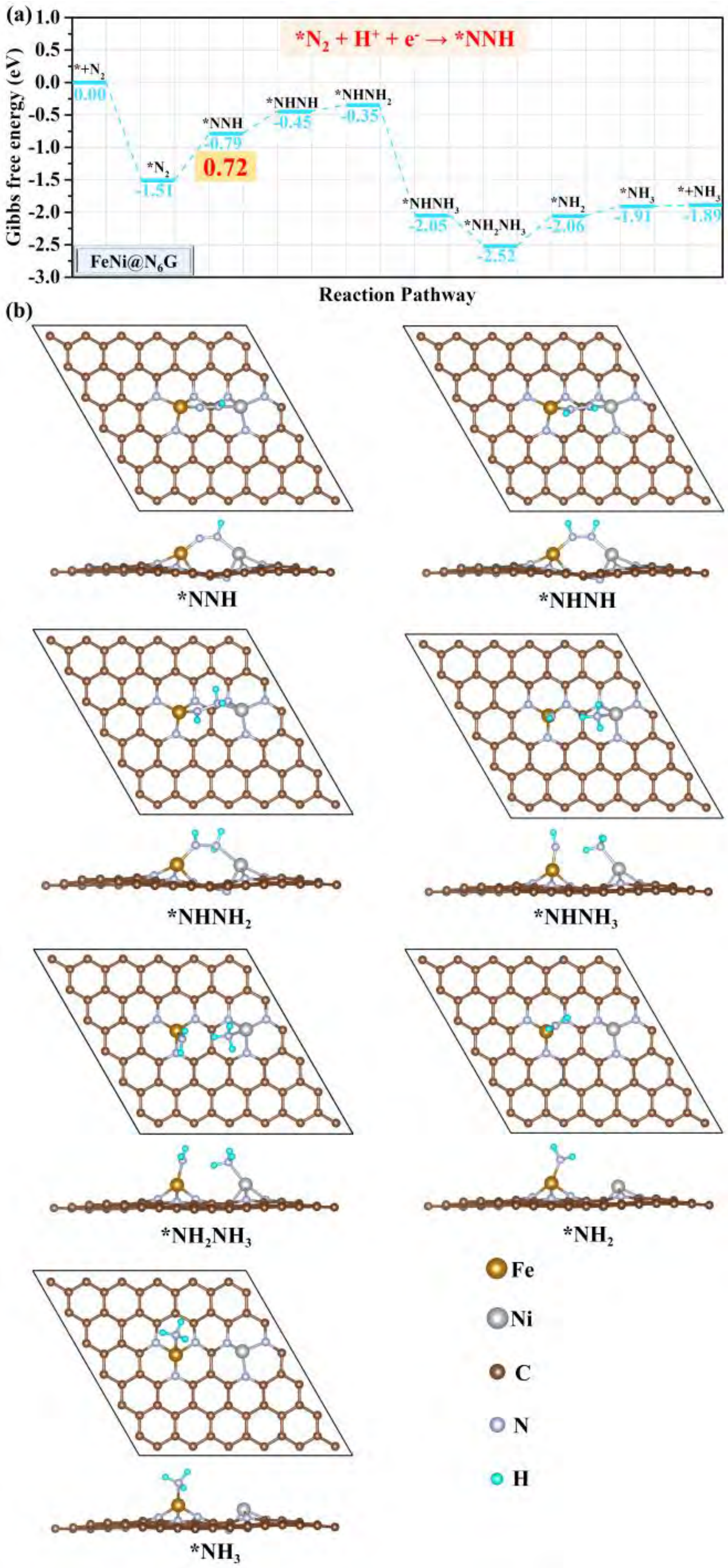

Figure S134. (a) Gibbs free energy diagram for N_2 reduction to NH_3 production on the FeNi@N_6G system. (b) Optimized structures of various intermediates along the hydrogenation pathway of N_2 reduction to NH_3 production on the FeNi@N_6G system.

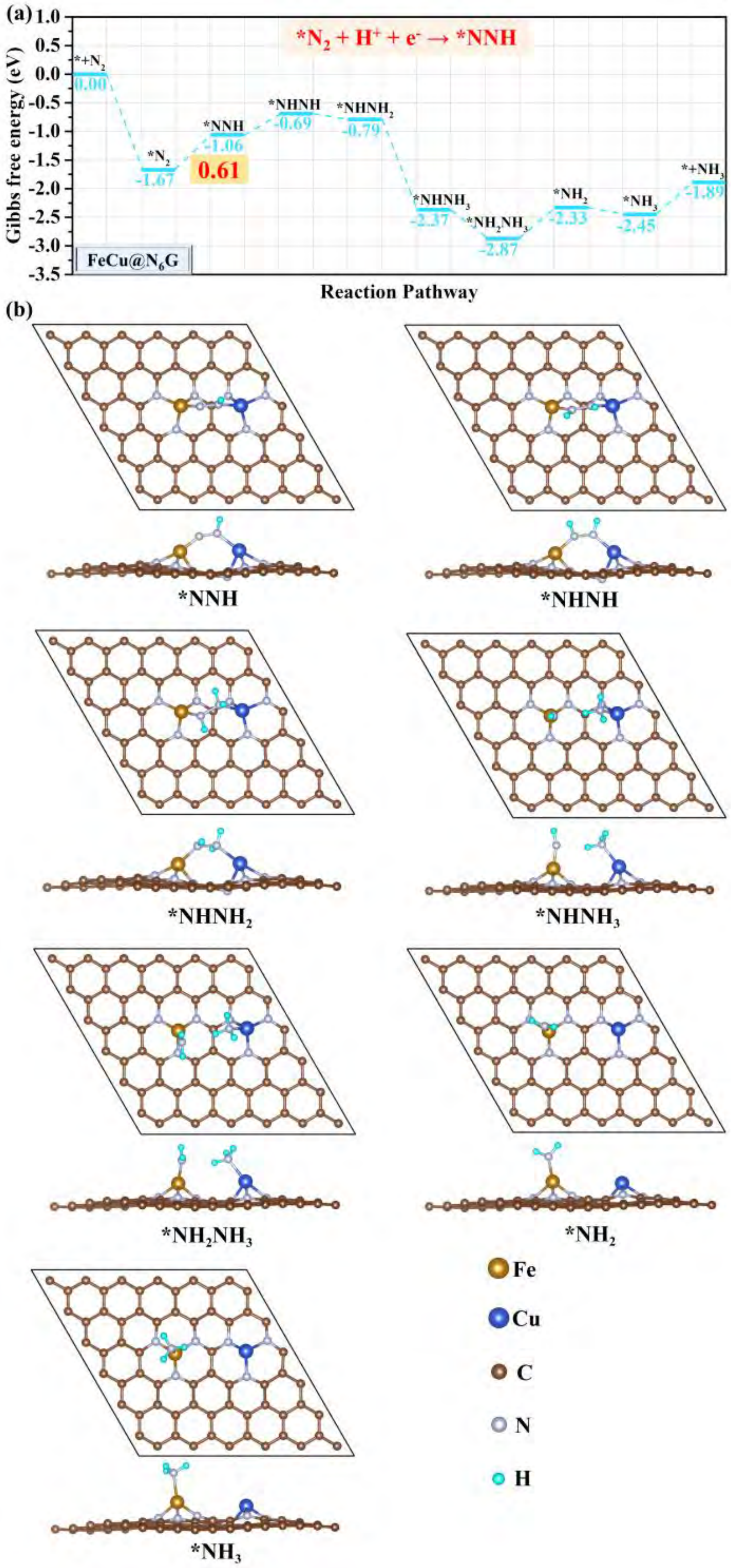

Figure S135. (a) Gibbs free energy diagram for N_2 reduction to NH_3 production on the FeCu@N_6G system. (b) Optimized structures of various intermediates along the hydrogenation pathway of N_2 reduction to NH_3 production on the FeCu@N_6G system.

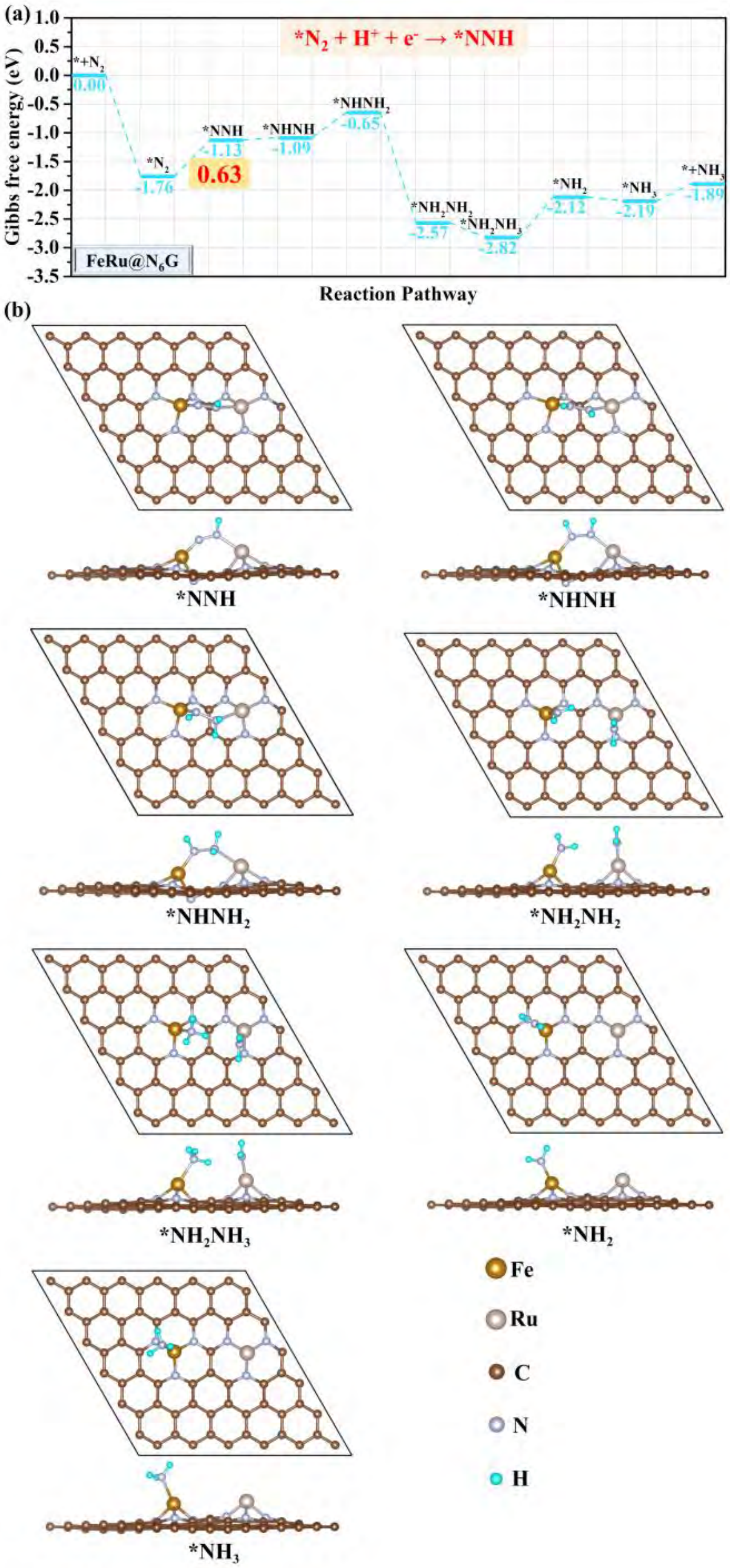

Figure S136. (a) Gibbs free energy diagram for N_2 reduction to NH_3 production on the FeRu@N$_6$G system. (b) Optimized structures of various intermediates along the hydrogenation pathway of N_2 reduction to NH_3 production on the FeRu@N$_6$G system.

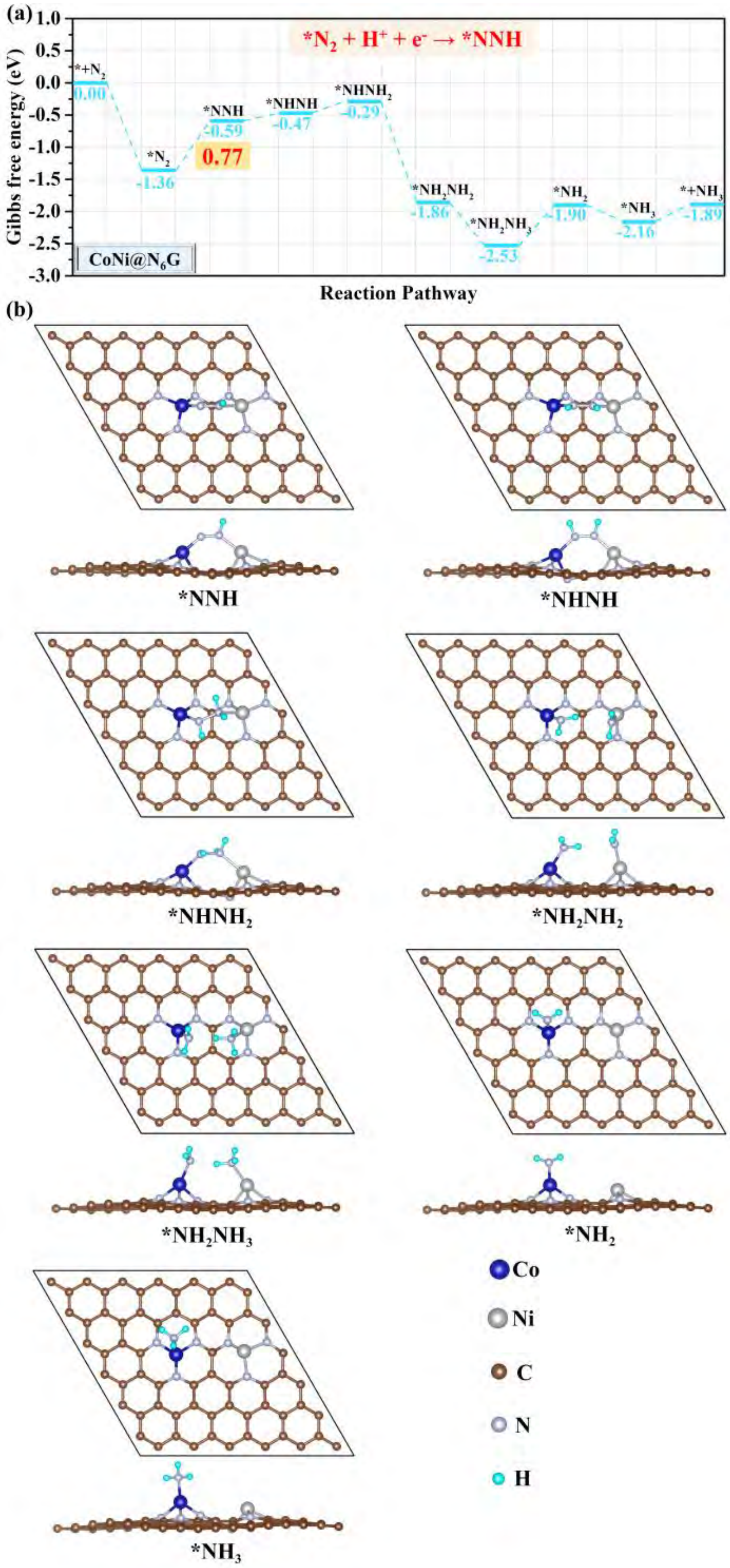

Figure S137. (a) Gibbs free energy diagram for N_2 reduction to NH_3 production on the CoNi@N_6G system. (b) Optimized structures of various intermediates along the hydrogenation pathway of N_2 reduction to NH_3 production on the CoNi@N_6G system.

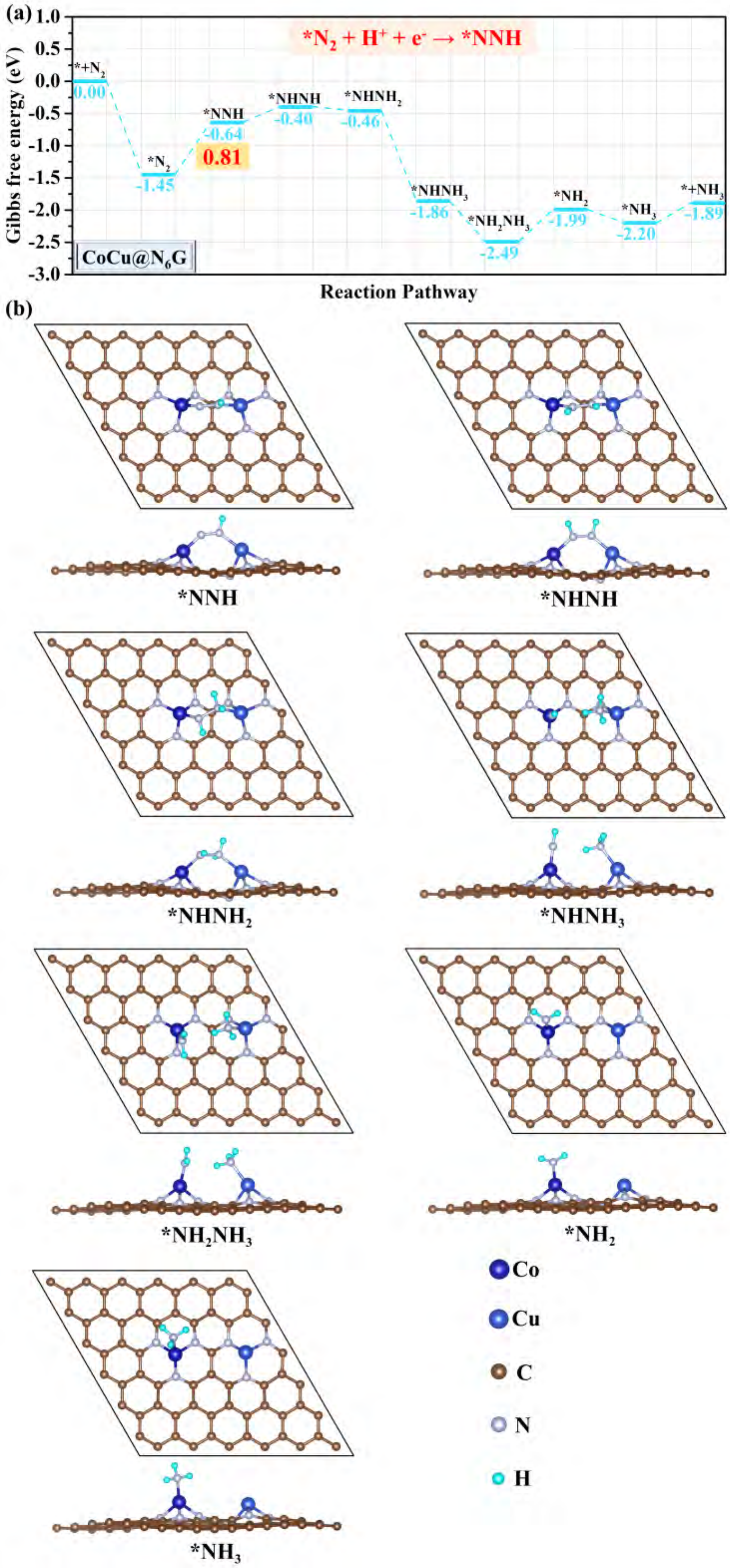

Figure S138. (a) Gibbs free energy diagram for N_2 reduction to NH_3 production on the CoCu@N_6G system. (b) Optimized structures of various intermediates along the hydrogenation pathway of N_2 reduction to NH_3 on the CoCu@N_6G system.

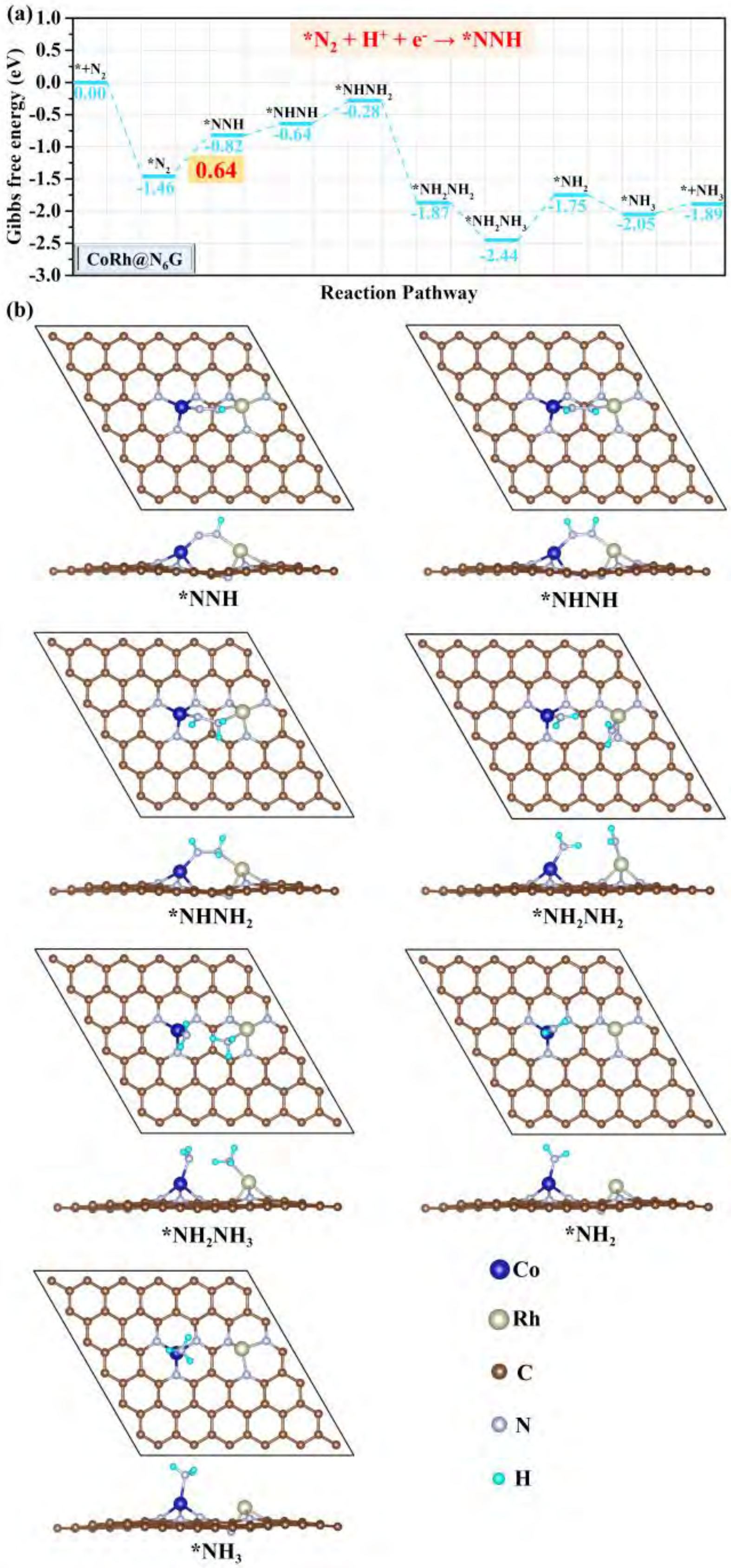

Figure S139. (a) Gibbs free energy diagram for N_2 reduction to NH_3 production on the CoRh@N_6G system. (b) Optimized structures of various intermediates along the hydrogenation pathway of N_2 reduction to NH_3 on the CoRh@N_6G system.

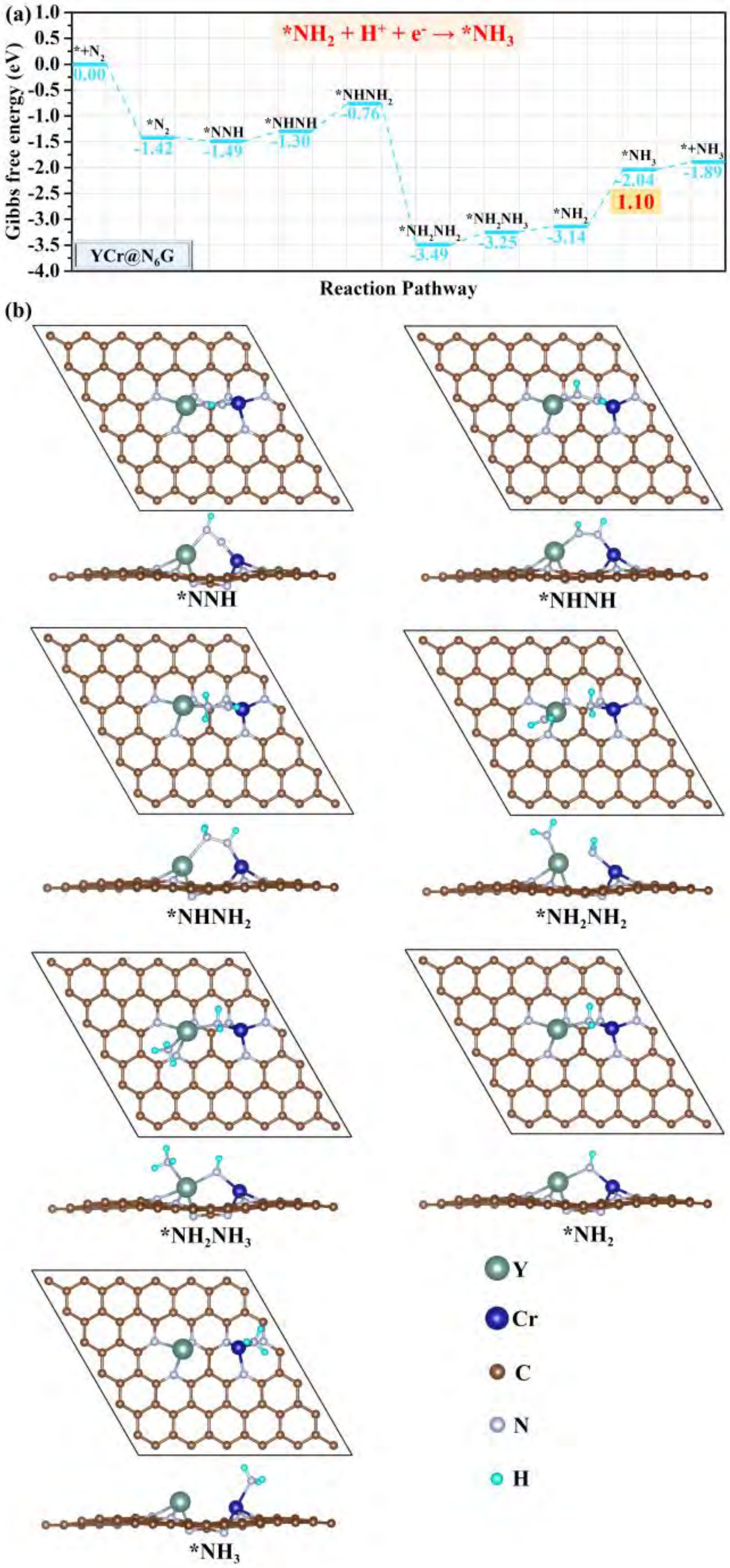

Figure S140. (a) Gibbs free energy diagram for N_2 reduction to NH_3 production on the YCr@N_6G system. (b) Optimized structures of various intermediates along the hydrogenation pathway of N_2 reduction to NH_3 production on the YCr@N_6G system.

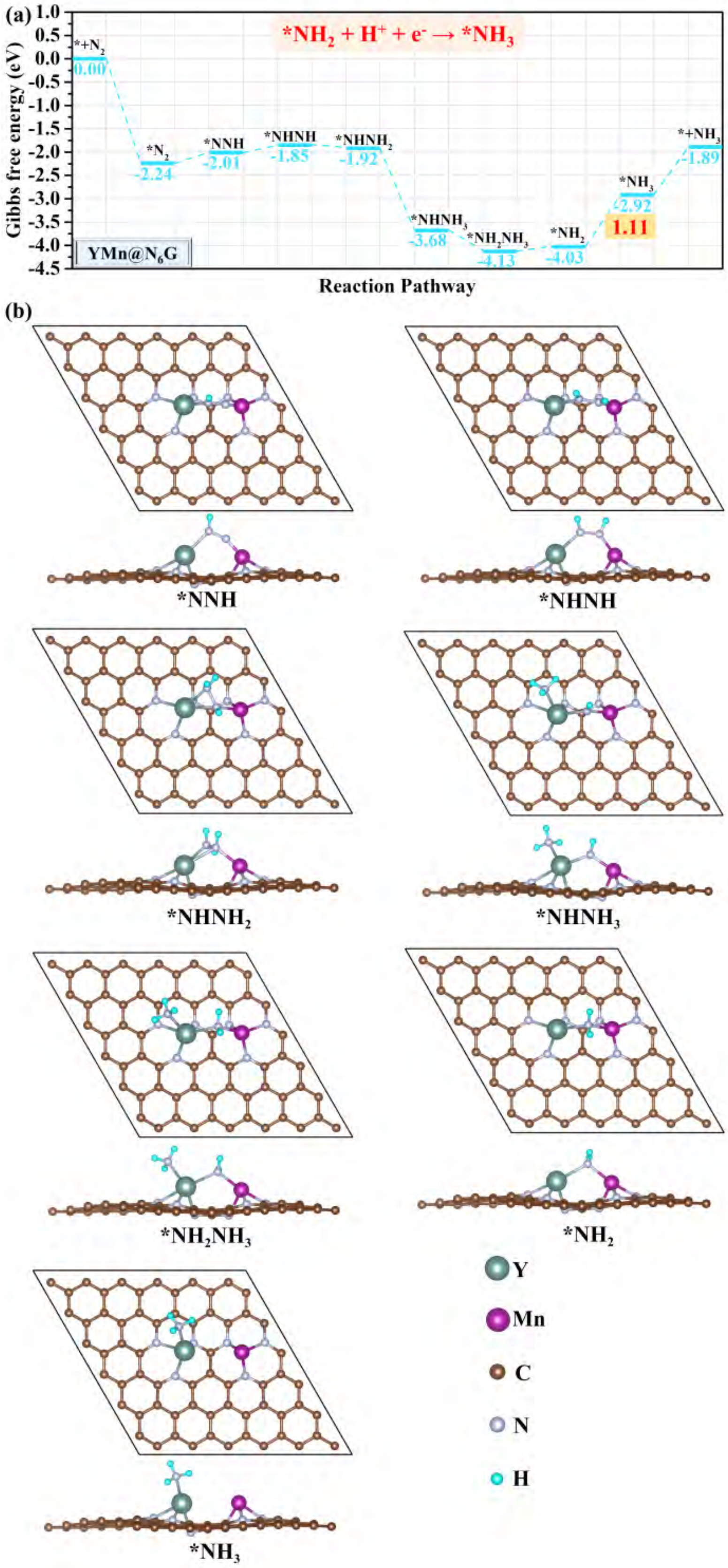

Figure S141. (a) Gibbs free energy diagram for N_2 reduction to NH_3 production on the YMn@N$_6$G system. (b) Optimized structures of various intermediates along the hydrogenation pathway of N_2 reduction to NH_3 production on the YMn@N$_6$G system.

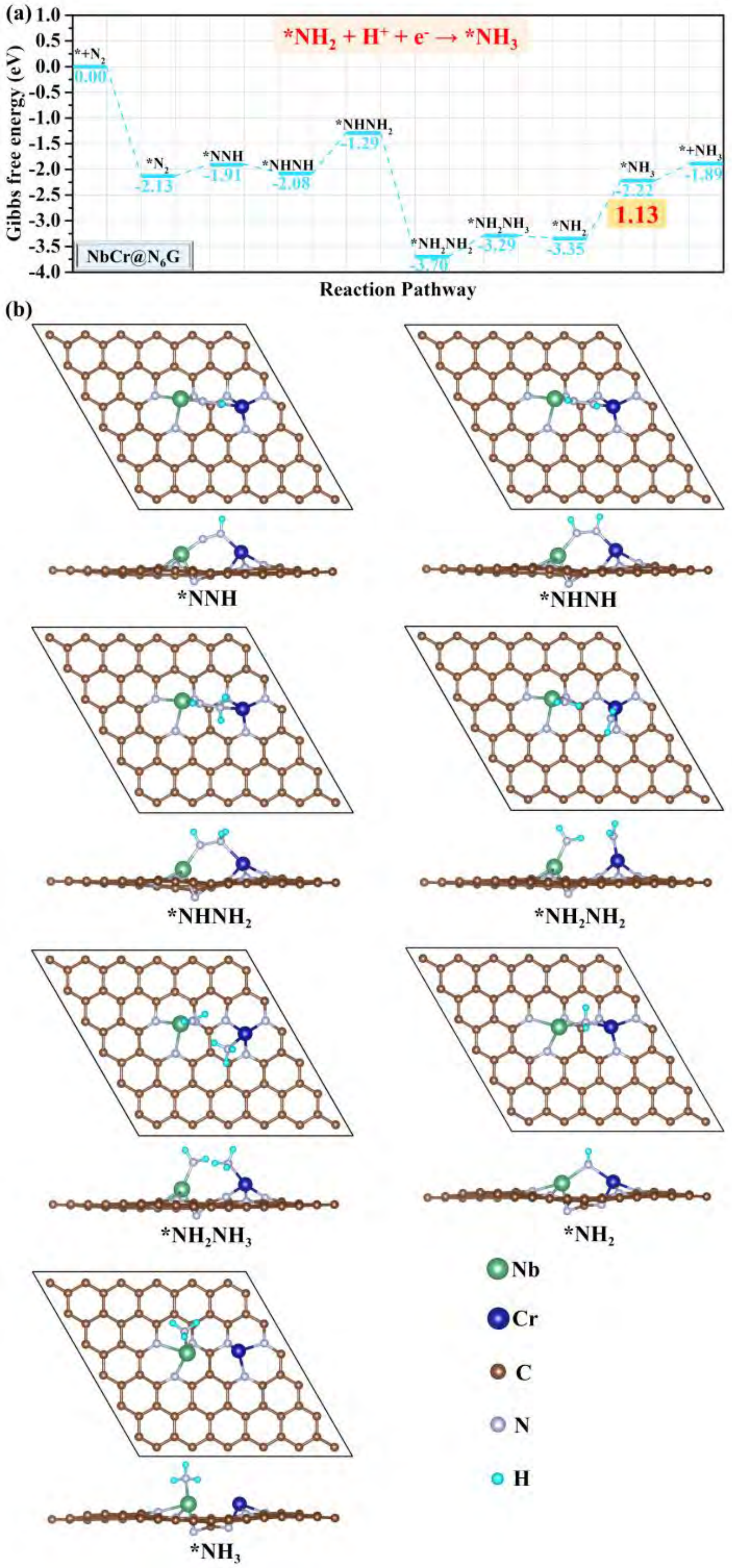

Figure S142. (a) Gibbs free energy diagram for N_2 reduction to NH_3 production on the NbCr@N_6G system. (b) Optimized structures of various intermediates along the hydrogenation pathway of N_2 reduction to NH_3 on the NbCr@N_6G system.

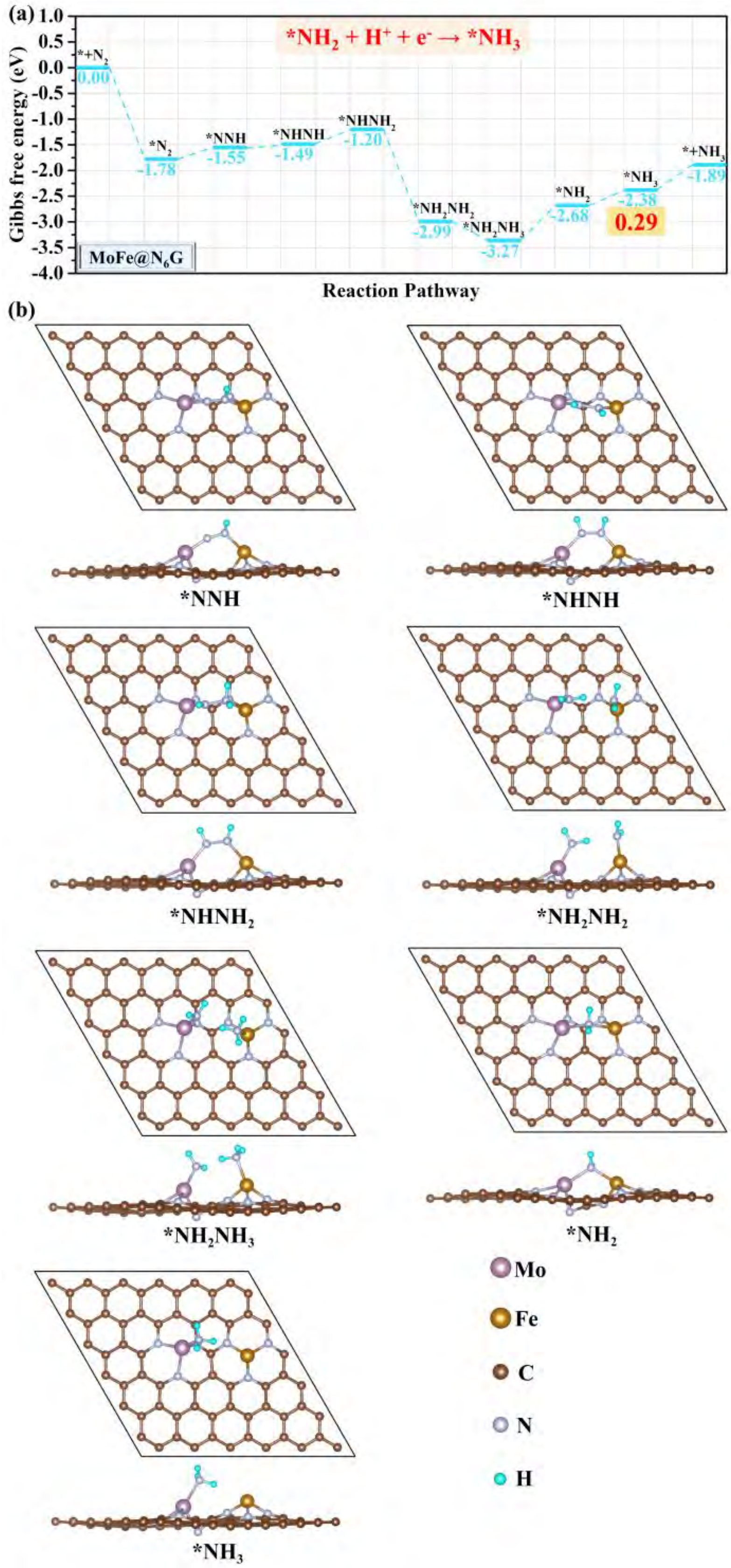

Figure S143. (a) Gibbs free energy diagram for N_2 reduction to NH_3 production on the MoFe@N_6G system. (b) Optimized structures of various intermediates along the hydrogenation pathway of N_2 reduction to NH_3 on the MoFe@N_6G system.

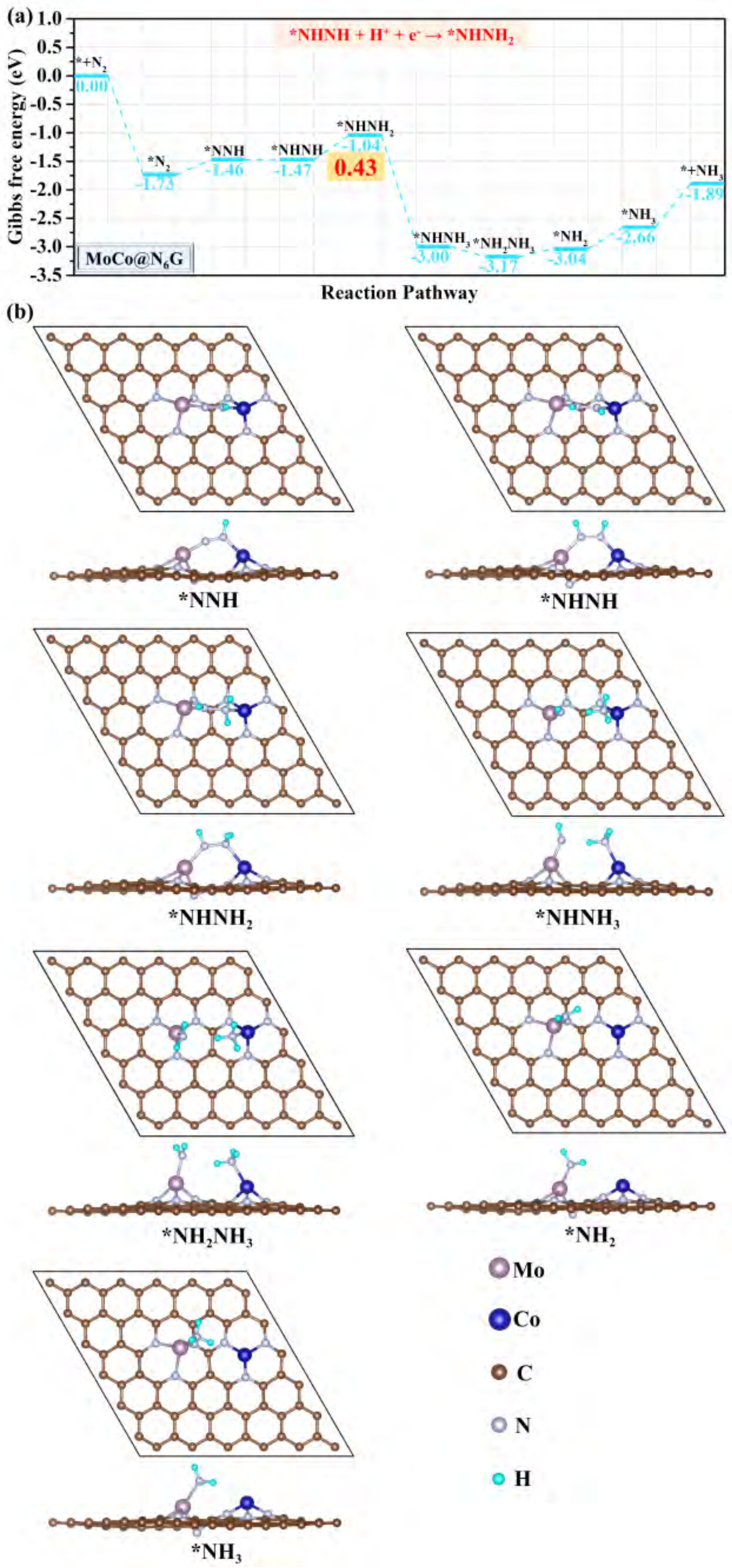

Figure S144. (a) Gibbs free energy diagram for N_2 reduction to NH_3 production on the MoCo@N_6G system. (b) Optimized structures of various intermediates along the hydrogenation pathway of N_2 reduction to NH_3 on the MoCo@N_6G system.

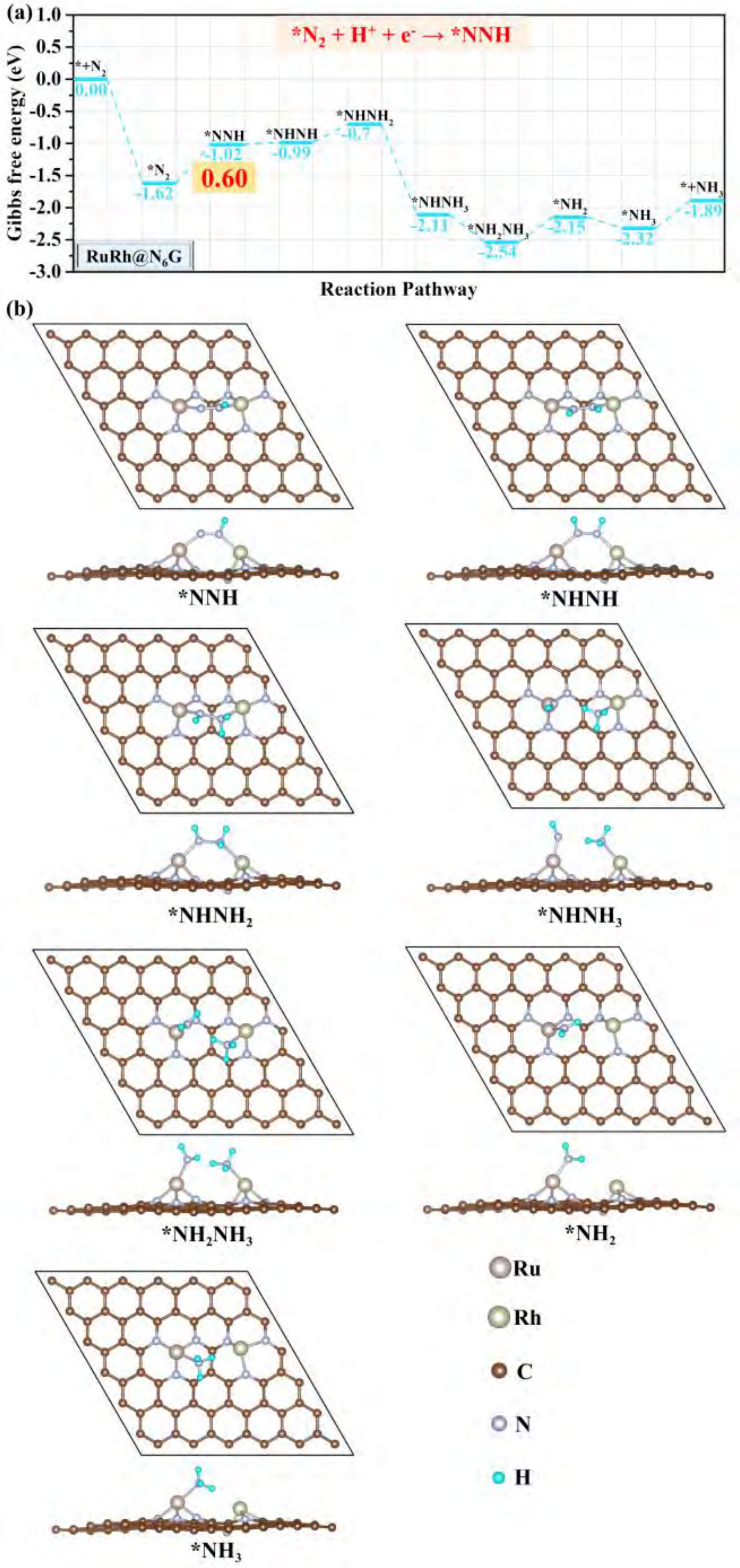

Figure S145. (a) Gibbs free energy diagram for N_2 reduction to NH_3 production on the RuRh@N$_6$G system. (b) Optimized structures of various intermediates along the hydrogenation pathway of N_2 reduction to NH_3 on the RuRh@N$_6$G system.

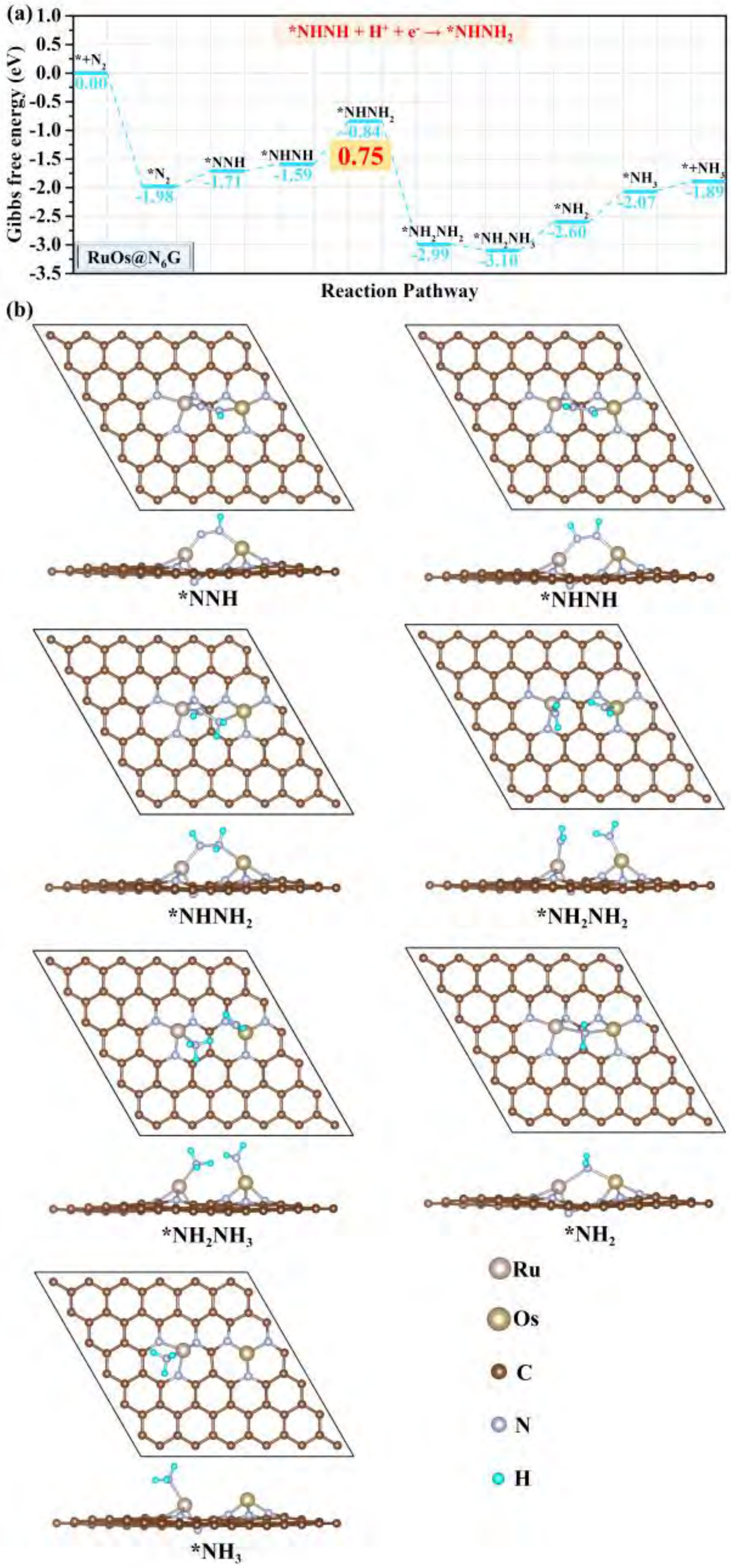

Figure S146. (a) Gibbs free energy diagram for N_2 reduction to NH_3 production on the RuOs@N_6G system. (b) Optimized structures of various intermediates along the hydrogenation pathway of N_2 reduction to NH_3 on the RuOs@N_6G system.

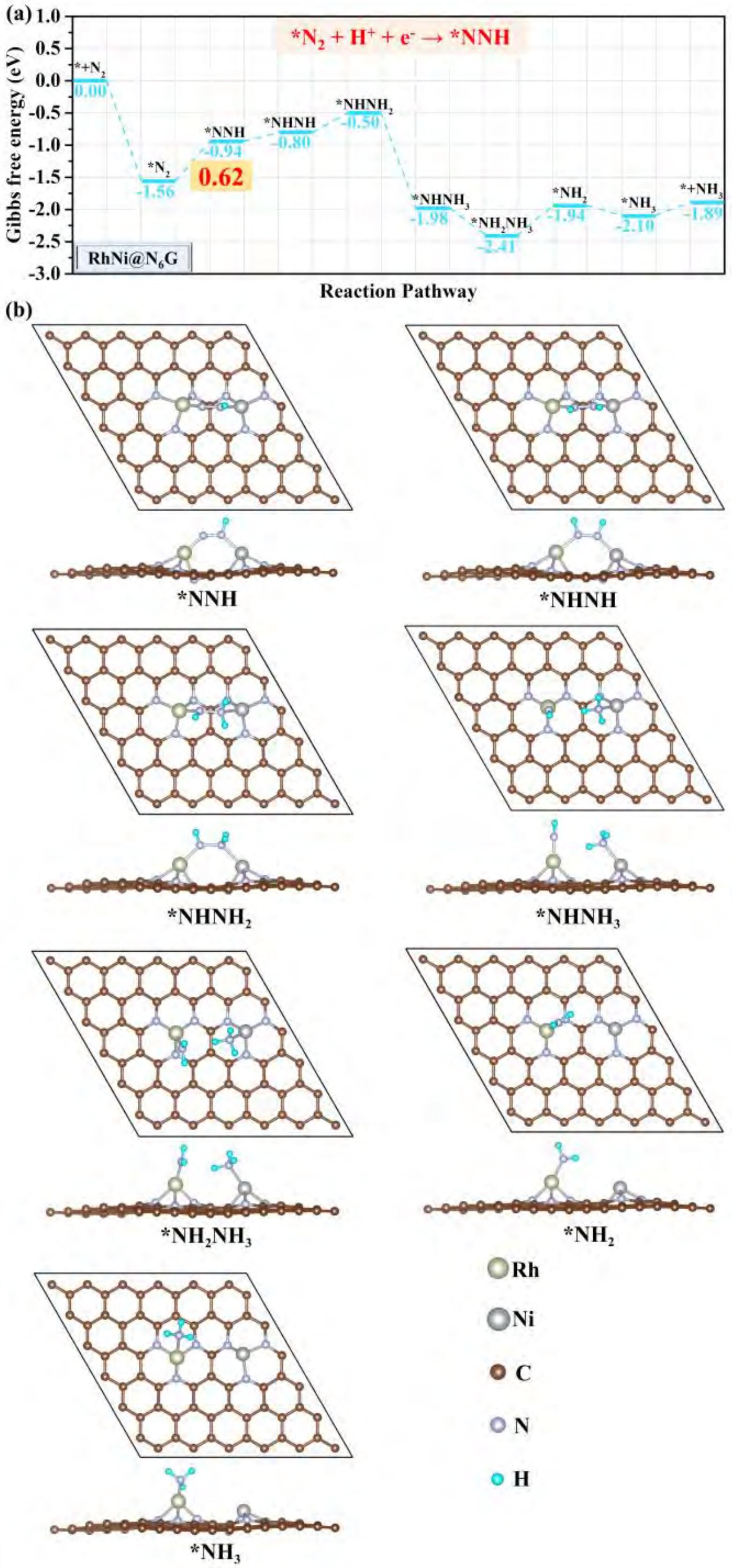

Figure S147. (a) Gibbs free energy diagram for N_2 reduction to NH_3 production on the RhNi@N_6G system. (b) Optimized structures of various intermediates along the hydrogenation pathway of N_2 reduction to NH_3 production on the RhNi@N_6G system.

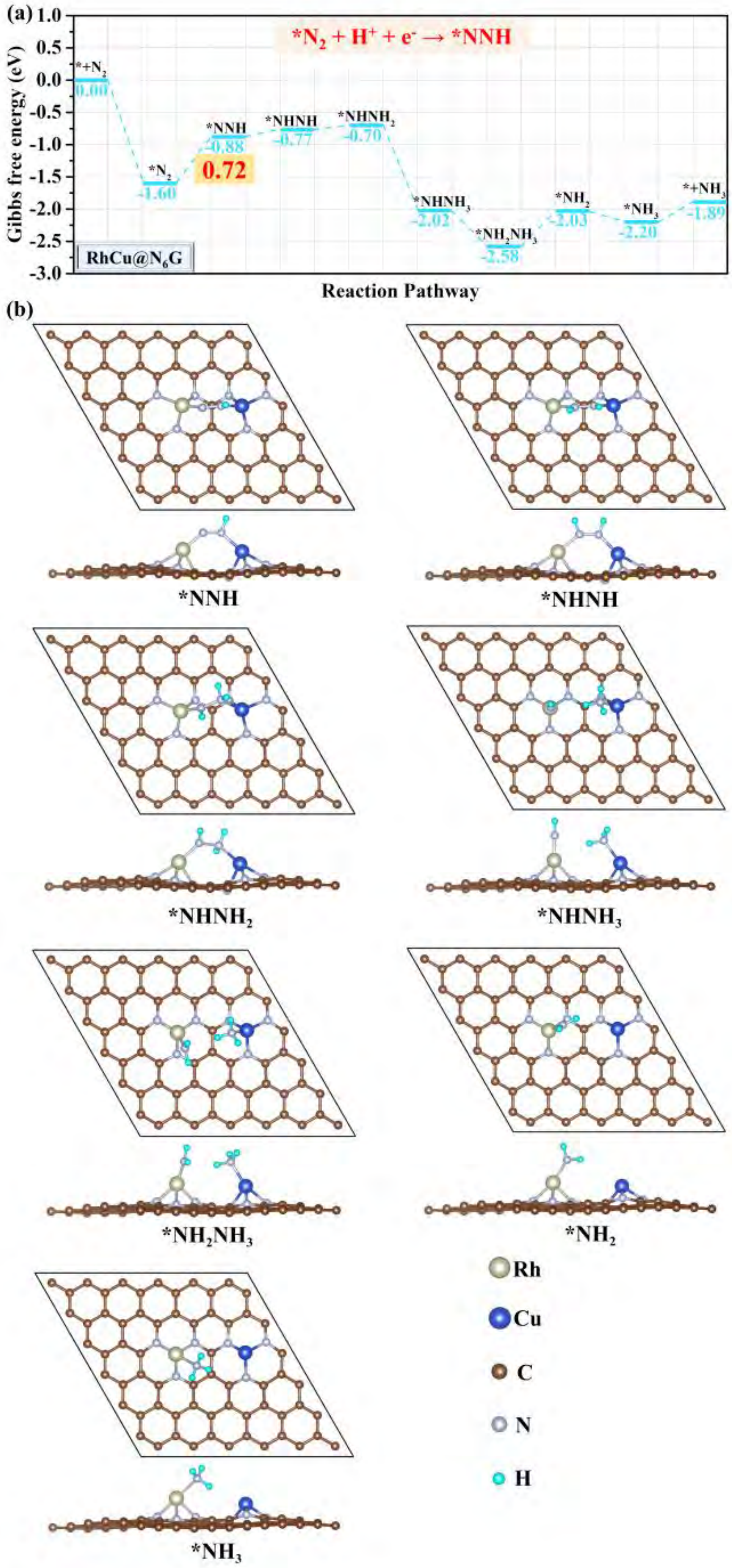

Figure S148. (a) Gibbs free energy diagram for N_2 reduction to NH_3 production on the RhCu@N_6G system. (b) Optimized structures of various intermediates along the hydrogenation pathway of N_2 reduction to NH_3 on the RhCu@N_6G system.

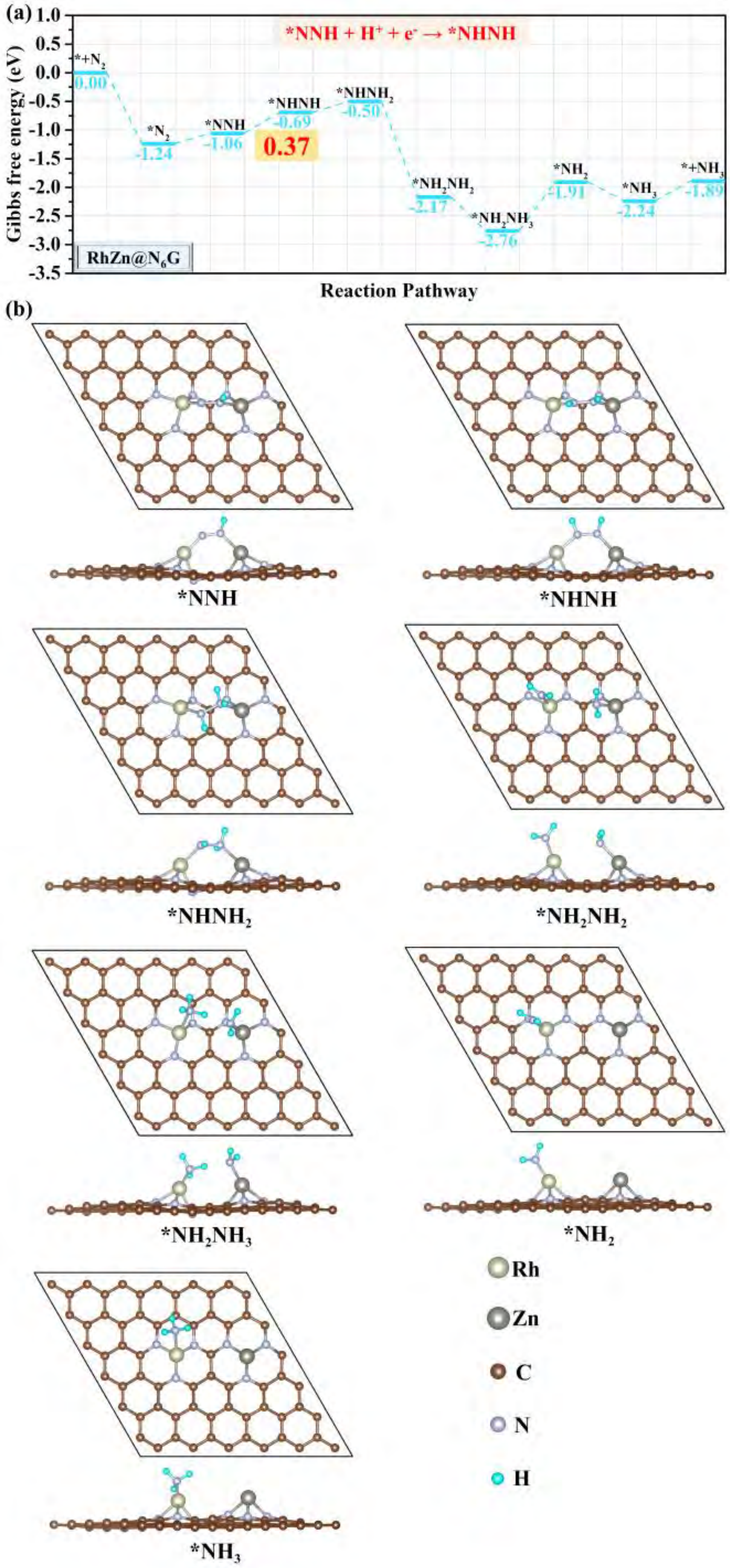

Figure S149. (a) Gibbs free energy diagram for N_2 reduction to NH_3 production on the RhZn@N_6G system. (b) Optimized structures of various intermediates along the hydrogenation pathway of N_2 reduction to NH_3 on the RhZn@N_6G system.

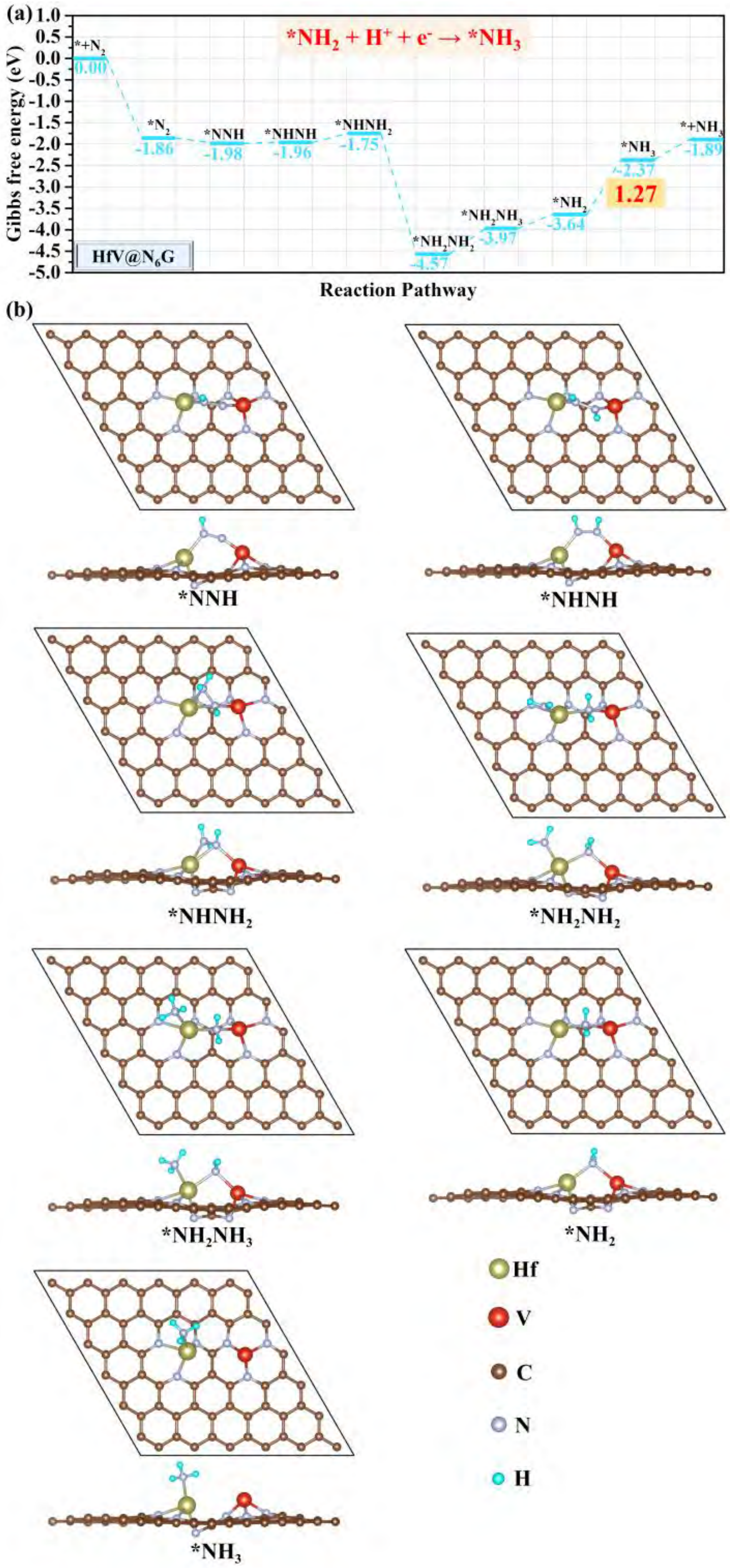

Figure S150. (a) Gibbs free energy diagram for N_2 reduction to NH_3 production on the HfV@N_6G system. (b) Optimized structures of various intermediates along the hydrogenation pathway of N_2 reduction to NH_3 production on the HfV@N_6G system.

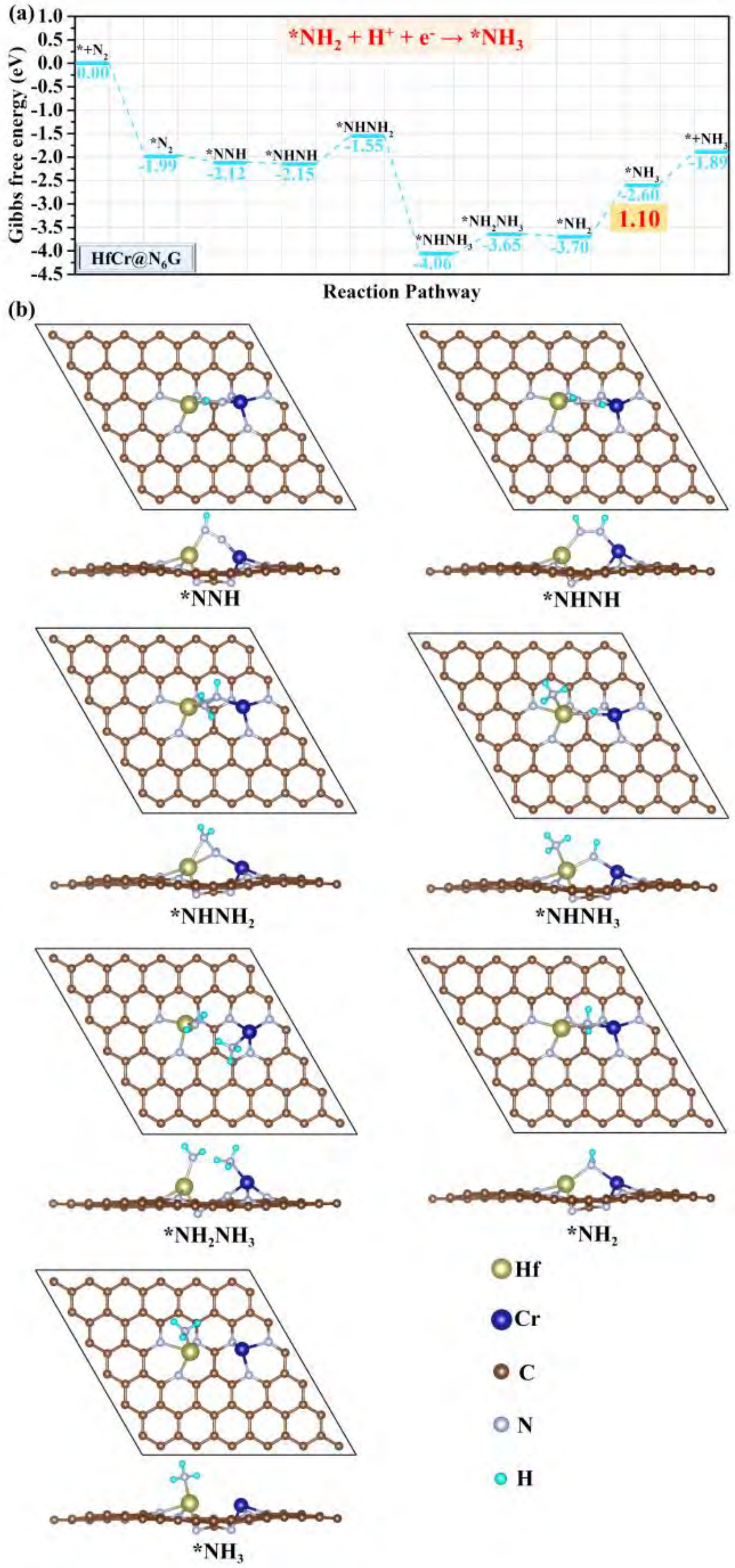

Figure S151. (a) Gibbs free energy diagram for N_2 reduction to NH_3 production on the HfCr@N_6G system. (b) Optimized structures of various intermediates along the hydrogenation pathway of N_2 reduction to NH_3 production on the HfCr@N_6G system.

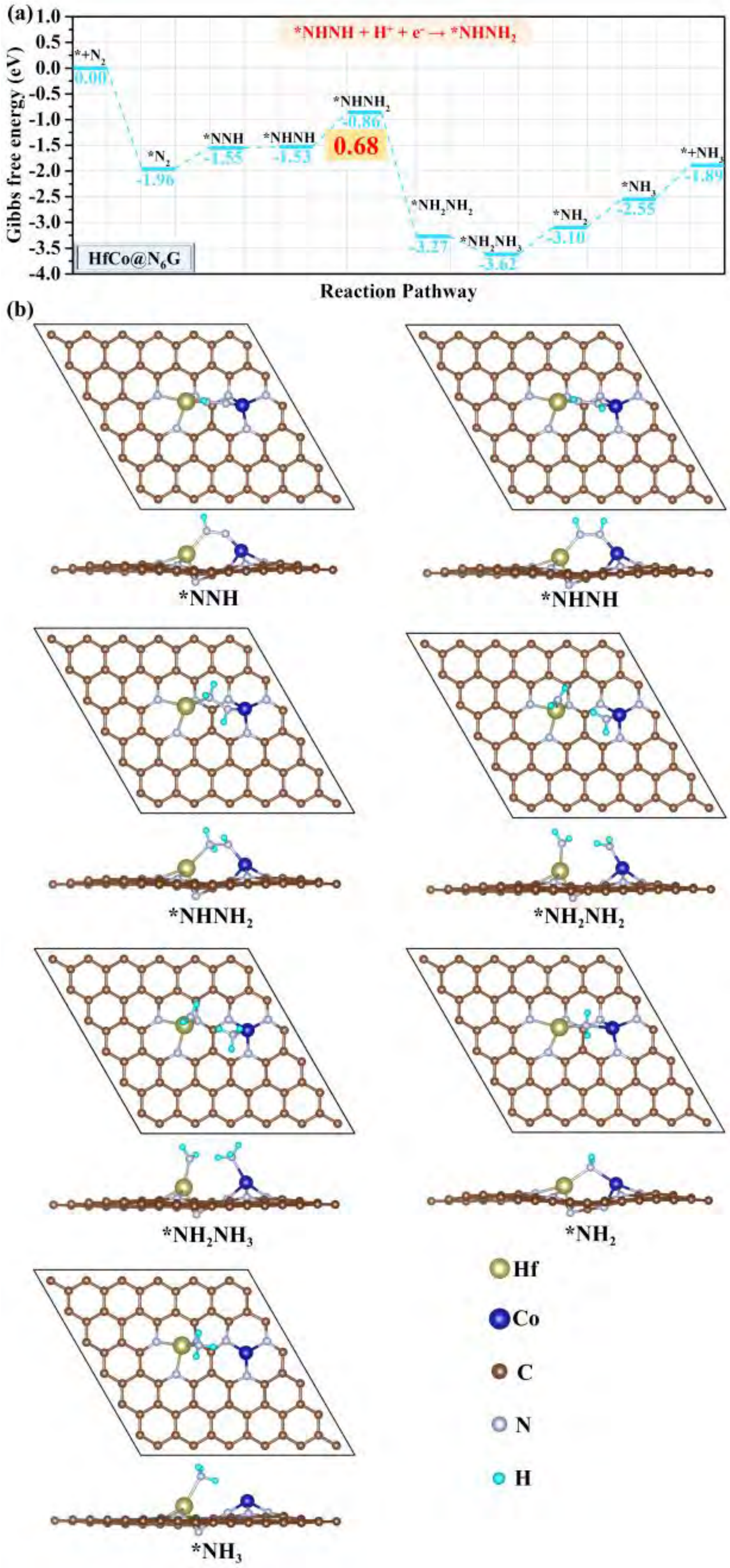

Figure S152. (a) Gibbs free energy diagram for N_2 reduction to NH_3 production on the HfCo@N_6G system. (b) Optimized structures of various intermediates along the hydrogenation pathway of N_2 reduction to NH_3 on the HfCo@N_6G system.

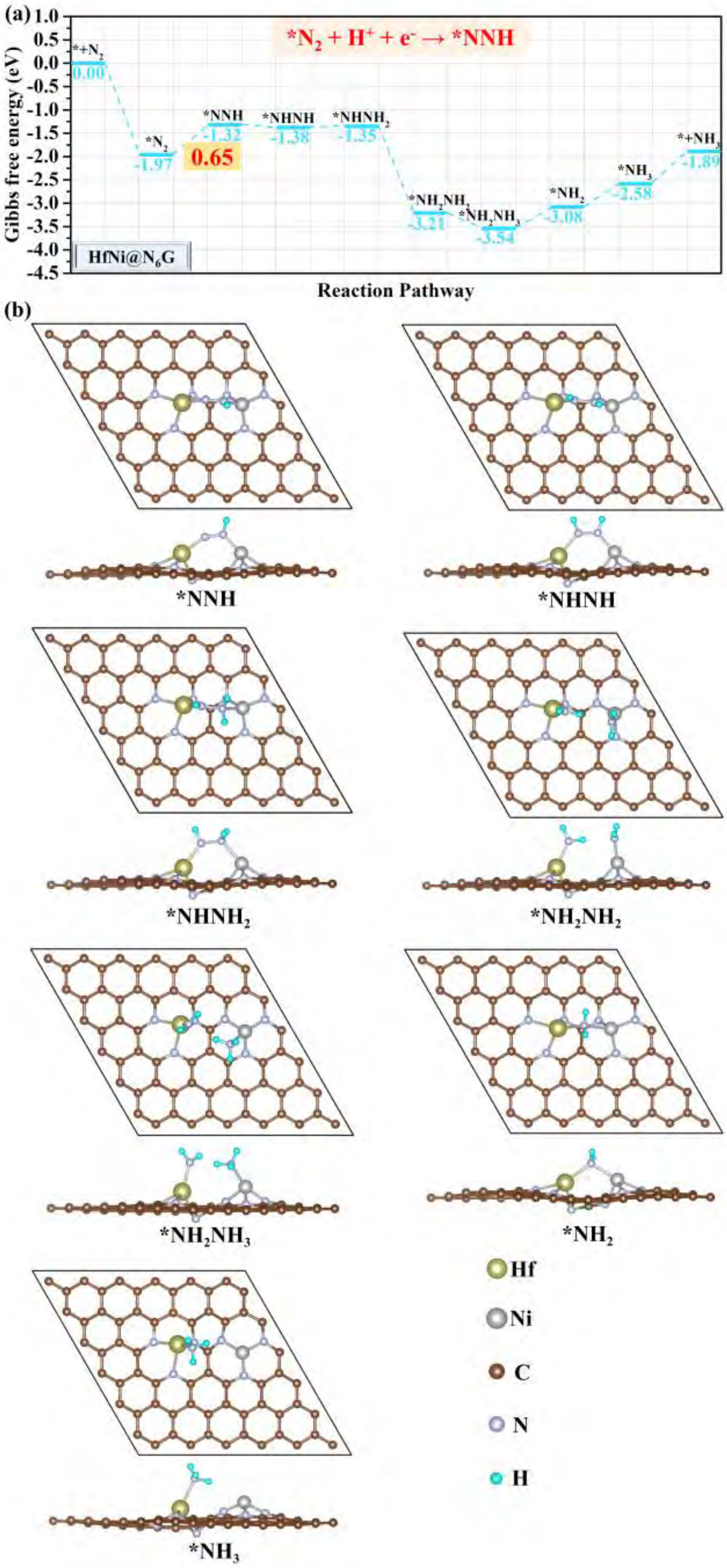

Figure S153. (a) Gibbs free energy diagram for N_2 reduction to NH_3 production on the HfNi@N_6G system. (b) Optimized structures of various intermediates along the hydrogenation pathway of N_2 reduction to NH_3 production on the HfNi@N_6G system.

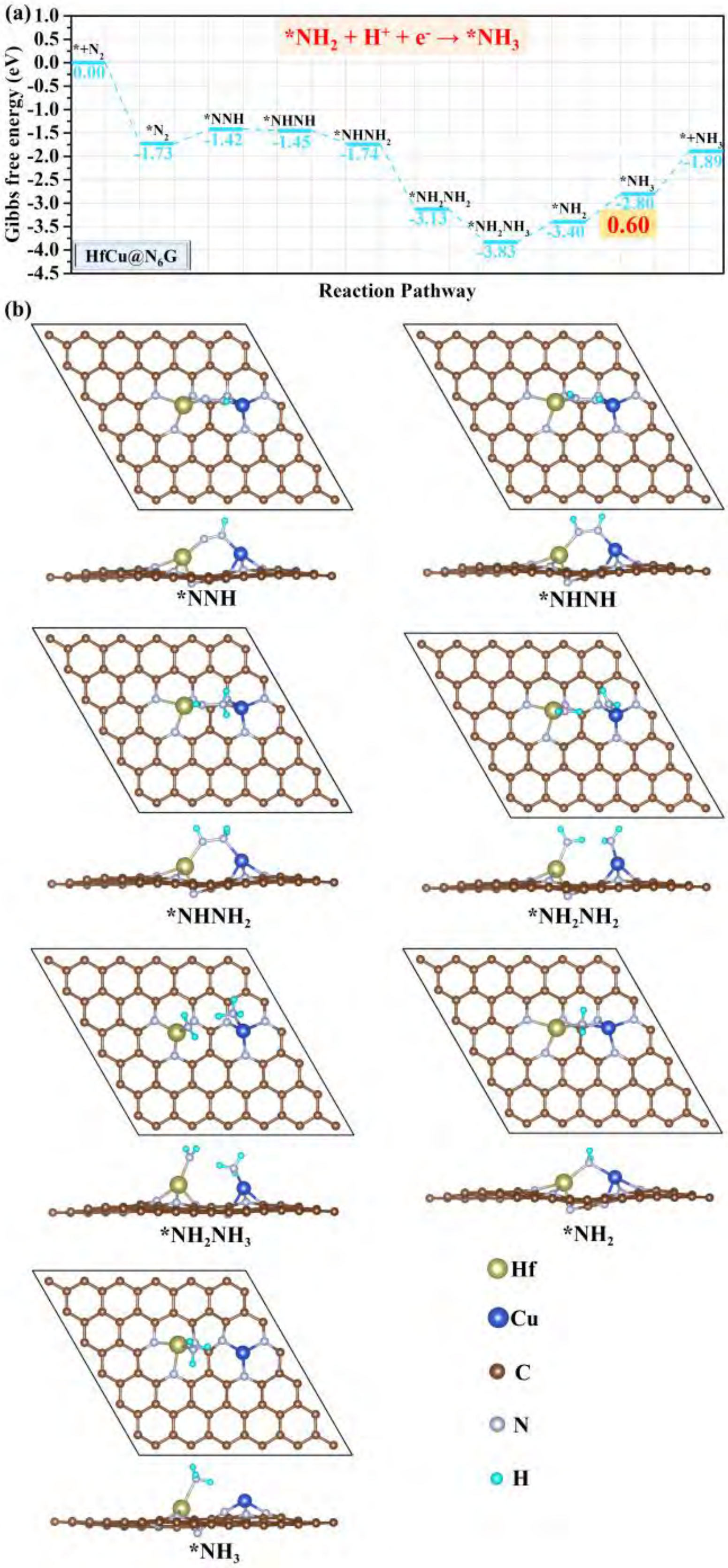

Figure S154. (a) Gibbs free energy diagram for N_2 reduction to NH_3 production on the HfCu@N_6G system. (b) Optimized structures of various intermediates along the hydrogenation pathway of N_2 reduction to NH_3 on the HfCu@N_6G system.

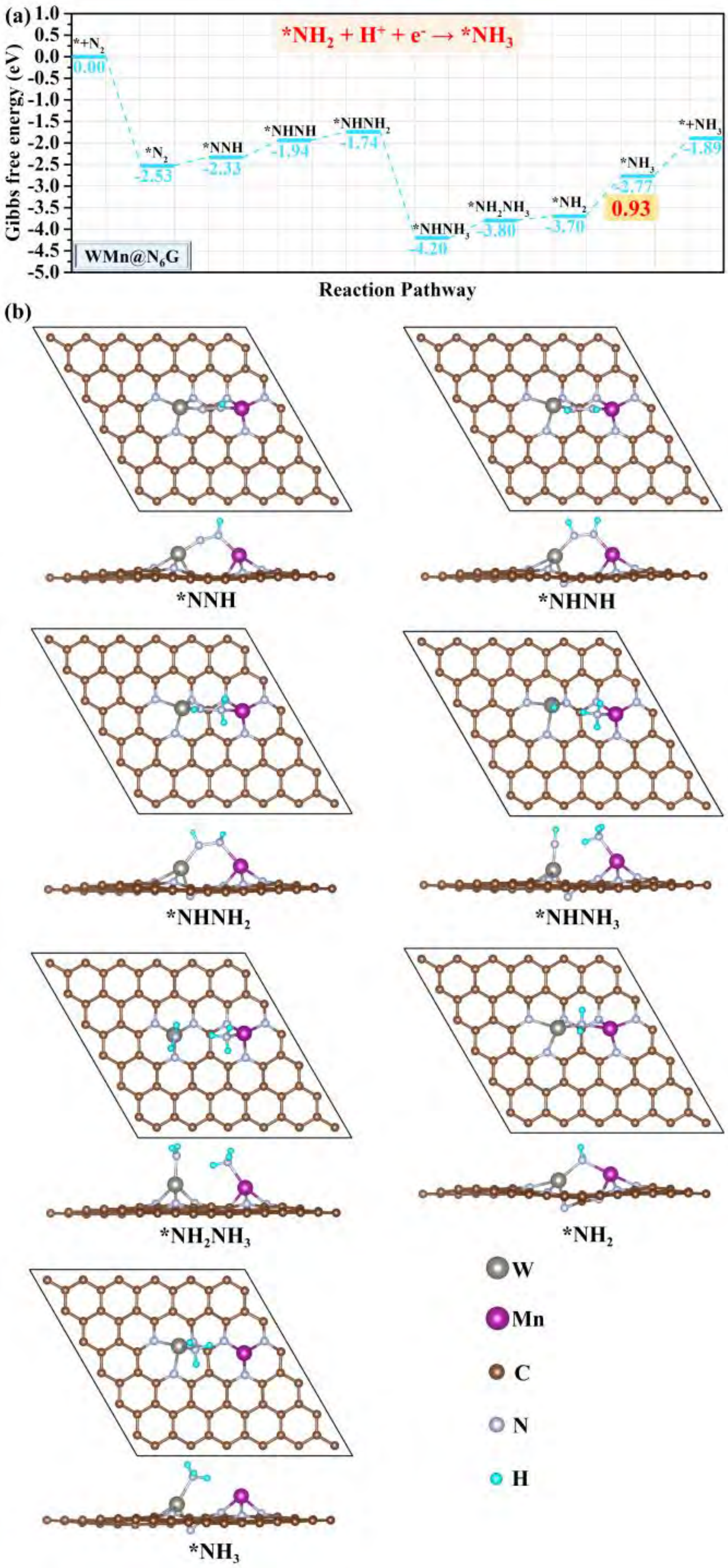

Figure S155. (a) Gibbs free energy diagram for N_2 reduction to NH_3 production on the WMn@N_6G system. (b) Optimized structures of various intermediates along the hydrogenation pathway of N_2 reduction to NH_3 on the WMn@N_6G system.

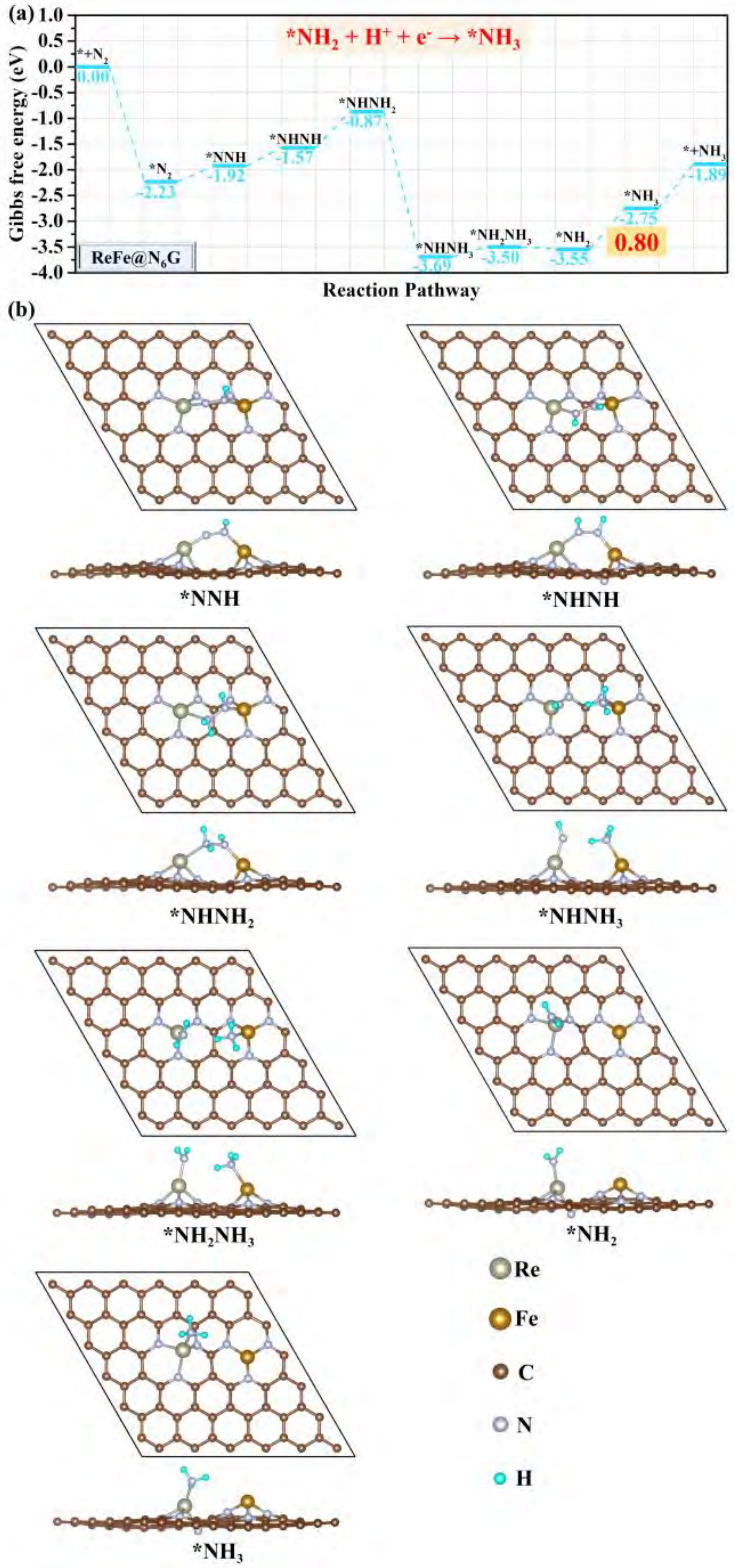

Figure S156. (a) Gibbs free energy diagram for N_2 reduction to NH_3 production on the ReFe@N_6G system. (b) Optimized structures of various intermediates along the hydrogenation pathway of N_2 reduction to NH_3 production on the ReFe@N_6G system.

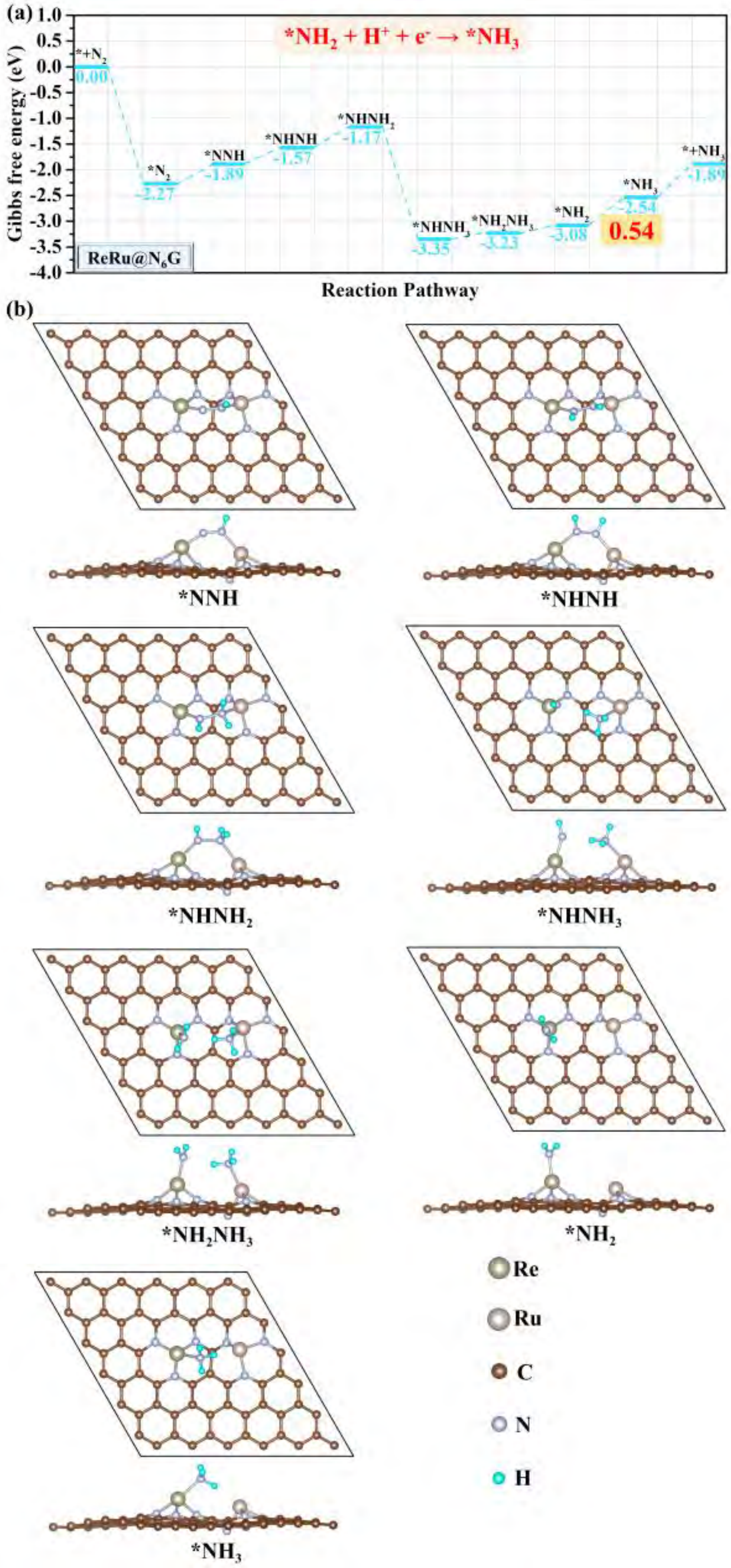

Figure S157. (a) Gibbs free energy diagram for N_2 reduction to NH_3 production on the ReRu@N_6G system. (b) Optimized structures of various intermediates along the hydrogenation pathway of N_2 reduction to NH_3 on the ReRu@N_6G system.

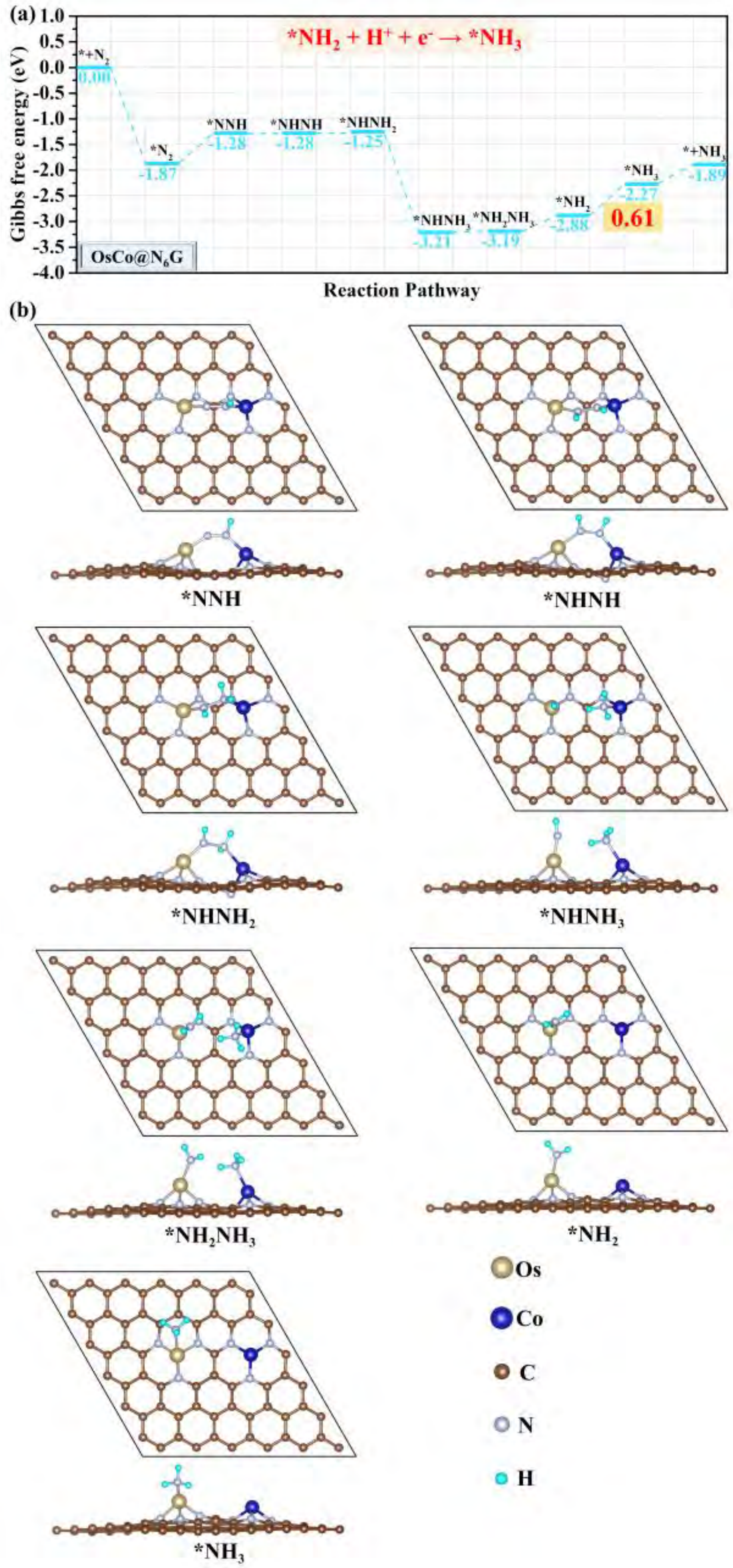

Figure S158. (a) Gibbs free energy diagram for N_2 reduction to NH_3 production on the OsCo@N_6G system. (b) Optimized structures of various intermediates along the hydrogenation pathway of N_2 reduction to NH_3 on the OsCo@N_6G system.

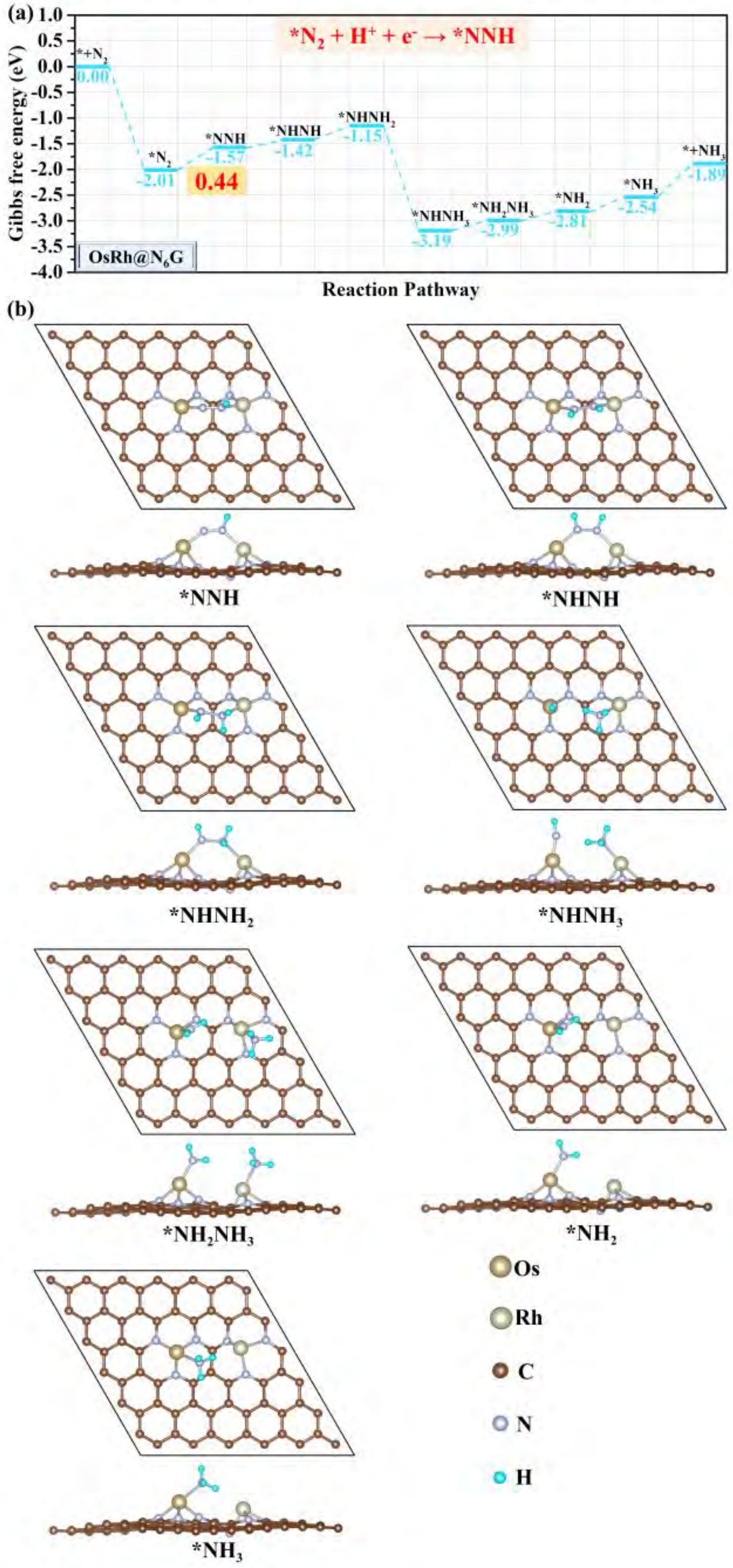

Figure S159. (a) Gibbs free energy diagram for N_2 reduction to NH_3 production on the OsRh@N_6G system. (b) Optimized structures of various intermediates along the hydrogenation pathway of N_2 reduction to NH_3 on the OsRh@N_6G system.

References

[1] M. A. Hunter, J. M. T. A. Fischer, Q. Yuan, M. Hankel, D. J. Searles, *ACS Catal.* **2019**, *9*, 7660.

[2] a) X. Guo, S. Lin, J. Gu, S. Zhang, Z. Chen, S. Huang, *ACS Catal.* **2019**, *9*, 11042; b) X. Guo, J. Gu, S. Lin, S. Zhang, Z. Chen, S. Huang, *J. Am. Chem. Soc.* **2020**, *142*, 5709.

[3] J. Greeley, J. K. Nørskov, *Electrochim. Acta* **2007**, *52*, 5829.

[4] W. Pei, S. Zhou, J. Zhao, X. Xu, Y. Du, S.X. Dou, *Nano Energy* **2020**, *76*, 105049.

[5] K. Yuan, D. Lützenkirchen-Hecht, L. Li, L. Shuai, Y. Li, R. Cao, M. Qiu, X. Zhuang, M. K. H. Leung, Y. Chen, U. Scherf, *J. Am. Chem. Soc.* **2020**, *142*, 2404.

[6] H. Yang, Y. Liu, Y. Luo, S. Lu, B. Su, J. Ma, *ACS Sustainable Chem. Eng.* **2020**, *8*, 12809.

[7] W. Zang, T. Sun, T. Yang, S. Xi, M. Waqar, Z. Kou, Z. Lyu, Y. P. Feng, J. Wang, S. J. Pennycook, *Adv. Mater.* **2021**, *33*, 2003846.

[8] Z. Geng, Y. Liu, X. Kong, P. Li, K. Li, Z. Liu, J. Du, M. Shu, R. Si, J. Zeng, *Adv. Mater.* **2018**, *30*, 1803498.

[9] a) J. K. Nørskov, J. Rossmeisl, A. Logadottir, L. Lindqvist, J. R. Kitchin, T. Bligaard, H. Jonsson, *J. Phys. Chem. B* **2004**, *108*, 17886; b) J. Rossmeisl, A. Logadottir, J. K. Nørskov, *Chem. Phys.* **2005**, *319*, 178.

[10] a) Y. Wang, H. Yuan, Y. Li, Z. Chen, *Nanoscale* **2015**, *7*, 11633; b) X. Zou, L. Wang, B. I. Yakobson, *Nanoscale* **2018**, *10*, 1129; c) V. Wang, N. Xu, J. C. Liu, G. Tang, W. T. Geng, *Comput. Phys. Commun.* **2021**, *267*, 108033.

[11] M. Sun, T. Wu, A. W. Dougherty, M. Lam, B. Huang, Y. Li, C. H. Yan, *Adv. Energy Mater.* **2021**, *11*, 2003796.

[12] M. Huang, S. Fabris, *J. Phys. Chem. C*, **2008**, *112*, 8643.

[13] Z. Hu, H. Metiu, *J. Phys. Chem. C* **2011**, *115*, 5841.

[14] a) Z. Zhang, J. Xiao, X. J. Chen, S. Yu, L.Yu, R. Si, Y. Wang, S. Wang, X. Meng, Y. Wang, Z. Q. Tian, D. Deng, *Angew. Chem., Int. Ed.* **2018**, *57*, 16339; b) Y. Wang, Y. J. Tang, K. Zhou, *J. Am. Chem. Soc.* **2019**, *141*, 14115.

[15] X. Guo, S. Lin, J. Gu, S. Zhang, Z. Chen, S. Huang, *Adv. Funct. Mater.* **2021**, *31*, 2008056.
